# Merging nucleophilic phosphine catalysis and photocatalysis for the rapid assembly of 2-oxabicyclo-[2.1.1]hexane scaffolds from feedstock allyl alcohols†

**DOI:** 10.1039/d4sc06684g

**Published:** 2024-11-04

**Authors:** David M. Whalley, Luca Carlino, Okky Dwichandra Putra, Niall A. Anderson, Susannah C. Coote, Olivier Lorthioir

**Affiliations:** a Medicinal Chemistry, Research and Early Development, Oncology R&D AstraZeneca Cambridge CB2 0AA UK david.whalley2@astrazeneca.com olivier.lorthioir@astrazeneca.com; b Early Product Development and Manufacturing, Pharmaceutical Sciences R&D AstraZeneca Pepparedsleden 1, Mölndal SE-43183 Gothenburg Sweden; c Department of Chemistry, University of Bath 1 South, Claverton Down Bath BA2 7AY UK

## Abstract

The previously unreported combination of nucleophilic phosphine catalysis and energy transfer catalysis allows for the rapid construction of structurally distinct 2-oxabicyclo[2.1.1]hexanes (2-oxa-BCH) from readily available building blocks with high atom economy. Previous multistep routes to these important phenyl ring bioisosteres have largely depended on the use of bespoke strain-release agents or on multiple post-functionalisation reactions to access structural diversity of the scaffold. In contrast, this cascade reaction allows the medicinal chemist to exploit the breadth of commercial allyl alcohols to synthesise systematically diverse 2-oxa-BCH architectures. Using a combination of polar and radical disconnections in the same reaction flask, every position of the scaffold can be substituted with useful functional handles such as protected amines, esters and alcohols, as well as arenes and alkyl groups. Cyclic allyl alcohols can even be employed to yield single diastereomers of sp^3^-rich bridged spirocyclic structures. Aromatic groups at the 1-position can be varied to incorporate a plethora of arenes including medicinally relevant heterocycles such as indole, pyrazole and pyridine.

## Abstract 2

A synthesis of 2-oxabicyclo-[2.1.1]hexanes from readily available allyl alcohols and electron-poor arylalkynes. The transformation utilises phosphine catalysis and energy transfer catalysis for rapid assembly of these diverse scaffolds.

## Graphical abstract

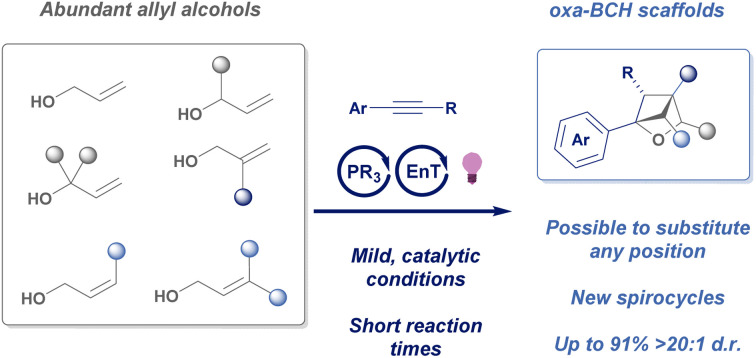

## Introduction

In recent years, there has been an increasing demand for novel sp^3^-rich scaffolds to replace traditional planar arenes in medicinal chemistry campaigns.^1^ Such proposed solutions include 1,3-bicyclo[1.1.1]pentanes (BCP), bicyclo[2.1.1]hexanes (BCH), bicyclo[3.1.1]heptanes (BCHep) and substituted cubanes.^2^ However, it should be noted that the inclusion of a heteroatom in these scaffolds is linked to increased aqueous solubility as well as the potential for increasing drug potency, owing to the ability to form non-covalent interactions with water or the protein of interest respectively.^3^ One such structure that has been relatively underexplored in comparison to its all-carbon counterparts is the 2-oxabicyclo[2.1.1]hexane (2-oxa-BCH).^4^ Whilst initial reports have demonstrated the structure's use as a phenyl ring bioisostere,^5^ a recent study from Peterson concerning the development of a CNS-penetrant IRAK4 inhibitor also found that a 2-oxa-BCH outperformed conventional ether substituents in terms of potency whilst improving microsomal stability and lowering efflux, owing to the removal of a potentially metabolically labile benzylic C–H bond and installation of a quaternary centre.^6^

**Scheme 1:**
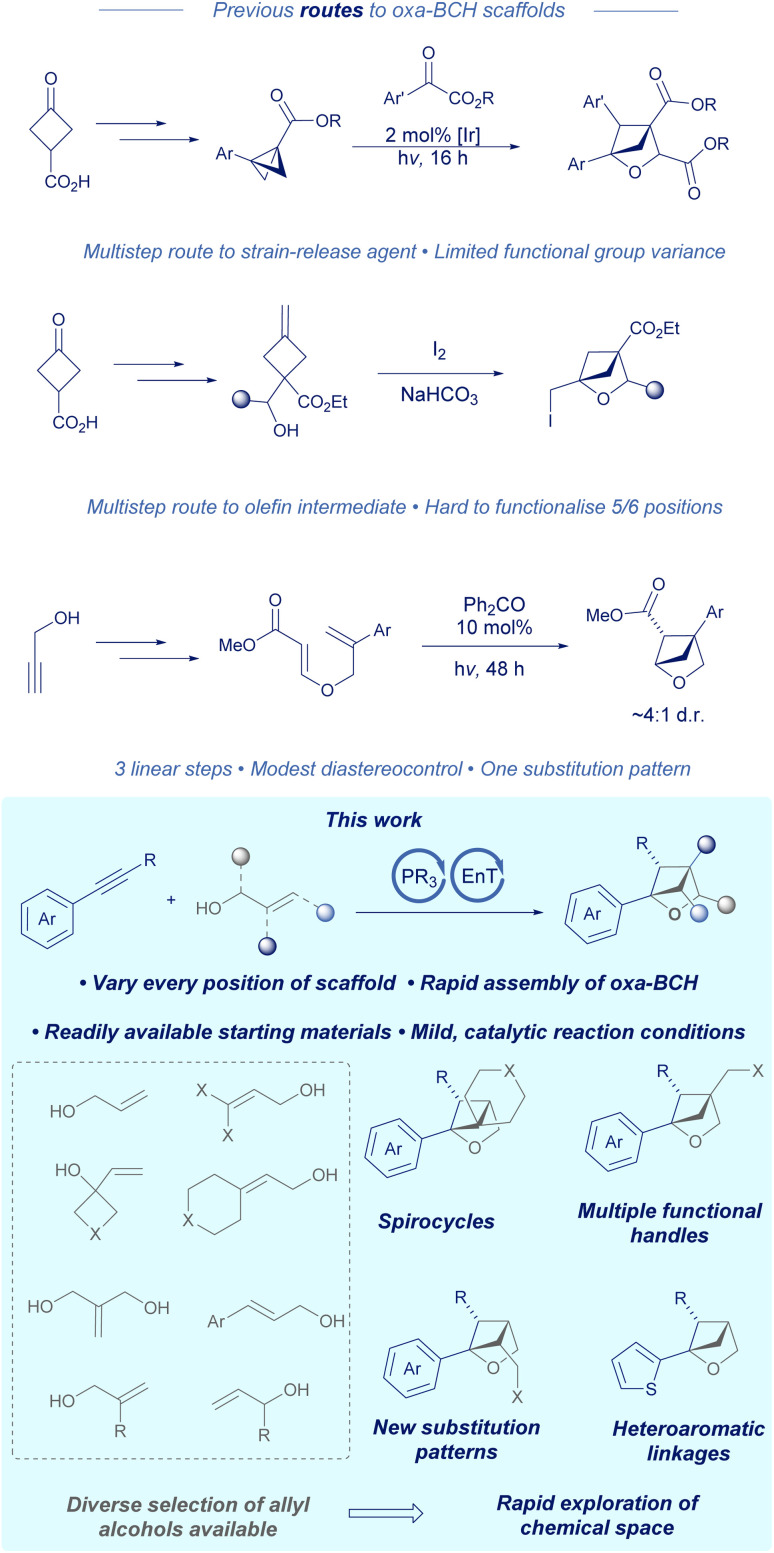
Comparison to previous routes to 2-oxabicyclo[2.1.1]hexanes.

For chemists wishing to synthesise 2-oxa-BCHs, there are a handful of multistep routes (Scheme 1). One method developed by Glorius involves using a bicyclobutane derived from 3-oxocyclobutane-1-carboxylic acid synthesised in four linear steps, followed by a Lewis acid or iridium catalysed cycloaddition with a carbonyl compound to give a tri- or tetra-substituted 2-oxa-BCH.^7^ The pendant ester or ketone group(s) would then need to undergo a post-functionalisation reaction to a suitable synthetic handle. Alternatively, using the work of Mykhailiuk and coworkers, propargylic alcohol can be arylated using Grignard/copper chemistry and then subsequently converted into a styrenyl diene starting material.^8^ Irradiation for 48 hours with a benzophenone catalyst gives a 4,5-disubstituted 2-oxa-BCH in good yield bearing a pendant ester. Lastly, work from the same group describes a different route whereby 3-oxocyclobutane-1-carboxylic acid can be converted to the respective olefin using Wittig chemistry, followed by an iodine mediated alkoxy-iodination reaction to give the 2-oxa-BCH product with a pendant alkyl iodide.^5,9^ Either the initial 3-oxocyclobutane-1-carboxylic acid starting material can be converted in several steps to change the substitution pattern of the scaffold or the alkyl iodide can be derivatised to yield different functional handles.^10^

A common theme in the aforementioned routes is that each method either gives a fixed substitution pattern or requires multistep modifications of the starting material in order to engineer structural diversity. Given that a photochemical [2 + 2] disconnection of the 2-oxa-BCH negates the need for a cyclobutane containing starting material, we sought to design a cascade reaction that could utilise commonly available allyl alcohols as one of the respective olefin synthons, potentially unlocking a wealth of structural diversity and access to every position of the scaffold. We hypothesised that an arylalkyne could serve as a second coupling partner and provide a suitable chromophore for photocycloaddition.

## Results and discussion

We initiated our study with the commercial 3-phenylpropiolonitrile 1 and allyl alcohol 2 under a combination of nucleophilic phosphine catalysis and energy transfer catalysis to mediate a conjugate addition and [2 + 2] cycloaddition respectively.^11,12^ Dichloromethane was identified as the optimal solvent (Table 1 Entry 1–3), providing a quantitative yield of 3a with good diastereoselectivity at 0.20 mmol scale. After confirming that the reaction did not proceed in the presence of an analogous base (Table 1, Entry 6) and that tributylphosphine/thioxanthone was a superior catalyst combination (Table 1, Entries 4–8), we were able to perform the reaction on a 1.0 mmol scale and confirmed the importance of both catalysts and light in the transformation (Table 1, Entries 10–12).

**Table 1:** Reaction optimisation

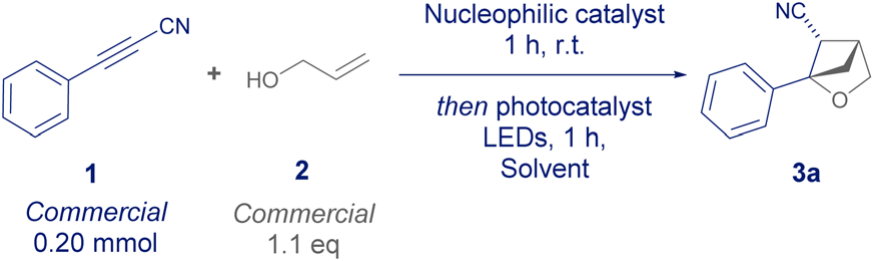				
Entry	Solvent	Nucleophilic catalyst	Photocatalyst	Yield^a^ (d.r.)^b^
1	Acetone	PBu_3_ (10 mol%)	TXO (15 mol%)	26% (6 : 1)
2	Toluene	PBu_3_ (10 mol%)	TXO (15 mol%)	52% (5 : 1)
3	DCM	PBu_3_(10 mol%)	TXO (15 mol%)	>99% (7 : 1)
4	DCM	PMe_3_ (10 mol%)	TXO (15 mol%)	96% (7 : 1)
5	DCM	PPh_3_ (10 mol%)	TXO (15 mol%)	12% (5 : 1)
6	DCM	NEt_3_ (10 mol%)	TXO (15 mol%)	n.d.
7	DCM	PBu_3_ (10 mol%)	[Ir cat] (2 mol%)	40% (8 : 1)
8	DCM	PBu_3_ (10 mol%)	Ph_2_CO(15 mol%)	43% (7 : 1)
**9**	**DCM**	**PBu**_**3**_ **(5 mol%)**	**TXO (5 mol%)**	**86%^c^ (8 : 1)**
10	DCM	PBu_3_ (5 mol%)	—	<5%
11	DCM	—	TXO (5 mol%)	n.d.
12^d^	DCM	PBu_3_ (5 mol%)	TXO (5 mol%)	n.d.

a ^1^H NMR yield against internal standard.

b Determined *via* integration of crude ^1^H NMR spectrum.

c Isolated yield.

d No irradiation. TXO = thioxanthen-9-one (370 nm irradiation); [Ir cat] = Ir(dFCF_3_ppy)_2_(dtbbpy)PF_6_ (456 nm irradiation); Ph_2_CO (370 nm irradiation).

Whilst it is a common strategy to synthesise polyene precursors in a multistep process followed by a radical cyclisation cascade,^13^ to the best of our knowledge, this protocol is the first example to demonstrate the compatibility of nucleophilic phosphine catalysts with energy transfer catalysis.^14^ Moreover, the lack of stoichiometric reagents and modest equivalents of allyl alcohol renders the reaction highly atom economical and enables straightforward purification.

**Scheme 2:**
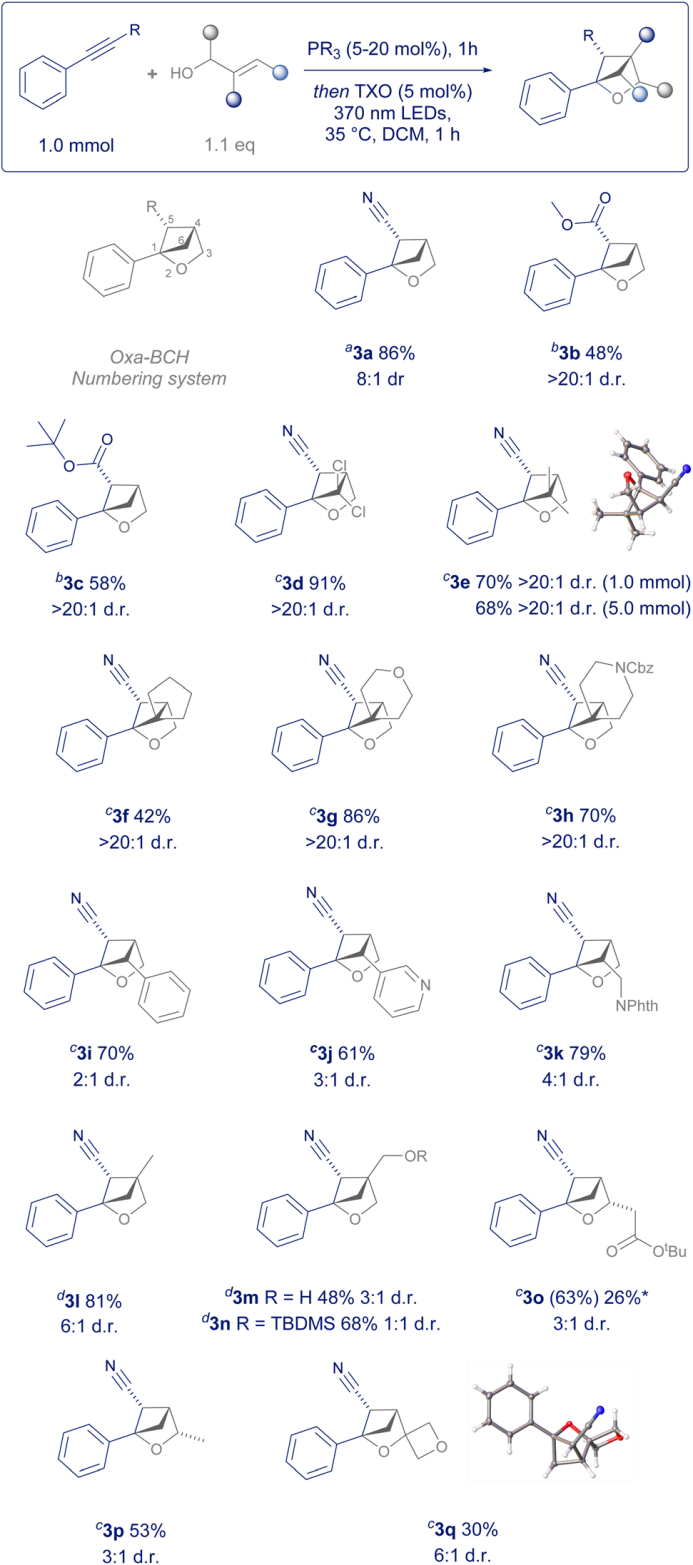
Scope of electron-withdrawing group and allyl alcohols. Major diastereomer depicted in all cases. Diastereomeric ratio (d.r.) determined from the crude ^1^H NMR spectrum and reported to the nearest whole number. *a* = 1 h PBu_3_ (5 mol%) catalysed conjugate addition, 1 h irradiation; *b* = 1 h PBu_3_ (15 mol%) catalysed conjugate addition, 1 h irradiation; *c* = 1 h PMe_3_ (20 mol%) catalysed conjugate addition, 1 h irradiation; *d* = 1 h PMe_3_ (20 mol%) catalysed conjugate addition, 16 h irradiation; *63% ^1^H NMR yield and 26% isolated yield after recrystallisation.

To explore the scope of this transformation, we firstly showed that commercially available methyl 3-phenylpropiolate was a competent reaction partner, giving product 3b as a single diastereomer without the need for previously employed recrystallisation (Scheme 2).^8^3c demonstrated that *tert*-butyl esters were also a competent functional group for the transformation, whereby the increased steric bulk diminished the side reactivity observed in less hindered esters.

We then systematically explored every position on the 2-oxabicyclo[2.1.1]hexane by varying the allyl alcohol coupling partner. Firstly, allyl alcohols substituted on the terminus of the alkene translated into 2-oxabicyclo[2.1.1]hexanes substituted on the 6- position, leading to a plethora of novel substitution patterns. Dichloro (3d) and dimethyl (3e) examples were given as single diastereomers in excellent yield with the configuration confirmed *via* single crystal X-ray diffraction.‡‡Deposition number 2382092 (3e) 2382093 (3q) contain(s) the supplementary crystallographic data for this paper. These data are provided free of charge by the Cambridge Crystallographic Data Centre.3e could also be scaled to 5.0 mmol in batch, when irradiated for an additional three hours. Likewise, allyl alcohols with a cyclic terminal substituent resulted in unusual spirocyclic products such as cyclopentyl 3f, tetrahydropyran 3g and benzylcarbamate protected piperidine 3h, again as single diastereomers.

Commercial cinnamoyl alcohols gave rise to arylated and hetereoarylated examples 3i and 3j, whilst an allyl alcohol bearing a protected amine could be prepared in one step and then converted to oxa-BCH 3k. Both the 4- and 3-positions could also be substituted using this method. 2-Methylprop-2-en-1-ol, 2-methylenepropane-1,3-diol and its commercially available monoprotected silyl ether could be transformed into examples 3l–3n. However, increasing steric bulk at this position does lead to an incremental loss of control of diastereoselectivity and required extended irradiation times.

Racemic allyl alcohols with ester or alkyl substitution on the methylene position translated to 3-substituted 2-oxa-BCH scaffolds 3o and 3p in moderate yield. Whilst the respective dimethyl substituted allyl alcohol (2-methylbut-3-en-2-ol) was too hindered to undergo the required conjugate addition, the less encumbered 1-vinylcyclobutan-1-ol was shown to be a competent reaction partner in the process, allowing for a 3-substituted spirocyclic 2-oxabicyclo[2.1.1]hexane (3q) to be synthesised in one step from commercial sources and the structure confirmed by single crystal X-ray diffraction.‡ Overall, the scope of readily available allyl alcohols demonstrates that this protocol enables the systematic exploration of every position on the 2-oxa-BCH scaffold, a crucial requirement for an extensive structure–activity relationship (SAR) study on any medicinal chemistry campaign.

**Scheme 3:**
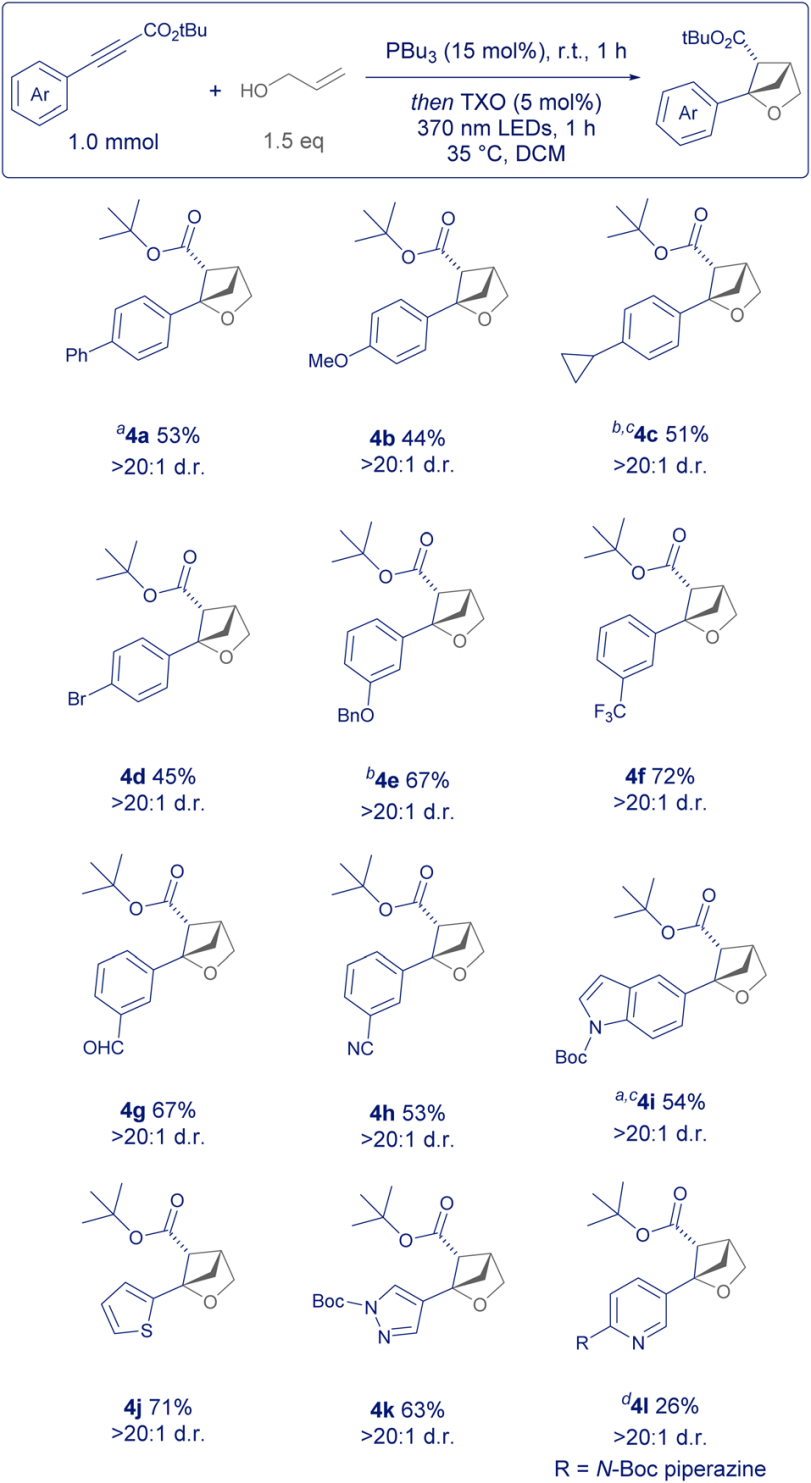
Scope of aryl group. *a* = 3 h PBu_3_ catalysed conjugate addition; *b* = 3 h irradiation; *c* = reaction carried out on 0.20 mmol scale; *d* = 3 h PMe_3_ (20 mol%) catalysed conjugate addition, 1 h irradiation.

Next, we turned our attention to the scope of the aromatic substituent of the arylalkyne (Scheme 3). Whilst simple arylpropiolates or arylpropiolonitriles are commercially available, there are also many ways of accessing them synthetically. Sonogashira disconnections from the parent aryl halide, copper catalysed couplings from the arylboronic acid or functionalisation of the terminal alkyne with chloroformates are the most popular.^15^

Our reaction design allows for the incorporation of aromatic structures that may have been incompatible with previously reported conditions which relied heavily on strong electrophiles such as molecular iodine or nucleophilic Grignard reagents (Scheme 1).^5,7–9^

Firstly, *para*-substituted phenyl rings were well tolerated such as examples 4a and 4b, as well as groups such as cyclopropyl (4c) and bromide (4d) without significant ring opening or dehalogenation of the product. Likewise, protected phenols such as 4e and highly electron-withdrawing groups in the *meta* position such as trifluoromethyl (4f) gave the 2-oxa-BCH product in good yield. Reactive groups such as aldehyde (4g) and nitrile (4h) were tolerated despite the former being previously employed as an electrophilic substrate in other areas of phosphine organocatalysis.^11^ A limitation of this process, however, is the use of *ortho*-substituted aromatics (such as 2-methyl), given their propensity to sterically hinder addition to the β-position of the alkyne starting material and diminish reactivity. Lastly, medicinally relevant hetereoaromatics such as indole (4i), thiophene (4j) and pyrazole (4k) gave product in good yield and excellent diastereoselectivity. Example 4l was a challenging substrate, potentially due to the nucleophilic pyridine nitrogen and electron rich piperazine substituent.

**Scheme 4:**
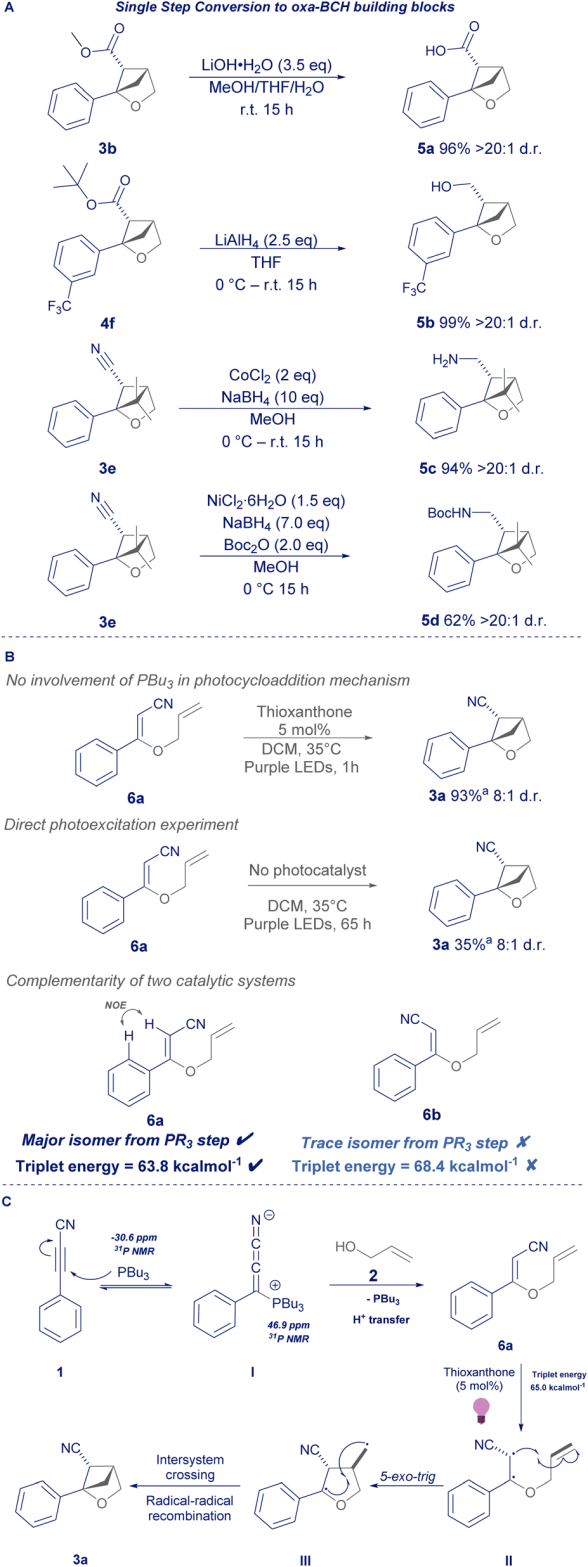
(A) = product post-functionalisation; (B) = mechanistic experiments; (C) = proposed mechanism. *a* = ^1^H NMR yield.

Having demonstrated that this process could use either esters or nitriles in the 5-position, we were keen to demonstrate that they can be converted to other useful functional handles in one step (Scheme 4A). Firstly, 3b could be hydrolysed under standard conditions to free acid 5a, and likewise a scope example from Scheme 3 was selected to demonstrate that the *tert*-butyl ester 4f could be reduced in quantitative yield to the respective primary alcohol (5b). Similarly, the nitrile in 3e could be reduced to free amine 5c or be intercepted by di-*tert*-butyl dicarbonate to give *N*-Boc-protected amine 5d.^16,17^

To probe the mechanism of the transformation, we initially followed the phosphine-mediated conjugate addition by ^31^P NMR spectroscopy, which suggested that the reaction proceeded *via* a likely phosphonium intermediate (see ESI†).^18^ Moreover, the lack of reactivity observed for species such as triethylamine and DABCO, implies that the phosphine is not acting as a Brønsted base in this transformation, but more likely a nucleophilic catalyst.^19,20^ Isolation of intermediate 6a and subsequent NMR analysis confirmed that the conjugate addition yields the proposed dienyl structure bearing a *Z*-configuration. 6a was subjected to the reaction conditions in the absence of phosphine (Scheme 4B), giving high yield of product 3a, confirming that the phosphine is not necessary for the photocycloaddition and that the trialkylphosphine can act as a spectator during the photocycloaddition.

Secondly, intermediate 6a was subjected to prolonged irradiation in the absence of photocatalyst, giving modest yield of 3a, suggesting that the mechanism doesn't require a redox event to undergo photocycloaddition. Lastly it was found that 6a is stable to the mild thermal conditions of the photochemistry and does not undergo significant amounts of photoisomerisation (see ESI†).

Whilst triplet energies are generally difficult to measure experimentally, gratifyingly, time-dependent density functional theory (TD-DFT) revealed that the *Z*-isomer of the diene intermediate possesses a triplet energy lower than the photocatalyst (6a*Z*-isomer 63.8 kcal mol^−1^; 6b*E*-isomer 68.4 kcal mol^−1^; thioxanthone 65.0 kcal mol^−1^), suggesting that the energy transfer pathway can proceed smoothly without the need for an initial photoisomerisation.^21^

Overall, we propose that the reaction proceeds *via* a phosphine catalysed conjugate addition of alkynyl starting material 1 and allyl alcohol 2, *via* intermediate I to give 6a (Scheme 4C). Irradiation at 370 nm facilitates the triplet energy transfer of the T_1_ excited state of thioxanthone to 6a, giving biradical intermediate II. A 5-*exo*- trig cyclisation (II to III), intersystem crossing and radical–radical recombination furnishes product 3a.

## Conclusions

In conclusion we have shown that a diverse array of readily accessible coupling partners can be used to construct 2-oxabicyclo[2.1.1]hexane scaffolds in a straightforward, economical procedure. The reaction can yield an array of substitution patterns and useful functional handles for further incorporation into medicinal scaffolds inaccessible through previous methods, without the need to isolate synthetic intermediates. Future work of our laboratory will focus on expanding this chemistry to new medicinally relevant scaffolds.

## Data availability

All the data related to the above-mentioned manuscript are available in the ESI.†

## Author contributions

D. M. W. was responsible for conceptualisation, investigation and methodology experiments. O. L. and N. A. A. were responsible for funding acquisition. O. L., N. A. A. and S. C. C. supervised the project. L. C. conducted the DFT calculations and O. D. P. acquired the X-ray crystallography data. The manuscript was written and revised by D. M. W., O. L., S. C. C. and N. A. A.

## Conflicts of interest

There are no conflicts to declare.

## Supplementary Material

SC-015-D4SC06684G-s001

SC-015-D4SC06684G-s002
