# Supplementary material for: Merging nucleophilic phosphine catalysis and photocatalysis for the rapid assembly of 2-oxabicyclo-[2.1.1]hexane scaffolds from feedstock allyl alcohols
Source: Chem Sci. 2024 Nov 4;15(46):19564–70. doi: 10.1039/d4sc06684g (PMC11575532; doi:10.1039/d4sc06684g)
Supplement: SC-015-D4SC06684G-s001 [file SC-015-D4SC06684G-s001.pdf]

Supplementary Information (SI) for Chemical Science.
This journal is © The Royal Society of Chemistry 2024

Merging Nucleophilic Phosphine Catalysis and Photocatalysis for the Rapid Assembly of 2-Oxabicyclo-[2.1.1]hexane Scaffolds from Feedstock Allyl Alcohols

Supporting Information

Table of Contents

General Remarks.. 3
Reaction Optimisation 4
General Procedures 6
Starting Material Synthesis 8
2-Oxabicyclo[2.1.1.]hexane Scope Entries.. 14
Aryl Scope Entries 24
Post-Functionalisations of Oxa-BCH Scaffolds 29
Attempted Addition of Allylamines and Longer Chain Alcohols 32
X-Ray Structure of 3e 33
X-Ray Structure of 3q 35
Mechanistic Studies 37

General Remarks

Reagents and anhydrous solvents were purchased from commercial suppliers unless otherwise stated and used without further purification.

NMR spectra were recorded on a Bruker 600 MHz or Bruker 500 MHz NMR machine with 3 mm NMR tubes. Measurements were taken at ambient temperature unless otherwise specified and were determined in $CDCl_3$, DCM-d_2, or MeCN-d_3. Splitting patterns are indicated as follows: s, singlet; d, doublet; t, triplet; q, quartet; m, multiplet. Coupling constants (J values) are reported in Hz. All chemical shifts are measured relative to residual solvent peaks and quoted in parts per million (ppm), measured from the centre of the signal except in the case of multiplets, which are quoted as a range.

Reactions were monitored using Ultra Pressure Liquid Chromatography Mass Spectrometry (LCMS) and thin layer chromatography on aluminium backed silica plates. Normal phase chromatography was performed on Teledyne Isco CombiFlash® Rf instruments using prepacked Puriflash high-capacity Silica columns (50 µm, spherical particles). High Resolution Mass Spectra (HRMS) were obtained using a Thermo Orbitrap IQ spectrometer in Electrospray (ESI) +ve or -ve ion mode.

Photochemical reactions were performed using two Kessil 'Gen 2' 370 nm LEDs with the vials placed 5 cm from the light sources and stirred from below with a magnetic stirrer plate. The reactions were cooled from above with an electric fan. The temperature of the reactions was measured with a thermometer to be 35 °C For 1.0 mmol scale reactions, 40 mL screw/septum glass vials were used, for 0.20 mmol scale, either 4 mL or 8 mL screw/septum glass vials were used. Tributylphosphine and trimethylphosphine were purchased from Merck (Sigma Aldrich) and used as received, 9H-thioxanthen-9-one was purchased from Fluorochem and used as received. 3-phenylpropiolonitrile was purchased from both Merck and Fluorochem and used as received.

Reaction Optimisation

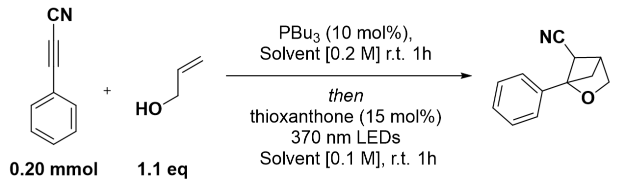

Entry	Solvent	Product ^{1}H NMR yield	d.r.
1	Toluene	52%	5:1
2	MeCN	98%	4:1
3	DMSO-d_6	49%	4:1
4	THF	46%	6:1
5	1,4-dioxane	54%	5:1
6	DCM	>99%	7:1
7	DCE	90%	6:1
8	Ethyl acetate	17%	4:1
9	Acetone	26%	6:1

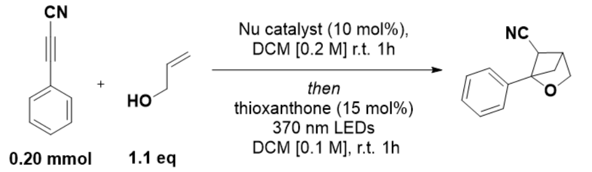

Entry	Nucleophilic catalyst	Product ^{1}H NMR yield	d.r.
1	PBu_3	>99%	7:1
2	PMe_3	96%	7:1
3	PPh_3	12%	5:1
4	DABCO	0%	
5	Et_3N	0%	

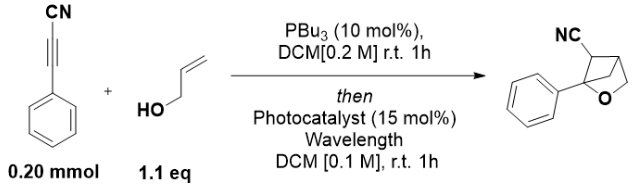

Entry	Solvent	Wavelength	Product ^{1}H NMR yield	d.r.
1	TXO	**370 nm (Kessil Gen 2)**	>99%	7:1
2	dOMe-TXO	**427 nm (Kessil)**	46%	7:1
3	Ir(dFCF$_3$ppy)$_2$(dtbbpy)PF$_6$ (2 mol%)	**456 nm (Kessil)**	40%	8:1
4	Benzophenone	**370 nm (Kessil Gen 2)**	43%	7:1
5	Mes-Acr	**456 nm (Kessil)**	n.d.	-

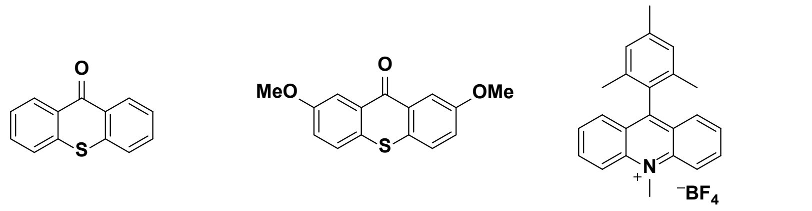

9*H*-thioxanthen-9-one (**TXO**)

9*H*-thioxanthen-9-one (**dOMe-TXO**)

9-mesityl-10-methylacridin-10-ium tetrafluoroborate (**Mes-Acr**)

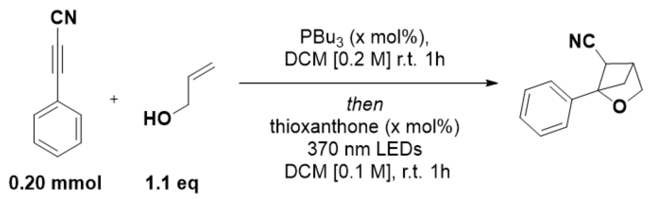

Entry	Loading of PBu_3	Loading of TXO	Product ^{1}H NMR	d.r.
**1**	**5%**	**5%**	**98%**	**7:1**
2	5%	10%	98%	7:1
3	10%	10%	>99%	7:1
4	10%	15%	>99%	7:1
5	15%	10%	>99%	6:1
6	15%	15%	98%	7:1

- ***Equivalents of allylic alcohol maintained at 1.1 eq***
- ***Effect of concentration shown to vary on scale (see below)***
- ***PBu_3 more convenient to use than PMe_3 (less volatile, safer)***

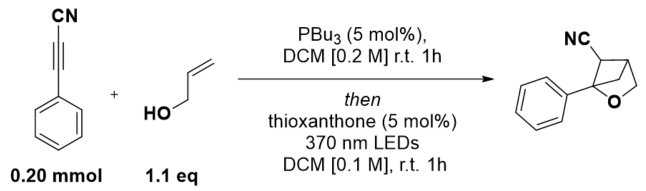

Entry	Change from above	Product ^{1}H NMR	d.r.
**1**	No PBu3	No product	n.d.
2	No Photocatalyst	<5%	n.d.
3	No Irradiation	No product	n.d.

Scaling to 1.0 mmol:

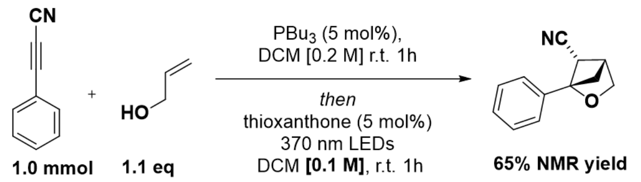

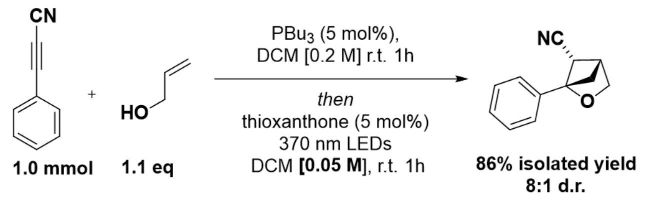

General Procedures

General Procedure A

A 40 mL vial charged with a stirrer bar and 3-phenylpropiolonitrile (127 mg, 1.0 mmol) underwent three vacuum/nitrogen cycles. Anhydrous DCM (5 mL, 0.2 M) was added, followed by the respective allylic alcohol derivative (1.10 mmol, 1.1 eq) under nitrogen atmosphere. **Tributylphosphine** (0.012 mL, 0.05, **5 mol%**) was added via microsyringe at room temperature under nitrogen atmosphere. The reaction was then stirred at room temperature for 1 hour. 9H-thioxanthen-9-one (10.61 mg, 0.05 mmol, 5 mol%) was added in DCM solution (15 mL) and the resulting reaction mixture was irradiated with two Kessil lamps (370 nm Gen 2) for **1 hour** at 35 °C. The solvent was then reduced under reduced pressure and the crude diastereomeric ratio was determined by ^{1}H NMR. The reaction mixture was then purified directly via flash column chromatography to give the product.

General Procedure B

A 40 mL vial charged with a stirrer bar and 3-phenylpropiolonitrile (127 mg, 1.0 mmol) underwent three vacuum/nitrogen cycles. Anhydrous DCM (5 mL, 0.2 M) was added, followed by the respective allylic alcohol derivative (1.10 mmol, 1.1 eq) under nitrogen atmosphere**. Tributylphosphine** (0.037 mL, 0.15 mmol**, 15 mol%)** was added via microsyringe at room temperature under nitrogen atmosphere. The reaction was then stirred at room temperature for 1 hour. 9H-thioxanthen-9-one (10.61 mg, 0.05 mmol, 5 mol%) was added in DCM solution (15 mL) and the resulting reaction mixture was irradiated with two Kessil lamps (370 nm Gen 2) for **1 hour** at 35 °C. The solvent was then reduced under reduced pressure and the crude diastereomeric ratio was determined by ^{1}H NMR. The reaction mixture was then purified directly via flash column chromatography to give the product.

General Procedure C

A 40 mL vial charged with a stirrer bar and 3-phenylpropiolonitrile (127 mg, 1.0 mmol) underwent three vacuum/nitrogen cycles. Anhydrous DCM (5 mL, 0.2 M) was added, followed by the respective allylic alcohol derivative (1.10 mmol, 1.1 eq) under nitrogen atmosphere. **Trimethylphosphine (1 M solution in toluene, 0.20 mL, 20 mol%)** was added via microsyringe at room temperature under nitrogen atmosphere. The reaction was then stirred at room temperature for **1 hour**. 9H-thioxanthen-9-one (10.61 mg, 0.05 mmol, 5 mol%) was added in DCM solution (15 mL) and the resulting reaction mixture was irradiated with two Kessil lamps (370 nm Gen 2) for **1 hour** at 35 °C. The solvent was then reduced under reduced pressure and the crude diastereomeric ratio was determined by ^{1}H NMR. The reaction mixture was then purified directly via flash column chromatography to give the product.

General Procedure D

A 40 mL vial charged with a stirrer bar and 3-phenylpropiolonitrile (127 mg, 1.0 mmol) underwent three vacuum/nitrogen cycles. Anhydrous DCM (5 mL, 0.2 M) was added, followed by the respective allylic alcohol derivative (1.10 mmol, 1.1 eq) under nitrogen atmosphere**. Trimethylphosphine** (1 M solution in toluene, 0.20 mL, 20 mol%) was added via microsyringe at room temperature under nitrogen atmosphere. The reaction was then stirred at room temperature for **1 hour**. 9H-thioxanthen-9-one (10.61 mg, 0.05 mmol, 5 mol%) was added in DCM solution (15 mL) and the resulting reaction mixture was irradiated with two Kessil lamps (370 nm Gen 2) for **16 hour** 35 °C. The solvent was then reduced under reduced pressure and the crude diastereomeric ratio was determined by ^{1}H NMR. The reaction mixture was then purified directly via flash column chromatography to give the product.

General Procedure E

A 40 mL vial charged with a stirrer bar and *tert*-butyl arylpropiolate (1.0 mmol, 1.0 eq) underwent three vacuum/nitrogen cycles. Anhydrous DCM (5 mL, 0.2 M) was added, followed by the respective **allylic alcohol derivative (1.10 mmol, 1.5 eq)** under nitrogen atmosphere**. Tributylphosphine** (0.037 mL, 0.15 mmol**, 15 mol%)**

was added via microsyringe at room temperature under nitrogen atmosphere. The reaction was then stirred at room temperature for **1 – 3 hours**. 9H-thioxanthen-9-one (10.61 mg, 0.05 mmol, 5 mol%) was added in DCM solution (15 mL) and the resulting reaction mixture was irradiated with two Kessil lamps (370 nm Gen 2) for **1 hour** at 35 °C. The solvent was then reduced under reduced pressure and the crude diastereomeric ratio was determined by ^{1}H NMR. The reaction mixture was then purified directly via flash column chromatography to give the product.

Sonogashira Procedure

Aryl iodide (5.0 mmol, 1 eq), CuI (38.1 mg, 4.0 mol%), $Pd(Cl)_2(PPh_3)_2$ (70.2 mg, 2.0 mol%), K_2CO_3 (1.38 g, 10.0 mmol 2.0 eq) and *tert*-butylpropiolate (946 mg, 7.5 mmol, 1.5 eq) was suspended in anhydrous THF (40 mL) and stirred at 65°C for 15 hours and allowed to cool to room temperature. The solids were removed via vacuum filtration and the solvent removed under reduced pressure. The crude reaction mixture was purified directly via flash column chromatography to yield the desired arylpropiolates.

Starting Material Synthesis

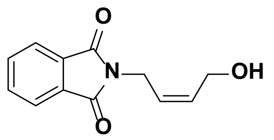

(Z)-2-(4-hydroxybut-2-en-1-yl)isoindoline-1,3-dione (S1)

DIAD (0.984 mL, 5.00 mmol) was added dropwise to phthalimide (0.608 mL, 5.00 mmol), (*Z*)-but-2-ene-1,4-diol (0.411 mL, 5.00 mmol) and triphenylphosphine (1.31 g, 5.00 mmol) in THF (50 mL) at room temperature under nitrogen. The resulting solution was stirred at room temperature overnight. The reaction mixture was concentrated under reduced pressure and the resulting crude product was purified directly by flash column chromatography (SiO_2, EtOAc in heptane 0 – 100% gradient, R_f = 0.19 at 50% EtOAc in heptane). The title compound was given as a colourless solid, (370 mg, 1.70 mmol, 34% yield).

^{1}H NMR (500 MHz, $CDCl_3$) 2.57 (t, *J* = 6.2 Hz, 1H), 4.38 (td, *J* = 6.8, 1.3 Hz, 2H), 4.41 (dd, *J* = 7.8, 1.3 Hz, 2H), 5.56 (dtt, *J* = 10.8, 7.7, 1.2 Hz, 1H), 5.90 (dtt, *J* = 10.8, 6.8, 1.2 Hz, 1H), 7.69 – 7.74 (m, 2H), 7.81 – 7.86 (m, 2H).

^{13}C NMR (126 MHz, $CDCl_3$) 168.3, 134.3, 133.4, 132.2, 125.1, 123.5, 58.2, 34.6.

Data was in accordance with literature: *Org. Lett.* **2011**, *13*, 10, 2686–2689

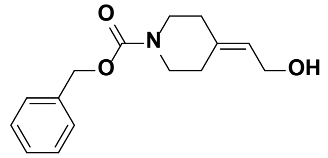

Benzyl 4-(2-hydroxyethylidene)piperidine-1-carboxylate (S2)

Methyl 2-(dimethoxyphosphoryl)acetate (0.809 mL, 5.00 mmol) was added to a suspension of NaH (0.200 g, 5.00 mmol) in THF (20 mL) at 0°C dropwise under nitrogen. The suspension was stirred for 1 hour, then benzyl 4-oxopiperidine-1-carboxylate (1166 mg, 5 mmol) was added under nitrogen and the cooling bath removed. The reaction mixture was allowed to warm to room temperature, then heated to 65°C for 1 hour. The reaction mixture was then allowed to stir at room temperature overnight, quenched with water and extracted three times with ethyl acetate. The organic extracts were combined and dried with $MgSO_4$, filtered and the solvent removed under reduced pressure, giving the crude Wittig intermediate as an oil. This was carried forward directly to the next step without further purification.

Crude benzyl 4-(2-methoxy-2-oxoethylidene)piperidine-1-carboxylate (868 mg, 3.0 mmol) was dissolved in THF (30 mL) and cooled to -78 °C under nitrogen. Diisobutylaluminum hydride solution (7.20 mL, 7.20 mmol, 1 M in toluene) was added slowly and the reaction was stirred for 45 minutes at -78 °C. The cooling bath was removed and the reaction mixture was allowed to stir at room temperature overnight. An additional diisobutylaluminum hydride was added at 0°C under nitrogen and stirred for 4 h. The reaction mixture was quenched with water at 0°C, diluted with brine and extracted three times with ethyl acetate. $MgSO_4$, the organic layer extracts combined and dried with $MgSO_4$, filtered and solvent removed under reduced pressure. The crude product was purified via flash column chromatography (SiO_2, EtOAc in heptane 0 – 100% gradient, R_f = 0.13 at 50% EtOAc in heptane), giving the title compound as a colourless oil (70 mg, 0.27 mmol, 8% yield).

^{1}H NMR (500 MHz, $CDCl_3$) 1.36 – 1.46 (m, 1H), 2.14 – 2.25 (m, 2H), 2.25 – 2.35 (m, 2H), 3.44 – 3.56 (m, 4H), 4.16 (dd, J = 6.9, 4.3 Hz, 2H), 5.14 (s, 2H), 5.50 (tt, J = 7.0, 1.3 Hz, 1H), 7.28 – 7.34 (m, 1H), 7.34 – 7.39 (m, 4H).

^{13}C NMR (126 MHz, CDCl3) 155.3, 139.0, 136.9, 128.6, 128.1, 128.0, 123.4, 67.3, 58.4, 45.6, 44.9, 35.8, 28.6

HRMS (ESI): calculated for $C_{15}H_{19}NO_3$ $[M+H]^+$: 262.14377, found 262.14365, error 0.458 ppm.

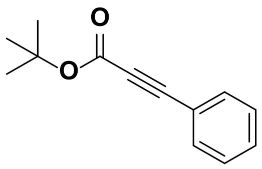

***tert*-Butyl 3-phenylpropiolate (S3)**

Iodobenzene (0.783 mL, 7.00 mmol) was added to *tert*-butyl propiolate (0.686 mL, 5 mmol), copper(I) iodide (6.78 µl, 0.20 mmol), potassium carbonate (0.854 mL, 15.00 mmol) and $PdCl_2(PPh_3)_2$ (70.2 mg, 0.10 mmol) in THF (40 mL) under nitrogen. The resulting suspension was stirred at 65 °C from overnight. The reaction mixture was filtered through Celite and washed with DCM (20 mL). The mixture was washed with brine and the aqueous layer extracted three times with DCM. The combined organic layers were dried with $MgSO_4$ and the solvent removed under reduced pressure. The crude product was purified via flash column chromatography (SiO_2, EtOAc in heptane 0 – 100% gradient, R_f = 0.36 at 20% EtOAc in heptane), giving the title compound as a yellow oil (519 mg, 2.57 mmol, 51% yield).

^{1}H NMR (500 MHz, $CDCl_3$) 1.55 (s, 9H), 7.33 – 7.38 (m, 2H), 7.4 – 7.44 (m, 1H), 7.54 – 7.59 (m, 2H).

^{13}C NMR (126 MHz, $CDCl_3$) 153.3, 133.0, 130.4, 128.6, 120.2, 83.9, 83.6, 82.2, 28.2.

Data was in accordance with literature: *Angew. Chem. Int. Ed*. **2023**, *62*, e202309169

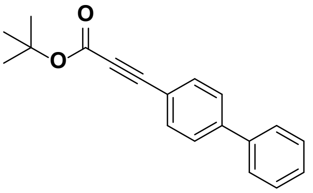

***tert*-Butyl 3-([1,1'-biphenyl]-4-yl)propiolate (S4)**

Synthesised according to the Sonogashira General Procedure, the crude product was purified via flash column chromatography (SiO_2, EtOAc in heptane 0 – 100% gradient, R_f = 0.32 at 20% EtOAc in heptane), giving the product as a light yellow solid (559 mg, 2.01 mmol, 40% yield).

^{1}H NMR (500 MHz, $CDCl_3$) 1.56 (s, 9H), 7.36 – 7.41 (m, 1H), 7.43 – 7.48 (m, 2H), 7.56 – 7.61 (m, 4H), 7.62 – 7.67 (m, 2H).

^{13}C NMR (126 MHz, $CDCl_3$) 153.3, 143.2, 140.0, 133.5, 129.1, 128.2, 127.3, 127.3, 118.9, 84.05, 83.7, 82.8, 28.2.

HRMS (ESI): Compound would not ionise

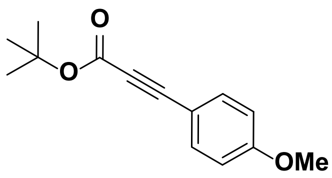

***tert*-Butyl 3-(4-methoxyphenyl)propiolate (S5)**

Synthesised according to the Sonogashira General Procedure, the crude product was purified via flash column chromatography (SiO_2, EtOAc in heptane 0 – 100% gradient, R_f = 0.23 at 20% EtOAc in heptane), giving the product as a yellow oil (635 mg, 2.73 mmol, 55% yield).

^{1}H NMR (500 MHz, $CDCl_3$) 1.54 (s, 9H), 3.82 (s, 3H), 6.86 (d, *J* = 8.9 Hz, 2H), 7.51 (d, *J* = 8.9 Hz, 2H).

^{13}C NMR (126 MHz, $CDCl_3$) 161.4, 153.6, 134.9, 114.3, 112.0, 84.7, 83.3, 81.6, 55.5, 28.2.

Data was in accordance with literature: *Chem. Commun.*, **2015**, *51*, 13004-13007

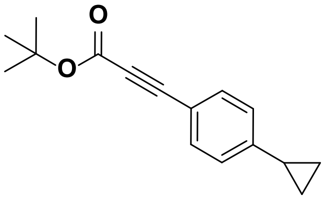

***tert*-Butyl 3-(4-cyclopropylphenyl)propiolate (S6)**

tert-Butyl propiolate (0.695 mL, 5.06 mmol) was added to (4-cyclopropylphenyl)boronic acid (410 mg, 2.53 mmol) and copper(I) oxide (36.2 mg, 0.25 mmol) in pyridine (1 mL) and DCM (10 mL). The resulting suspension was stirred at room temperature over 60 h open to air. The reaction mixture was filtered and the filtrate purified directly via flash column chromatography (SiO_2, EtOAc in heptane 0 – 40% gradient, R_f = 0.34 at 20% EtOAc in heptane), giving the title compound as a brown solid (613 mg, 0.73 mmol, 29% yield).

^{1}H NMR (500 MHz, $CDCl_3$) 0.68 – 0.78 (m, 2H), 0.97 – 1.08 (m, 2H), 1.54 (s, 9H), 1.89 (tt, *J* = 8.4, 5.0 Hz, 1H), 7.03 (d, *J* = 8.3 Hz, 2H), 7.45 (d, *J* = 8.5 Hz, 2H).

^{13}C NMR (126 MHz, $CDCl_3$) 153.5, 147.4, 133.1, 125.8, 116.7, 84.6, 83.5, 81.9, 28.2, 15.9, 10.2.

HRMS (ESI): Compound would not ionise

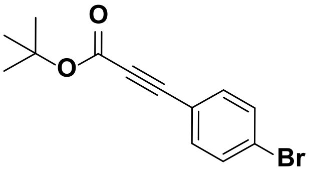

***tert*-Butyl 3-(4-bromophenyl)propiolate (S7)**

Synthesised according to the Sonogashira General Procedure, the crude product was purified via flash column chromatography (SiO_2, EtOAc in heptane 0 – 100% gradient, R_f = 0.38 at 20% EtOAc in heptane), giving the product as a brown oil (1.25 g, 4.44 mmol, 89% yield).

^{1}H NMR (500 MHz, $CDCl_3$) 1.54 (s, 9H), 7.4 – 7.44 (m, 2H), 7.49 – 7.52 (m, 2H).

^{13}C NMR (126 MHz, $CDCl_3$) 153.0, 134.3, 132.0, 125.2, 119.1, 83.9, 83.1, 82.7, 28.2.

Data was in accordance with literature: *Chem. Commun.,* **2016***,52,* 7482-7485

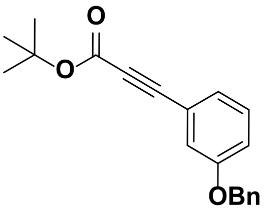

***tert*-Butyl 3-(3-(benzyloxy)phenyl)propiolate (S8)**

Synthesised according to the Sonogashira General Procedure, the crude product was purified via flash column chromatography (SiO_2, EtOAc in heptane 0 – 100% gradient, R_f = 0.30 at 20% EtOAc in heptane), giving the product as a pale yellow solid (950 mg, 0.308 mmol, 62% yield).

^{1}H NMR (500 MHz, $CDCl_3$) 1.55 (s, 9H), 5.06 (s, 2H), 7.05 (ddd, *J* = 8.3, 2.6, 1.1 Hz, 1H), 7.15 – 7.21 (m, 2H), 7.25 – 7.28 (m, 1H), 7.31 – 7.36 (m, 1H), 7.37 – 7.44 (m, 4H).

^{13}C NMR (126 MHz, $CDCl_3$) 158.7, 153.2, 136.6, 129.8, 128.8, 128.3, 127.6, 125.8, 121.1, 118.5, 118.2, 83.8, 83.7, 81.9, 70.3, 28.2.

HRMS (ESI): calculated for $C_{20}H_{20}O_3$ $[M+H]^+$: 309.14852, found 309.14859, error 0.223 ppm

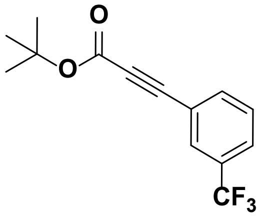

tert-Butyl 3-(3-(trifluoromethyl)phenyl)propiolate (S9)

Synthesised according to the Sonogashira General Procedure, (SiO_2, EtOAc in heptane 0 – 100% gradient, R_f = 0.44 at 20% EtOAc in heptane), giving the title compound as a yellow oil (1.20 g, 4.44 mmol, 89% yield).

^{1}H NMR (500 MHz, $CDCl_3$) 1.54 (s, 9H), 7.46 – 7.52 (m, 1H), 7.64 – 7.68 (m, 1H), 7.69 – 7.75 (m, 1H), 7.79 – 7.86 (m, 1H).

^{13}C NMR (126 MHz, $CDCl_3$) 28.2, 81.7, 83.2, 84.1, 121.2, 123.5 (*J* = 272.5), 126.9 (*J* = 3.7), 129.3, 129.7 (*J* = 3.9), 131.4 (*J* = 33.0), 135.9 (*J* = 1.4), 152.8.

^{19}F NMR (471 MHz, $CDCl_3$) -63.2.

HRMS (ESI): calculated for $C_{14}H_{13}F_3O_2$ [M-H]-: 269.07839, found 269.07968, error 0.715 ppm.

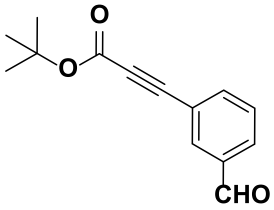

***tert*-Butyl 3-(3-formylphenyl)propiolate (S10)**

Synthesised according to the Sonogashira General Procedure, (SiO_2, EtOAc in heptane 0 – 100% gradient, R_f = 0.16 at 20% EtOAc in heptane), giving the product as an orange oil (953 mg, 4.14 mmol, 83% yield).

^{1}H NMR (500 MHz, $CDCl_3$) 1.55 (s, 9H), 7.52 – 7.58 (m, 1H), 7.78 – 7.83 (m, 1H), 7.9 – 7.97 (m, 1H), 8.04 – 8.1 (m, 1H), 10.00 (s, 1H).

^{13}C NMR (126 MHz, $CDCl_3$) 191.0, 152.8, 138.2, 136.7, 134.3, 130.8, 129.5, 121.5, 84.1, 83.2, 81.9, 28.2.

HRMS (ESI): calculated for $C_{14}H_{14}O_3$ $[M+H]^+$: 231.10157, found 231.10077, error 3.465 ppm.

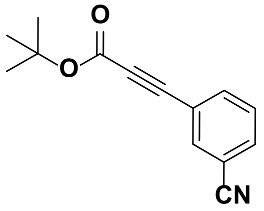

tert-Butyl 3-(3-cyanophenyl)propiolate (S11)

Synthesised according to the Sonogashira General Procedure, (SiO_2, EtOAc in heptane 0 – 100% gradient, R_f = 0.14 at 20% EtOAc in heptane), giving the product as an orange oil (852 mg, 3.75 mmol, 88% yield).

^{1}H NMR (500 MHz, $CDCl_3$) 1.53 (9H, s), 7.49 (td, *J* = 7.9, 0.7 Hz, 1H), 7.69 (dt, *J* = 7.8, 1.4 Hz, 1H), 7.76 (dt, *J* = 7.9, 1.4 Hz, 1H), 7.82 (td, *J* = 1.7, 0.6 Hz, 1H).

^{13}C NMR (126 MHz, $CDCl_3$) 152.5, 136.7, 136.0, 133.4, 129.7, 121.9, 117.6, 113.4, 84.3, 83.8, 80.5, 28.1.

HRMS (ESI): Compound would not ionise

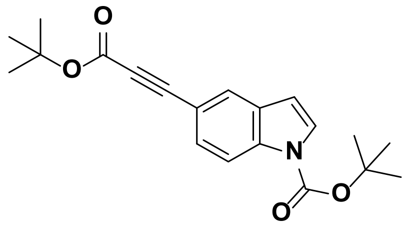

tert-Butyl 5-(3-(tert-butoxy)-3-oxoprop-1-yn-1-yl)-1H-indole-1-carboxylate (S12)

Synthesised according to the Sonogashira General Procedure, (SiO_2, EtOAc in heptane 0 – 100% gradient, R_f = 0.28 at 20% EtOAc in heptane), giving the product as a light yellow solid (80 mg, 0.23 mmol, 5% yield).

n.b. coupling goes to 87:13 product:starting material by LCMS peak area under Sonogashira General Procedure conditions. Similar Rf values of product and starting material hindered purification.

^{1}H NMR (500 MHz, $CDCl_3$) 1.55 (s, 9H), 1.67 (s, 9H), 6.56 (dd, *J* = 3.8, 0.8 Hz, 1H), 7.51 (dd, *J* = 8.6, 1.6 Hz, 1H), 7.63 (d, *J* = 3.7 Hz, 1H), 7.82 (dd, *J* = 1.6, 0.7 Hz, 1H), 8.13 (d, *J* = 8.6 Hz, 1H).

^{13}C NMR (126 MHz, $CDCl_3$) 153.5, 149.5, 136.1, 130.6, 128.9, 127.4, 126.5, 115.5, 114.0, 107.2, 85.3, 84.5, 83.4, 81.3, 28.3, 28.3.

HRMS (ESI): Compound would not ionise

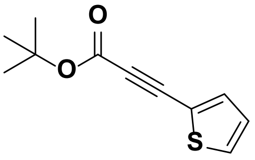

tert-Butyl 3-(thiophen-2-yl)propiolate (S13)

Synthesised according to the Sonogashira General Procedure, (SiO_2, EtOAc in heptane 0 – 100% gradient, R_f = 0.40 at 20% EtOAc in heptane), giving the product as an orange oil (777 mg, 3.73 mmol, 75% yield).

^{1}H NMR (500 MHz, $CDCl_3$) 1.54 (s, 9H), 7.03 (dd, J = 5.1, 3.7 Hz, 1H), 7.43 (dd, J = 5.1, 1.2 Hz, 1H), 7.46 (dd, J = 3.7, 1.2 Hz, 1H).

^{13}C NMR (126 MHz, $CDCl_3$) 153.1, 136.2, 130.8, 127.5, 120.0, 86.3, 83.8, 77.9, 28.2.

HRMS calculated for $C_{11}H_{12}O_2S$ $[M+H]^+$: 209.06308, found 209.06278, error 1.42 ppm

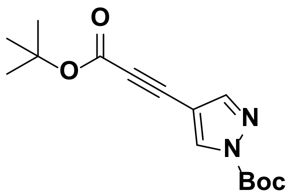

tert-Butyl 4-(3-(tert-butoxy)-3-oxoprop-1-yn-1-yl)-1H-pyrazole-1-carboxylate (S14)

Synthesised according to the Sonogashira General Procedure, (SiO_2, EtOAc in heptane 0 – 100% gradient, R_f = 0.09 at 20% EtOAc in heptane), giving the product as a yellow solid (1.03 g, 3.53 mmol, 71% yield).

^{1}H NMR (500 MHz, $CDCl_3$) 1.52 (s, 9H), 1.65 (s, 9H), 7.83 (d, J = 0.7 Hz, 1H), 8.32 (d, J = 0.7 Hz, 1H).

^{13}C NMR (126 MHz, $CDCl_3$) 152.7, 146.5, 146.0, 135.4, 103.7, 86.9, 85.0, 83.9, 74.4, 28.1, 27.9.

HRMS (ESI): calculated for $C_{15}H_{20}N_2O_4$ $[M+Na]^+$: 315.13153, found 315.13113, error 1.264 ppm

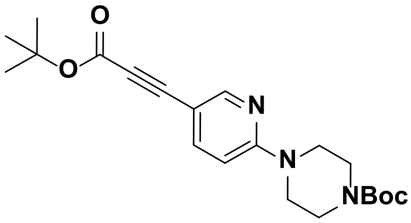

tert-Butyl 4-(5-(3-(tert-butoxy)-3-oxoprop-1-yn-1-yl)pyridin-2-yl)piperazine-1-carboxylate (S15)

Synthesised according to the Sonogashira General Procedure, (SiO_2, EtOAc in heptane 0 – 100% gradient, R_f = 0.50 at 50% EtOAc in heptane), giving the product as a brown solid (1.39 g, 3.60 mmol, 72% yield).

^{1}H NMR (500 MHz, $CDCl_3$) 1.48 (s, 9H), 1.54 (s, 9H), 3.45 – 3.59 (m, 4H), 3.59 – 3.68 (m, 4H), 6.56 (dd, *J* = 9.0, 0.8 Hz, 1H), 7.61 (dd, *J* = 8.9, 2.4 Hz, 1H), 8.38 (dd, *J* = 2.4, 0.7 Hz, 1H).

^{13}C NMR (126 MHz, $CDCl_3$) 158.6, 154.9, 153.5, 153.5, 141.5, 105.9, 105.1, 83.8, 83.4, 83.3, 80.3, 44.5, 28.6, 28.3.

HRMS (ESI): calculated for $C_{21}H_{29}N_3O_4$ $[M+H]^+$: 388.22308, found 388.22357, error 1.255 ppm

2-Oxabicyclo[2.1.1.]hexane Scope Entries

Some oxa-bicyclo[2.1.1]hexanes were synthesised as a mixture of diastereomers. The d.r. was recorded from the crude ^{1}H NMR spectrum and reported to the nearest whole number. In the case of **3k** and **3q**, the compounds were purified via reverse phase liquid chromatography and only the major diastereomer's data is reported. For compounds where there is a d.r. of less than 4:1, both the major and minor diastereomer's data is reported. Configurations were assigned using NOESY/ROESY NMR techniques as well as single crystal X-Ray diffraction.

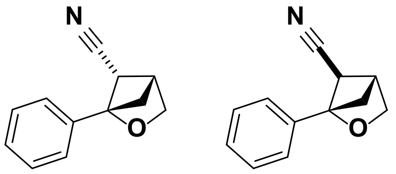

Crude ^{1}H NMR d.r. : 8 : 1

(±)-1-Phenyl-2-oxabicyclo[2.1.1]hexane-5-carbonitrile (3a)

Synthesised according to General Procedure A using prop-2-en-1-ol as the allylic alcohol. The crude ^{1}H NMR showed a d.r. of 8:1 and the purified product (SiO_2, EtOAc in heptane 0 – 100% gradient, R_f = 0.52 at 50% EtOAc in heptane) was given as a pale yellow oil (160 mg, 0.86 mmol, 86% yield).

^{1}H NMR (600 MHz, $CDCl_3$) 1.88 (d, *J* = 8.0 Hz, 1H), 2.23 (ddd, *J* = 8.0, 3.2, 1.0 Hz, 1H), 2.85 (d, *J* = 2.9 Hz, 1H), 3.32 (t, *J* = 3.1 Hz, 1H), 4.08 (d, *J* = 7.0 Hz, 1H), 4.28 (d, *J* = 7.0 Hz, 1H), 7.37 – 7.42 (m, 3H), 7.46 – 7.49 (m, 2H).

^{13}C NMR (151 MHz, CDCl3, 27°C) 134.8, 129.3, 128.7, 126.2, 116.1, 91.4, 68.4, 41.4, 41.2, 40.2.

HRMS (ESI): calculated for $C_{12}H_{11}ON$ $[M+H]^{+}$: 186.09134, found 186.09074, error 3.227 ppm.

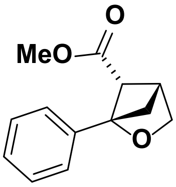

(±)-Methyl-1-phenyl-2-oxabicyclo[2.1.1]hexane-5-carboxylate (3b)

Synthesised according to General Procedure B using prop-2-en-1-ol (0.075 mL, 1.10 mmol) as the allylic alcohol. The crude ^{1}H NMR showed a single diastereomer (as well as an allyl ester adduct) and the purified product (SiO_2, EtOAc in heptane 0 – 100% gradient, R_f = 0.58 at 50% EtOAc in heptane) was given as a yellow solid, 105 mg, 0.48 mmol, 48% yield.

^{1}H NMR (500 MHz, $CDCl_3$) 1.74 (dt, *J* = 7.5, 1.0 Hz, 1H), 2.04 (ddd, *J* = 7.5, 3.2, 1.1 Hz, 1H), 2.95 (dd, *J* = 3.1, 1.1 Hz, 1H), 3.23 (td, *J* = 3.1, 0.8 Hz, 1H), 3.62 (s, 3H) 3.94 (d, *J* = 6.1 Hz, 1H), 4.28 (d, *J* = 6.1 Hz, 1H), 7.29 – 7.34 (m, 1H), 7.35 – 7.4 (m, 2H), 7.48 – 7.54 (m, 2H).

^{13}C NMR (126 MHz, $CDCl_3$) 169.4, 136.5, 128.3, 128.2, 126.6, 91.7, 68.4, 53.0, 51.5, 42.0, 39.8.

HRMS (ESI): calculated for $C_{13}H_{14}O_3$ $[M+H]^{+}$: 219.10157, found 219.10138, error 0.871 ppm.

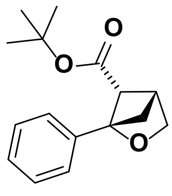

(±)-*tert*-Butyl-1-phenyl-2-oxabicyclo[2.1.1]hexane-5-carboxylate (3c)

Synthesised according to General Procedure B using **S3** and prop-2-en-1-ol (0.075 mL, 1.10 mmol) as the allylic alcohol. The crude ^{1}H NMR showed a single diastereomer and the purified product (SiO_2, EtOAc in heptane 0 – 100% gradient, R_f = 0.71 at 50% EtOAc in heptane) was given as a yellow oil (150 mg, 0.58 mmol, 58% yield).

^{1}H NMR (500 MHz, $CDCl_3$) 1.35 (s, 9H), 1.69 (dd, *J* = 7.5, 1.2 Hz, 1H), 2.01 (ddd, *J* = 7.5, 3.1, 1.1 Hz, 1H), 2.83 (dd, *J* = 3.1, 1.2 Hz, 1H), 3.16 – 3.18 (m, 1H), 3.91 (d, *J* = 6.0 Hz, 1H), 4.26 (d, *J* = 6.2 Hz, 1H), 7.27 – 7.32 (m, 1H), 7.33 – 7.38 (m, 2H), 7.49 – 7.53 (m, 2H).

^{13}C NMR (126 MHz, $CDCl_3$, 27°C) 168.4, 136.8, 128.0, 128.0, 126.9, 91.5, 80.8, 68.3, 54.2, 41.4, 39.8, 28.2.

HRMS (ESI): calculated for $C_{16}H_{20}O_3$ $[M+Na]^+$: 283.13047, found 283.12994, error 1.857 ppm.

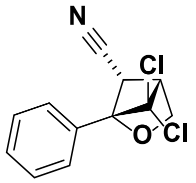

(±)-6,6-dichloro-1-phenyl-2-oxabicyclo[2.1.1]hexane-5-carbonitrile (3d)

Synthesised according to General Procedure C using 3,3-dichloroprop-2-en-1-ol as the allylic alcohol. The crude ^{1}H NMR showed the product as a single diastereomer. The purified product (SiO_2, EtOAc in heptane 0 – 100% gradient, R_f = 0.71 at 50% EtOAc in heptane) was given as a colourless solid, 232 mg, 0.91 mmol, 91% yield.

^{1}H NMR (500 MHz, $CDCl_3$) 3.64 (d, J = 3.0 Hz, 1H), 4.02 (d, J = 3.0 Hz, 1H), 4.36 (d, J = 8.0 Hz, 1H), 4.43 (d, J = 7.9 Hz, 1H), 7.43 – 7.53 (m, 3H), 7.54 – 7.59 (m, 2H).

^{13}C NMR (126 MHz, $CDCl_3$) 130.6, 128.8, 128.7, 127.2, 114.7, 96.0, 86.5, 66.8, 56.0, 35.4.

HRMS (ESI): calculated for $C_{12}H_9Cl_2NO$ $[M+H]^+$: 254.0134, found 254.01244, error 3.763 ppm.

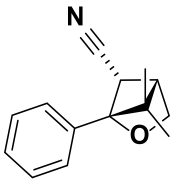

(±)-6,6-dimethyl-1-phenyl-2-oxabicyclo[2.1.1]hexane-5-carbonitrile (3e)

Synthesised according to General Procedure C using 3-methyl-2-buten-1-ol as the allylic alcohol. The crude ^{1}H NMR showed the product as a single diastereomer. The purified product (SiO_2, EtOAc in heptane 0 – 100% gradient, R_f = 0.61 at 50% EtOAc in heptane) was given as a colourless solid, 149 mg, 0.70 mmol, 70% yield.

Scaling to 5.0 mmol:

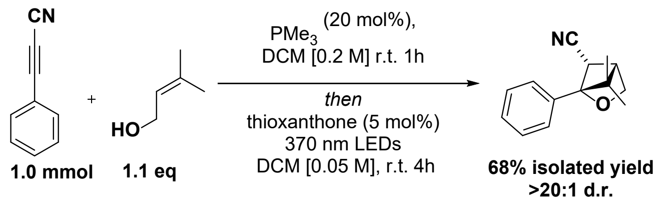

A 150 mL round-bottom flask was charged with a stirrer bar and 3-phenylpropiolonitrile (636 mg, 5.0 mmol) and underwent three vacuum/nitrogen cycles. Anhydrous DCM (25 mL) was added under nitrogen, followed by 3-methylbut-2-en-1-ol (0.559 mL, 5.50 mmol). Trimethylphosphane (1.00 mL, 1.00 mmol) was added via syringe at 0 °C under nitrogen atmosphere **(A)**. The reaction was then stirred at room temperature for 1 hour. 9H-thioxanthen-9-one (53 mg, 0.25 mmol, 5 mol%) was added in DCM solution (75 mL) **(B)** and the resulting reaction mixture was irradiated with two Kessil lamps (370 nm Gen 2) for 4 hours at 35 °C, cooled with a fan **(C)**. The solvent was then reduced under reduced pressure and the crude diastereomeric ratio was determined by ^{1}H NMR. The reaction mixture was then purified directly via flash column chromatography (SiO_2, EtOAc in heptane 0 – 100% gradient, R_f = 0.61 at 50% EtOAc in heptane) to give the product (725 mg, 3.40 mmol, 68%).

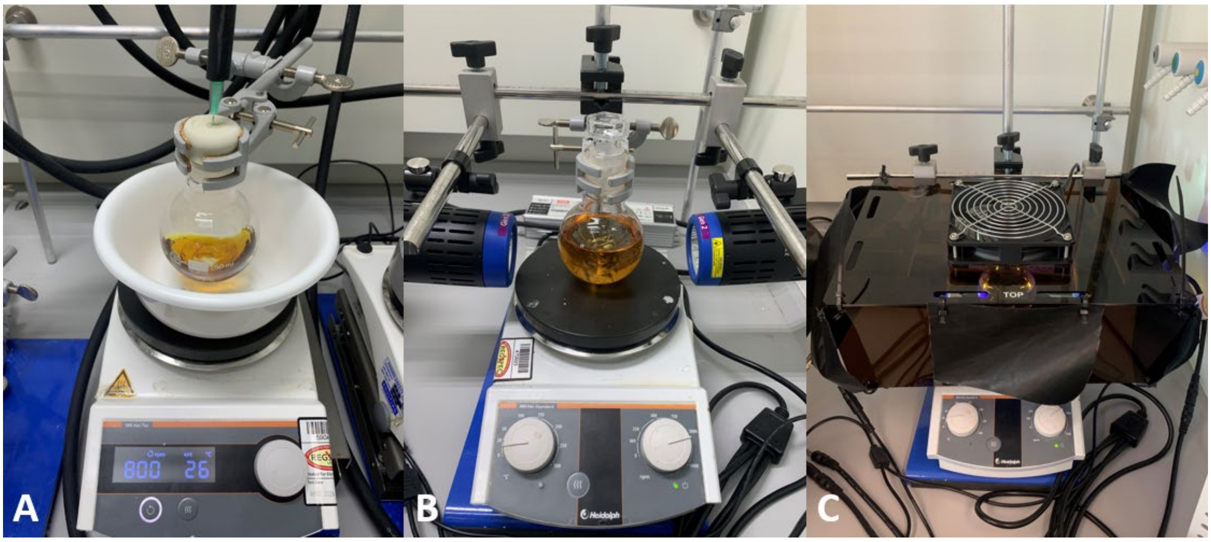

^{1}H NMR (600 MHz, $CDCl_3$, 27°C) 1.07 (s, 3H), 1.19 (s, 3H), 2.90 (d, *J* = 3.2 Hz, 1H), 3.57 (d, *J* = 3.1 Hz, 1H), 4.08 (d, *J* = 7.4 Hz, 1H), 4.19 (d, *J* = 7.4 Hz, 1H), 7.33 – 7.43 (m, 5H).

^{13}C NMR (151 MHz, $CDCl_3$, 27°C) 132.9, 129.0, 128.7, 126.5, 117.0, 93.6, 65.6, 50.5, 48.9, 35.2, 19.7, 17.2.

HRMS (ESI): calculated for $C_{14}H_{15}NO$ $[M+H]^+$: 214.12264, found 214.12199, error 3.039 ppm.

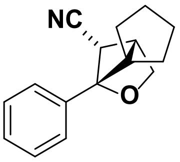

(±)-1-phenyl-2-oxaspiro[bicyclo[2.1.1]hexane-5,1'-cyclopentane]-6-carbonitrile (3f)

Synthesised according to General Procedure C using 2-cyclopentylideneethan-1-ol as the allylic alcohol. The crude ^{1}H NMR showed the product as a single diastereomer. The purified product (SiO_2, EtOAc in heptane 0 – 100% gradient, R_f = 0.65 at 50% EtOAc in heptane) was given as an orange oil (101 mg, 0.42 mmol, 42% yield).

^{1}H NMR (500 MHz, $CDCl_3$) 1.2 – 1.29 (m, 1H), 1.32 – 1.58 (m, 4H), 1.65 (dt, *J* = 13.0, 7.7 Hz, 1H), 1.71 – 1.8 (1H, m), 1.94 – 2.03 (m, 1H), 2.96 (d, *J* = 3.2 Hz, 1H), 3.38 (d, *J* = 3.1 Hz, 1H), 4.02 (d, *J* = 7.4 Hz, 1H), 4.19 (d, *J* = 7.4 Hz, 1H), 7.34 – 7.42 (m, 5H).

^{13}C NMR (126 MHz, $CDCl_3$) 133.0, 129.0, 128.7, 126.5, 116.4, 94.3, 66.3, 61.7, 49.7, 34.9, 29.7, 28.3, 26.6, 25.8.

HRMS : calculated for $C_{16}H_{17}ON$ $[M+H]^+$: 240.13829, found 240.13751, error 3.251 ppm.

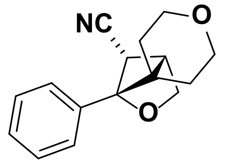

(±)-1-phenyltetrahydro-2-oxaspiro[bicyclo[2.1.1]hexane-5,4'-pyran]-6-carbonitrile (3g)

Synthesised according to General Procedure C using 2-(tetrahydro-4H-pyran-4-ylidene)ethan-1-ol as the allylic alcohol. The crude ^{1}H NMR showed the product as a single diastereomer. The purified product (SiO_2, EtOAc in heptane 0 – 100% gradient at R_f = 0.29 at 50% EtOAc in heptane) was given as a colourless oil (220 mg, 0.86 mmol, 86% yield).

^{1}H NMR (500 MHz, $CDCl_3$) 1.57 – 1.6 (m, 1H), 1.65 – 1.75 (m, 2H), 1.80 (ddt, J = 14.3, 4.8, 2.4 Hz, 1H), 3.17 (d, J = 3.2 Hz, 1H), 3.40 (ddd, J = 11.5, 10.0, 2.8 Hz, 1H), 3.49 (ddd, J = 11.6, 8.4, 4.4 Hz, 1H), 3.59 – 3.65 (m, 2H), 3.67 – 3.73 (m, 1H), 4.06 (d, J = 7.7 Hz, 1H), 4.26 (d, J = 7.7 Hz, 1H), 7.32 – 7.36 (m, 2H), 7.36 – 7.43 (m, 3H).

^{13}C NMR (126 MHz, $CDCl_3$) 132.4, 129.2, 128.8, 126.4, 116.6, 93.4, 65.3, 65.1, 65.0, 53.3, 45.7, 34.97, 29.6, 26.7.

HRMS calculated for $C_{16}H_{17}NO_2$ $[M+H]^+$: 256.13321, found 256.13287, error -1.33 ppm.

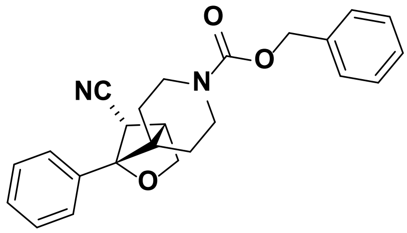

(±)-benzyl-6-cyano-1-phenyl-2-oxaspiro[bicyclo[2.1.1]hexane-5,4'-piperidine]-1'-carboxylate (3h)

Synthesised according to General Procedure C on a 0.20 mmol scale using **S2** as the allylic alcohol. The crude ^{1}H NMR showed the product as a single diastereomer. The purified product (SiO_2, EtOAc in heptane 0 – 100% gradient, R_f = 0.24 at 50% EtOAc in heptane) was given as a colourless solid, 52 mg, 0.134 mmol, 70% yield.

^{1}H NMR (500 MHz, $CDCl_3$) 1.45 – 1.54 (m, 1H), 1.57 – 1.67 (m, 1H), 1.75 – 1.83 (m, 1H), 1.83 – 1.9 (m, 1H), 2.91 – 3.05 (m, 1H), 3.05 – 3.13 (m, 1H), 3.15 (d, *J* = 3.2 Hz, 1H), 3.64 (d, *J* = 3.2 Hz, 1H), 3.76 (bs, 2H), 4.06 (d, *J* = 7.8 Hz, 1H), 4.27 (d, *J* = 7.8 Hz, 1H), 5.06 (s, 2H), 7.26 – 7.43 (m, 10H).

^{13}C NMR (126 MHz, $CDCl_3$) 155.2, 136.7, 132.2, 129.3, 128.9, 128.6, 128.2, 128.1, 126.4, 116.4, 93.4, 67.3, 65.1, 53.9, 45.4, 41.6, 41.1, 35.1, 28.5, 25.6.

HRMS (ESI): calculated for $C_{24}H_{24}N_2O_3$ $[M+H]^+$: 389.18597, found 389.18628, error 0.799 ppm.

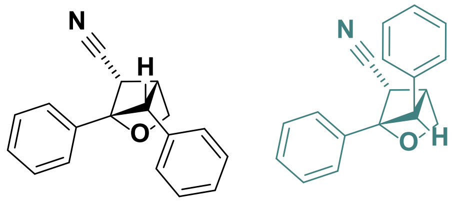

Crude ^{1}H NMR d.r. : 2 : 1

(±)-1,6-diphenyl-2-oxabicyclo[2.1.1]hexane-5-carbonitrile (3i)

Synthesised according to General Procedure C using (*E*)-3-phenylprop-2-en-1-ol as the allylic alcohol. The crude ^{1}H NMR showed the product in 2:1 d.r. and the purified product (SiO_2, EtOAc in heptane 0 – 100% gradient, R_f = 0.59 at 50% EtOAc in heptane) was given as a pale yellow solid (182 mg, 0.696 mmol, 70% yield).

Major isomer:

^{1}H NMR (500 MHz, $CDCl_3$) 2.84 (d, J = 2.9 Hz, 1H), 3.51 (t, J = 3.1 Hz, 1H), 3.6 – 3.61 (m, 1H), 3.78 (d, J = 7.4 Hz, 1H), 4.22 (d, J = 7.4 Hz, 1H), 7.15 – 7.19 (m, 2H), 7.22 – 7.25 (m, 3H), 7.41 – 7.44 (m, 3H), 7.57 – 7.61 (m, 2H).

^{13}C NMR (126 MHz, $CDCl_3$) 135.1, 134.3, 129.2, 128.9, 128.6, 127.8, 127.4, 126.6, 115.7, 92.7, 65.5, 51.5, 45.6, 40.2.

Minor isomer:

^{1}H NMR (500 MHz, $CDCl_3$) 3.54 – 3.58 (1H, m), 3.59 (d, J = 3.1 Hz, 1H), 3.67 (d, J = 2.9 Hz, 1H), 4.29 (d, J = 7.0 Hz, 1H), 4.41 (dd, J = 7.0, 0.8 Hz, 1H), 6.89 – 6.94 (m, 2H), 7.2 – 7.29 (5H, m), 7.33 – 7.4 (m, 3H).

^{13}C NMR (126 MHz, $CDCl_3$) 134.2, 133.2, 129.2, 128.7, 128.5, 128.3, 127.4, 126.9, 116.4, 93.3, 68.0, 59.7, 44.5, 35.9

HRMS calculated for $C_{18}H_{16}NO$ $[M+H]^+$: 262.12264, observed 262.12238, error -0.99 ppm.

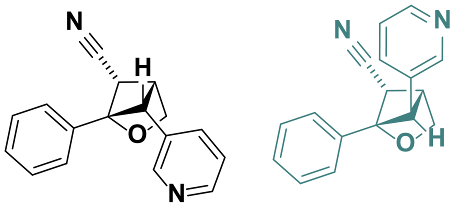

Crude ^{1}H NMR d.r. : 3 : 1

(±)-1-phenyl-6-(pyridin-3-yl)-2-oxabicyclo[2.1.1]hexane-5-carbonitrile (3j)

Synthesised according to General Procedure C using (*E*)-3-(pyridin-3-yl)prop-2-en-1-ol as the allylic alcohol. The crude ^{1}H NMR showed product in 2:1 d.r. and the purified product (SiO_2, EtOAc in heptane 0 – 100% gradient, R_f = 0.08 at 50% EtOAc in heptane) was given as a pale yellow solid (159 mg, 0.606 mmol, 61% yield).

Major isomer:

^{1}H NMR (500 MHz, $CDCl_3$) 2.91 (d, J = 2.8 Hz, 1H), 3.54 – 3.59 (m, 2H), 3.75 (d, J = 7.7 Hz, 1H), 4.26 (d, J = 7.7 Hz, 1H), 7.17 (ddd, J = 7.9, 4.9, 0.9 Hz, 1H), 7.34 – 7.47 (m, 4H), 7.52 – 7.56 (m, 2H), 8.41 – 8.46 (m, 1H), 8.46 – 8.5 (m, 1H).

^{13}C NMR (126 MHz, $CDCl_3$) 149.6, 148.9, 135.6, 133.7, 130.8, 129.5, 129.0, 126.5, 123.5, 115.3, 92.8, 65.4, 49.3, 45.6, 40.4.

Minor Isomer:

^{1}H NMR (500 MHz, $CDCl_3$) 3.50 (d, *J* = 3.0 Hz, 1H), 3.53 – 3.55 (m, 1H), 3.74 – 3.76 (m, 1H), 4.33 (d, *J* = 7.1 Hz, 1H), 4.44 (dd, *J* = 7.1, 0.8 Hz, 1H), 7.14 (dd, *J* = 3.3, 1.6 Hz, 2H), 7.21 – 7.26 (m, 2H), 7.35 – 7.4 (m, 2H), 7.52 (td, *J* = 2.0, 0.7 Hz, 2H), 8.26 (td, *J* = 1.7, 0.9 Hz, 1H).

^{13}C NMR (126 MHz, $CDCl_3$) 149.5, 148.9, 136.0, 132.6, 130.1, 129.7, 128.9, 126.8, 123.4, 115.9, 93.3, 68.2, 57.5, 44.1, 35.8

HRMS: calculated for $C_{17}H_{15}N_2O$ $[M+H]^+$: 263.11789, observed 263.11749, error -1.52 ppm

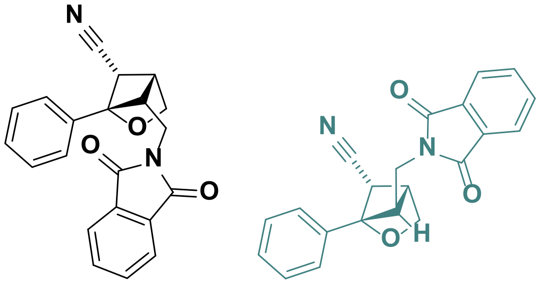

Crude ^{1}H NMR d.r. : 4 : 1

(±)-6-((1,3-dioxoisoindolin-2-yl)methyl)-1-phenyl-2-oxabicyclo[2.1.1]hexane-5-carbonitrile (3k)

Synthesised according to General Procedure C using **S1** as the allylic alcohol. The crude ^{1}H NMR showed the title compound as a 4:1 d.r and the title compound was given as a colourless solid, 274 mg, 0.794 mmol, 79% yield.

To demonstrate that a sample of only the major isomer could be obtained, the mixture underwent preparative achiral reverse phase separation (Column: Waters CSH C18 OBD, 5um silica, 30x100 mm; Mobile phase: A = 0.3% ammonium hydroxide, B = Acetonitrile; Gradient used: 30 - 60% B over 7 minutes; Flow rate: 50.0 mL/min) giving the major isomer as a colourless solid, 224 mg, 0.650 mmol, 65% yield.

^{1}H NMR (500 MHz, $CDCl_3$) 2.70 (d, *J* = 3.0 Hz, 1H), 2.73 (ddd, *J* = 8.7, 5.4, 2.9 Hz, 1H), 3.20 (t, *J* = 2.9 Hz, 1H), 3.69 – 3.79 (m, 2H), 4.34 (d, *J* = 7.9 Hz, 1H), 4.51 (d, *J* = 7.9 Hz, 1H), 7.3 – 7.34 (m, 1H), 7.37 (ddt, *J* = 8.0, 6.4, 1.2 Hz, 2H), 7.47 – 7.5 (m, 2H), 7.72 (dd, *J* = 5.5, 3.0 Hz, 2H), 7.81 (dd, *J* = 5.4, 3.1 Hz, 2H).

^{13}C NMR (126 MHz, $CDCl_3$) 168.1, 134.2, 133.0, 131.9, 129.2, 128.7, 126.2, 123.4, 115.1, 91.6, 65.2, 47.6, 43.4, 39.3, 32.3.

HRMS (ESI)**:** calculated for $C_{21}H_{17}N_2O_3$ $[M+H]^+$: 345.12337, observed 345.12317, error -0.58 ppm.

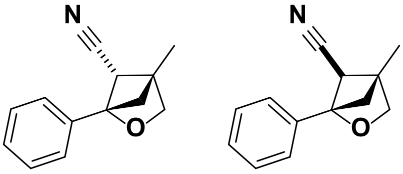

Crude ^{1}H NMR d.r. : 6 : 1

(±)-4-methyl-1-phenyl-2-oxabicyclo[2.1.1]hexane-5-carbonitrile (3l)

Synthesised according to General Procedure D using 2-methylprop-2-en-1-ol (0.093 mL, 1.10 mmol) as the allylic alcohol. The crude ^{1}H NMR showed a d.r. of 6:1 and the purified product (SiO_2, EtOAc in heptane 0 – 100% gradient, R_f = 0.61 at 50% EtOAc in heptane) was given as a yellow oil (162 mg, 0.81 mmol, 81% yield).

^{1}H NMR (500 MHz, $CDCl_3$, 27°C) 1.50 (s, 3H), 1.96 (dd, *J* = 7.9, 0.9 Hz, 1H), 2.03 (dd, *J* = 7.9, 1.2 Hz, 1H), 2.62 (s, 1H), 3.86 (d, *J* = 6.8 Hz, 1H), 4.06 (dd, *J* = 6.9, 1.2 Hz, 1H), 7.35 – 7.42 (m, 3H), 7.43 – 7.47 (m, 2H).

^{13}C NMR (126 MHz, $CDCl_3$, 27°C) 135.0, 129.2, 128.7, 126.2, 115.9, 89.7, 72.6, 50.4, 46.1, 44.9, 14.3.

HRMS (ESI): calculated for $C_{13}H_{14}ON$ $[M+H]^+$: 200.10699, found 200.1067, error 1.452 ppm.

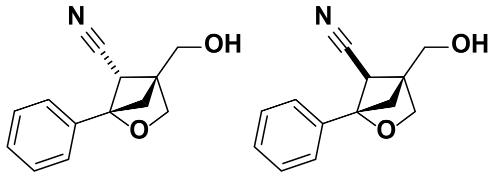

Crude ^{1}H NMR d.r. : 3 : 1

(±)-4-(hydroxymethyl)-1-phenyl-2-oxabicyclo[2.1.1]hexane-5-carbonitrile (3m)

Synthesised according to General Procedure D using 2-methylprop-2-en-1-ol (0.093 mL, 1.10 mmol) as the allylic alcohol. The crude ^{1}H NMR showed a d.r. of 3:1 and the purified product (SiO_2, EtOAc in heptane 0 – 100% gradient, R_f = 0.14 at 50% EtOAc in heptane) was given as a yellow solid (104 mg, 0.48 mmol, 48% yield).

Major

^{1}H NMR (500 MHz, $CDCl_3$) 1.94 (d, *J* = 7.9 Hz, 1H), 2.26 (d, *J* = 7.9 Hz, 1H), 2.90 (s, 1H), 3.96 (d, *J* = 6.8 Hz, 1H), 3.98 – 4.03 (m, 2H), 4.17 (d, *J* = 6.9 Hz, 1H), 7.38 – 7.43 (m, 3H), 7.45 – 7.5 (m, 2H).

^{13}C NMR (126 MHz, $CDCl_3$) 134.7, 129.3, 128.7, 126.2, 116.0, 89.6, 69.2, 59.3, 55.9, 42.3, 41.2.

Minor

1H NMR (500 MHz, $CDCl_3$) 2.12 (t, J = 8.5 Hz, 1H), 2.79 (d, J = 8.4 Hz, 1H), 3.04 (d, J = 8.4 Hz, 1H), 4.01 – 4.13 (m, 4H), 4.14 – 4.16 (m, 1H), 7.41 – 7.44 (m, 3H), 7.49 – 7.51 (m, 2H).

^{13}C NMR (126 MHz, $CDCl_3$) 134.1, 129.4, 128.7, 126.3, 118.6, 90.3, 71.0, 59.8, 54.7, 46.2, 42.4.

HRMS (ESI): calculated for $C_{13}H_{13}O_2N$ $[M+H]^+$: 216.10191, found 216.1012, error 3.263 ppm.

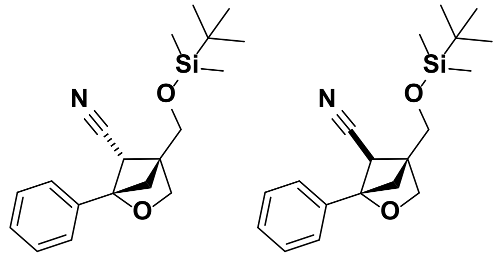

Crude ^{1}H NMR d.r. : 1 : 1

(±)-4-(((*tert*-Butyldimethylsilyl)oxy)methyl)-1-phenyl-2-oxabicyclo[2.1.1]hexane-5-carbonitrile (3n)

Synthesised according to General Procedure D using 2-(((tert-butyldimethylsilyl)oxy)methyl)prop-2-en-1-ol as the allylic alcohol. The crude ^{1}H NMR showed a d.r. of 1:1 and the purified product (SiO_2, EtOAc in heptane 0 – 100% gradient, R_f = 0.69 at 50% EtOAc in heptane) was given as a pale yellow solid (224 mg, 0.68 mmol, 68%).

Major

^{1}H NMR (500 MHz, $CDCl_3$) 0.10 (s, 6H), 0.92 (s, 9H), 1.91 (dd, J = 7.8, 0.9 Hz, 1H), 2.19 (dd, J = 7.8, 1.1 Hz, 1H), 2.81 (s, 1H), 3.9 – 3.97 (m, 3H), 4.15 (dd, J = 6.7, 1.1 Hz, 1H), 7.37 – 7.43 (m, 3H), 7.48 (ddd, J = 10.1, 8.0, 1.6 Hz, 2H).

^{13}C NMR (126 MHz, $CDCl_3$) 134.9, 129.3, 128.7, 126.2, 115.9, 89.4, 69.2, 59.9, 56.2, 42.3, 41.4, 26.0, 18.4, -5.4.

Minor

1H NMR (500 MHz, $CDCl_3$) 0.11 (d, J = 4.4 Hz, 6H), 0.92 (s, 9H), 2.07 (t, J = 8.4 Hz, 1H), 2.73 (dd, J = 8.4, 1.1 Hz, 1H), 3.00 (d, J = 8.4 Hz, 1H), 3.96 (d, J = 6.6 Hz, 1H), 4 – 4.04 (m, 2H), 4.11 (d, J = 11.4 Hz, 1H), 7.4 – 7.43 (m, 3H), 7.48 – 7.5 (m, 2H).

^{13}C NMR (126 MHz, $CDCl_3$) 134.4, 129.3, 128.7, 126.3, 118.4, 90.3, 71.2, 60.2, 54.8, 46.4, 42.4, 25.9, 18.4, -5.4.

HRMS calculated for $C_{19}H_{28}NO_2Si$ $[M+H]^+$: 330.18839, observed 330.18820, error -0.58 ppm.

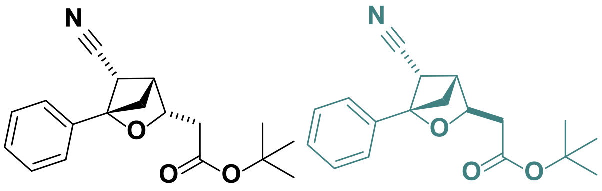

Crude ^{1}H NMR d.r. : 3 : 1

(±)-*tert*-Butyl 2-5-cyano-1-phenyl-2-oxabicyclo[2.1.1]hexan-3-yl)acetate (3o)

Synthesised according to General Procedure C using *tert*-butyl 3-hydroxypent-4-enoate as the allylic alcohol. The crude ^{1}H NMR showed the title compound as a 3:1 d.r. in 63% ^{1}H NMR yield. The crude reaction mixture was purified via flash column chromatography (SiO_2, EtOAc in heptane 0 – 100% gradient, R_f = 0.59 at 50% EtOAc in heptane), followed by a recrystallisation (ethyl acetate/heptane) and the title compound in 1:1 d.r. as a colourless solid (77 mg, 0.26 mmol, 26% yield).

Major Isomer:

^{1}H NMR (600 MHz, $CDCl_3$) 1.48 (s, 9H), 1.98 (dd, J = 8.0, 1.3 Hz, 1H), 2.21 (dd, J = 8.0, 3.1 Hz, 1H), 2.79 – 2.82 (m, 1H), 3.07 – 3.16 (m, 2H), 3.37 (t, J = 3.0 Hz, 1H), 4.63 (dd, J = 7.7, 6.3 Hz, 1H), 7.38 – 7.39 (m, 3H), 7.45 – 7.47 (m, 2H).

^{13}C NMR (126 MHz, $CDCl_3$) 170.3, 134.7, 129.3, 128.8, 126.2, 117.3, 91.8, 81.4, 76.7, 44.7, 37.6, 37.4, 37.2, 28.3.

Minor Isomer:

^{1}H NMR (600 MHz, $CDCl_3$) 1.48 (d, J = 0.8 Hz, 9H), 1.93 (d, J = 8.5 Hz, 1H), 2.13 (dd, J = 8.5, 3.0 Hz, 1H), 2.51 (dd, J = 15.8, 8.6 Hz, 1H), 2.78 – 2.88 (m, 1H), 2.93 (d, J = 3.0 Hz, 1H), 3.27 (t, J = 3.0 Hz, 1H), 4.85 (dd, J = 8.6, 5.6 Hz, 1H), 7.36 – 7.42 (m, 3H), 7.43 – 7.53 (m, 2H).

^{13}C NMR (126 MHz, $CDCl_3$) 169.4, 134.7, 129.4, 128.8, 126.2, 115.7, 91.1, 81.5, 74.5, 44.4, 44.2, 42.4, 39.2, 28.3.

HRMS (ESI): calculated for $C_{18}H_{21}NO_3$ [M+H]$^+$: 300.15942, found 300.15900, error -1.40 ppm.

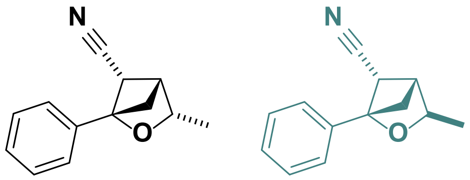

Crude ^{1}H NMR d.r. : 3 : 1

(±)-3-methyl-1-phenyl-2-oxabicyclo[2.1.1]hexane-5-carbonitrile (3p)

Synthesised according to General Procedure C using *rac*-but-3-en-2-ol as the allylic alcohol. The crude ^{1}H NMR showed the title compound as complex mixture and the title compound was given as a yellow oil in 3:1 d.r. after flash column chromatography (SiO_2, EtOAc in heptane 0 – 100% gradient, R_f = 0.53 at 50% EtOAc in heptane) (106 mg, 0.53 mmol, 53% yield).

^{1}H NMR (500 MHz, $CDCl_3$, 27°C) 1.70 (d, *J* = 6.7 Hz, 3H), 1.93 (dd, *J* = 7.9, 1.3 Hz, 1H), 2.18 (dd, *J* = 7.9, 3.1 Hz, 1H), 2.80 (dd, *J* = 2.8, 1.2 Hz, 1H), 3.15 (t, *J* = 3.0 Hz, 1H), 4.42 (q, *J* = 6.7 Hz, 1H), 7.37 – 7.42 (m, 3H), 7.47 – 7.5 (m, 2H).

^{13}C NMR (126 MHz, $CDCl_3$) 135.1, 129.2, 128.7, 126.1, 117.6, 91.7, 77.0, 45.6, 44.7, 37.1, 17.7.

Minor

^{1}H NMR (500 MHz, $CDCl_3$) 1.41 (d, J = 6.3 Hz, 3H), 1.98 (dt, J = 8.3, 0.7 Hz, 1H), 2.11 (dd, J = 8.3, 3.0 Hz, 1H), 2.90 (d, J = 2.9 Hz, 1H), 3.04 (td, J = 3.0, 0.7 Hz, 1H), 4.69 (q, J = 6.3 Hz, 1H), 7.34 – 7.43 (m, 3H), 7.44 – 7.51 (m, 2H).

^{13}C NMR (151 MHz, $CDCl_3$) 135.0, 129.2, 128.7, 126.1, 116.1, 90.9, 74.3, 45.0, 42.8, 37.2, 18.5.

HRMS (ESI): calculated for $C_{13}H_{13}ON$ [M+H]$^+$: 200.10699, found 200.10715, error 0.796 ppm.

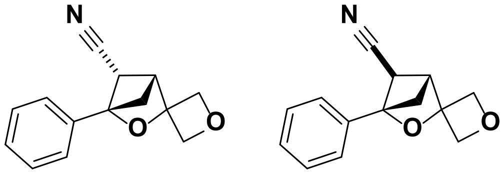

Crude ^{1}H NMR d.r. : 6 : 1

(±)-4-phenyl-3-oxaspiro[bicyclo[2.1.1]hexane-2,3'-oxetane]-5-carbonitrile (3q)

Synthesised according to General Procedure C using 1-vinylcyclobutan-1-ol as the allylic alcohol. The crude ^{1}H NMR showed the title compound as a. The major isomer was isolated by reverse phase chromatography (Column: Waters CSH C18 OBD, 5um silica, 30x100 mm; Mobile phase: A = 0.3% ammonium hydroxide, B = Acetonitrile; Gradient used: 25 - 50% B over 7 minutes; Flow rate: 50.0 mL/min) giving the title compound as a colourless solid (68.0 mg, 0.30 mmol, 30% yield).

^{1}H NMR (500 MHz, $CDCl_3$) 1.74 (dd, J = 8.4, 0.8 Hz, 1H), 2.25 (dd, J = 8.4, 3.0 Hz, 1H), 2.93 (d, J = 2.8 Hz, 1H), 3.60 (t, J = 2.9 Hz, 1H), 4.63 (d, J = 7.3 Hz, 1H), 4.92 – 5.08 (m, 2H), 5.12 – 5.27 (m, 1H), 7.39 – 7.44 (m, 3H), 7.44 – 7.48 (m, 2H).

^{13}C NMR (126 MHz, $CDCl_3$) 133.8, 129.7, 128.9, 126.1, 116.1, 91.3, 82.2, 81.5, 80.7, 46.7, 41.2, 41.2.

HRMS (ESI): calculated for $C_{14}H_{14}NO_2$ $[M+H]^+$: 228.10191, found 228.10156, error -1.53 ppm.

Aryl Scope Entries

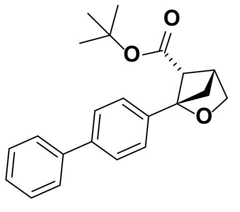

(±)-*tert*-Butyl-1-([1,1'-biphenyl]-4-yl)-2-oxabicyclo[2.1.1]hexane-5-carboxylate (4a)

Synthesised according to General Procedure E, 3 h conjugate addition and 1 h irradiation, using **S4** as the respective aryl propiolate and purified via flash column chromatography (SiO_2, EtOAc in heptane 0 – 100% gradient, R_f = 0.66 at 50% EtOAc in heptane). The title compound was given as a yellow solid (177 mg, 0.526 mmol, 53% yield).

^{1}H NMR (500 MHz, $CDCl_3$) 1.37 (s, 9H), 1.73 (dd, J = 7.5, 1.2 Hz, 1H), 1.98 – 2.15 (m, 1H), 2.87 (dd, J = 3.0, 1.1 Hz, 1H), 3.1 – 3.34 (m, 1H), 3.94 (d, J = 6.0 Hz, 1H), 4.27 (d, J = 6.0 Hz, 1H), 7.32 – 7.38 (m, 1H), 7.41 – 7.47 (m, 2H), 7.57 – 7.62 (m, 6H).

^{13}C NMR (126 MHz, $CDCl_3$) 168.4, 141.1, 140.9, 135.9, 128.9, 127.4, 127.3, 127.3, 126.8, 91.3, 80.9, 68.3, 54.3, 41.4, 39.8, 28.2.

HRMS (ESI): calculated for $C_{22}H_{24}O_3$ $[M+H]^+$: 337.17982, found 337.18045, error 1.865 ppm

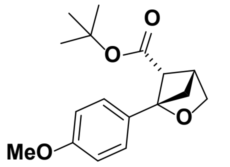

(±)-*tert*-Butyl-1-(4-methoxyphenyl)-2-oxabicyclo[2.1.1]hexane-5-carboxylate (4b)

Synthesised according to General Procedure E, 1 h conjugate addition and 1 h irradiation, with **S5** used as the respective aryl propiolate and purified via flash column chromatography (SiO_2, EtOAc in heptane 0 – 100% gradient, R_f = 0.61 at 50% EtOAc in heptane). The title compound was given as a yellow oil (128 mg, 0.441 mmol, 44% yield).

^{1}H NMR (500 MHz, $CDCl_3$) 1.37 (s, 9H), 1.67 (dd, *J* = 7.5, 1.2 Hz, 1H), 1.96 (ddd, *J* = 7.5, 3.1, 1.1 Hz, 1H), 2.77 (dd, *J* = 3.0, 1.2 Hz, 1H), 3.11 – 3.16 (m, 1H), 3.80 (s, 3H), 3.89 (d, *J* = 5.9 Hz, 1H), 4.24 (d, *J* = 5.7 Hz, 1H), 6.88 (d, *J* = 8.8 Hz, 2H), 7.44 (d, *J* = 8.8 Hz, 2H).

^{13}C NMR (126 MHz, $CDCl_3$) 168.5, 159.5, 129.2, 128.1, 113.4, 91.3, 80.8, 68.3, 55.4, 54.2, 41.3, 39.7, 28.2.

HRMS (ESI): calculated for $C_{17}H_{22}O_4$ $[M+H]^+$: 291.15909, found 291.15906, error 0.088 ppm

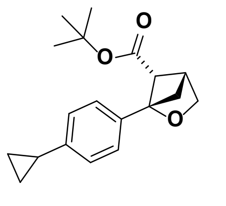

(±)-*tert*-Butyl-1-(4-cyclopropylphenyl)-2-oxabicyclo[2.1.1]hexane-5-carboxylate (4c)

Synthesised according to General Procedure E, 1 h conjugate addition and 3 h irradiation, on a 0.20 mmol scale using **S6** as the respective aryl propiolate and purified via flash column chromatography (SiO_2, EtOAc in heptane 0 – 100% gradient, R_f = 0.66 at 50% EtOAc in heptane). The title compound was given as a viscous oil (30.6 mg, 0.102 mmol, 51% yield).

^{1}H NMR (500 MHz, $CDCl_3$) 0.64 – 0.72 (m, 2H), 0.91 – 0.98 (m, 2H), 1.36 (s, 9H), 1.66 (dd, *J* = 7.5, 1.2 Hz, 1H), 1.89 (tt, *J* = 8.4, 5.1 Hz, 1H), 1.96 (ddd, *J* = 7.4, 3.2, 1.1 Hz, 1H), 2.79 (dd, *J* = 3.0, 1.1 Hz, 1H), 3.14 (tt, *J* = 3.2, 0.8 Hz, 1H), 3.89 (d, *J* = 6.0 Hz, 1H), 4.24 (d, *J* = 5.9 Hz, 1H), 7.05 (d, *J* = 8.1 Hz, 2H), 7.39 (d, *J* = 8.3 Hz, 2H).

^{13}C NMR (151 MHz, $CDCl_3$) 168.5, 143.9, 133.9, 126.8, 125.3, 91.5, 80.8, 68.3, 54.1, 41.3, 39.7, 28.2, 15.4, 9.3.

HRMS (ESI): calculated for $C_{19}H_{24}O_3$ $[M+H]^+$: 301.17982, found 301.17929, error 1.764 ppm.

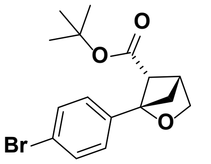

(±)-*tert*-Butyl-1-(4-bromophenyl)-2-oxabicyclo[2.1.1]hexane-5-carboxylate (4d)

Synthesised according to General Procedure E, 1 h conjugate addition and 1 h irradiation, with **S7** used as the respective aryl propiolate and purified via flash column chromatography (SiO_2, EtOAc in heptane 0 – 100% gradient, R_f = 0.66 at 50% EtOAc in heptane). The title compound was given as yellow solid (152 mg, 0.45 mmol, 45% yield).

^{1}H NMR (500 MHz, $CDCl_3$) 1.35 (s, 9H), 1.67 (dd, *J* = 7.5, 1.2 Hz, 1H), 1.97 (ddd, *J* = 7.5, 3.2, 1.0 Hz, 1H), 2.81 (dd, *J* = 3.2, 1.1 Hz, 1H), 3.17 (t, *J* = 3.1 Hz, 1H), 3.90 (d, *J* = 6.0 Hz, 1H), 4.23 (d, *J* = 6.0 Hz, 1H), 7.38 (d, *J* = 8.5 Hz, 2H), 7.48 (d, *J* = 8.6 Hz, 2H).

^{13}C NMR (126 MHz, $CDCl_3$) 168.2, 135.9, 131.2, 128.7, 122.0, 90.8, 81.1, 68.3, 54.2, 41.4, 39.8, 28.2.

HRMS (ESI): calculated for $C_{16}H_{19}BrO_3$ $[M+H]^+$: 339.05903, found 339.05753, error 4.435 ppm

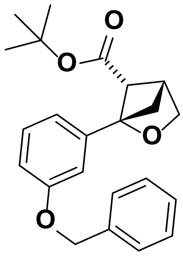

(±)-*tert*-Butyl-1-(3-(benzyloxy)phenyl)-2-oxabicyclo[2.1.1]hexane-5-carboxylate (4e)

Synthesised according to General Procedure E, 1 h conjugate addition and 3 h irradiation, with **S8** used as the respective aryl propiolate and purified via flash column chromatography (SiO_2, EtOAc in heptane 0 – 100% gradient, R_f = 0.35 at 50% EtOAc in heptane). The title compound was given as a pale yellow oil (247 mg, 0.67 mmol, 67% yield).

^{1}H NMR (500 MHz, $CDCl_3$) 1.36 (s, 9H), 1.69 (dd, *J* = 7.5, 1.2 Hz, 1H), 1.98 (ddd, *J* = 7.5, 3.2, 1.1 Hz, 1H), 2.83 (dd, *J* = 3.1, 1.1 Hz, 1H), 3.14 – 3.18 (m, 1H), 3.91 (d, *J* = 6.0 Hz, 1H), 4.26 (d, *J* = 6.0 Hz, 1H), 5.08 (s, 2H), 6.91 (ddd, *J* =

8.3, 2.6, 1.0 Hz, 1H), 7.11 (ddd, *J* = 7.6, 1.6, 1.0 Hz, 1H), 7.19 (dd, *J* = 2.6, 1.5 Hz, 1H), 7.25 – 7.29 (m, 1H), 7.3 – 7.34 (m, 1H), 7.36 – 7.4 (m, 2H), 7.42 – 7.46 (m, 2H).

^{13}C NMR (126 MHz, $CDCl_3$) 168.3, 158.7, 138.4, 137.2, 129.1, 128.7, 128.1, 127.7, 119.6, 114.4, 113.5, 91.4, 80.8, 70.1, 68.3, 54.2, 41.6, 39.7, 28.2.

HRMS (ESI): calculated for $C_{23}H_{26}O_4$ $[M+H]^+$: 367.19039, found 367.19055, error 0.447 ppm

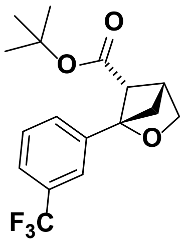

(±)-*tert*-Butyl-1-(3-(trifluoromethyl)phenyl)-2-oxabicyclo[2.1.1]hexane-5-carboxylate (4f)

Synthesised according to General Procedure E, 1 h conjugate addition and 1 h irradiation, with **S9** used as the respective aryl propiolate and purified via flash column chromatography (SiO_2, EtOAc in heptane 0 – 100% gradient, R_f = 0.63 at 50% EtOAc in heptane). The title compound was given as pale yellow oil (236 mg, 0.72 mmol, 72% yield).

^{1}H NMR (500 MHz, $CDCl_3$) 1.33 (s, 9H), 1.72 (dd, *J* = 7.5, 1.2 Hz, 1H), 2.03 (ddd, *J* = 7.5, 3.2, 1.0 Hz, 1H), 2.87 (dd, *J* = 3.1, 1.1 Hz, 1H), 3.21 (t, *J* = 3.1 Hz, 1H), 3.93 (d, *J* = 6.0 Hz, 1H), 4.27 (d, *J* = 6.0 Hz, 1H), 7.44 – 7.5 (m, 1H), 7.54 – 7.59 (m, 1H), 7.66 – 7.71 (m, 1H), 7.79 (tt, *J* = 1.7, 0.7 Hz, 1H).

^{13}C NMR (126 MHz, $CDCl_3$) 28.1, 39.9, 41.5, 54.3, 68.3, 81.2, 90.7, 123.9 (*J* = 3.8), 124.3 (J = 272.4), 124.8 (*J* = 3.8), 128.6, 130.3 (J = 1.4), 130.52 (J = 32.3), 137.9, 168.1.

^{19}F NMR (471 MHz, $CDCl_3$) -62.6.

HRMS (ESI): calculated for $C_{17}H_{19}F_3O_3$ $[M+H]^+$: 329.13591, found 329.13562, error 4.2 ppm.

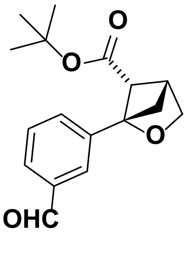

(±)-*tert*-Butyl-1-(3-formylphenyl)-2-oxabicyclo[2.1.1]hexane-5-carboxylate (4g)

Synthesised according to General Procedure E, 1 h conjugate addition and 1 h irradiation, with **S10** used as the respective aryl propiolate and purified via flash column chromatography (SiO_2, EtOAc in heptane 0 – 100% gradient, R_f = 0.55 at 50% EtOAc in heptane). The title compound was given as a yellow solid (193 mg, 0.67 mmol, 67% yield).

^{1}H NMR (500 MHz, $CDCl_3$) 1.33 (s, 9H), 1.72 (dd, *J* = 7.5, 1.2 Hz, 1H), 2.06 (ddd, *J* = 7.5, 3.2, 1.1 Hz, 1H), 2.89 (dd, *J* = 3.1, 1.1 Hz, 1H), 3.2 – 3.23 (m, 1H), 3.93 (d, *J* = 6.0 Hz, 1H), 4.25 (dd, *J* = 6.0, 1.1 Hz, 1H), 7.53 (td, *J* = 7.6, 0.5 Hz, 1H), 7.78 – 7.84 (m, 2H), 8.02 (td, *J* = 1.7, 0.6 Hz, 1H), 10.03 (s, 1H).

^{13}C NMR (126 MHz, $CDCl_3$) 192.4, 168.1, 138.1, 136.3, 133.0, 129.4, 128.8, 128.3, 90.7, 81.1, 68.3, 54.3, 41.4, 39.9, 28.1.

HRMS (ESI): calculated for $C_{17}H_{20}O_4$ $[M+H]^+$: 289.14344, found 289.14218, error 4.343 ppm.

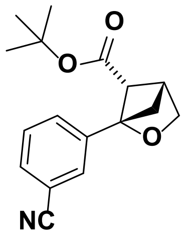

(±)-*tert*-Butyl-1-(3-cyanophenyl)-2-oxabicyclo[2.1.1]hexane-5-carboxylate (4h)

Synthesised according to General Procedure E, 1 h conjugate addition and 1 h irradiation, with **S11** used as the respective aryl propiolate and purified via flash column chromatography (SiO_2, EtOAc in heptane 0 – 100% gradient, R_f = 0.55 at 50% EtOAc in heptane). The title compound was given as a yellow oil (150 mg, 0.53 mmol, 53% yield).

^{1}H NMR (500 MHz, $CDCl_3$) 1.34 (s, 9H), 1.71 (dd, *J* = 7.6, 1.2 Hz, 1H), 2.01 (ddd, *J* = 7.5, 3.2, 1.0 Hz, 1H), 2.86 (dd, *J* = 3.2, 1.1 Hz, 1H), 3.22 (t, *J* = 3.2 Hz, 1H), 3.92 (d, *J* = 6.1 Hz, 1H), 4.22 (d, *J* = 6.1 Hz, 1H), 7.47 (t, *J* = 7.8 Hz, 1H), 7.59 (dt, *J* = 7.7, 1.4 Hz, 1H), 7.74 (dt, *J* = 7.9, 1.5 Hz, 1H), 7.84 (t, *J* = 1.8 Hz, 1H).

^{13}C NMR (126 MHz, $CDCl_3$) 167.9, 138.4, 131.6, 131.4, 130.9, 128.9, 118.9, 112.3, 90.1, 81.4, 68.3, 54.4, 41.3, 39.9, 28.2.

HRMS (ESI): calculated for $C_{17}H_{19}NO_3$ $[M+H]^+$: 286.14377, found 286.1437, error 0.245 ppm

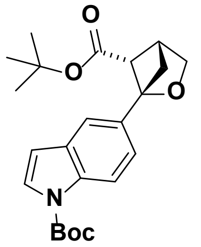

(±)-*tert*-Butyl-5-(tert-butoxycarbonyl)-2-oxabicyclo[2.1.1]hexan-1-yl)-1H-indole-1-carboxylate (4i)

Synthesised according to General Procedure E, 3 h conjugate addition and 1 h irradiation, on a 0.20 mmol scale using **S12** as the respective aryl propiolate and purified via flash column chromatography (SiO_2, EtOAc in heptane 0 – 100% gradient, R_f = 0.69 at 50% EtOAc in heptane). The title compound was given as a viscous yellow oil (43 mg, 0.108 mmol, 54% yield).

^{1}H NMR (500 MHz, $CDCl_3$) 1.36 (s, 9H), 1.67 (s, 9H), 1.74 (dd, *J* = 7.5, 1.2 Hz, 1H), 2.04 (ddd, *J* = 7.5, 3.2, 1.1 Hz, 1H), 2.86 (dd, *J* = 3.1, 1.1 Hz, 1H), 3.15 – 3.21 (m, 1H), 3.94 (d, *J* = 6.0 Hz, 1H), 4.29 (d, *J* = 5.9 Hz, 1H), 6.55 (dd, *J* = 3.7, 0.8 Hz, 1H), 7.46 (dd, *J* = 8.6, 1.7 Hz, 1H), 7.55 – 7.6 (m, 1H), 7.74 (dd, *J* = 1.7, 0.7 Hz, 1H), 8.11 (d, *J* = 8.6 Hz, 1H).

^{13}C NMR (126 MHz, $CDCl_3$) 168.4, 149.8, 134.9, 131.2, 130.2, 126.2, 123.1, 119.3, 114.5, 107.4, 91.7, 83.7, 80.7, 68.3, 54.2, 41.5, 39.6, 28.2, 28.1.

HRMS (ESI): calculated for $C_{23}H_{29}NO_5$ $[M+H]^+$: 400.21185, found 400.2121, error 0.626 ppm.

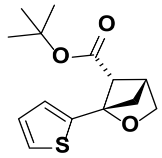

(±)-*tert*-Butyl (1S,4R,5R)-1-(thiophen-2-yl)-2-oxabicyclo[2.1.1]hexane-5-carboxylate (4j)

Synthesised according to General Procedure E, 1 h conjugate addition and 1 h irradiation, with **S13** used as the respective aryl propiolate and purified via flash column chromatography (SiO_2, EtOAc in heptane 0 – 100% gradient, R_f = 0.45 at 50% EtOAc in heptane). The title compound was given as a colourless oil (190 mg, 0.71 mmol, 71% yield).

^{1}H NMR (500 MHz, $CDCl_3$) 1.40 (s, 9H), 1.78 (dd, *J* = 7.5, 1.2 Hz, 1H), 2.00 (ddd, *J* = 7.5, 3.2, 1.1 Hz, 1H), 2.82 (dd, *J* = 3.1, 1.2 Hz, 1H), 3.15 (tt, *J* = 3.1, 0.7 Hz, 1H), 3.91 (d, *J* = 6.0 Hz, 1H), 4.23 (d, *J* = 6.0 Hz, 1H), 6.99 (dd, *J* = 5.0, 3.5 Hz, 1H), 7.13 (dd, *J* = 3.5, 1.2 Hz, 1H), 7.29 (dd, *J* = 5.0, 1.2 Hz, 1H).

^{13}C NMR (126 MHz, $CDCl_3$) 168.1, 139.3, 126.5, 125.7, 125.7, 87.8, 81.2, 68.6, 55.2, 42.6, 40.0, 28.2.

HRMS (ESI): calculated for $C_{14}H_{18}O_3S$ $[M+H]^+$: 267.10494, found 267.10413, error 3.039 ppm.

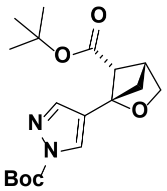

(±)-*tert*-Butyl-5-(tert-butoxycarbonyl)-2-oxabicyclo[2.1.1]hexan-1-yl)-1H-pyrazole-1-carboxylate (4k)

Synthesised according to General Procedure E, 1 h conjugate addition and 1 h irradiation, with **S14** used as the respective aryl propiolate and purified via flash column chromatography (SiO_2, EtOAc in heptane 0 – 100% gradient, R_f = 0.34 at 50% EtOAc in heptane). The title compound was given as a colourless oil (220 mg, 0.63 mmol, 63% yield).

^{1}H NMR (500 MHz, $CDCl_3$) 1.33 (s, 9H), 1.55 (s, 9H), 1.60 (dd, *J* = 7.5, 1.2 Hz, 1H), 1.79 (ddd, *J* = 7.5, 3.2, 1.1 Hz, 1H), 2.68 (dd, *J* = 3.1, 1.1 Hz, 1H), 3.07 (t, *J* = 3.1 Hz, 1H), 3.76 (d, *J* = 6.0 Hz, 1H), 4.07 (d, *J* = 6.1 Hz, 1H), 7.69 (d, *J* = 0.8 Hz, 1H), 8.08 (d, *J* = 0.7 Hz, 1H).

^{13}C NMR (151 MHz, $CDCl_3$) 167.9, 147.3, 142.7, 129.1, 120.8, 85.3, 84.2, 81.0, 67.8, 53.9, 41.1, 40.2, 28.0, 27.8.

HRMS (ESI): calculated for $C_{18}H_{26}N_2O_5$ $[M+H]^+$: 351.19145, found 351.19131, error 0.394 ppm

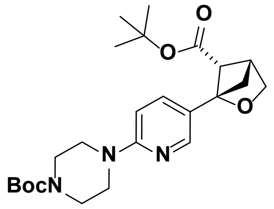

(±)-*tert*-Butyl-4-(tert-butoxycarbonyl)-2-oxabicyclo[2.1.1]hexan-1-yl)pyridin-2-yl)piperazine-1-carboxylate (4l)

Synthesised according to General Procedure E, 4 h conjugate addition with 20 mol% PMe_3 in place of PBu_3 and 1 h irradiation, with **S15** used as the respective aryl propiolate and purified via flash column chromatography (SiO_2,

EtOAc in heptane 0 – 100% gradient, R_f = 0.34 at 50% EtOAc in heptane). The title compound was given as viscous yellow oil (115 mg, 0.26 mmol, 26% yield).

^{1}H NMR (500 MHz, $CDCl_3$) 1.38 (s, 9H), 1.48 (s, 9H), 1.66 (dd, *J* = 7.5, 1.2 Hz, 1H), 1.95 (ddd, *J* = 7.5, 3.2, 1.0 Hz, 1H), 2.74 (dd, *J* = 3.1, 1.1 Hz, 1H), 3.07 – 3.21 (m, 1H), 3.53 (8H, s), 3.87 (d, *J* = 6.0 Hz, 1H), 4.19 (d, *J* = 6.0 Hz, 1H), 6.62 (dd, *J* = 8.8, 0.8 Hz, 1H), 7.70 (dd, *J* = 8.8, 2.4 Hz, 1H), 8.26 (dd, *J* = 2.4, 0.8 Hz, 1H).

^{13}C NMR (126 MHz, $CDCl_3$) 168.4, 159.2, 155.0, 146.6, 136.6, 122.2, 106.4, 89.7, 81.0, 80.1, 68.2, 54.2, 45.4, 40.8, 39.9, 28.6, 28.3.

n.b. The piperazine moiety exhibits a single resonance in $CDCl_3$ at 300K (3.53 (8H, s)) which is coincidental as the proton signals are not theoretically equivalent. However, 2 distinct signals for both the ^{13}C (45.2 / 43.5 ppm) and ^{15}N (77.3 / 84.4 ppm) resonances are observed for the piperazine moiety.

HRMS (ESI): calculated for $C_{24}H_{35}N_3O_5$ $[M+H]^+$: 446.26495, found 446.26428, error 1.496 ppm

Post-Functionalisations of Oxa-BCH Scaffolds

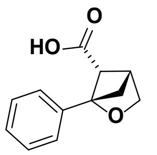

(±)-1-phenyl-2-oxabicyclo[2.1.1]hexane-5-carboxylic acid (5a)

Lithium hydroxide hydrate (0.029 g, 0.70 mmol) was added to a stirred solution of **3b** (0.044 g, 0.2 mmol) in THF (0.75 mL), methanol (0.38 mL) and water (0.19 mL) at room temperature for 15 hours. The reaction mixture was diluted with methanol the solvent reduced under reduced pressure. The crude reaction mixture was dissolved in NH_4Cl and extracted six times with ethyl acetate, the organic extracts combined and dried with $MgSO_4$, filtered and the solvent removed under reduced pressure, giving the product as a colourless viscous oil that solidified upon standing (36 mg, 0.191 mmol, 96% yield)

^{1}H NMR (500 MHz, CD_3CN) 1.62 (dd, J = 7.5, 1.2 Hz, 1H), 2.07 (ddd, J = 7.5, 3.2, 1.1 Hz, 1H), 3.05 (dd, J = 3.0, 1.2 Hz, 1H), 3.1 – 3.19 (m, 1H), 3.86 (d, J = 6.0 Hz, 1H), 4.15 (d, J = 5.9 Hz, 1H), 7.29 – 7.35 (m, 1H), 7.35 – 7.4 (m, 2H), 7.47 – 7.55 (m, 2H).

^{13}C NMR (126 MHz, CD_3CN) 170.5, 138.1, 129.0, 128.8, 127.5, 92.2, 68.6, 53.4, 42.5, 40.5.

HRMS (ESI): calculated for $C_{12}H_{12}O_3$ $[M-H]^-$: 203.07027, found 203.07167, error 1.489 ppm

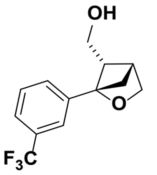

(±)-1-(3-(trifluoromethyl)phenyl)-2-oxabicyclo[2.1.1]hexan-5-yl)methanol (5b)

A solution of $LiAlH_4$ (0.520 mL, 0.52 mmol, 1M in THF) was added dropwise to a stirred solution of **4f** (65.7 mg, 0.2 mmol) in THF (2 mL) at 0 °C, over a period of 2 minutes under nitrogen atmosphere. The resulting solution was stirred at room temperature for 15 hours. Reaction mixture was quenched with saturated NH_4Cl solution slowly at 0 °C and the reaction mixture was extracted three times with DCM, the organic extracts combined, dried with

MgSO4, filtered and the solvent removed under reduced pressure, giving the product as a pale yellow oil (51 mg, 0.197 mmol, 99% yield).

^{1}H NMR (500 MHz, $CDCl_3$) 1.75 (dd, *J* = 7.5, 1.0 Hz, 1H), 2.01 (ddd, *J* = 7.6, 3.3, 1.0 Hz, 1H), 2.43 (tdd, *J* = 6.6, 3.1, 1.0 Hz, 1H), 3.00 (t, *J* = 3.2 Hz, 1H), 3.60 (dd, *J* = 11.3, 6.5 Hz, 1H), 3.67 (dd, *J* = 11.3, 6.6 Hz, 1H), 3.89 (d, *J* = 6.5 Hz, 1H), 3.92 (d, *J* = 0.7 Hz, 1H), 7.44 – 7.49 (m, 1H), 7.53 – 7.56 (m, 1H), 7.59 – 7.65 (m, 1H), 7.67 – 7.78 (m, 1H).

^{19}F NMR (471 MHz, $CDCl_3$) -62.6.

^{13}C NMR (151 MHz, $CDCl_3$) 38.9, 42.4, 52.9, 58.2, 67.1, 89.5, 123.4 (q, *J* = 3.8 Hz), 124.1 (q, *J* = 272.3), 124.8 (q, *J* = 3.8 Hz), 128.9, 129.9 (d, *J* = 1.4 Hz), 130.8 (q, *J* = 32.2 Hz), 138.5.

HRMS (ESI): calculated for $C_{13}H_{13}F_3O_2$ $[M-H]^-$: 257.07839, found 257.07935, error 0.535 ppm.

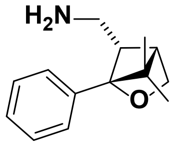

(±)-6,6-dimethyl-1-phenyl-2-oxabicyclo[2.1.1]hexan-5-yl)methanamine (5c)

Based on a procedure adapted from (A. Karmakar, S. Ramalingam, M. Basha, G. K. Indasi, M. Belema, N. A. Meanwell, T. G. Dhar, R. Rampulla, A. Mathur, A. Gupta, A.K Gupta, *Synthesis,* **2019**, *52*, 441–449.)

A 25 mL flask was charged with **3e** (42.7 mg, 0.20 mmol) and cobalt(II) chloride (51.9 mg, 0.40 mmol) and underwent three vacuum/nitrogen cycles, cooled to 0 °C, then addition of anhydrous MeOH (2 mL) and a slow addition of $NaBH_4$ (76 mg, 2.0 mmol). The reaction was stirred for 15 hours at room temperature.

The reaction was then diluted with 5 mL methanol added slowly to a stirred aqueous HCl solution (2M) at room temperature and stirred for 5 mins. This was then basified with aqueous NaOH solution (2 M) to (pH 12) and extracted three times with ethyl acetate and dried with $MgSO_4$ and filtered. The solvent removed under reduced pressure giving the title compound as a colourless oil (41 mg, 0.189 mmol, 94% yield).

^{1}H NMR (500 MHz, CD_3CN) 0.88 (s, 3H), 1.13 (s, 3H), 2.49 (d, *J* = 3.2 Hz, 1H), 2.56 (dd, *J* = 12.9, 7.6 Hz, 1H), 2.62 (dd, *J* = 12.9, 6.0 Hz, 1H), 2.96 (ddd, *J* = 7.6, 6.0, 3.2 Hz, 1H), 3.67 (d, *J* = 6.7 Hz, 1H), 3.83 (d, *J* = 6.7 Hz, 1H), 7.26 – 7.31 (m, 1H), 7.31 – 7.4 (m, 4H).

^{13}C NMR (126 MHz, CD_3CN) 136.8, 129.0, 128.4, 127.7, 92.4, 64.2, 49.3, 48.4, 46.9, 37.4, 20.0, 17.0.

HRMS (ESI): calculated for $C_{14}H_{19}NO$ $[M+H]^+$: 218.15394, found 218.15369, error 1.15 ppm

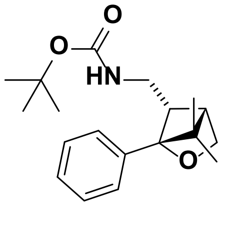

(±)-*tert*-Butyl (((1R,4S,5S)-6,6-dimethyl-1-phenyl-2-oxabicyclo[2.1.1]hexan-5-yl)methyl)carbamate (5d)

Based on a procedure adapted from (S. Caddick, D. B. Judd, A. K. de K. Lewis, M. T. Reich, M. R. V. Williams, *Tetrahedron*, **2003**, *59*, 5417-542)

$NaBH_4$ (132 mg, 3.50 mmol) was added portionwise to nickel(II) chloride hexahydrate (178 mg, 0.75 mmol), **3e** (107 mg, 0.5 mmol) and di-*tert*-butyl dicarbonate (0.230 mL, 1.00 mmol) in MeOH (5 mL) at 0 °C over a period of 2 minutes under air. The resulting solution was stirred at room temperature under nitrogen for 15 hours. The reaction mixture was diluted with DCM (25 mL), washed with saturated NH_4Cl (100 mL). The organic layer was dried with MgSO4, filtered and evaporated to afford crude product. The crude product was purified via flash column chromatography (SiO_2, EtOAc in heptane, 0 – 100% gradient, R_f = 0.63 at 50% EtOAc in heptane) giving the product as a colourless oil (98 mg, 0.31 mmol, 62%).

^{1}H NMR (500 MHz, $CDCl_3$) 0.92 (s, 3H), 1.14 (s, 3H), 1.43 (s, 9H), 2.49 (d, *J* = 2.6 Hz, 1H), 3.03 – 3.13 (m, 2H), 3.16 – 3.26 (m, 1H), 3.76 (d, *J* = 7.0 Hz, 1H), 3.91 (d, *J* = 7.0 Hz, 1H), 4.43 (s, 1H), 7.26 – 7.3 (m, 1H), 7.3 – 7.37 (m, 4H).

^{13}C NMR (126 MHz, $CDCl_3$) 156.2, 135.1, 128.3, 127.7, 126.7, 91.9, 79.3, 63.9, 49.1, 46.5, 44.5, 36.2, 28.6, 19.7, 16.7.

HRMS (ESI): calculated for $C_{19}H_{27}NO_3$ $[M+H]^+$: 318.20637, found 318.20538, error 3.112 ppm.

Attempted Addition of Allylamines and Longer Chain Alcohols

The following allylamine derivatives were also tested in the reaction (General Procedure D) but yielded no product, likely owing to steric hinderance of the conjugate addition step (monitored by LCMS).

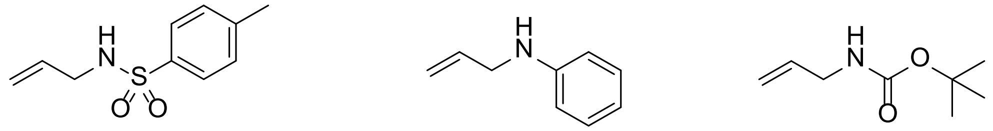

N-allyl-4-methylbenzenesulfonamide *N*-allylaniline *tert*-butyl allylcarbamate

Likewise, the following allylic alcohols also failed to undergo the conjugate addition step (monitored by LCMS) due to steric hinderance.

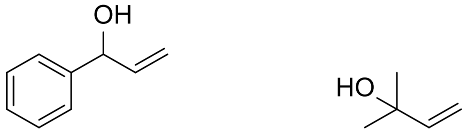

1-phenylprop-2-en-1-ol 2-methylbut-3-en-2-ol

The following longer chain alkenyl alcohols were also tested in the reaction (General Procedure D) but yielded no bridged or fused products:

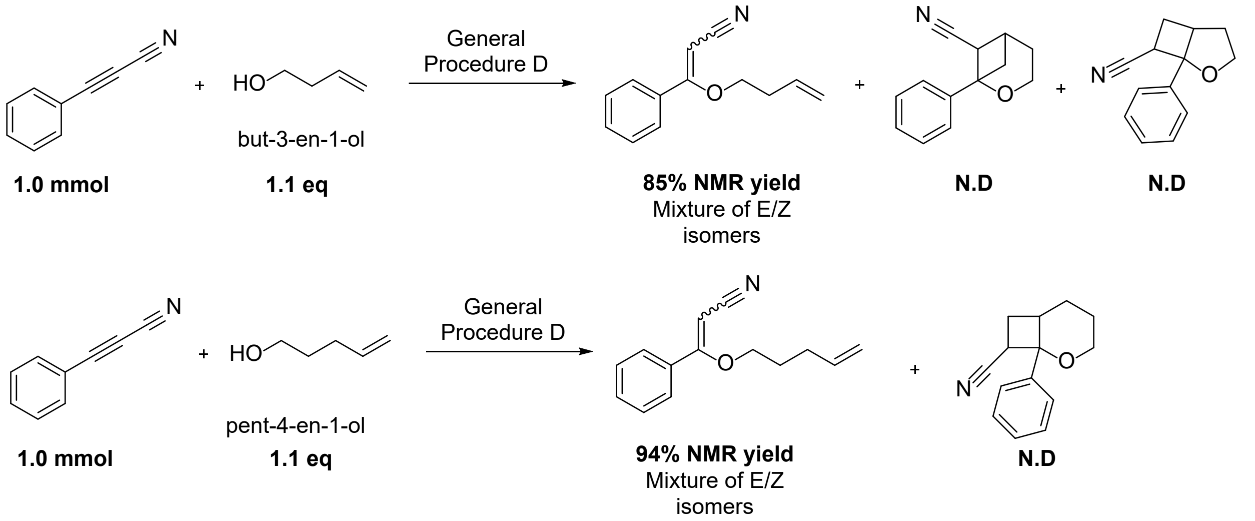

X-Ray Structure of 3e

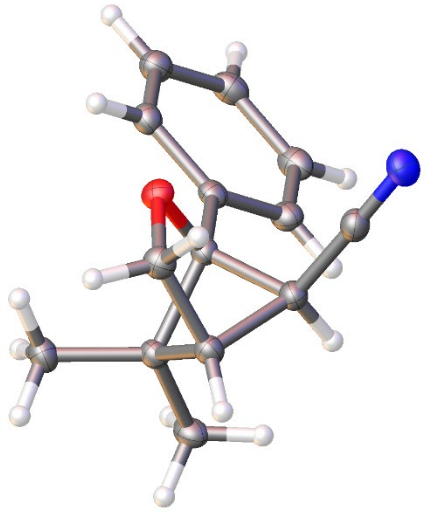

Deposition Number: 2382092

Experimental. Single clear colourless block-shaped crystal recrystallised from a mixture of acetonitrile and diethylether by slow evaporation. A suitable crystal with dimensions 0.16 × 0.07 × 0.05 mm^3 was selected and mounted on a MITIGEN holder in perfluoroether oil on a XtaLAB Synergy, Dualflex, HyPix-Arc 100 diffractometer. The crystal was kept at a steady T = 100(2) K during data collection. The structure was solved with the ShelXT 2018/2 (Sheldrick, 2018) solution program using dual methods and by using Olex2 1.5 (Dolomanov et al., 2009) as the graphical interface. The model was refined with ShelXL 2018/3 (Sheldrick, 2015) using full matrix least squares minimisation on $\boldsymbol{F^2}$.

Crystal Data. $C_{14}H_{15}NO$, M_r = 213.27, monoclinic, $P2_1/c$ (No. 14), a = 11.83321(10) Å, b = 6.75791(5) Å, c = 14.58577(12) Å, b = 106.2749(9)$^{\circ}$, a = g = 90°, V = 1119.653(17) $Å^3$, T = 100(2) K, Z = 4, Z' = 1, m(Cu K_a) = 0.624, 44123 reflections measured, 2383 unique (R_{int} = 0.0222) which were used in all calculations. The final wR_2 was 0.0804 (all data) and R_1 was 0.0332 (I≥2 s(I)).

**Formula**	$C_{14}H_{15}NO$
D $D_{calc.}$**/ g cm^{-3}**	1.265
***m*/mm^{-1}**	0.624
**Formula Weight**	213.27
**Colour**	clear colourless
**Shape**	block-shaped
**Size/mm^3**	0.16×0.07×0.05
***T*/K**	100(2)
**Crystal System**	monoclinic
**Space Group**	$P2_1/c$
***a*/Å**	11.83321(10)
***b*/Å**	6.75791(5)
***c*/Å**	14.58577(12)
***a*/°**	90
***b*/°**	106.2749(9)
***g*/°**	90
**V/Å^3**	1119.653(17)
Z	4
Z'	1
**Wavelength/Å**	1.54184
**Radiation type**	Cu K_{α}
**Q_{min}/°**	3.892
**Q_{max}/°**	78.687
**Measured Refl's.**	44123
**Indep't Refl's**	2383
**Refl's I≥2 *s*(I)**	2310
**R_{int}**	0.0222
**Parameters**	148
**Restraints**	0
**Largest Peak**	0.309
**Deepest Hole**	-0.181
**GooF**	1.051
**wR_2 (all data)**	0.0804
**wR_2**	0.0800
**R_1 (all data)**	0.0339
**R_1**	0.0332

X-Ray Structure of 3q

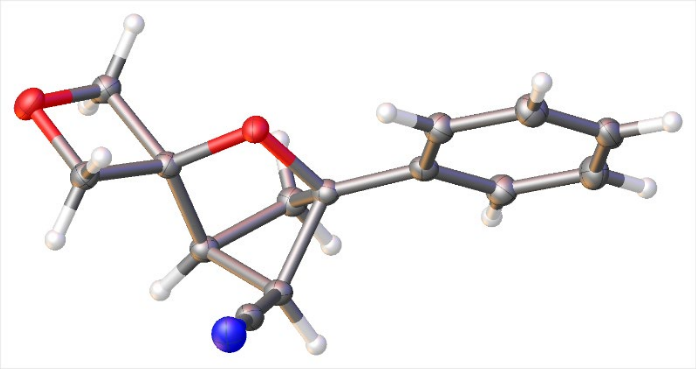

Deposition Number 2382093

Experimental. Single clear colourless block-shaped crystals recrystallised from chloroform by slow evaporation. A suitable crystal with dimensions 0.16 × 0.09 × 0.02 mm^3 was selected and mounted on a MITIGEN holder in perfluoroether oil on a XtaLAB Synergy, Dualflex, HyPix-Arc 100 diffractometer. The crystal was kept at a steady T = 100.00(10) K during data collection. The structure was solved with the ShelXT 2018/2 (Sheldrick, 2018) solution program using dual methods and by using Olex2 1.5 (Dolomanov et al., 2009) as the graphical interface. The model was refined with ShelXL 2018/3 (Sheldrick, 2015) using full matrix least squares minimisation on $\boldsymbol{F^2}$.

Crystal Data. $C_{14}H_{13}NO_2$, M_r = 227.25, orthorhombic, $P2_12_12_1$ (No. 19), a = 5.72327(3) Å, b = 7.75796(5) Å, c = 24.60196(14) Å, $a = b = g = 90^{\circ}$, V = 1092.349(11) Å^3, T = 100.00(10) K, Z = 4, Z' = 1, m(Cu K$_a$) = 0.750, 46038 reflections measured, 2327 unique (R_{int} = 0.0268) which were used in all calculations. The final wR_2 was 0.0626 (all data) and R_1 was 0.0245 (I≥2 s(I))

**Formula**	$C_{14}H_{13}NO_2$
$D_{calc.}$**/ g cm^{-3}**	1.382
***m*/mm^{-1}**	0.750
**Formula Weight**	227.25
**Colour**	clear colourless
**Shape**	block-shaped
**Size/mm^3**	0.16×0.09×0.02
***T*/K**	100.00(10)
**Crystal System**	orthorhombic
**Flack Parameter**	-0.02(3)
**Hooft Parameter**	-0.03(2)
**Space Group**	$P2_12_12_1$
***a*/Å**	5.72327(3)
***b*/Å**	7.75796(5)
***c*/Å**	24.60196(14)
***a*/°**	90
***b*/°**	90
***g*/°**	90
**V/Å^3**	1092.349(11)
Z	4
Z'	1
**Wavelength/Å**	1.54184
**Radiation type**	Cu K_a
Q_{min}**/°**	3.593
Q_{max}**/°**	77.502
**Measured Refl's.**	46038
**Indep't Refl's**	2327
**Refl's I≥2 *s*(I)**	2300
R_{int}	0.0268
**Parameters**	155
**Restraints**	0
**Largest Peak**	0.192
**Deepest Hole**	-0.142
**GooF**	1.061
wR_2 **(all data)**	0.0626
wR_2	0.0624
R_1 **(all data)**	0.0247
R_1	0.0245

Mechanistic Studies

Monitoring the formation of a likely phosphonium intermediate

^{31}P NMR of tributylphosphine in DCM-d_2:

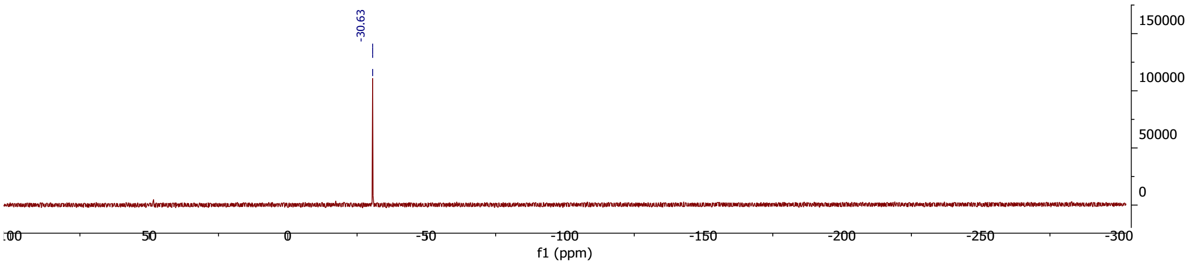

A 4 mL vial was charged with stirrer bar and 3-phenylpropiolonitrile (0.025 mL, 0.2 mmol) and underwent three vacuum/N_2 cycles then DCM-d_2 (1 mL) was added, followed by addition of tributylphosphine (2.498 µl, 10.00 µmol) under nitrogen. The reaction was stirred for 10 mins at room temperature.

^{31}P NMR was taken directly of the reaction mixture:

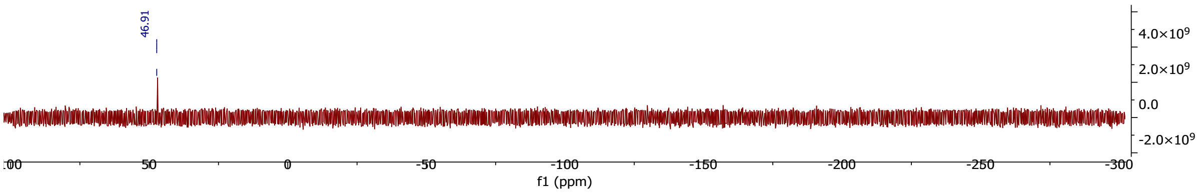

Confirmation of diene intermediate and its geometry

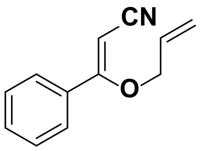

(*Z*)-3-(allyloxy)-3-phenylacrylonitrile (6a)

A 40 mL vial was charged with 3-phenylpropiolonitrile (0.127 mL, 1 mmol) and underwent three vacuum/N_2 cycles. DCM (5 mL) was added followed by prop-2-en-1-ol (0.075 mL, 1.10 mmol) and tributylphosphine (0.012 mL, 0.05 mmol). The reaction was stirred at room temperature for 1 hour. The reaction mixture was purified via flash column chromatography (SiO_2, EtOAc in heptane, 0 – 100% gradient, R_f = 0.62 at 50% EtOAc in heptane), giving the product as a colourless oil.

^{1}H NMR (500 MHz, CD_2Cl_2) 4.81 (dt, J = 5.5,1.4 Hz, 2H), **4.92 (s, 1H)**, 5.23 - 5.27 (m, 2H), 5.34 (dq, J = 17.3, 1.5 Hz, 1H), 5.97 (ddt, J = 17.2, 10.6, 5.4 Hz, 1H), 7.30 - 7.36 (m, 2H), 7.37 - 7.43 (m, 1H), 7.44 - 7.49 (m, 1H).

^{1}H NMR (600 MHz, **$CDCl_3$**) 4.95 (dt, J = 5.5, 1.5 Hz, 2H), **4.98 (s, 1H)**, 5.34 (ddd, J = 10.5, 1.3, 1.3 Hz, 1H), 5.44 (ddd, J = 17.2, 1.5, 1.5 Hz, 1H), 6.04 (ddt, J = 17.2, 10.8, 5.5 Hz, 1H), 7.39 – 7.43 (m, 2H), 7.45 – 7.49 (m, 1H), 7.52 – 7.56 (m, 2H).

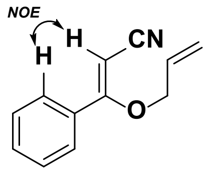

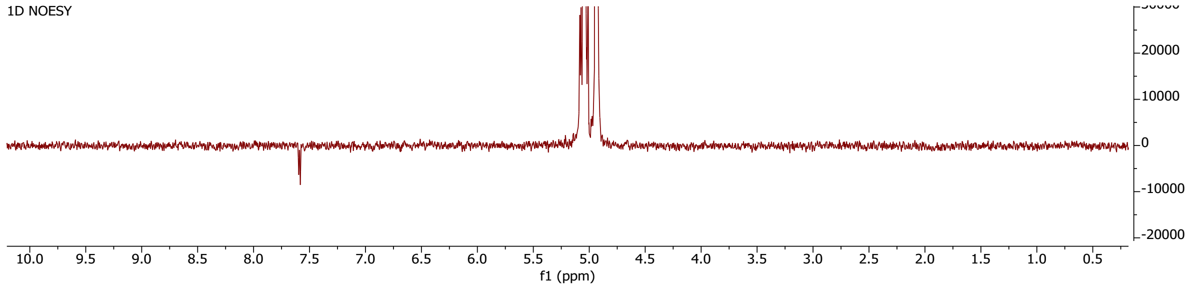

Literature comparison for ^{1}H NMR comparison of E/Z isomers

Org. Lett. **2016**, *18*, 4916–4919

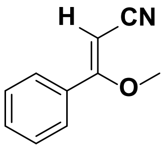

(*Z*)-3-methoxy-3-phenylacrylonitrile

^{1}H NMR (CDCl3, 400 MHz) δ: 7.53-7.36 (m, 5H), **4.94 (s, 1H)**, 4.24 (s, 3H).

Eur. J. Org. Chem. **2007**, 3122–3132

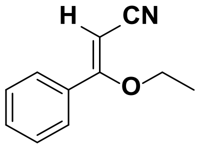

(*Z*)-3-ethoxy-3-phenylacrylonitrile

^{1}H NMR (300 MHz, $CDCl_3$) 7.58–7.30 (m, 5 H), **4.94 (s, 1 H)**, 4.54 (q, *J* = 7.0 Hz, 2 H), 1.45 (t, *J* = 7.0 Hz, 3 H).

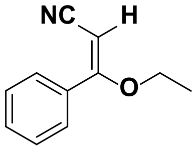

(*E*)-3-ethoxy-3-phenylacrylonitrile

^{1}H NMR (300 MHz, $CDCl_3$) 7.75 (d, J = 8.2 Hz, 2 H), (m, 3 H), **4.60 (s, 1 H)**, 3.99 (q, J = 7.0 Hz, 2 H), 1.46 (t, J = 7.0 Hz, 3 H)

Reiteration of relevant optimisation data

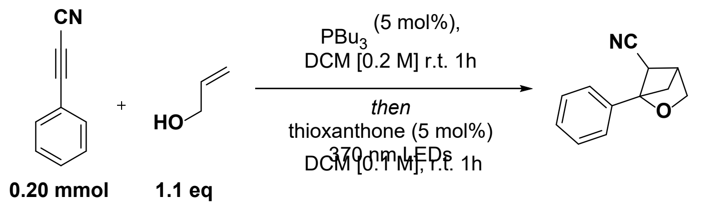

Entry	Change from above	Product ^{1}H NMR	d.r.
**1**	No PBu3	No product	n.d.
2	No Photocatalyst	<5%	n.d.
3	No Irradiation	No product	n.d.

Photochemistry proceeding in the absence of trialkylphosphine:

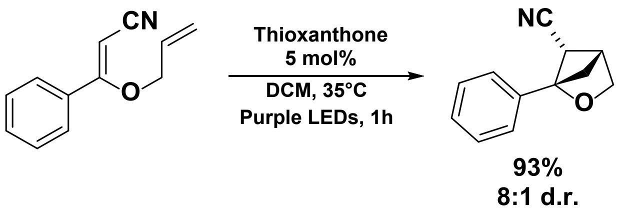

An 8 mL vial was charged with **6a** (37 mg, 0.20 mmol) and 9H-thioxanthen-9-one (2.1 mg, 0.001 mmol, 5 mol%) and underwent three vacuum/nitrogen cycles. DCM (4 mL) was added under nitrogen and the reaction mixture was irradiated for 1 hour with 2 x Kessil LEDs (370 nm Gen 2) at 35 °C. The solvent was removed under reduced pressure and ^{1}H NMR showed a 93% ^{1}H NMR yield, 8:1 d.r., suggesting that the tributylphosphine catalyst is not involved in the photochemical stage of the reaction.

Yield of product given over extended reaction times in the absence of phosphine or thioxanthone (photocatalyst):

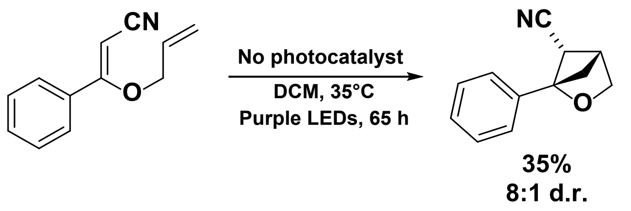

An 8 mL vial was charged with **6a** (37 mg, 0.20 mmol) and underwent three vacuum/nitrogen cycles. DCM (4 mL) was added under nitrogen and the reaction mixture was irradiated for 65 hours with 2 x Kessil LEDs (370 nm Gen 2) at 35 °C. The solvent was removed under reduced pressure and ^{1}H NMR showed a 35% ^{1}H NMR yield, 8:1 d.r., suggesting that the photochemical stage of the reaction can proceed in the absence of any potential redox process.

Probing the possibility of isomerisation of the diene intermediate under the reaction conditions:

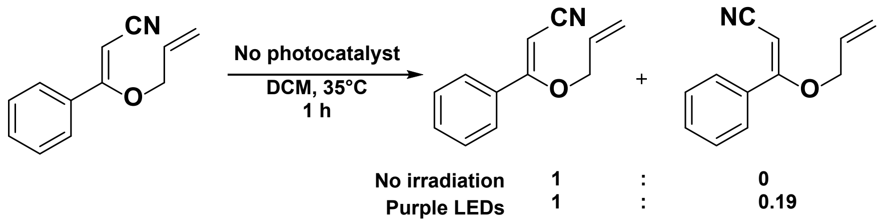

An 10 mL round-bottom flask was charged with **6a** (c.a. 10 mg) and DCM-d_2 (2 mL), fitted with a reflux condenser and stirred at 35 °C for 1 hour. ^{1}H NMR of the reaction mixture showed no isomerisation of **6a**. This suggests that under the heat of the LEDs alone, the diene intermediate cannot isomerise within 1 hour.

6a (c.a 5 mg) was dissolved in 300 microlitres of DCM-D2 and the reaction mixture syringed into a 3 mm NMR tube. The NMR tube was irradiated for 1 hour by 2 x Kessil LEDs (370 nm Gen 2). ^{1}H NMR analysis showed a 1:0.19 ratio of *Z* to *E*, suggesting that small amounts of diene isomerisation mediated by the 370 nm LEDs is possible but not significant during the reaction.

Computational Experiments Modelling the triplet energies of thioxanthone, 6a and 6b

All calculations were carried out with Gaussian16 [Gaussian, Inc., Wallingford CT, 2016].

Initially the geometries of the molecules were optimised using ground state density functional theory (DFT) in acetonitrile. The optimised geometries were then used as input coordinates to calculate the first exited state triplet energies using time-dependent density functional theory (TD-DFT) with no solvent model [as also reported by: https://pubs.acs.org/doi/10.1021/jacs.0c05069?goto=supporting-info#_i15].

Optimised geometries of thioxanthone

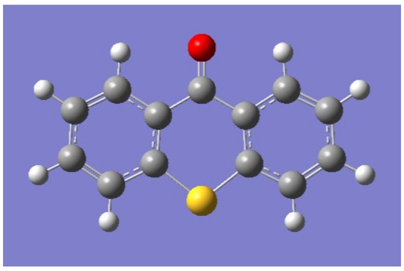

# opt b3lyp/6-31g(d,p)
scrf=(cpcm,solvent=acetonitrile)
geom=connectivity
Charge=0
Multiplicity=1
Energy = -973.65134 Hartree

```
   Atomic Coordinates (Angstroms)
 -----------------------------------
 C     -5.946241 -0.107229 -0.419175
 C     -4.565877 -0.000662 -0.415007
 C     -3.924177  1.255387 -0.416672
 C     -4.723658  2.415108 -0.422674
 C     -6.126117  2.306664 -0.426945
 C     -6.729240  1.057108 -0.425197
```

C	-2.439699	1.265012	-0.411918
C	-2.333809	3.820014	-0.418081
C	-1.709320	2.557424	-0.412373
C	-0.299749	2.507244	-0.406724
H	0.162404	1.526776	-0.402358
C	0.464700	3.661540	-0.406778
C	-0.171840	4.911975	-0.412588
C	-1.556976	4.992703	-0.418190
H	-6.419448	-1.083675	-0.417802
H	-3.933952	-0.881327	-0.410334
H	-6.735698	3.205142	-0.431557
H	-7.812525	0.987280	-0.428499
H	1.548020	3.600122	-0.402358
H	0.416027	5.824557	-0.412699
H	-2.045562	5.962309	-0.422622
O	-1.813463	0.199741	-0.407047
S	-4.080513	4.056182	-0.425543

First exited state triplet energy of thioxanthone

# td=(triplets,nstates=5) b3lyp/6-31g(d,p)
geom=connectivity
Charge=0
Multiplicity=1
Exited state 1: Triplet-A 2.8191 eV (65 kcal/mol)

Optimised geometries of 6a

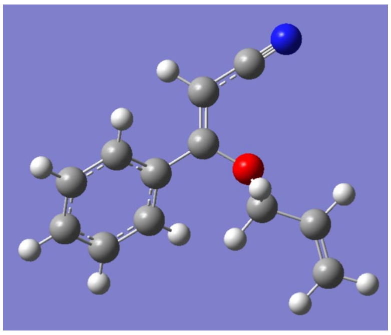

# opt b3lyp/6-31g(d,p)
scrf=(cpcm,solvent=acetonitrile)
geom=connectivity
Charge=0
Multiplicity=1
Energy = -593.83661 Hartree

Atomic Coordinates (Angstroms)

C	-2.116729	-1.758243	0.417839
C	-0.942257	-1.697406	-0.337390
C	-0.421979	-0.464330	-0.723608
C	-1.077690	0.726270	-0.363370
C	-2.255547	0.656495	0.398402
C	-2.771024	-0.579813	0.784521
H	-2.518246	-2.720105	0.721513
H	-0.433902	-2.611061	-0.629178

H	0.479892	-0.417109	-1.325573
H	-2.752317	1.571829	0.703897
H	-3.678370	-0.622375	1.378835
C	-0.544251	2.028206	-0.818578
C	-1.346733	3.034092	-1.254588
H	-2.416107	2.883482	-1.340736
C	-0.832394	4.294590	-1.652885
N	-0.443419	5.342792	-1.985433
O	0.800624	2.200401	-0.901728
C	1.609856	1.876387	0.267421
H	1.560706	0.806643	0.485198
H	1.199054	2.428043	1.122376
C	3.011366	2.309407	-0.026756
H	3.136125	3.354686	-0.303432
C	4.067006	1.501597	0.066950
H	3.962358	0.454357	0.339701
H	5.075000	1.860828	-0.116282

First exited state triplet energy of 6a

# td=(triplets,nstates=5) b3lyp/6-31g(d,p)
geom=connectivity
Charge=0
Multiplicity=1
Exited state 1: Triplet-A 2.7645 eV (63.8 kcal/mol)

Optimised geometries of 6b

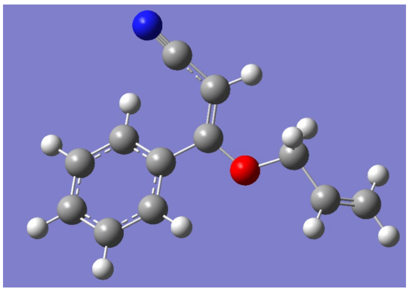

# opt b3lyp/6-31g(d,p)
scrf=(cpcm,solvent=acetonitrile)
geom=connectivity
Charge=0
Multiplicity=1
Energy= -593.840447 Hartree

Atomic Coordinates (Angstroms)

C	-2.428496	-1.808747	-0.153426
C	-1.139184	-1.721410	0.378067
C	-0.484052	-0.493158	0.426964
C	-1.119229	0.669592	-0.042622
C	-2.409665	0.571791	-0.587067
C	-3.058560	-0.661142	-0.639159
H	-2.936526	-2.767396	-0.194717
H	-0.642990	-2.610813	0.753946
H	0.517647	-0.424589	0.835902
H	-2.898154	1.451871	-0.988858
H	-4.053189	-0.725514	-1.069168
C	-0.399724	1.963570	0.021340

C	-0.957953	3.178039	0.299589
H	-0.359134	4.080234	0.282749
C	-2.311250	3.367896	0.667704
N	-3.411958	3.588848	0.988381
O	0.915157	1.802455	-0.217350
C	1.818631	2.923653	-0.065275
H	1.582399	3.689074	-0.812583
H	1.672255	3.349989	0.935793
C	3.213587	2.409116	-0.236102
H	3.509566	1.609086	0.439891
C	4.075907	2.901833	-1.124105
H	5.093614	2.530431	-1.192873
H	3.798468	3.700555	-1.807469

First exited state triplet energy of 6b

# td=(triplets,nstates=5) b3lyp/6-31g(d,p)
geom=connectivity
Charge=0
Multiplicity=1
Exited state 1: Triplet-A 2.9682 eV (68.4 kcal/mol)

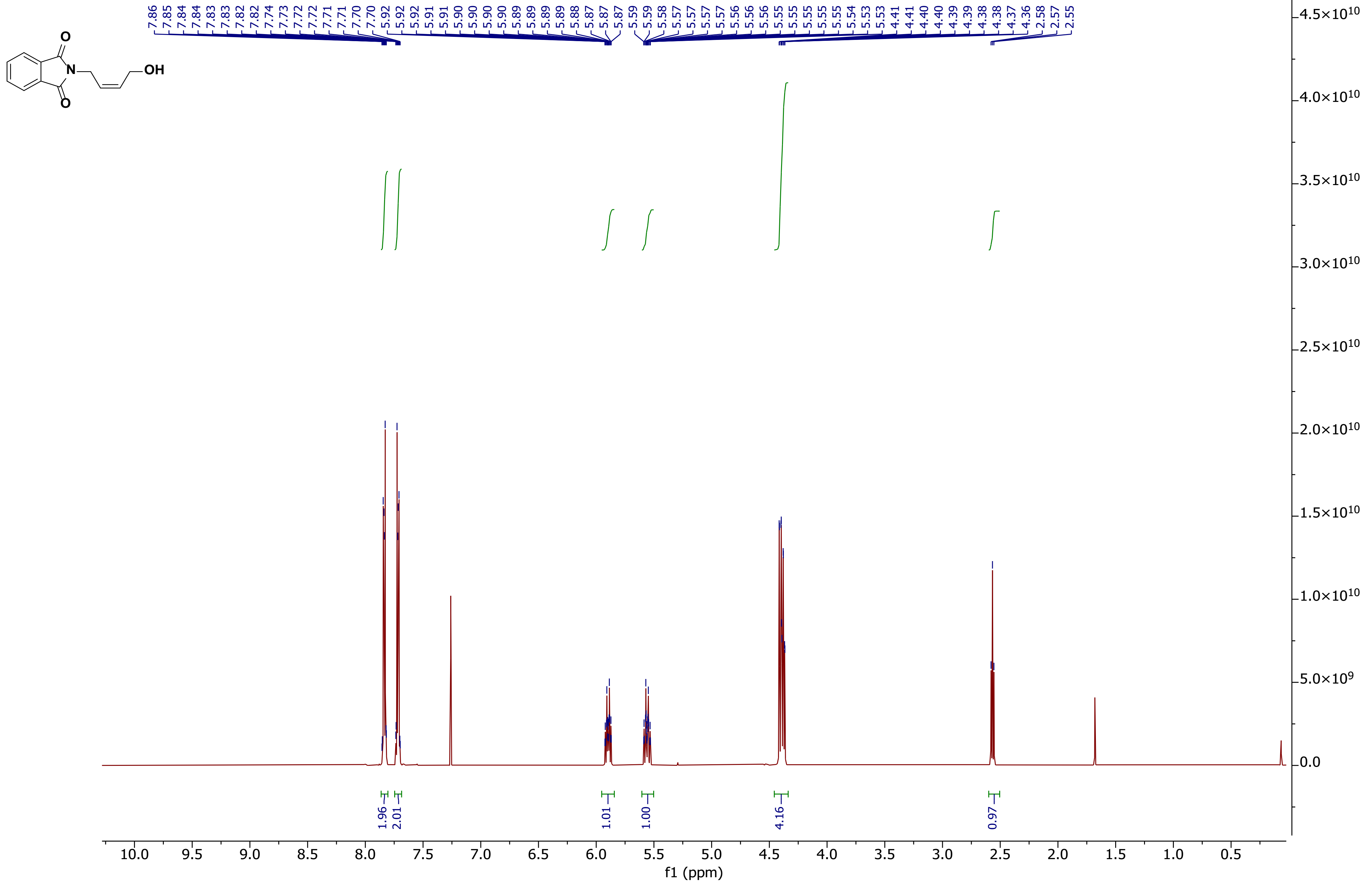

OH
N
O
O
7.86 7.85 7.84 7.84 7.83 7.83 7.82 7.82 7.74 7.73 7.72 7.72 7.71 7.71 7.70 7.70 5.92 5.92 5.92 5.91 5.91 5.90 5.90 5.90 5.90 5.89 5.89 5.89 5.89 5.88 5.87 5.87 5.87 5.59 5.59 5.58 5.57 5.57 5.57 5.57 5.56 5.56 5.56 5.55 5.55 5.55 5.55 5.55 5.54 5.53 5.53 4.41 4.41 4.40 4.39 4.39 4.38 4.38 4.37 4.36 2.58 2.57 2.55
1.96
2.01
1.01
1.00
4.16
0.97
10.0 9.5 9.0 8.5 8.0 7.5 7.0 6.5 6.0 5.5 5.0 4.5 4.0 3.5 3.0 2.5 2.0 1.5 1.0 0.5
f1 (ppm)
4.5×10^10
4.0×10^10
3.5×10^10
3.0×10^10
2.5×10^10
2.0×10^10
1.5×10^10
1.0×10^10
5.0×10^9
0.0

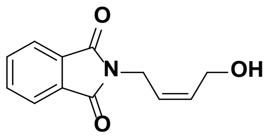

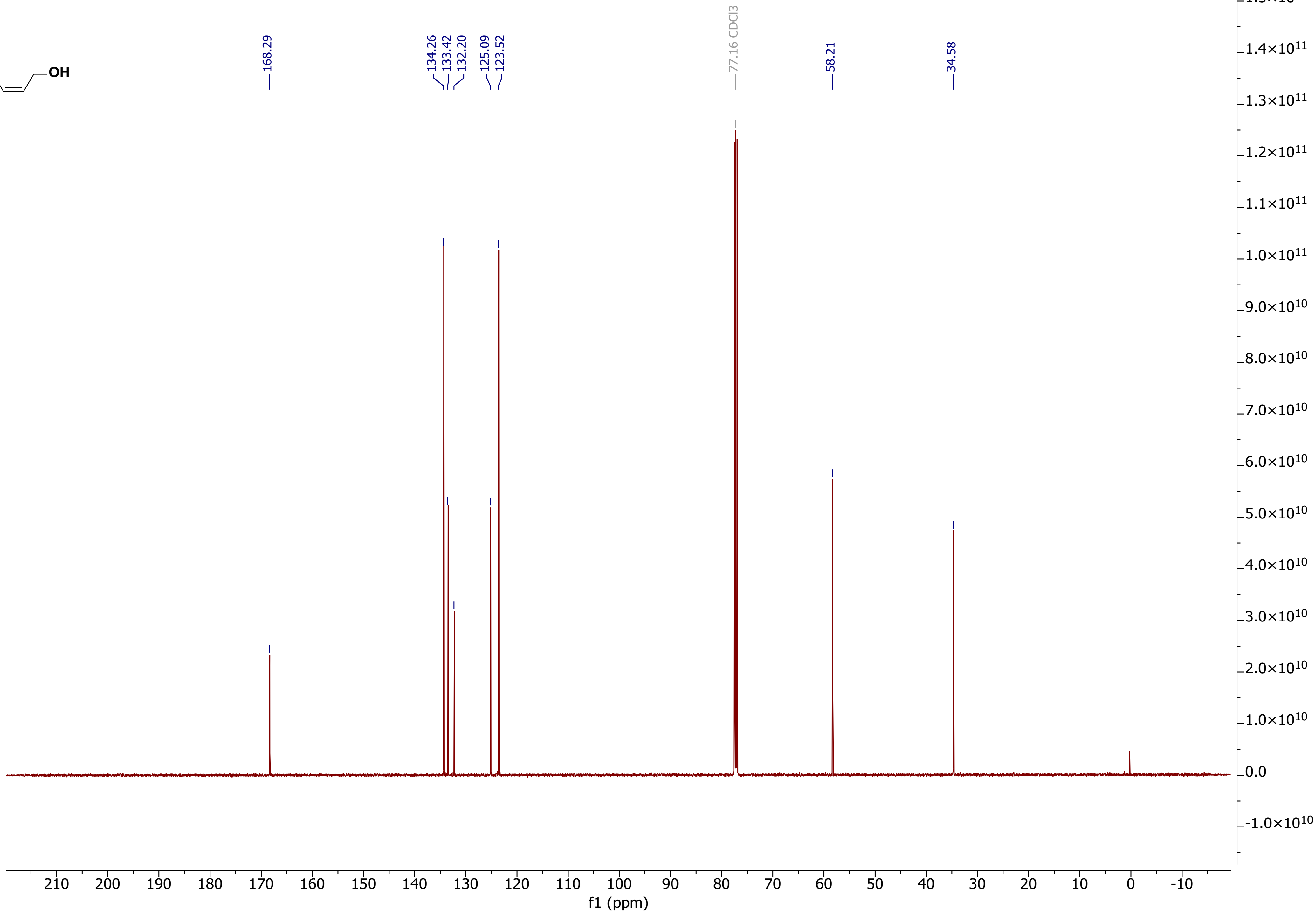
168.29
134.26
133.42
132.20
125.09
123.52
77.16 CDCl3
58.21
34.58
f1 (ppm)

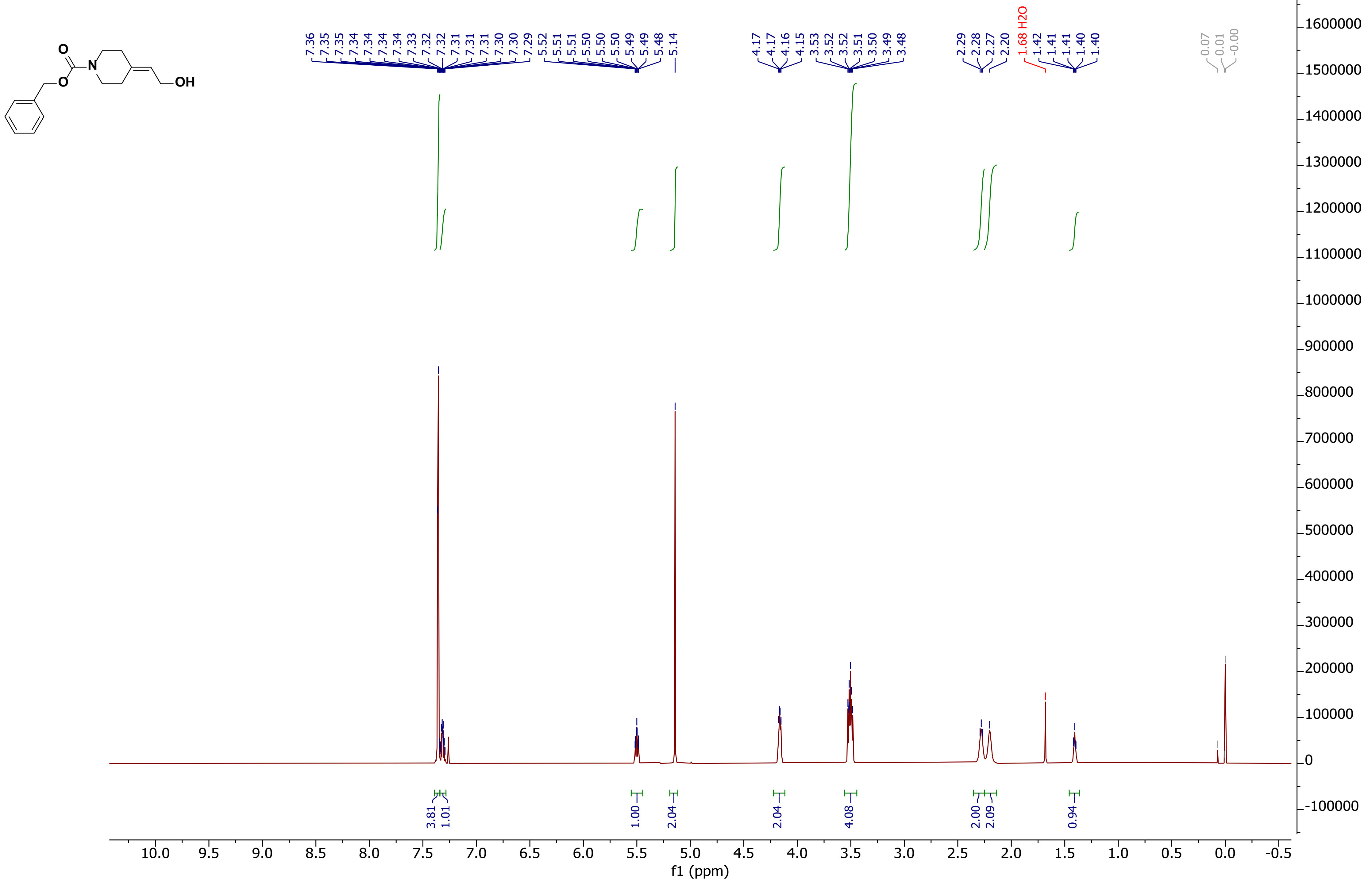
O
N
O
OH
7.36 7.35 7.35 7.34 7.34 7.34 7.34 7.33 7.32 7.32 7.31 7.31 7.31 7.30 7.30 7.29
5.52 5.51 5.51 5.50 5.50 5.50 5.49 5.49 5.48
5.14
4.17 4.17 4.16 4.15
3.53 3.52 3.52 3.51 3.50 3.49 3.48
2.29 2.28 2.27 2.20
1.68 H2O
1.42 1.41 1.41 1.40 1.40
0.07 0.01 -0.00
3.81 1.01 1.00 2.04 2.04 4.08 2.00 2.09 0.94
f1 (ppm)

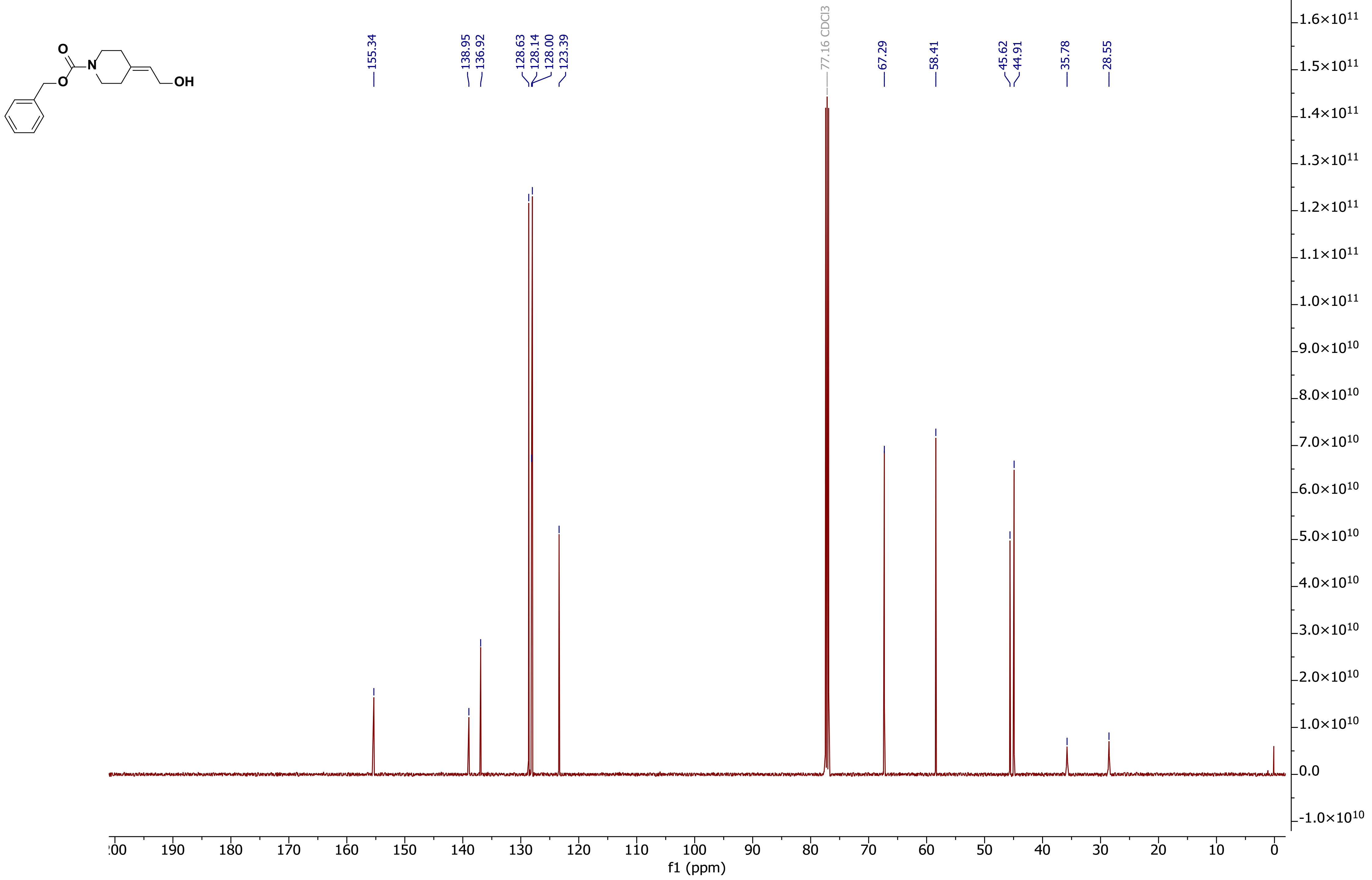
O
N
O
OH
155.34
138.95
136.92
128.63
128.14
128.00
123.39
77.16 CDCl3
67.29
58.41
45.62
44.91
35.78
28.55
1.6×10^11
1.5×10^11
1.4×10^11
1.3×10^11
1.2×10^11
1.1×10^11
1.0×10^11
9.0×10^10
8.0×10^10
7.0×10^10
6.0×10^10
5.0×10^10
4.0×10^10
3.0×10^10
2.0×10^10
1.0×10^10
0.0
-1.0×10^10
200 190 180 170 160 150 140 130 120 110 100 90 80 70 60 50 40 30 20 10 0
f1 (ppm)

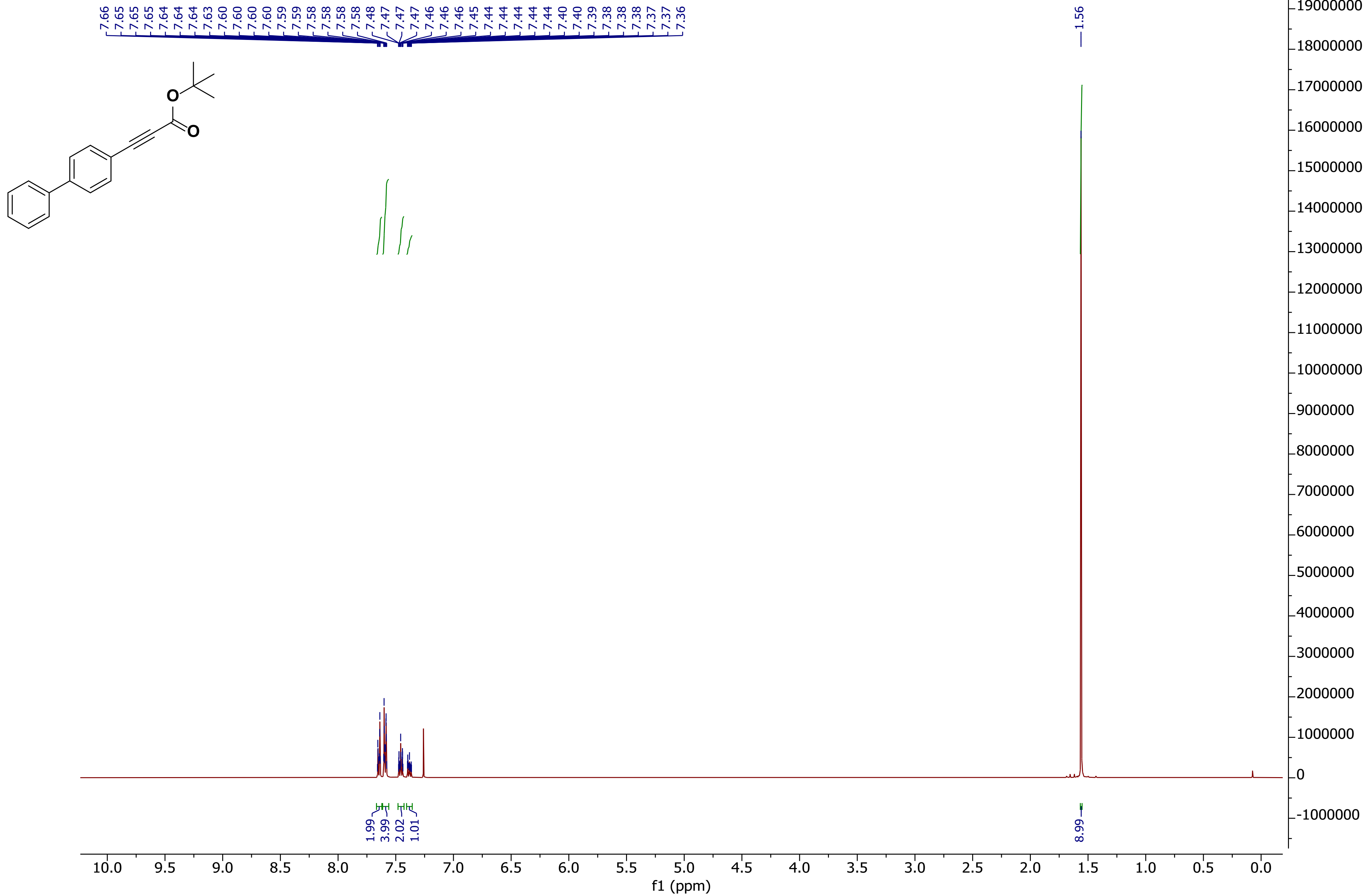

7.66 7.65 7.65 7.64 7.64 7.63 7.60 7.60 7.60 7.60 7.59 7.59 7.58 7.58 7.58 7.48 7.47 7.47 7.47 7.46 7.46 7.45 7.44 7.44 7.44 7.44 7.40 7.40 7.39 7.38 7.38 7.38 7.37 7.37 7.36
1.56
O
O
1.99
3.99
2.02
1.01
8.99
10.0 9.5 9.0 8.5 8.0 7.5 7.0 6.5 6.0 5.5 5.0 4.5 4.0 3.5 3.0 2.5 2.0 1.5 1.0 0.5 0.0
f1 (ppm)
19000000 18000000 17000000 16000000 15000000 14000000 13000000 12000000 11000000 10000000 9000000 8000000 7000000 6000000 5000000 4000000 3000000 2000000 1000000 0 -1000000

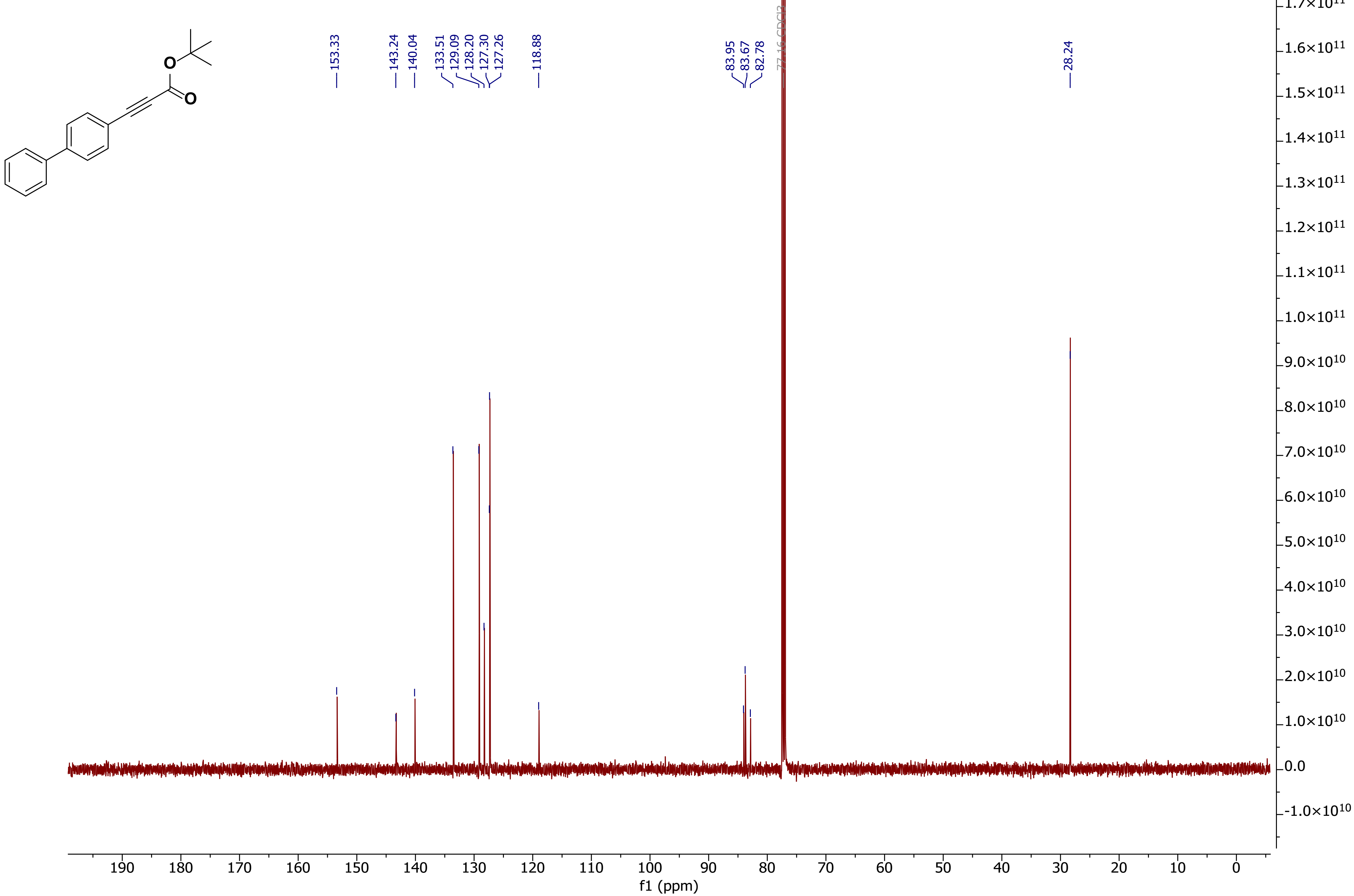

O
O
153.33
143.24
140.04
133.51
129.09
128.20
127.30
127.26
118.88
83.95
83.67
82.78
77.16 CDCl3
28.24
1.7×10^11
1.6×10^11
1.5×10^11
1.4×10^11
1.3×10^11
1.2×10^11
1.1×10^11
1.0×10^11
9.0×10^10
8.0×10^10
7.0×10^10
6.0×10^10
5.0×10^10
4.0×10^10
3.0×10^10
2.0×10^10
1.0×10^10
0.0
-1.0×10^10
190 180 170 160 150 140 130 120 110 100 90 80 70 60 50 40 30 20 10 0
f1 (ppm)

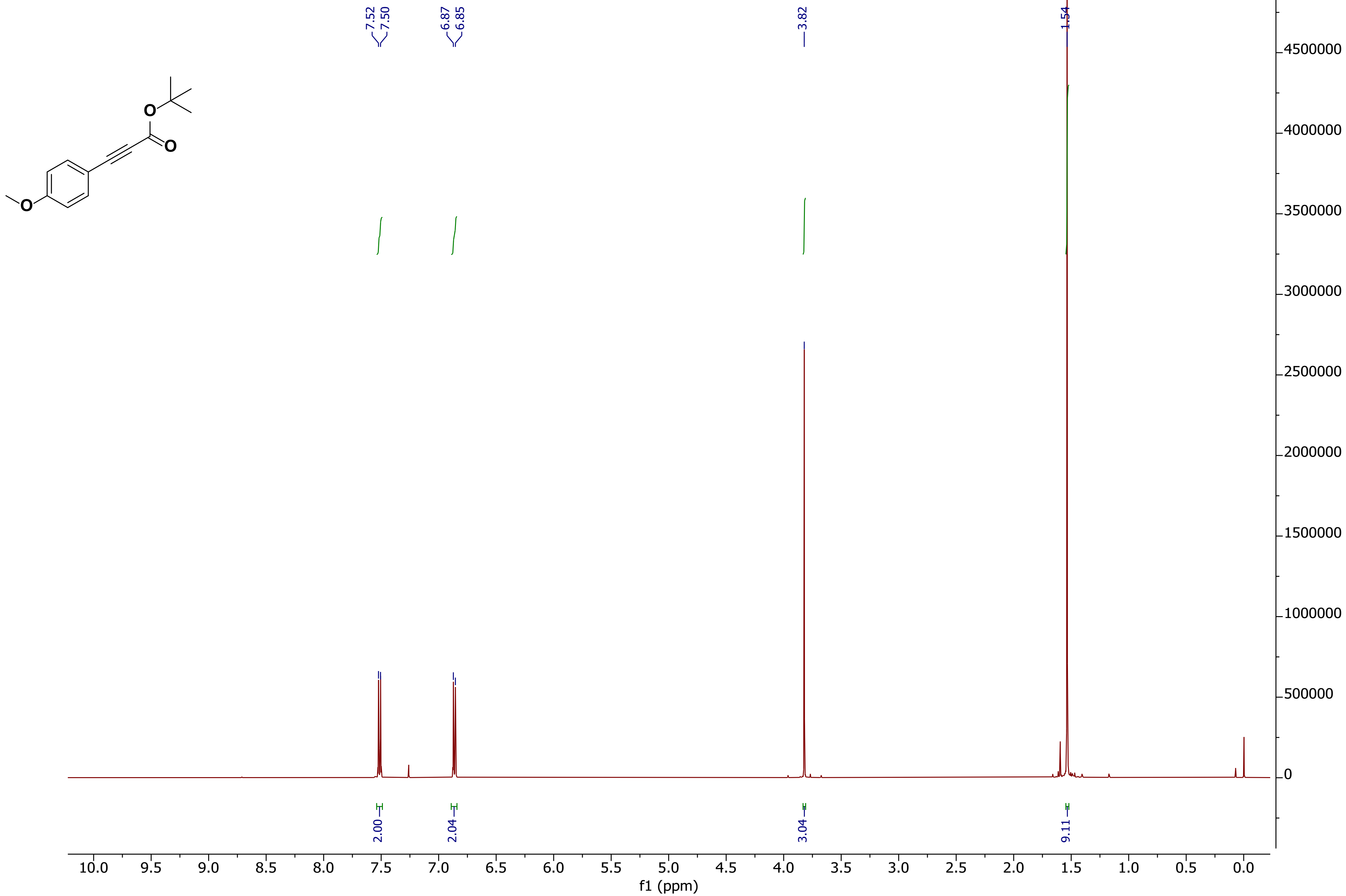
O
O
O
7.52
7.50
6.87
6.85
3.82
1.54
2.00
2.04
3.04
9.11
4500000
4000000
3500000
3000000
2500000
2000000
1500000
1000000
500000
0
10.0
9.5
9.0
8.5
8.0
7.5
7.0
6.5
6.0
5.5
5.0
4.5
4.0
3.5
3.0
2.5
2.0
1.5
1.0
0.5
0.0
f1 (ppm)

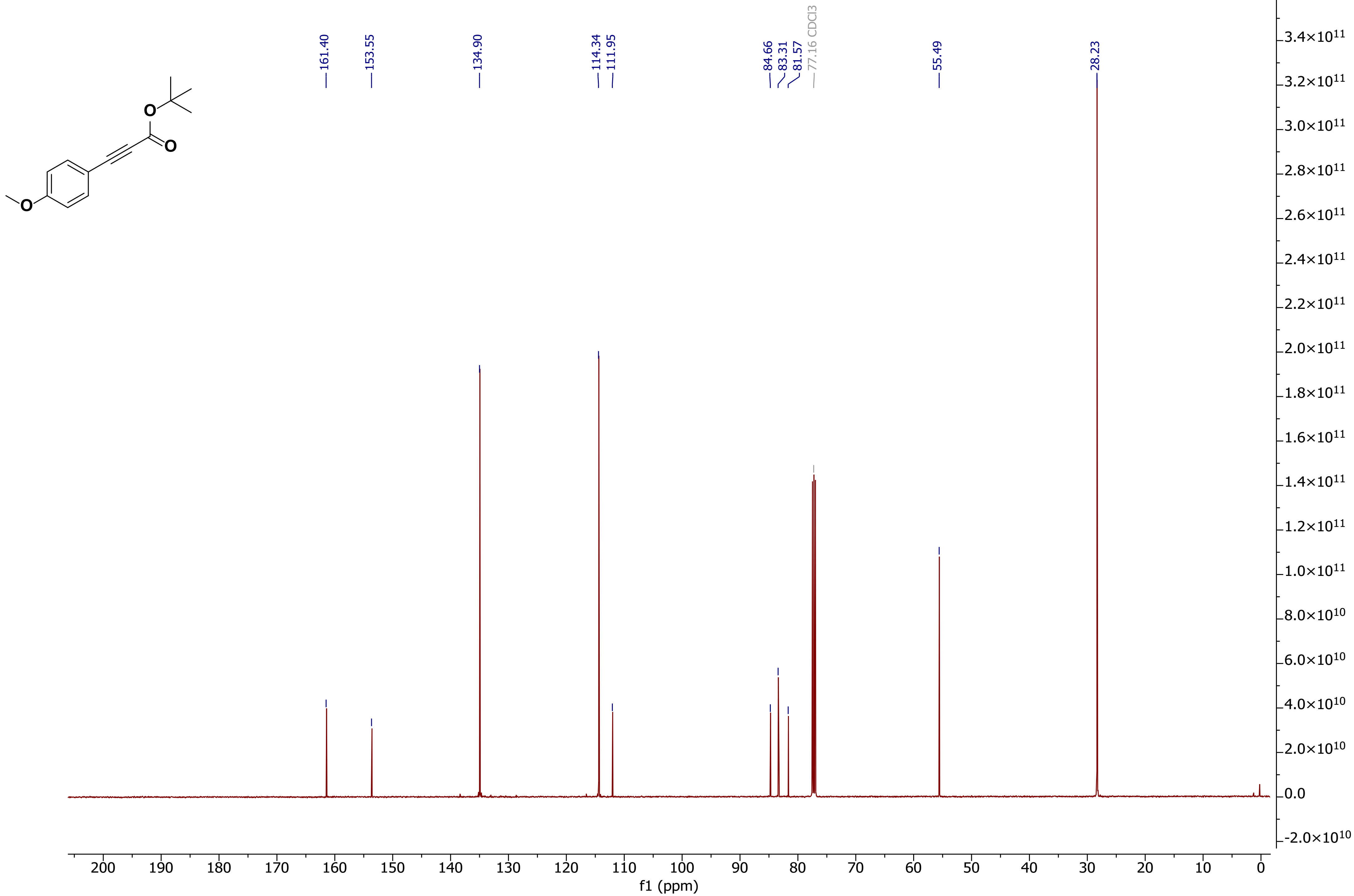
161.40
153.55
134.90
114.34
111.95
84.66
83.31
81.57
77.16 CDCl3
55.49
28.23
f1 (ppm)

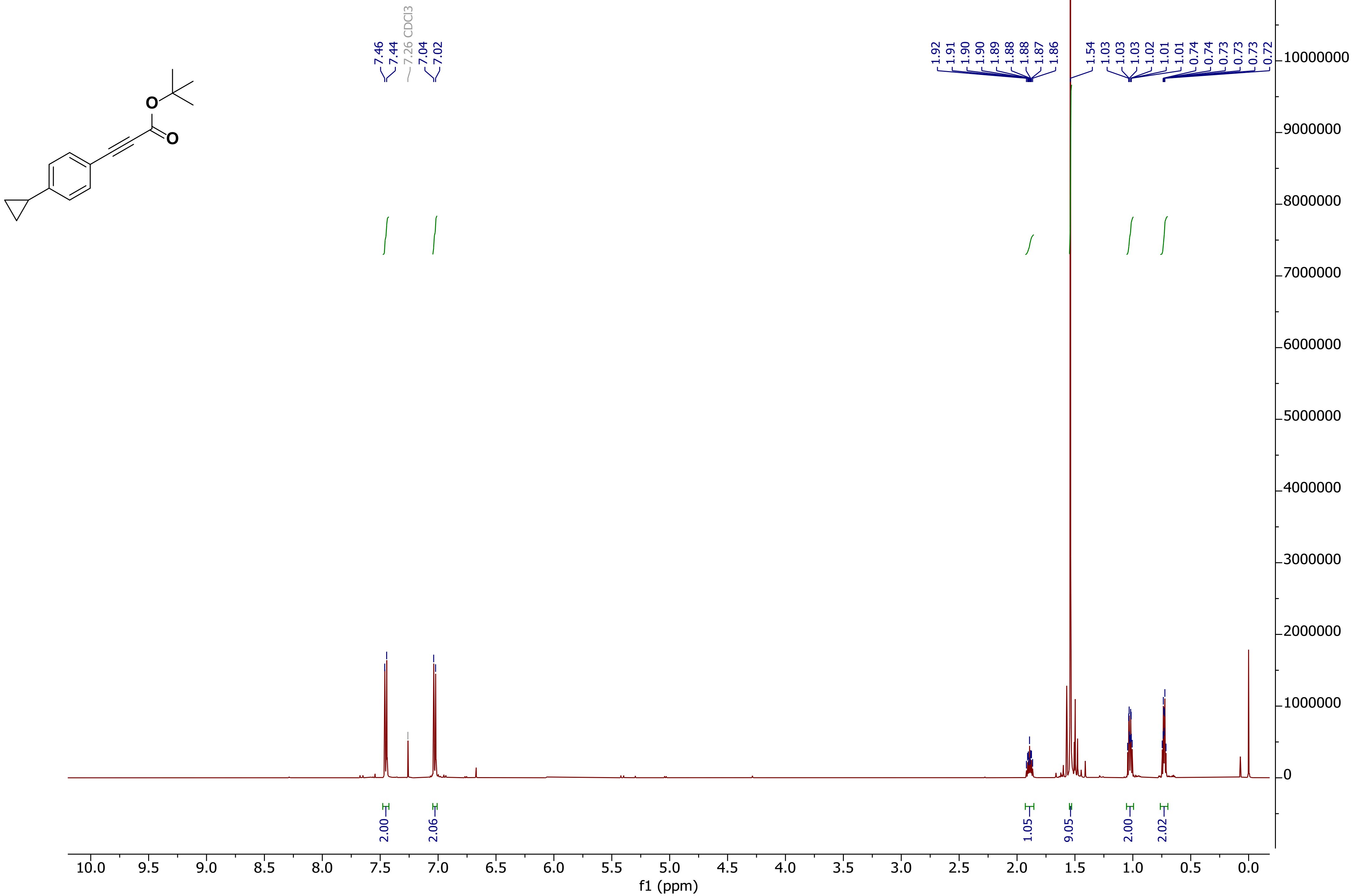
7.46
7.44
7.26 CDCl3
7.04
7.02
1.92
1.91
1.90
1.90
1.89
1.88
1.88
1.87
1.86
1.54
1.03
1.03
1.03
1.02
1.01
1.01
0.74
0.74
0.73
0.73
0.73
0.72
2.00
2.06
1.05
9.05
2.00
2.02
10.0 9.5 9.0 8.5 8.0 7.5 7.0 6.5 6.0 5.5 5.0 4.5 4.0 3.5 3.0 2.5 2.0 1.5 1.0 0.5 0.0
f1 (ppm)
10000000
9000000
8000000
7000000
6000000
5000000
4000000
3000000
2000000
1000000
0

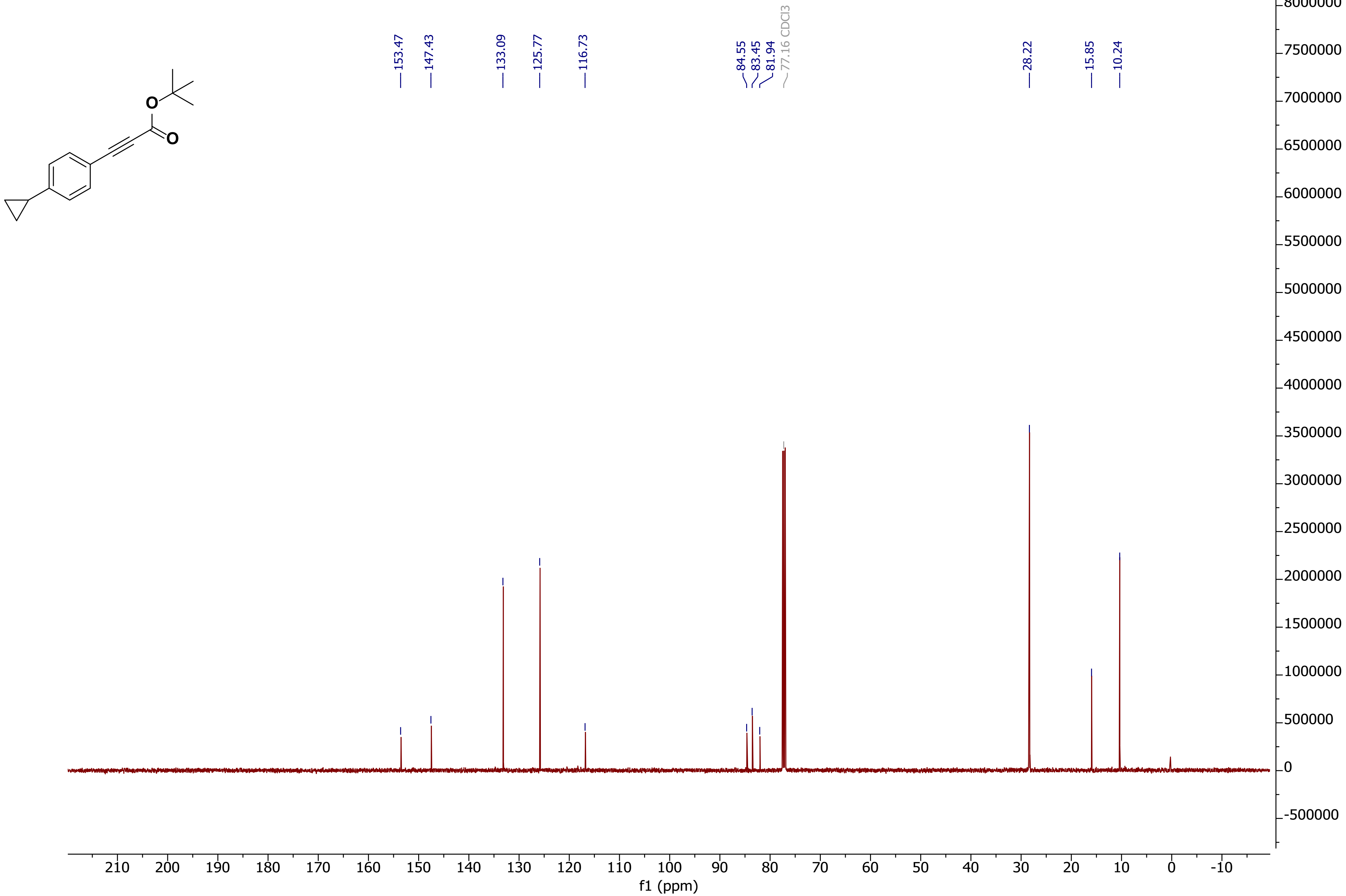

O
O
153.47
147.43
133.09
125.77
116.73
84.55
83.45
81.94
77.16 CDCl3
28.22
15.85
10.24
f1 (ppm)

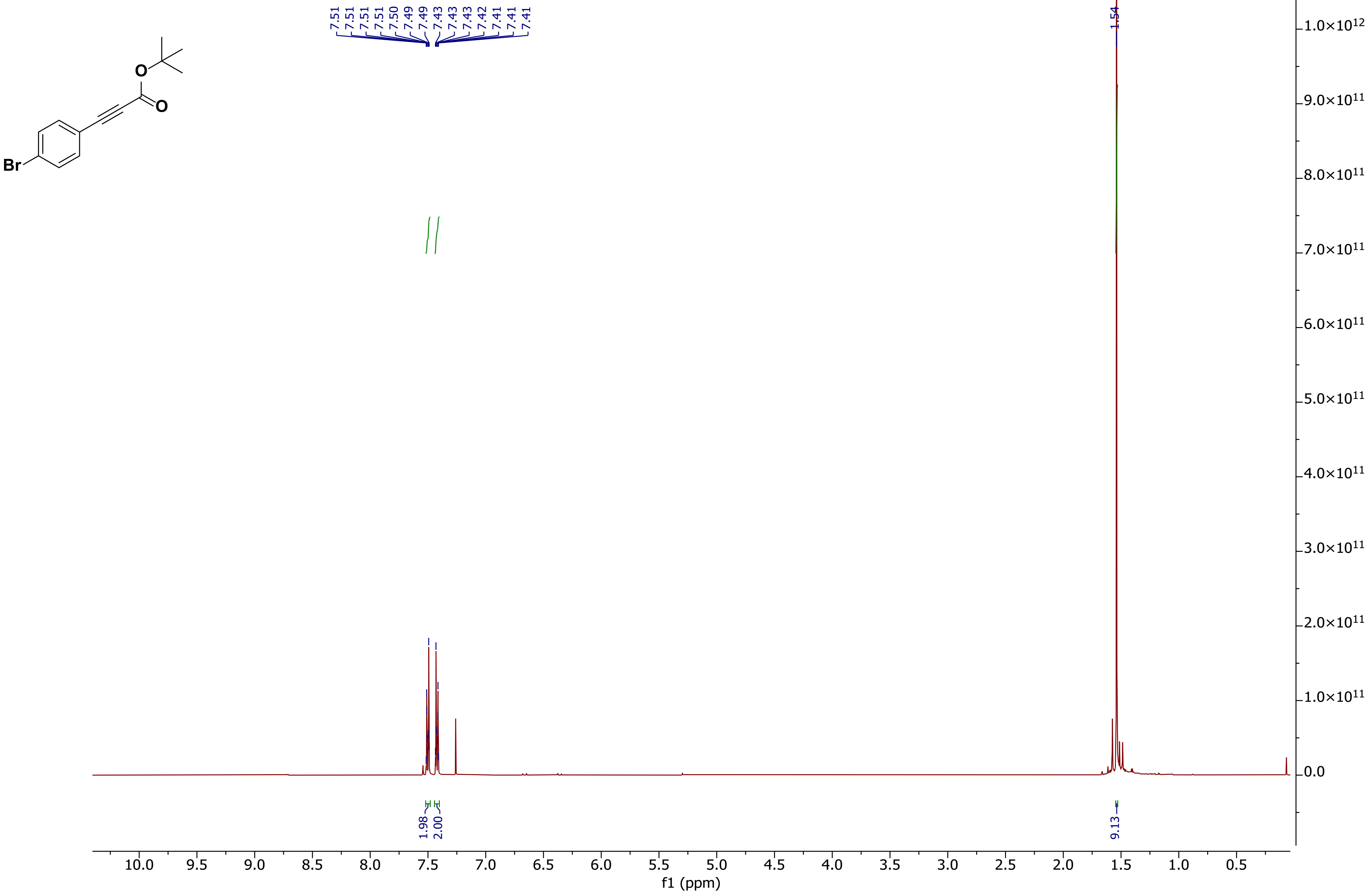
Br
O
O
7.51
7.51
7.51
7.51
7.50
7.49
7.49
7.43
7.43
7.43
7.42
7.41
7.41
7.41
1.54
1.98
2.00
9.13
1.0×10^12
9.0×10^11
8.0×10^11
7.0×10^11
6.0×10^11
5.0×10^11
4.0×10^11
3.0×10^11
2.0×10^11
1.0×10^11
0.0
10.0
9.5
9.0
8.5
8.0
7.5
7.0
6.5
6.0
5.5
5.0
4.5
4.0
3.5
3.0
2.5
2.0
1.5
1.0
0.5
f1 (ppm)

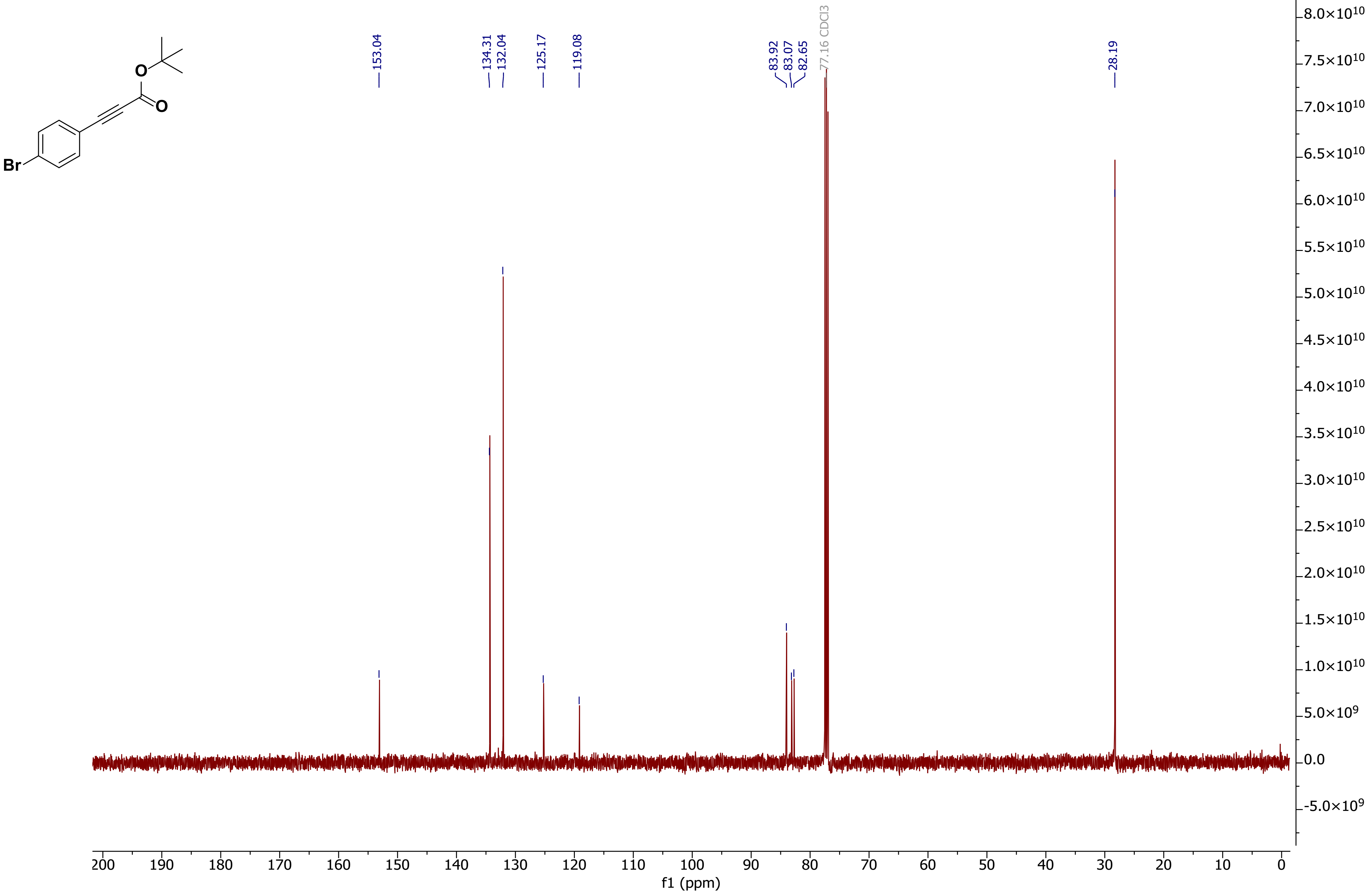

Br
O
O
153.04
134.31
132.04
125.17
119.08
83.92
83.07
82.65
77.16 CDCl3
28.19
f1 (ppm)

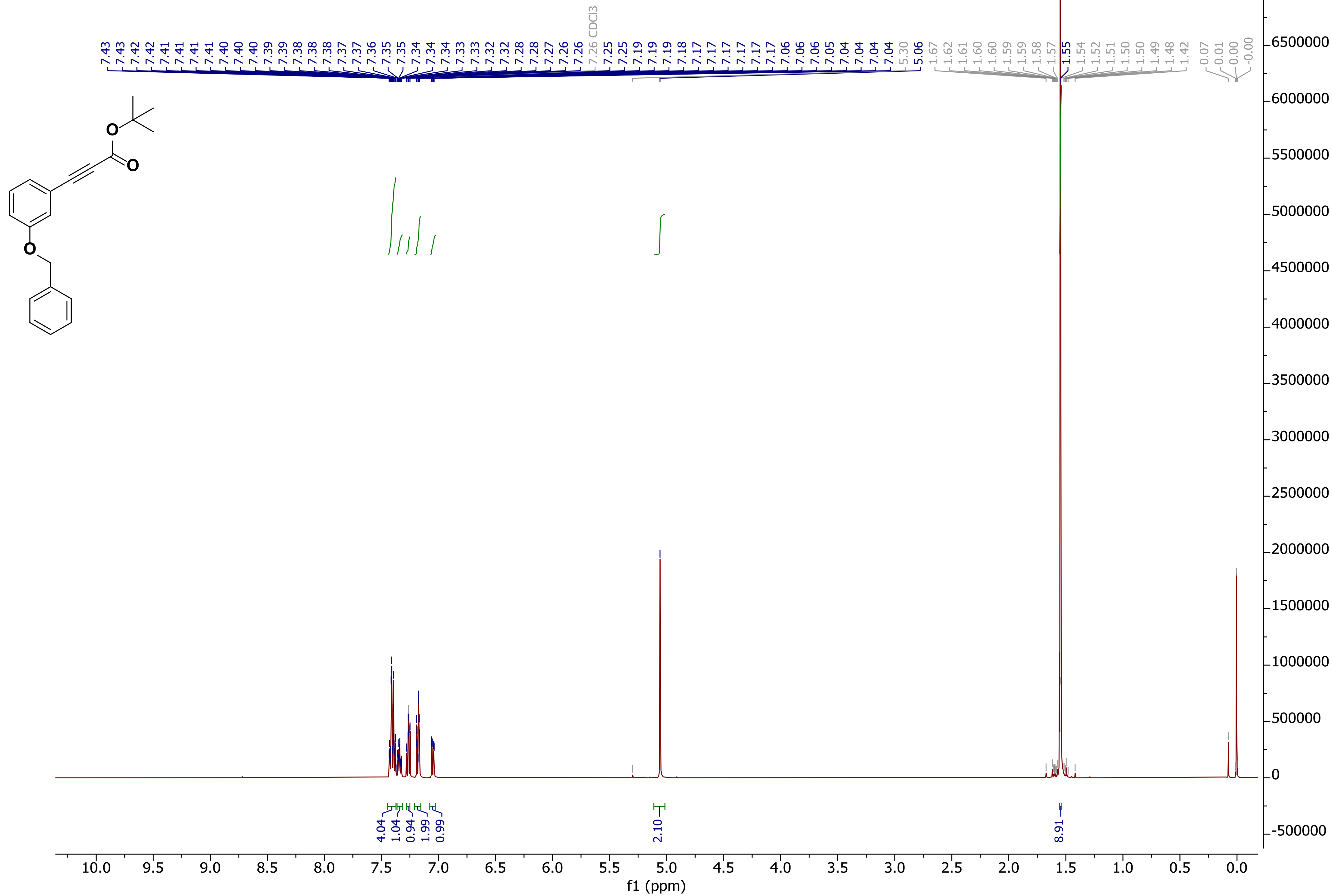
7.43 7.43 7.42 7.42 7.41 7.41 7.41 7.40 7.40 7.40 7.39 7.39 7.38 7.38 7.38 7.37 7.37 7.36 7.35 7.35 7.34 7.34 7.34 7.33 7.33 7.32 7.32 7.28 7.28 7.27 7.26 7.26
7.26 CDCl3
7.25 7.25 7.19 7.19 7.19 7.18 7.17 7.17 7.17 7.17 7.17 7.06 7.06 7.05 7.04 7.04 7.04 7.04 5.30 5.06 1.67 1.62 1.61 1.60 1.60 1.59 1.59 1.58 1.57 1.55 1.54 1.52 1.51 1.50 1.50 1.49 1.48 1.42 0.07 0.01 0.00 -0.00
O
O
O
4.04 1.04 0.94 1.99 0.99 2.10 8.91
f1 (ppm)

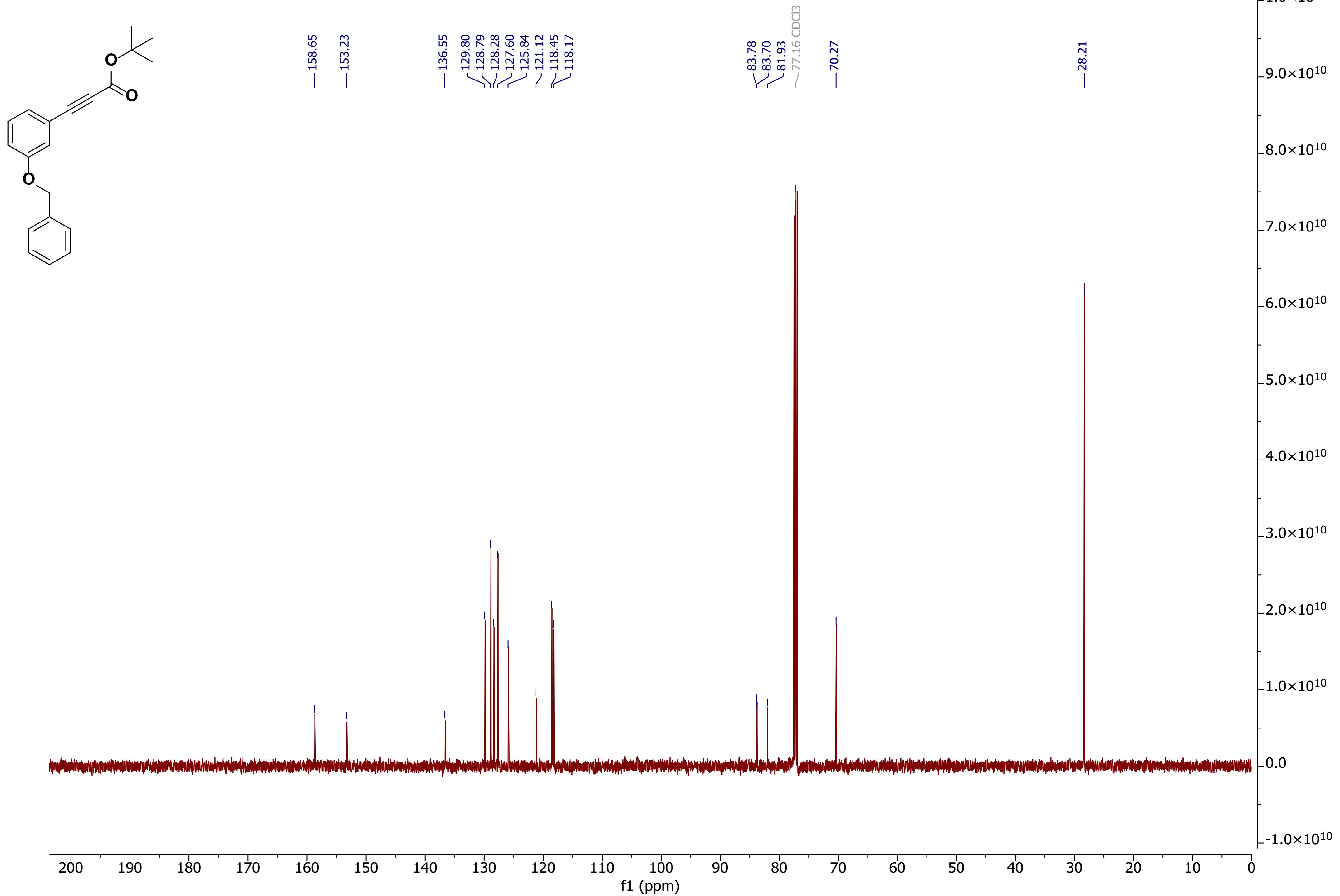

158.65
153.23
136.55
129.80
128.79
128.28
127.60
125.84
121.12
118.45
118.17
83.78
83.70
81.93
77.16 CDCl3
70.27
28.21
9.0×10^10
8.0×10^10
7.0×10^10
6.0×10^10
5.0×10^10
4.0×10^10
3.0×10^10
2.0×10^10
1.0×10^10
0.0
-1.0×10^10
200 190 180 170 160 150 140 130 120 110 100 90 80 70 60 50 40 30 20 10 0
f1 (ppm)

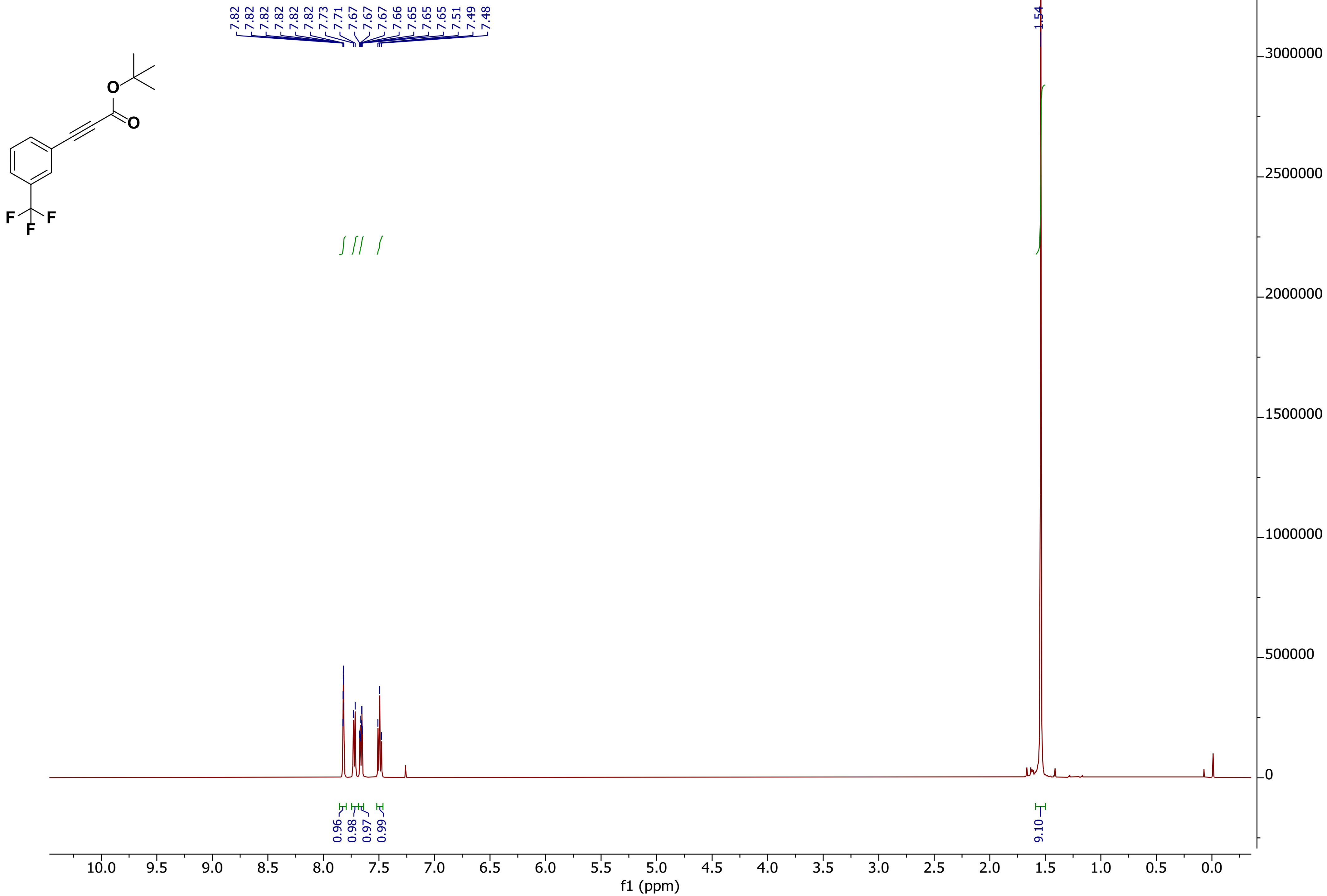

7.82
7.82
7.82
7.82
7.82
7.82
7.73
7.71
7.67
7.67
7.67
7.66
7.65
7.65
7.65
7.51
7.49
7.48
1.54
F
F
F
O
O
3000000
2500000
2000000
1500000
1000000
500000
0
0.96
0.98
0.97
0.99
9.10
10.0
9.5
9.0
8.5
8.0
7.5
7.0
6.5
6.0
5.5
5.0
4.5
4.0
3.5
3.0
2.5
2.0
1.5
1.0
0.5
0.0
f1 (ppm)

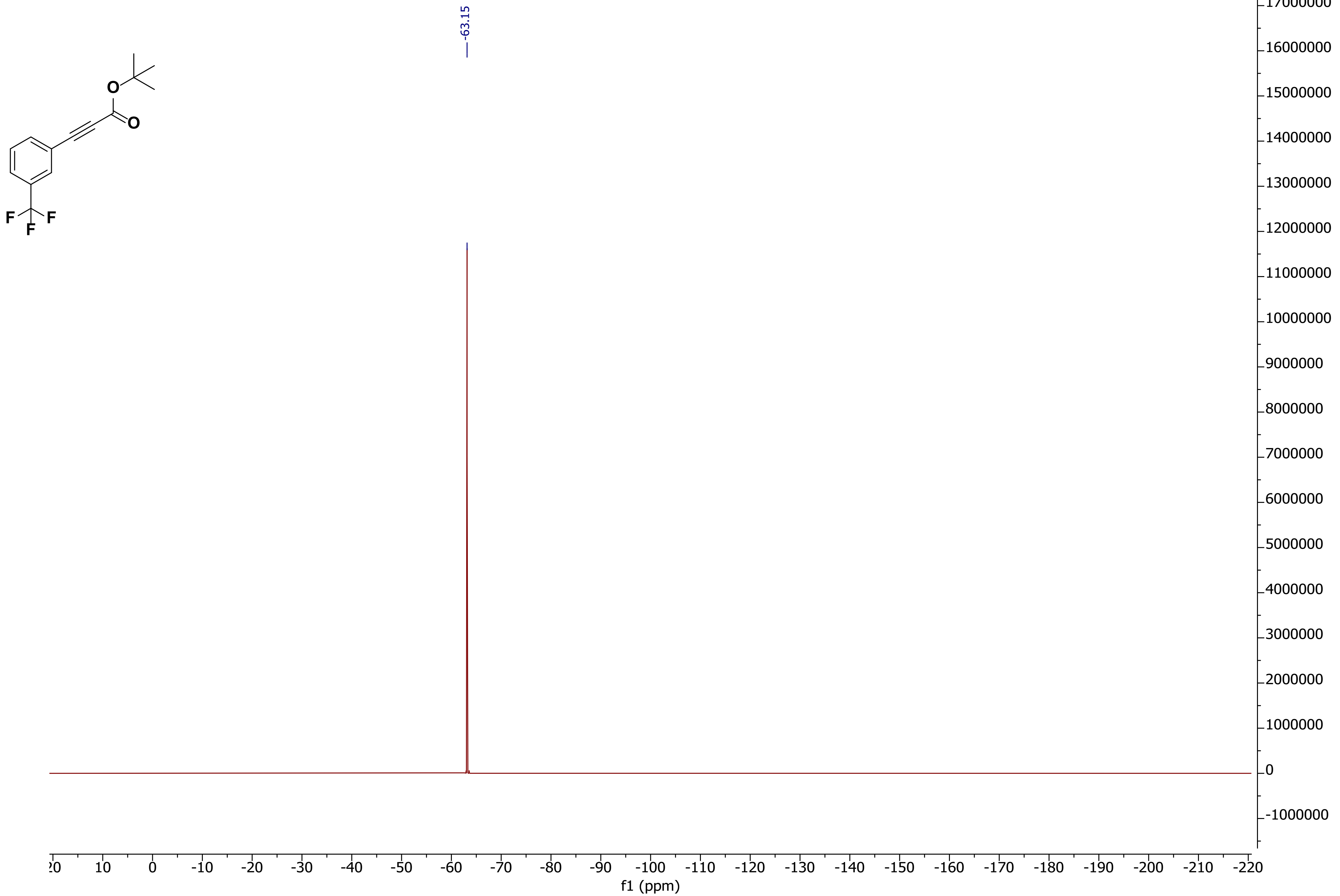

-63.15
F
F
F
O
O
f1 (ppm)

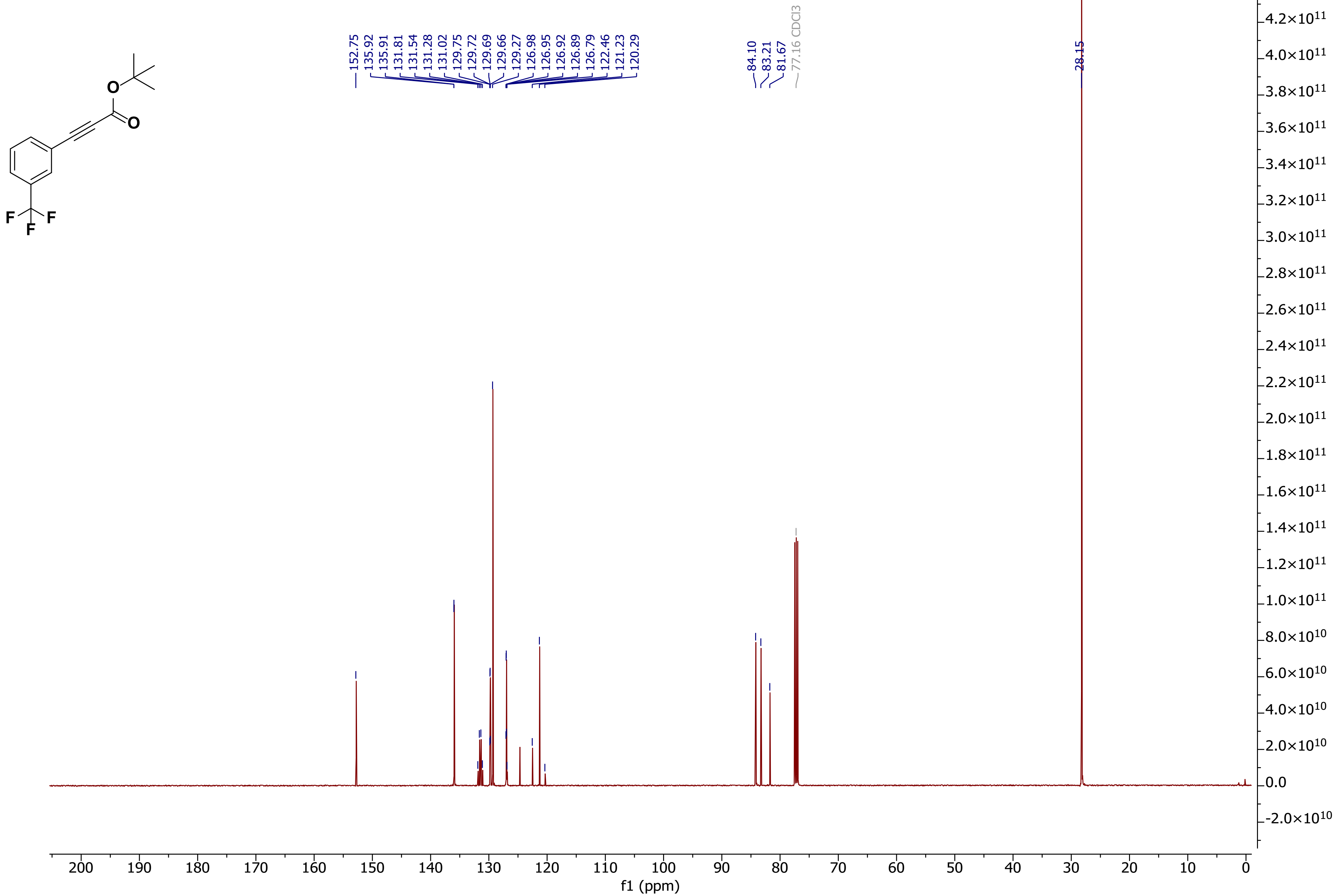

152.75
135.92
135.91
131.81
131.54
131.28
131.02
129.75
129.72
129.69
129.66
129.27
126.98
126.95
126.92
126.89
126.79
122.46
121.23
120.29
84.10
83.21
81.67
77.16 CDCl3
28.15
f1 (ppm)

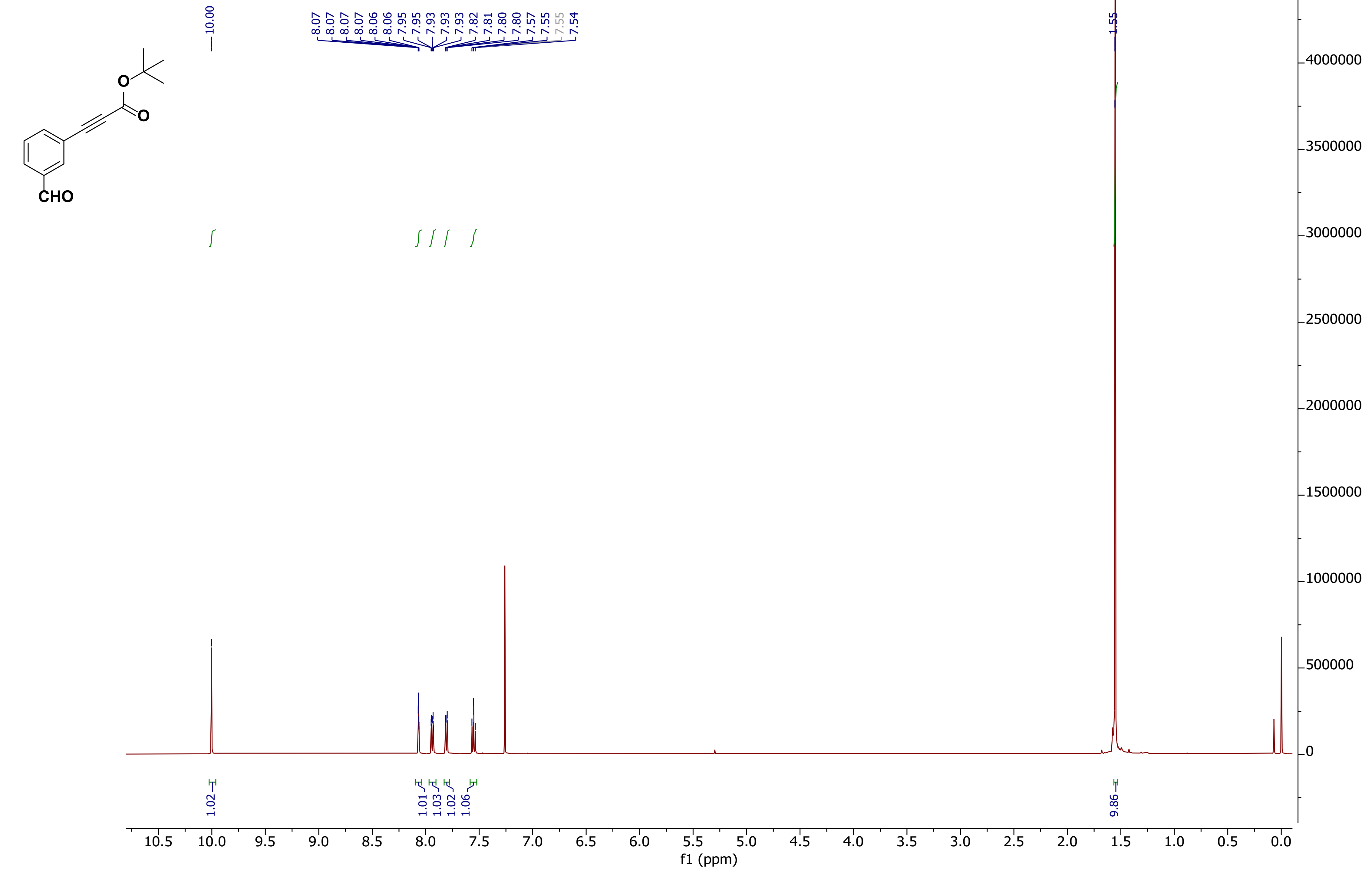

CHO
O
O
10.00
8.07
8.07
8.07
8.07
8.06
8.06
7.95
7.95
7.93
7.93
7.93
7.93
7.82
7.81
7.80
7.80
7.57
7.55
7.55
7.54
1.55
1.02
1.01
1.03
1.02
1.06
9.86
f1 (ppm)

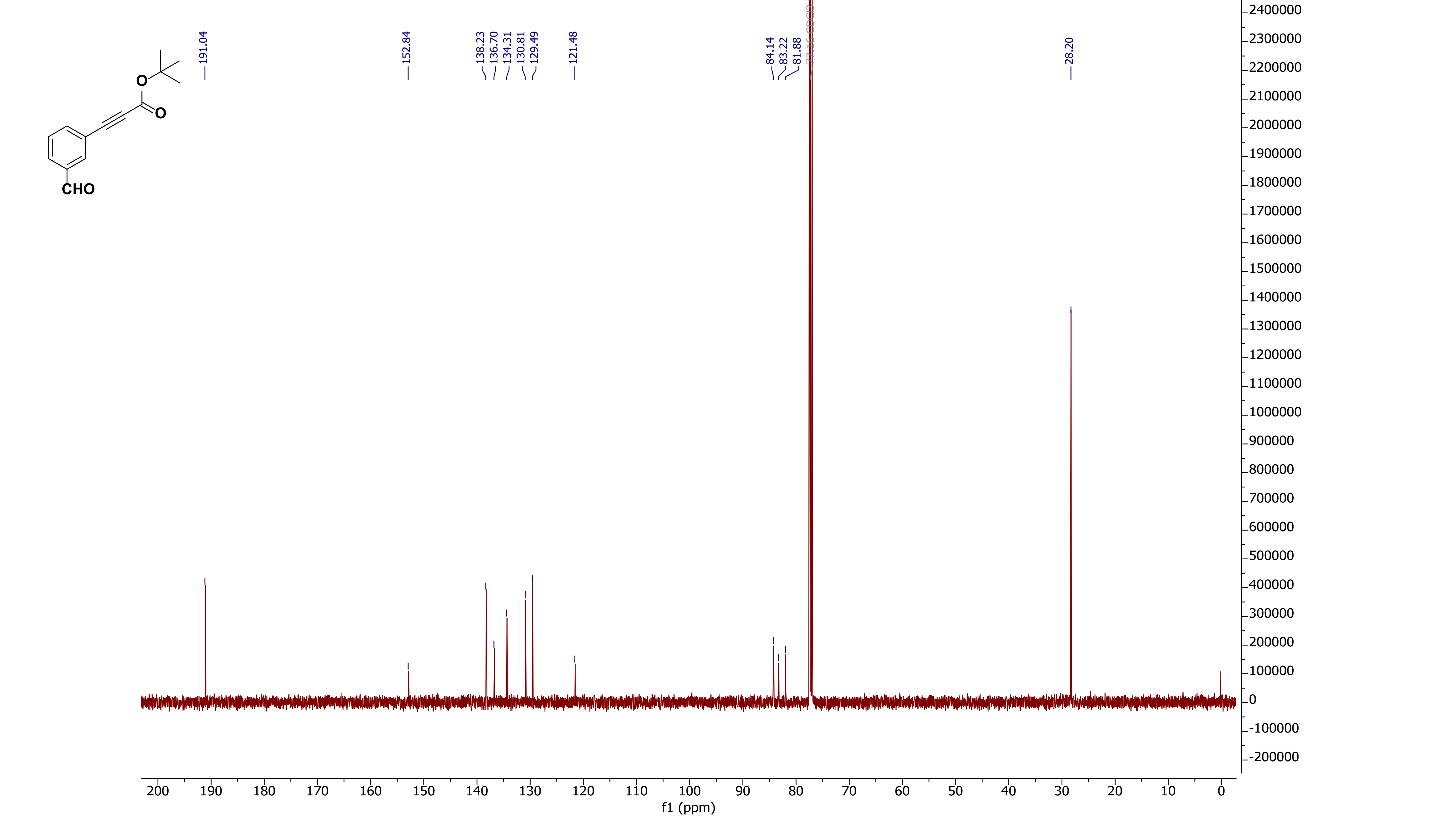
CHO
O
O
191.04
152.84
138.23
136.70
134.31
130.81
129.49
121.48
84.14
83.22
81.88
28.20
f1 (ppm)
200 190 180 170 160 150 140 130 120 110 100 90 80 70 60 50 40 30 20 10 0
2400000 2300000 2200000 2100000 2000000 1900000 1800000 1700000 1600000 1500000 1400000 1300000 1200000 1100000 1000000 900000 800000 700000 600000 500000 400000 300000 200000 100000 0 -100000 -200000

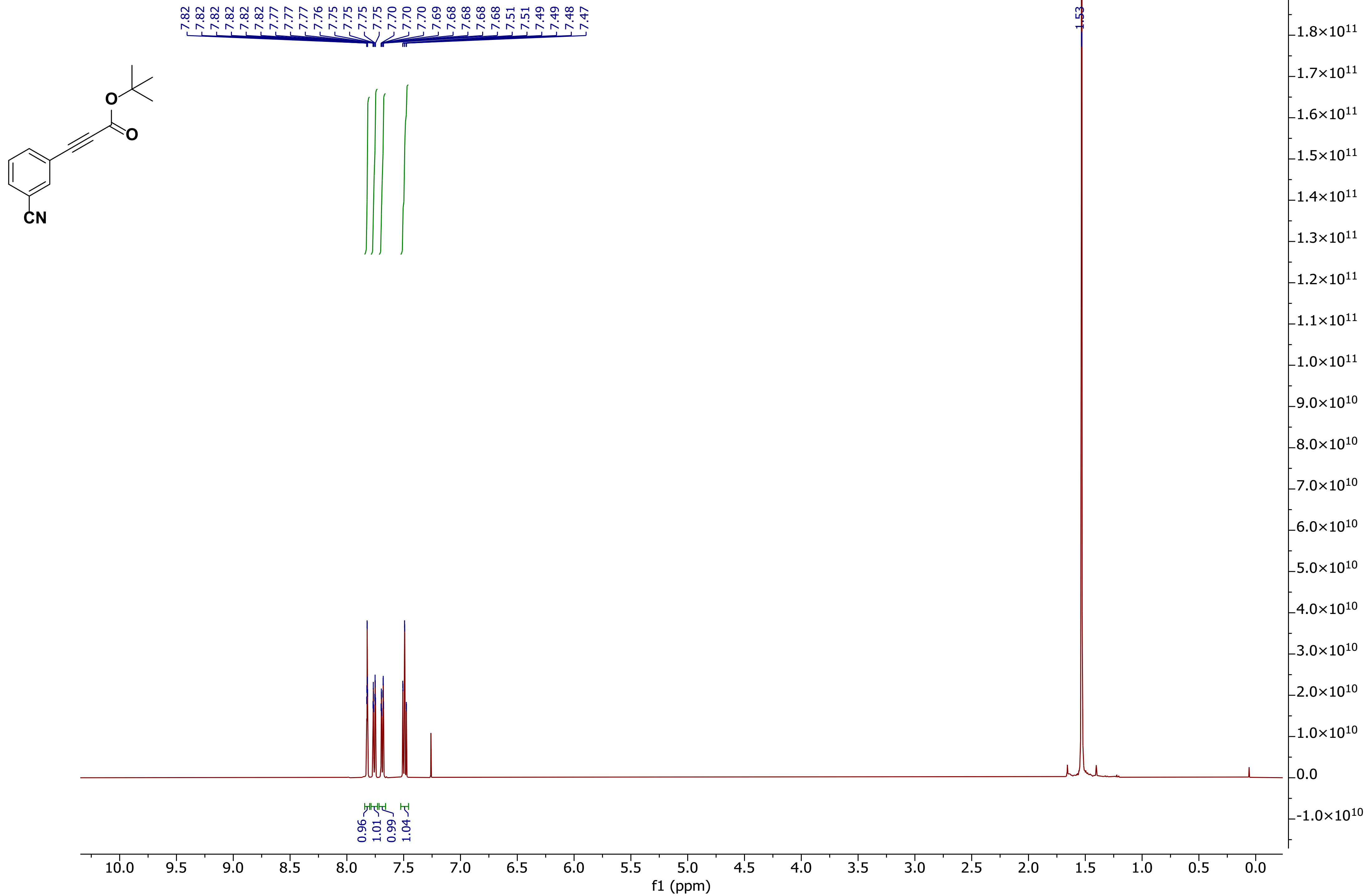
CN
O
O
7.82
7.82
7.82
7.82
7.82
7.82
7.77
7.77
7.77
7.76
7.75
7.75
7.75
7.75
7.75
7.70
7.70
7.70
7.69
7.68
7.68
7.68
7.68
7.51
7.51
7.49
7.49
7.48
7.47
1.53
0.96
1.01
0.99
1.04
f1 (ppm)

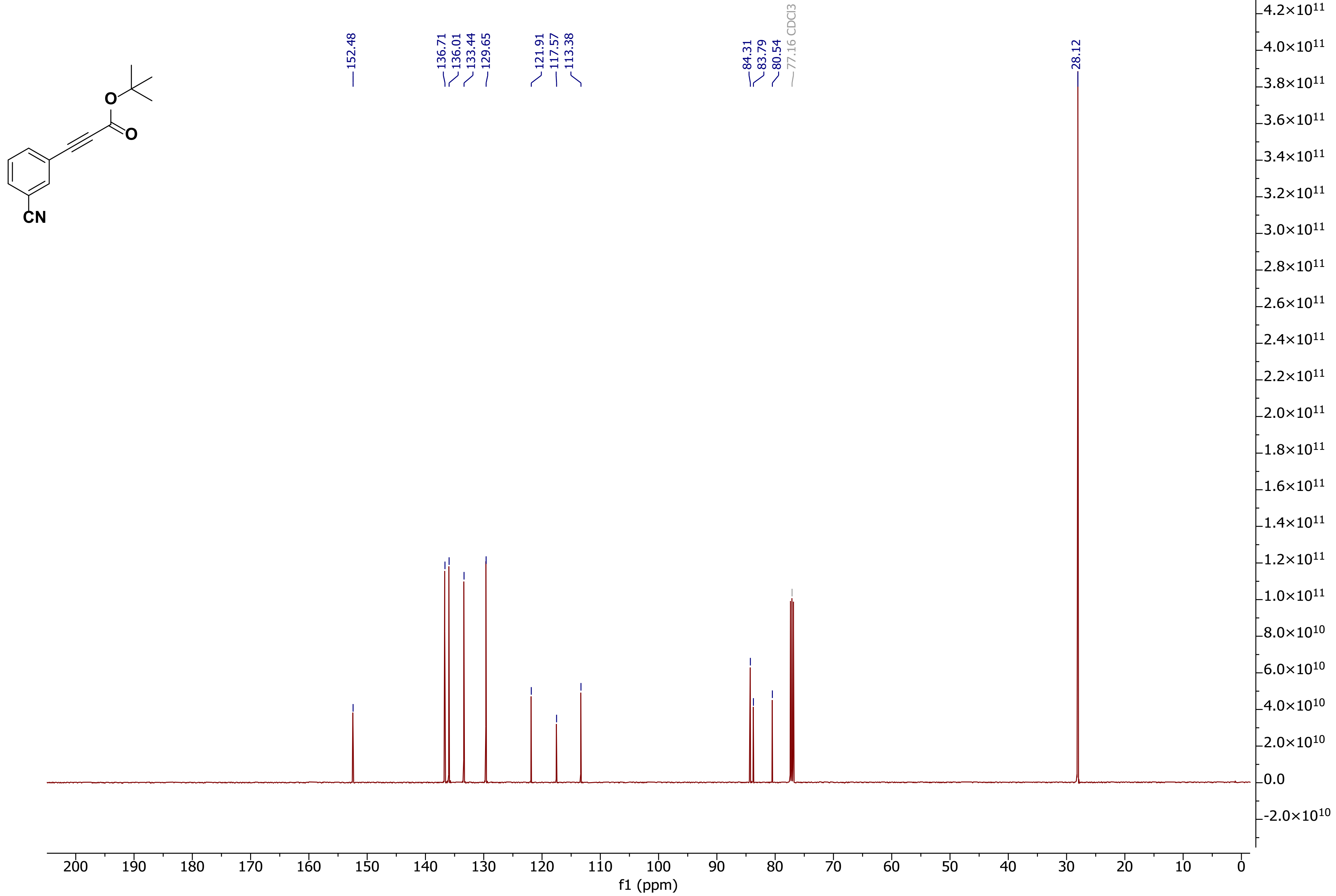

CN
O
O
152.48
136.71
136.01
133.44
129.65
121.91
117.57
113.38
84.31
83.79
80.54
77.16 CDCl3
28.12
f1 (ppm)

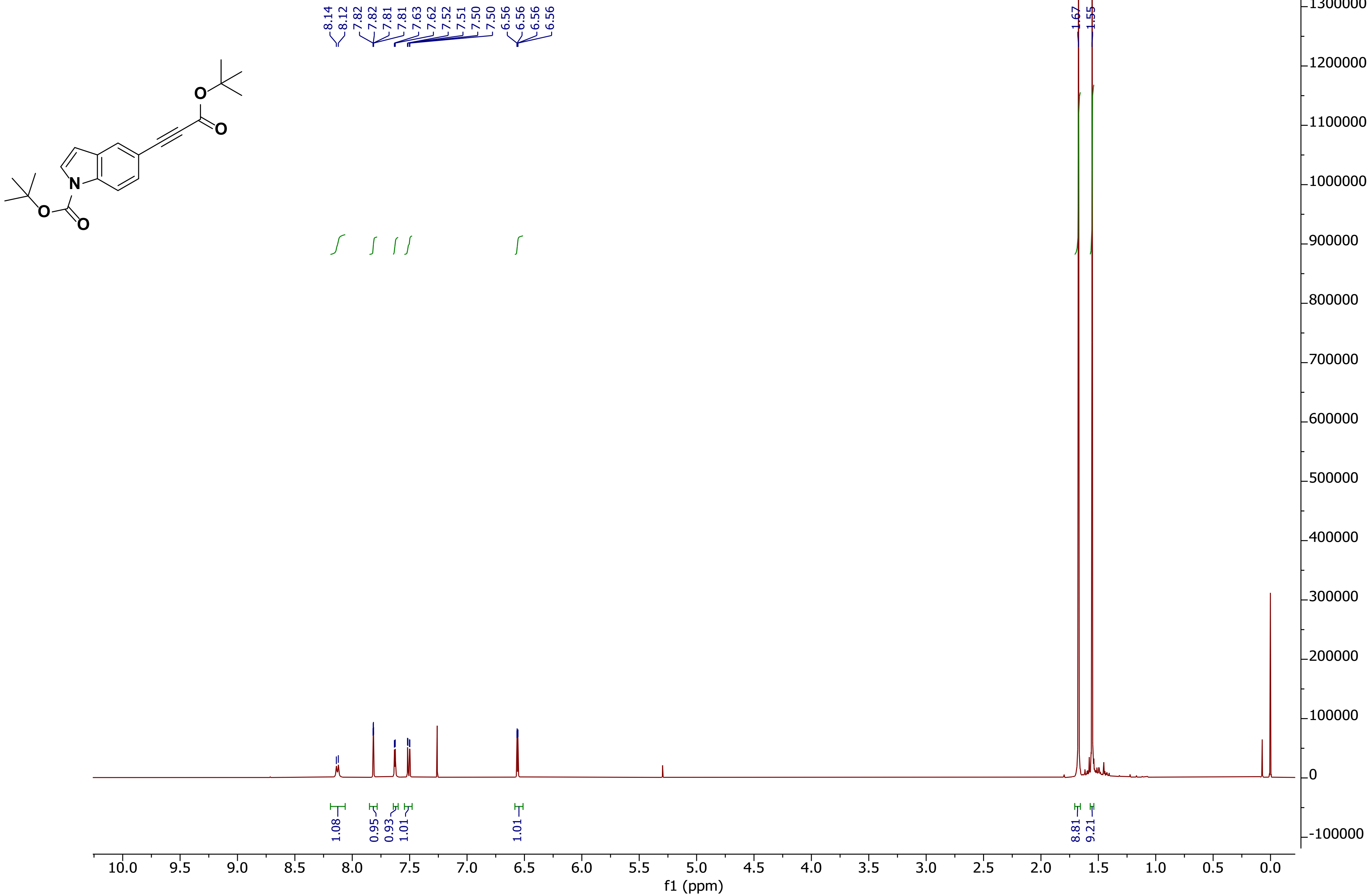

8.14
8.12
7.82
7.82
7.81
7.81
7.63
7.62
7.52
7.51
7.50
7.50
6.56
6.56
6.56
6.56
1.67
1.55
1.08
0.95
0.93
1.01
1.01
8.81
9.21
f1 (ppm)

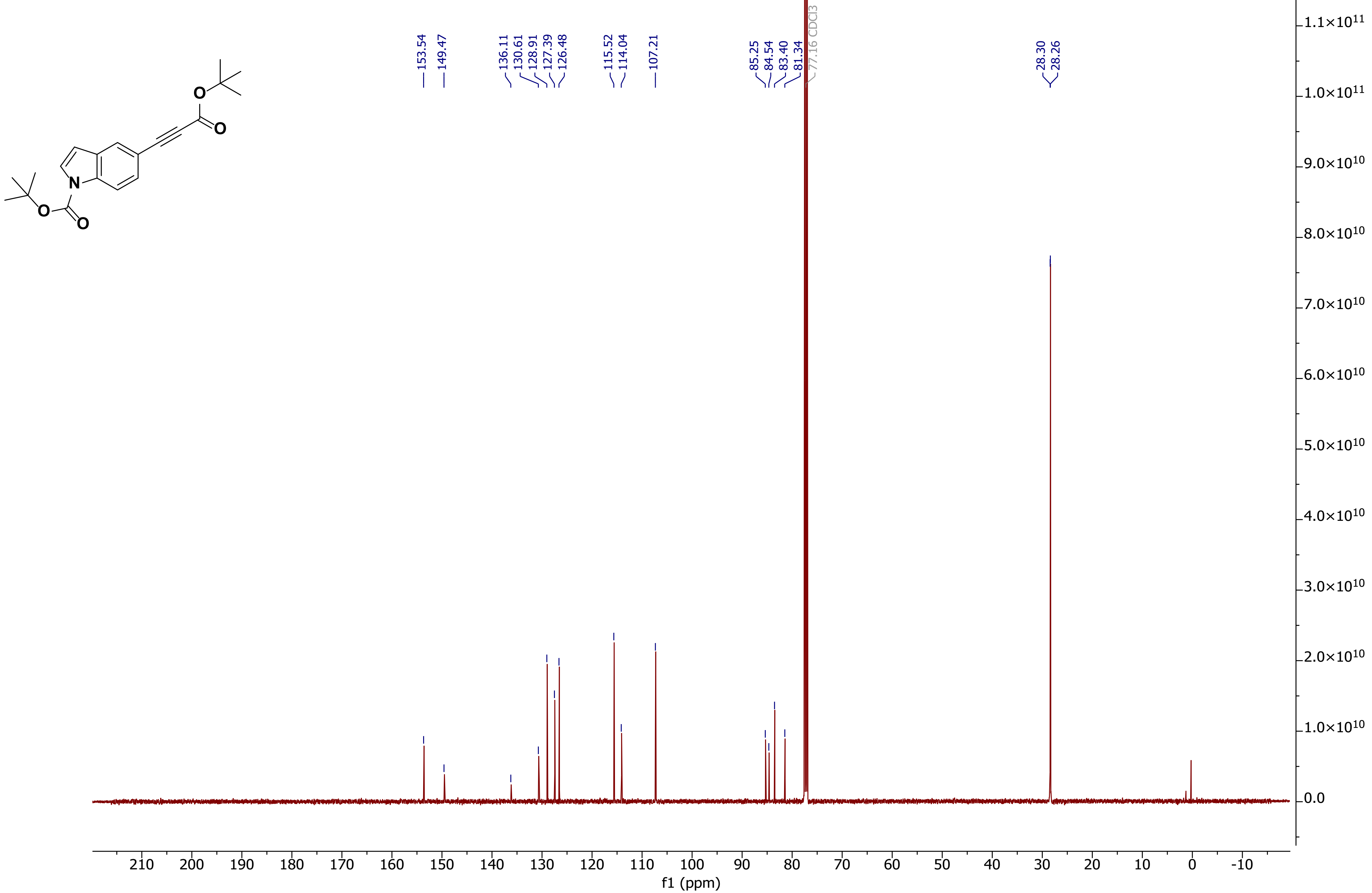
153.54
149.47
136.11
130.61
128.91
127.39
126.48
115.52
114.04
107.21
85.25
84.54
83.40
81.34
77.16 CDCl3
28.30
28.26
f1 (ppm)

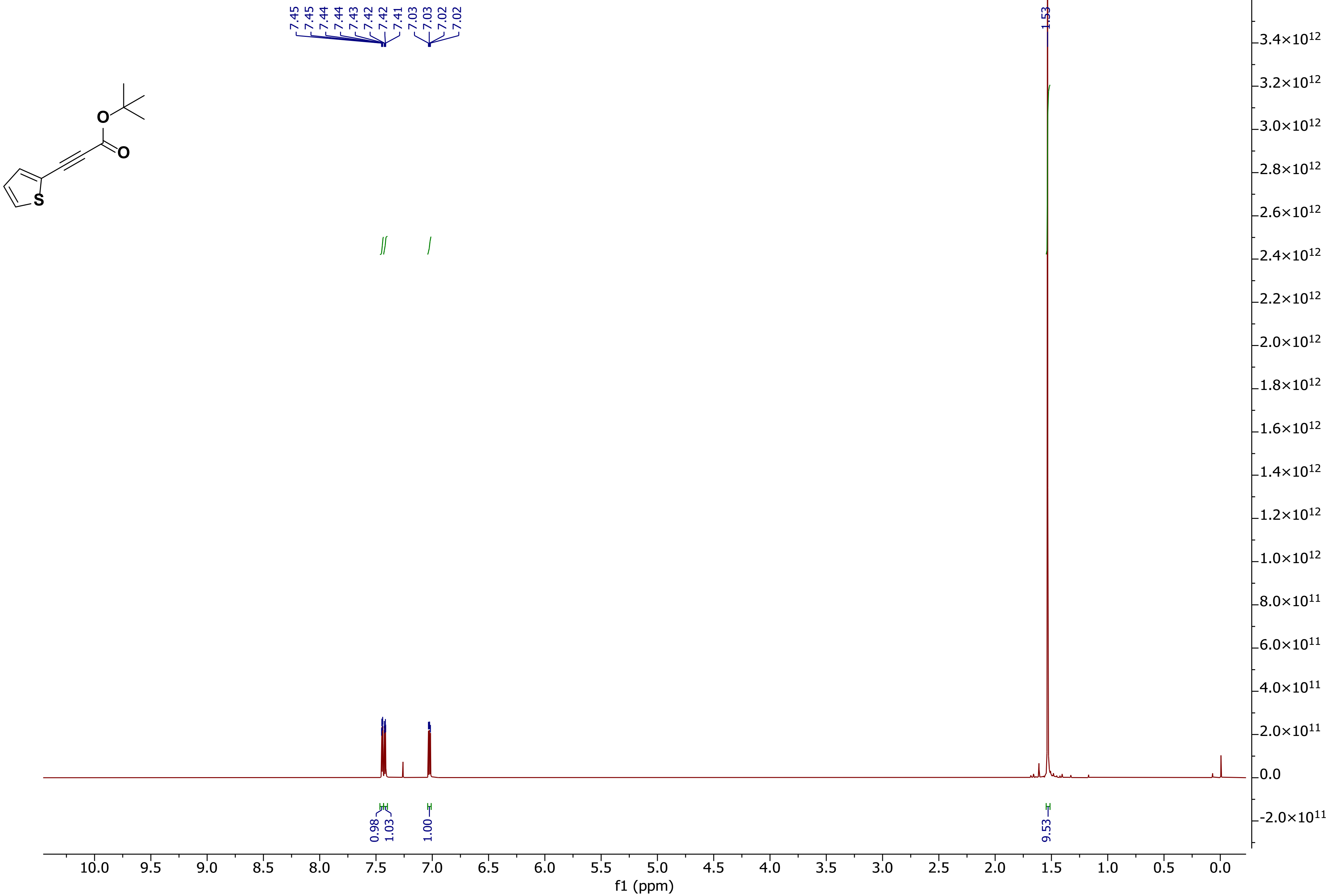

7.45
7.45
7.44
7.44
7.43
7.42
7.42
7.41
7.03
7.03
7.02
7.02
1.53
O
O
S
0.98
1.03
1.00
9.53
3.4×10^12
3.2×10^12
3.0×10^12
2.8×10^12
2.6×10^12
2.4×10^12
2.2×10^12
2.0×10^12
1.8×10^12
1.6×10^12
1.4×10^12
1.2×10^12
1.0×10^12
8.0×10^11
6.0×10^11
4.0×10^11
2.0×10^11
0.0
-2.0×10^11
10.0
9.5
9.0
8.5
8.0
7.5
7.0
6.5
6.0
5.5
5.0
4.5
4.0
3.5
3.0
2.5
2.0
1.5
1.0
0.5
0.0
f1 (ppm)

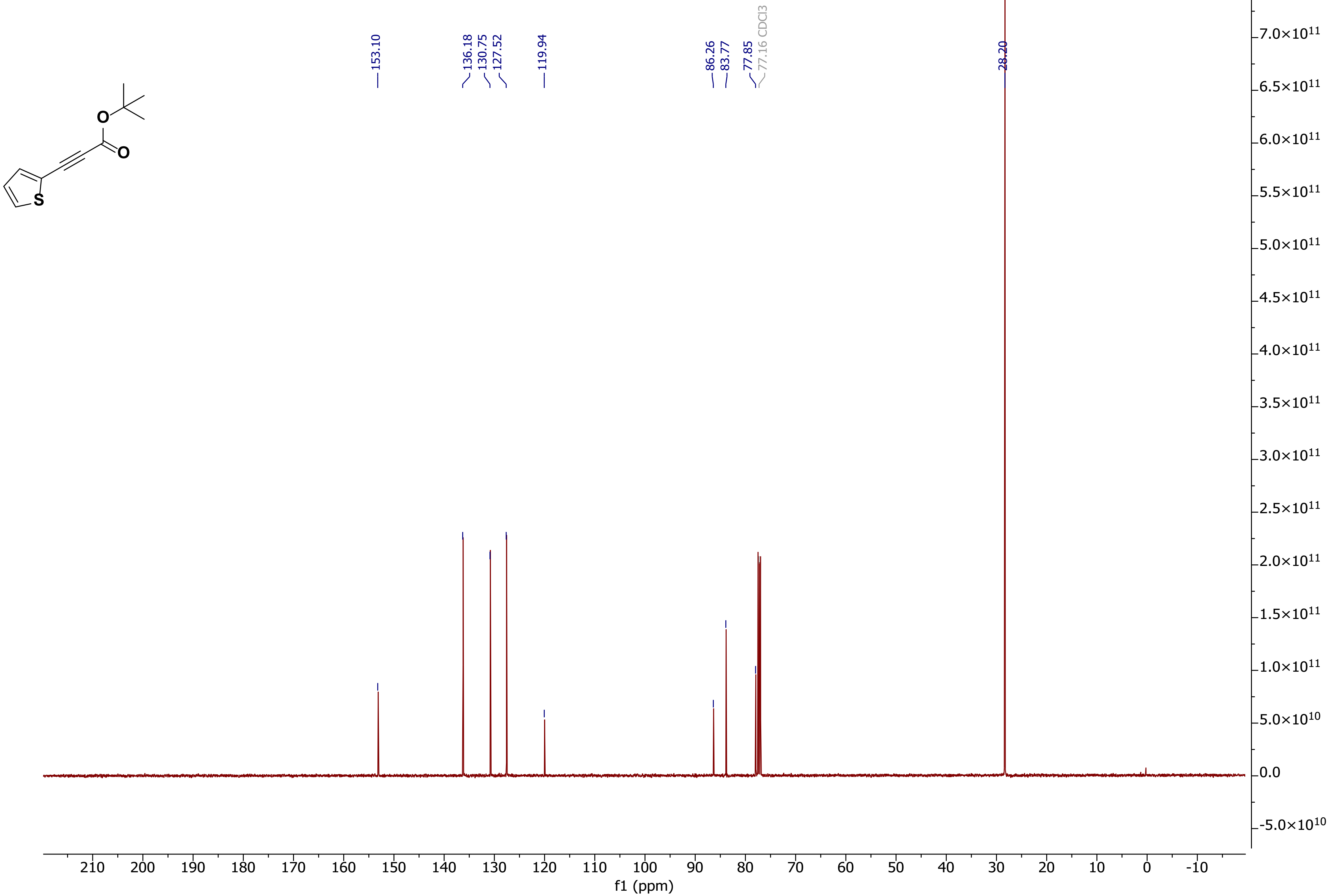
153.10
136.18
130.75
127.52
119.94
86.26
83.77
77.85
77.16 CDCl3
28.20
O
O
S
7.0×10^11
6.5×10^11
6.0×10^11
5.5×10^11
5.0×10^11
4.5×10^11
4.0×10^11
3.5×10^11
3.0×10^11
2.5×10^11
2.0×10^11
1.5×10^11
1.0×10^11
5.0×10^10
0.0
-5.0×10^10
210 200 190 180 170 160 150 140 130 120 110 100 90 80 70 60 50 40 30 20 10 0 -10
f1 (ppm)

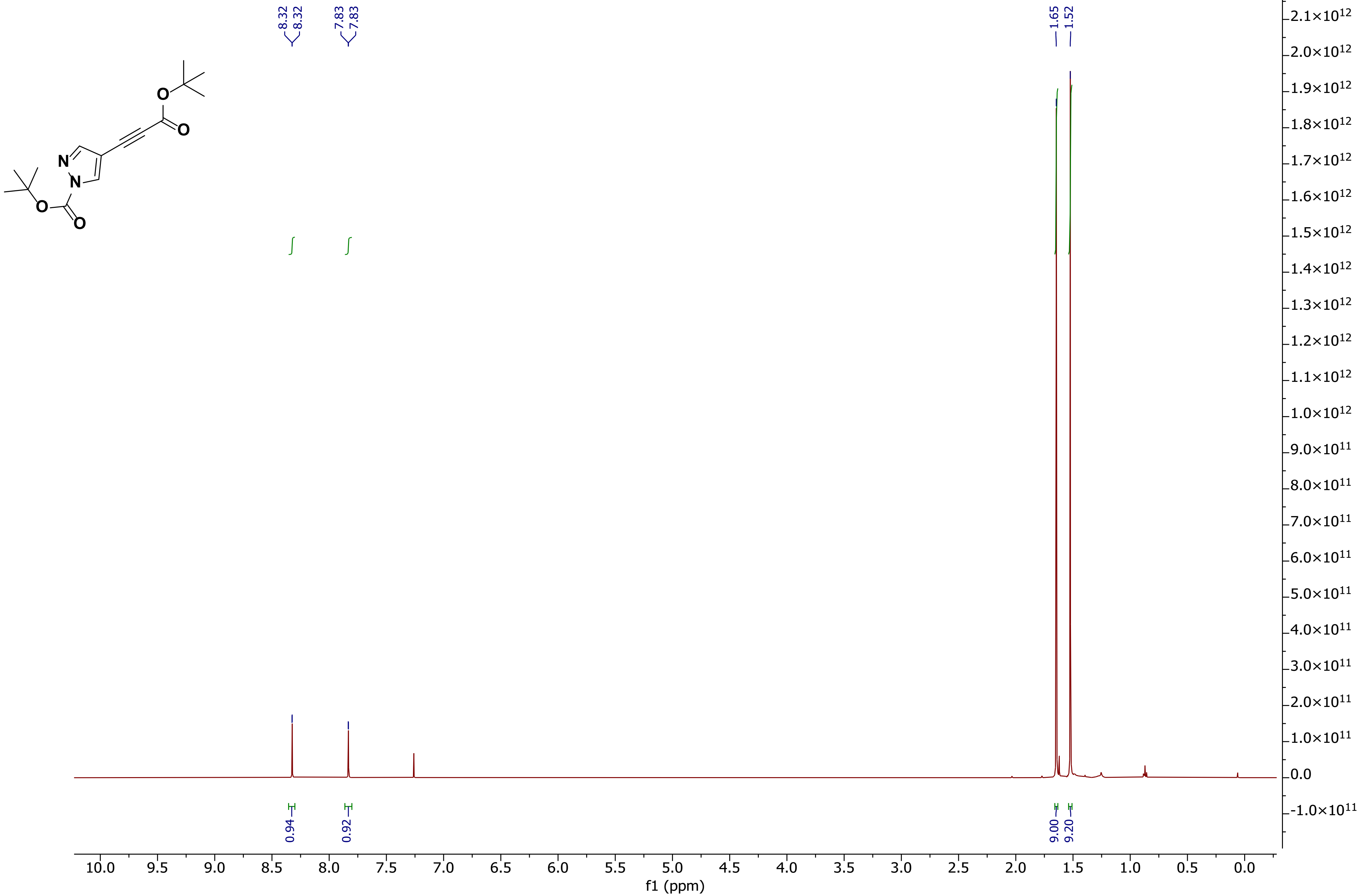

8.32
8.32
7.83
7.83
1.65
1.52
0.94
0.92
9.00
9.20
10.0 9.5 9.0 8.5 8.0 7.5 7.0 6.5 6.0 5.5 5.0 4.5 4.0 3.5 3.0 2.5 2.0 1.5 1.0 0.5 0.0
f1 (ppm)

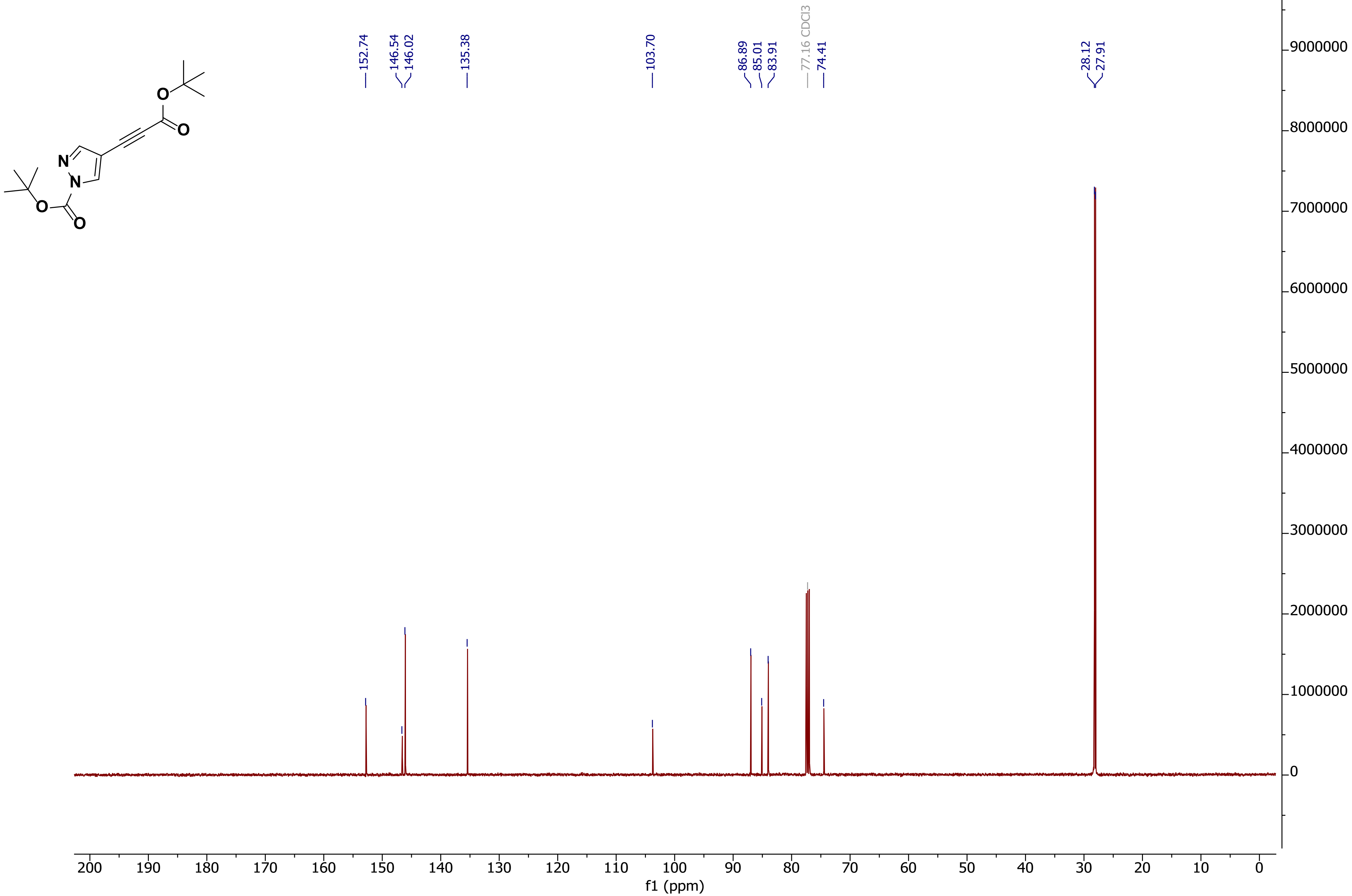

152.74
146.54
146.02
135.38
103.70
86.89
85.01
83.91
77.16 CDCl3
74.41
28.12
27.91
9000000
8000000
7000000
6000000
5000000
4000000
3000000
2000000
1000000
0
200 190 180 170 160 150 140 130 120 110 100 90 80 70 60 50 40 30 20 10 0
f1 (ppm)
O
O
N
N
O
O

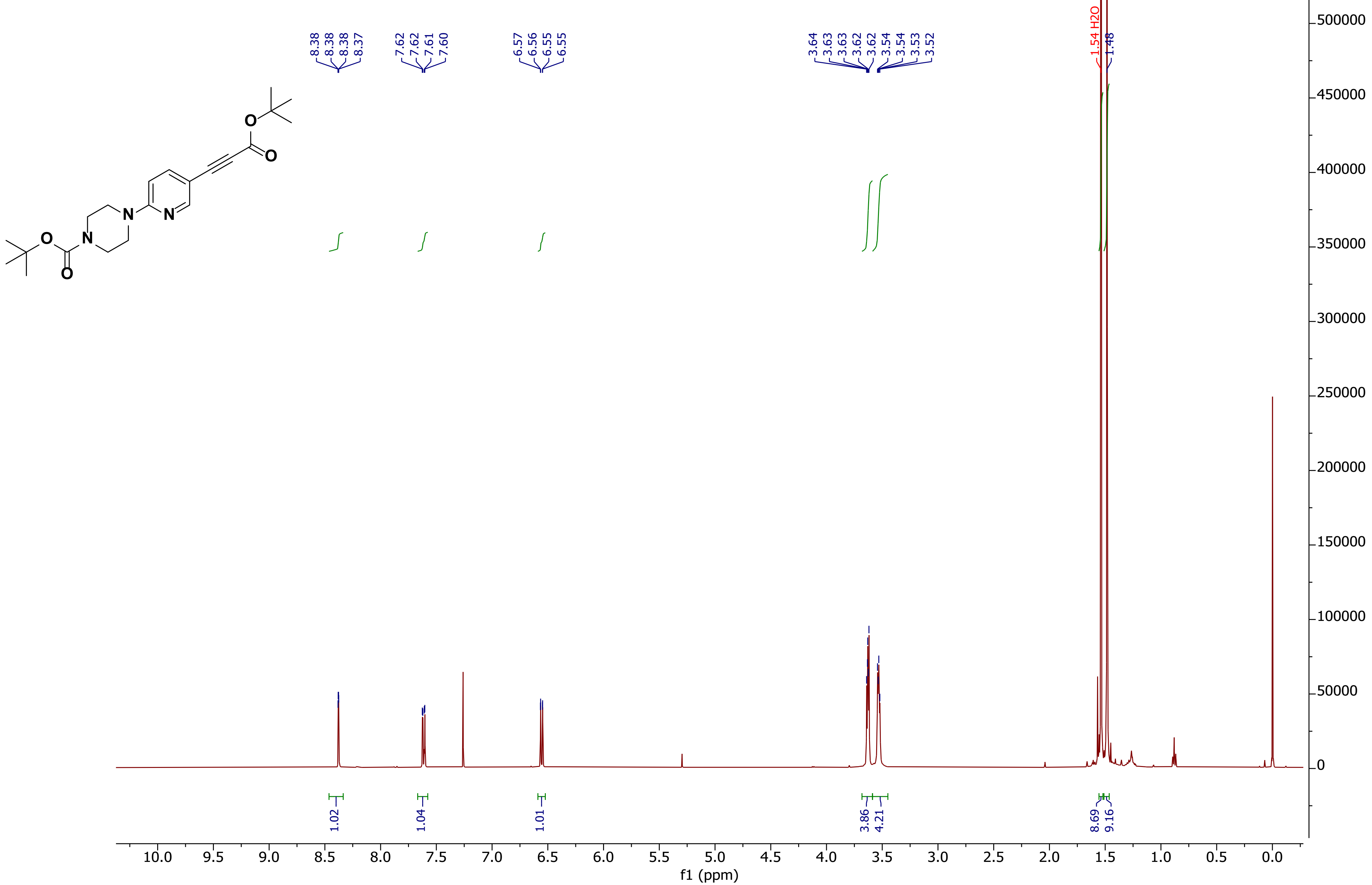

8.38 8.38 8.38 8.37
7.62 7.62 7.61 7.60
6.57 6.56 6.55 6.55
3.64 3.63 3.63 3.62 3.62 3.54 3.54 3.53 3.52
1.54 H2O
1.48
1.02
1.04
1.01
3.86
4.21
8.69
9.16
500000
450000
400000
350000
300000
250000
200000
150000
100000
50000
0
10.0 9.5 9.0 8.5 8.0 7.5 7.0 6.5 6.0 5.5 5.0 4.5 4.0 3.5 3.0 2.5 2.0 1.5 1.0 0.5 0.0
f1 (ppm)

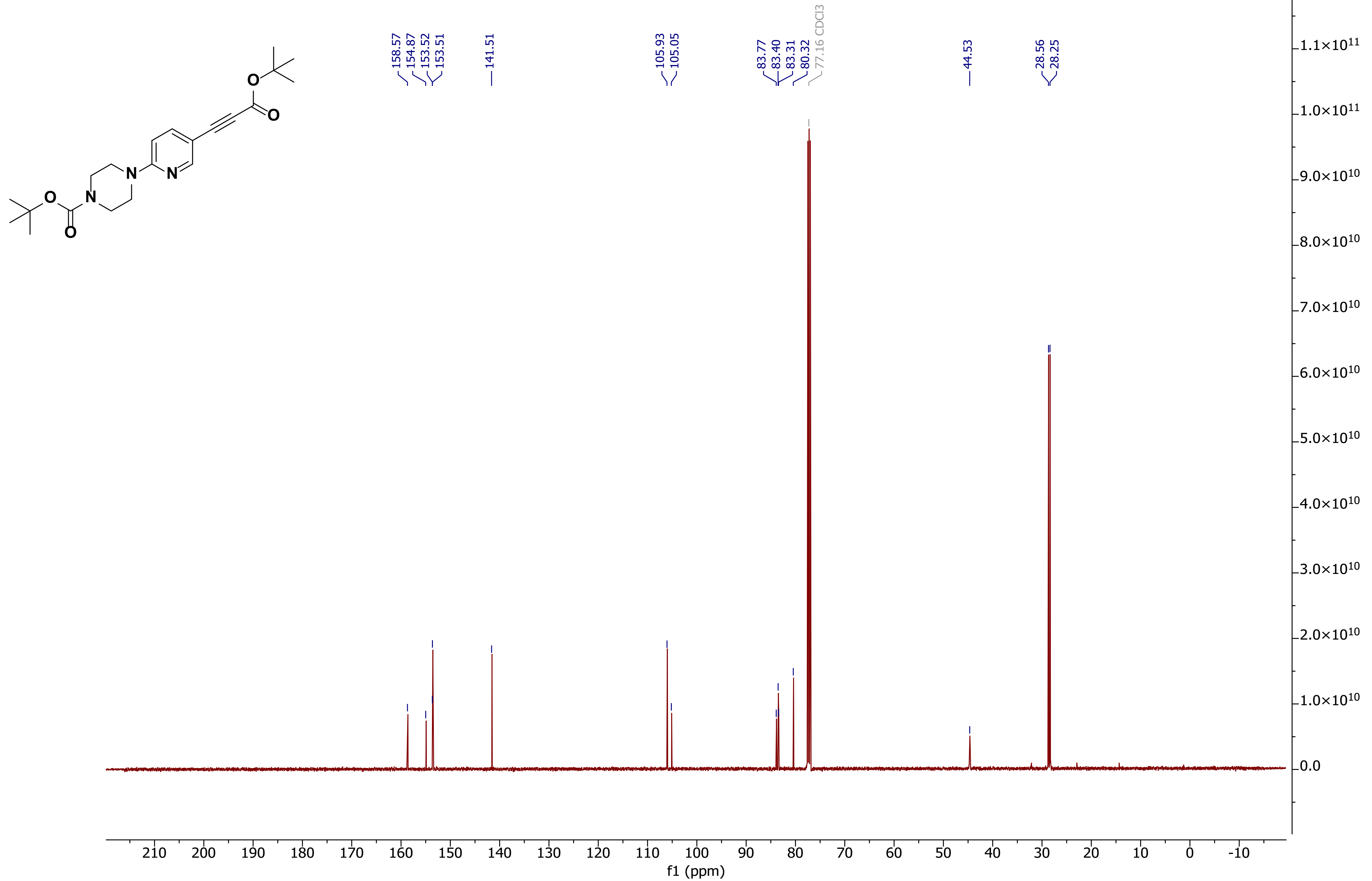

158.57
154.87
153.52
153.51
141.51
105.93
105.05
83.77
83.40
83.31
80.32
77.16 CDCl3
44.53
28.56
28.25
f1 (ppm)

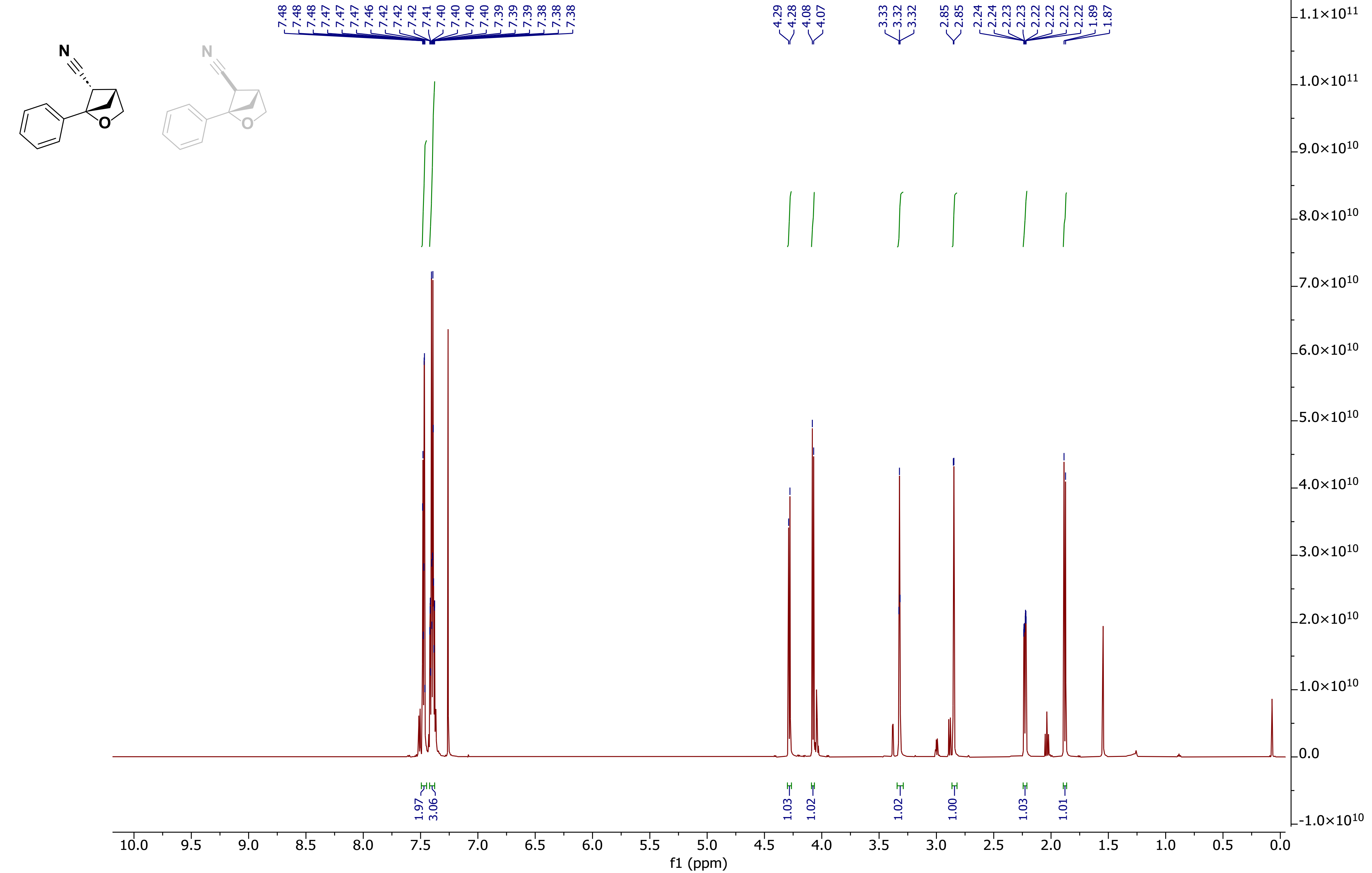

N
O
7.48
7.48
7.48
7.47
7.47
7.47
7.46
7.42
7.42
7.42
7.41
7.40
7.40
7.40
7.39
7.39
7.39
7.38
7.38
7.38
4.29
4.28
4.08
4.07
3.33
3.32
3.32
2.85
2.85
2.24
2.24
2.23
2.23
2.22
2.22
2.22
2.22
1.89
1.87
1.97
3.06
1.03
1.02
1.02
1.00
1.03
1.01
f1 (ppm)

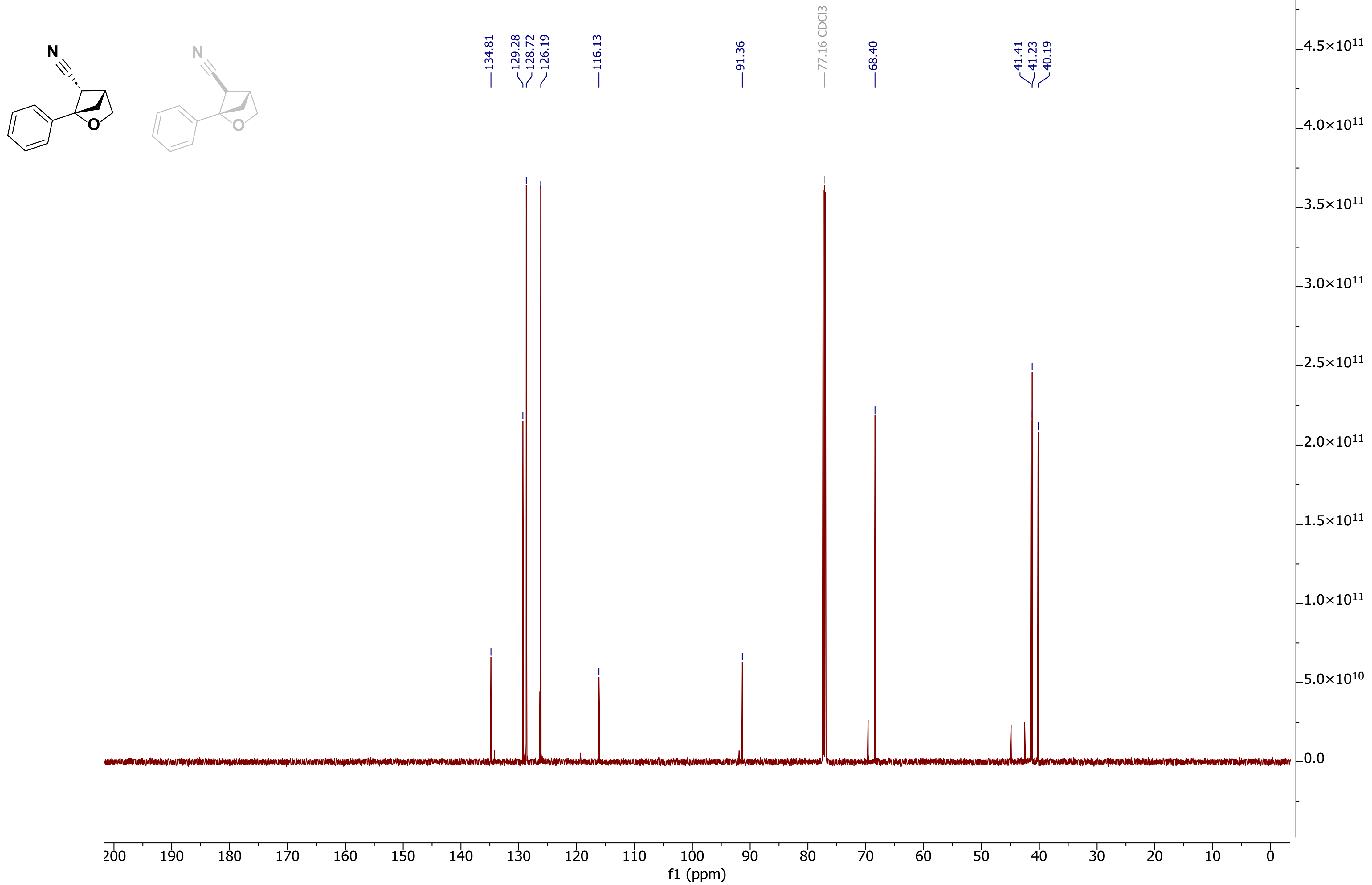
N
O
N
O
134.81
129.28
128.72
126.19
116.13
91.36
77.16 CDCl3
68.40
41.41
41.23
40.19
4.5×10^11
4.0×10^11
3.5×10^11
3.0×10^11
2.5×10^11
2.0×10^11
1.5×10^11
1.0×10^11
5.0×10^10
0.0
200
190
180
170
160
150
140
130
120
110
100
90
80
70
60
50
40
30
20
10
0
f1 (ppm)

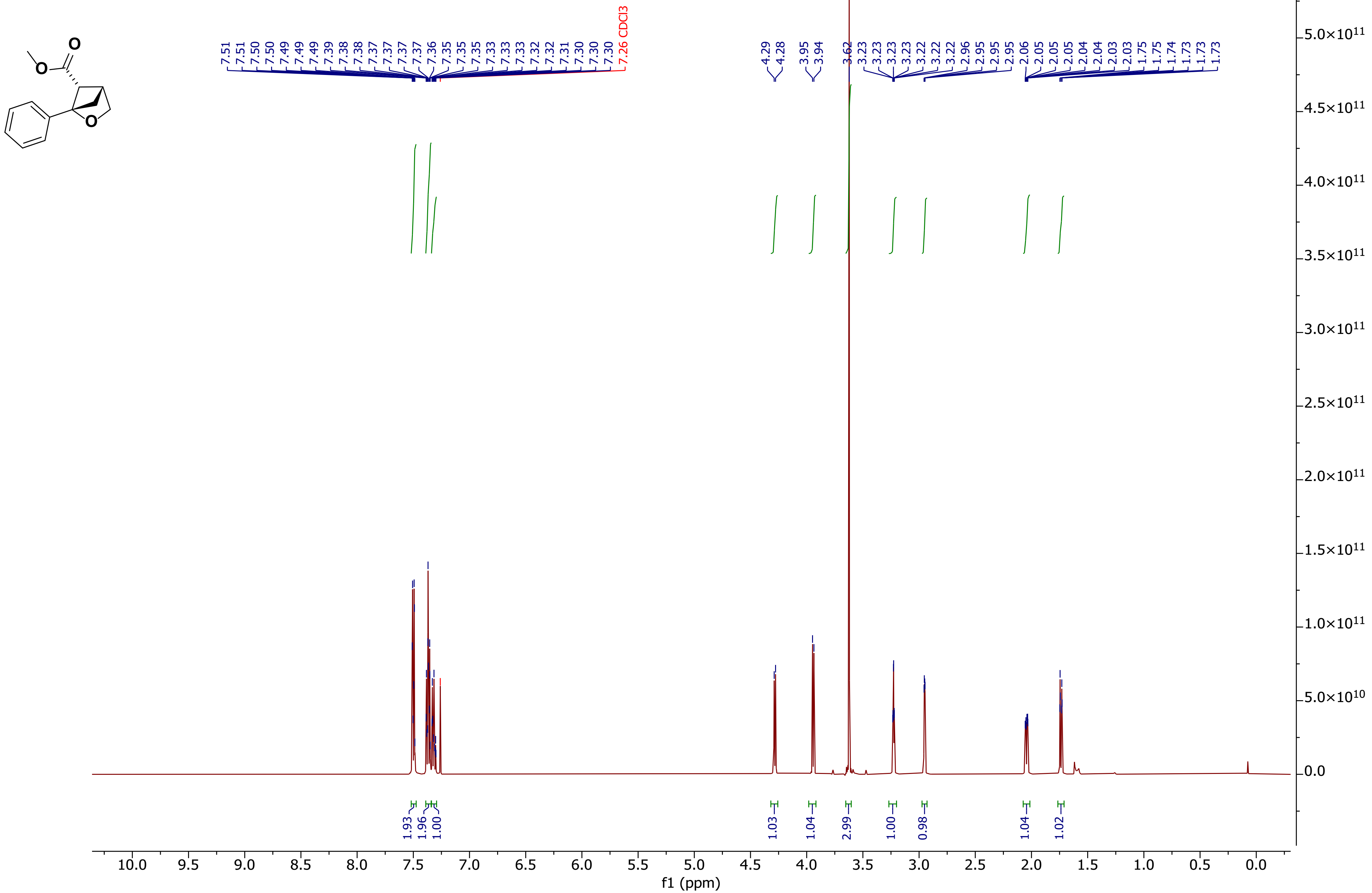
7.51 7.51 7.50 7.50 7.49 7.49 7.49 7.39 7.38 7.38 7.37 7.37 7.37 7.37 7.36 7.35 7.35 7.35 7.33 7.33 7.33 7.32 7.32 7.31 7.30 7.30 7.30
7.26 CDCl3
4.29 4.28 3.95 3.94 3.62 3.23 3.23 3.23 3.23 3.22 3.22 3.22 2.96 2.95 2.95 2.95 2.06 2.05 2.05 2.05 2.04 2.04 2.03 2.03 1.75 1.75 1.74 1.73 1.73 1.73
1.93 1.96 1.00 1.03 1.04 2.99 1.00 0.98 1.04 1.02
f1 (ppm)

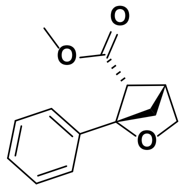

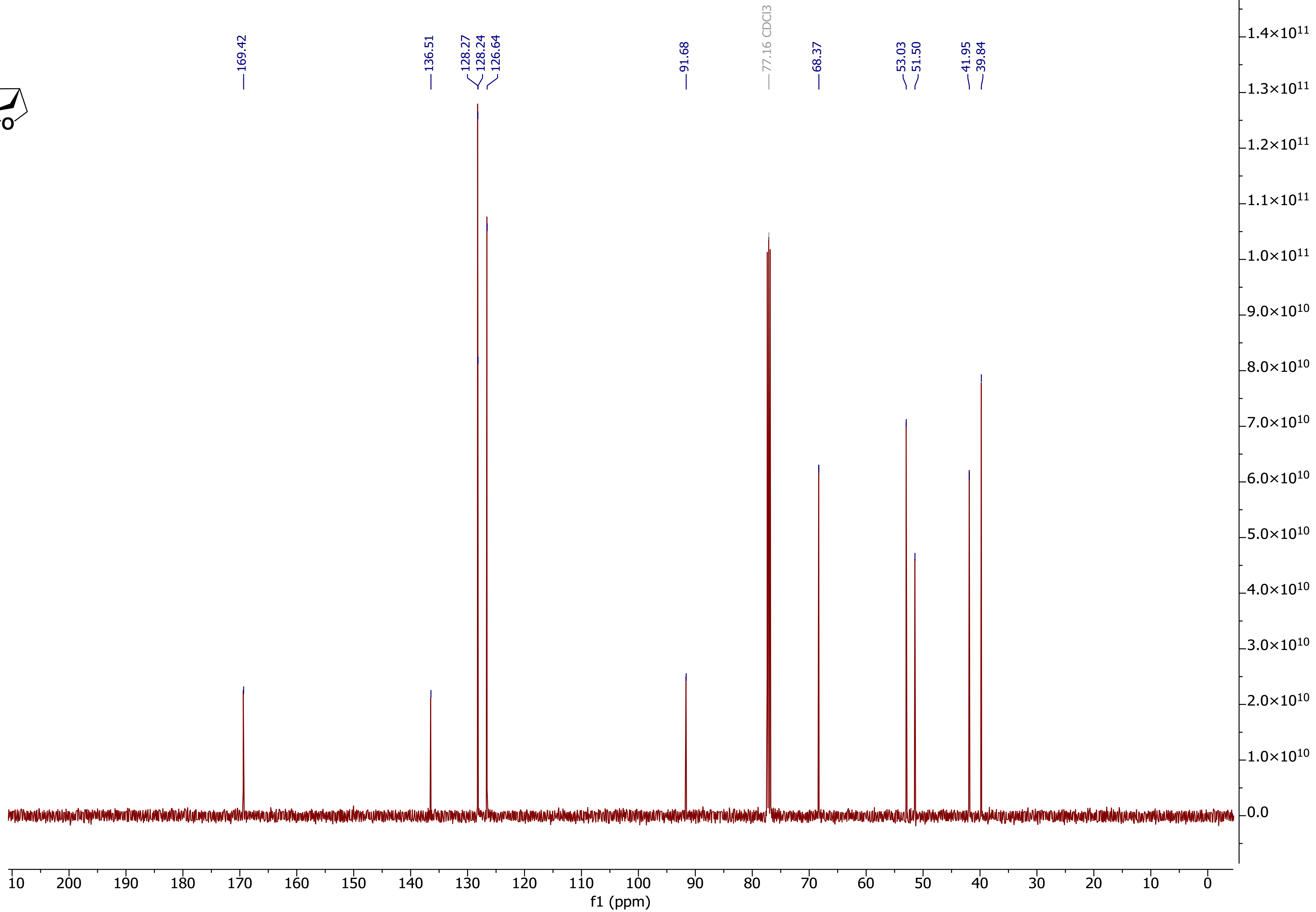

169.42
136.51
128.27
128.24
126.64
91.68
77.16 CDCl3
68.37
53.03
51.50
41.95
39.84
f1 (ppm)

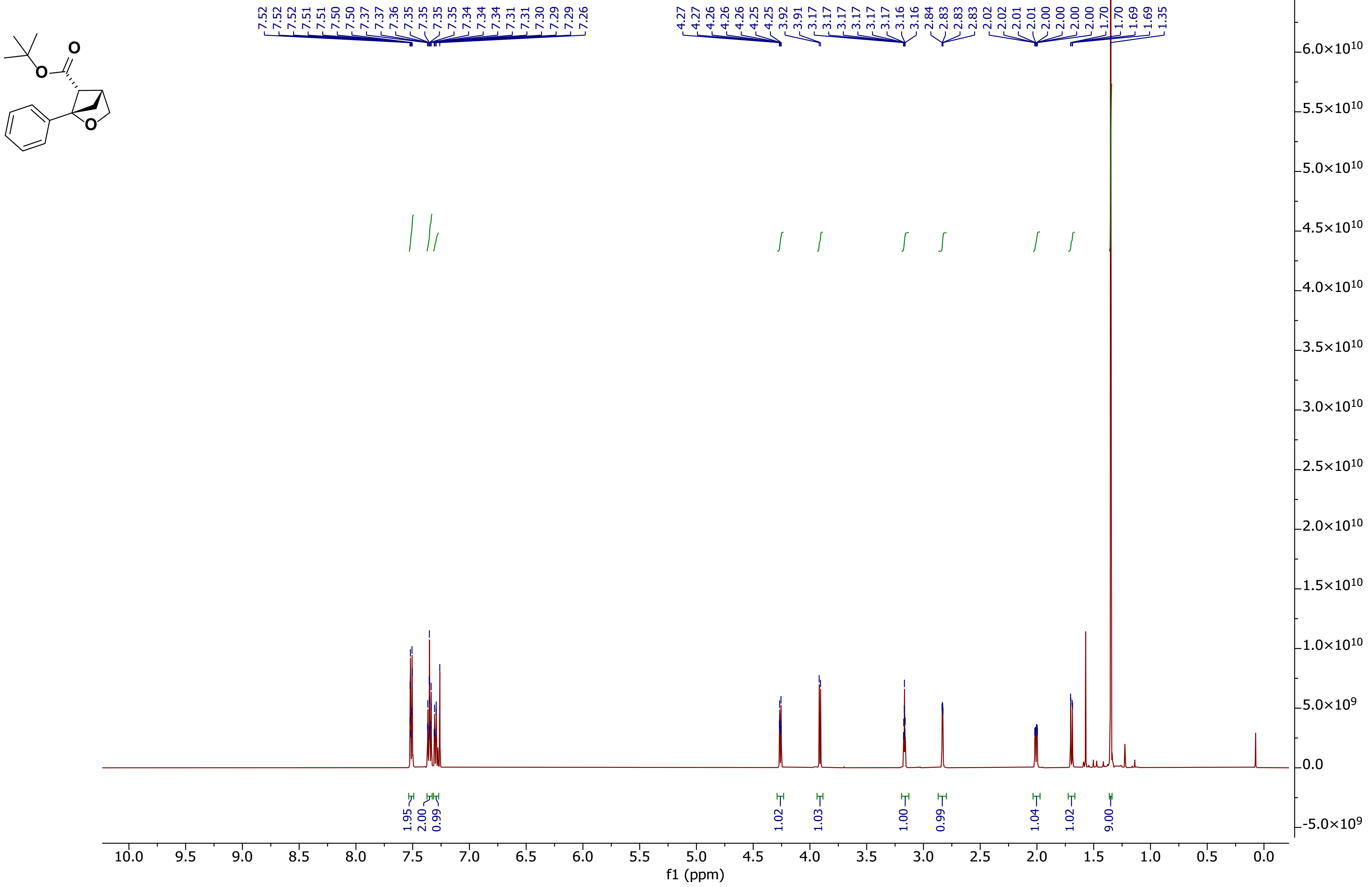

O
O
O
7.52 7.52 7.52 7.51 7.51 7.50 7.50 7.37 7.37 7.36 7.35 7.35 7.35 7.35 7.34 7.34 7.34 7.31 7.31 7.30 7.29 7.29 7.26
4.27 4.27 4.26 4.26 4.25 4.25 3.92 3.91 3.17 3.17 3.17 3.17 3.17 3.17 3.16 3.16 2.84 2.83 2.83 2.83 2.02 2.02 2.01 2.01 2.00 2.00 2.00 2.00 1.70 1.70 1.69 1.69 1.35
1.95 2.00 0.99 1.02 1.03 1.00 0.99 1.04 1.02 9.00
10.0 9.5 9.0 8.5 8.0 7.5 7.0 6.5 6.0 5.5 5.0 4.5 4.0 3.5 3.0 2.5 2.0 1.5 1.0 0.5 0.0
f1 (ppm)
6.0×10^10 5.5×10^10 5.0×10^10 4.5×10^10 4.0×10^10 3.5×10^10 3.0×10^10 2.5×10^10 2.0×10^10 1.5×10^10 1.0×10^10 5.0×10^9 0.0 -5.0×10^9

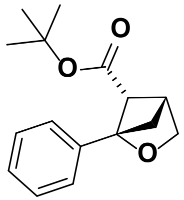

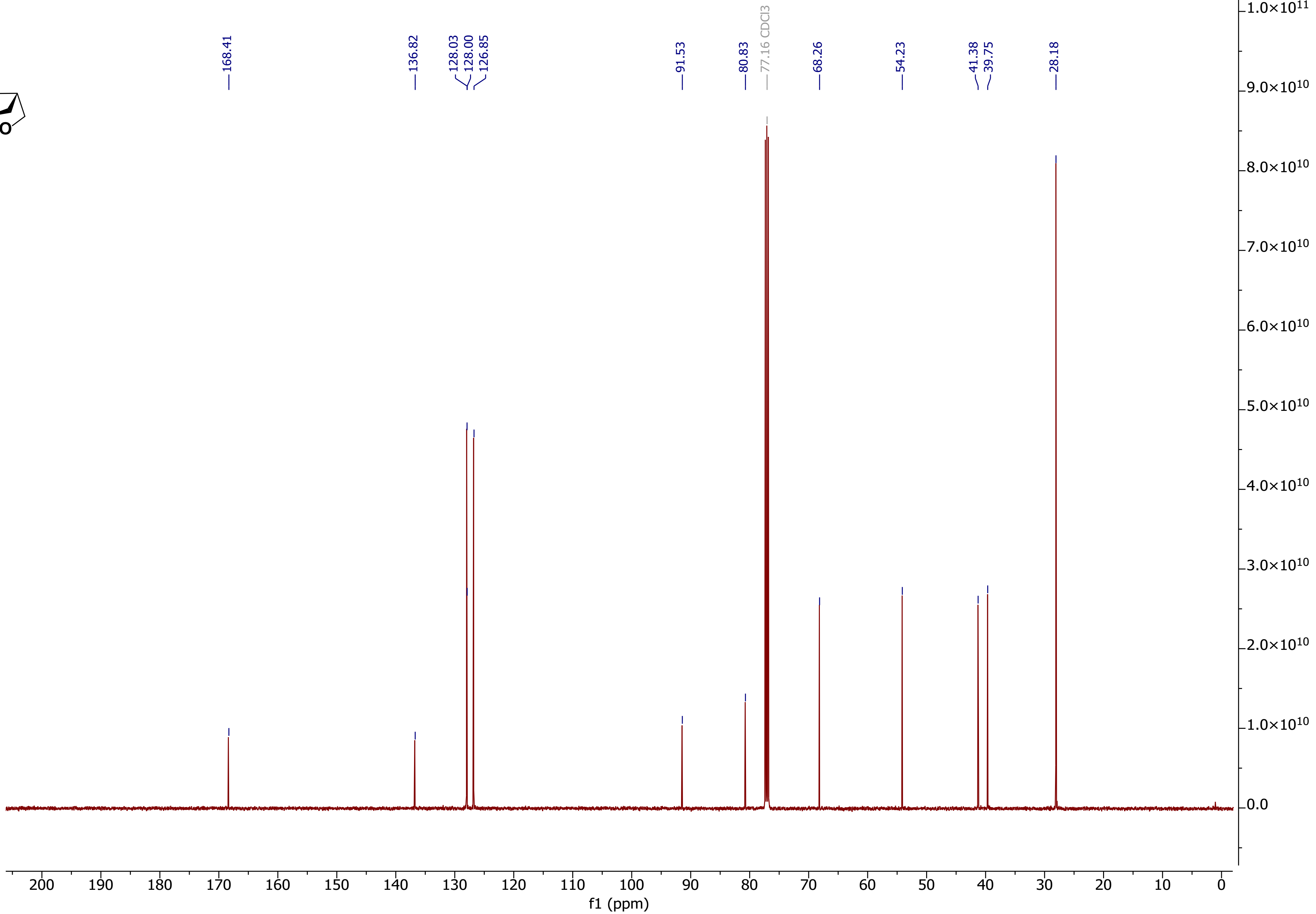
168.41
136.82
128.03
128.00
126.85
91.53
80.83
77.16 CDCl3
68.26
54.23
41.38
39.75
28.18
1.0×10¹¹
9.0×10¹⁰
8.0×10¹⁰
7.0×10¹⁰
6.0×10¹⁰
5.0×10¹⁰
4.0×10¹⁰
3.0×10¹⁰
2.0×10¹⁰
1.0×10¹⁰
0.0
200
190
180
170
160
150
140
130
120
110
100
90
80
70
60
50
40
30
20
10
0
f1 (ppm)

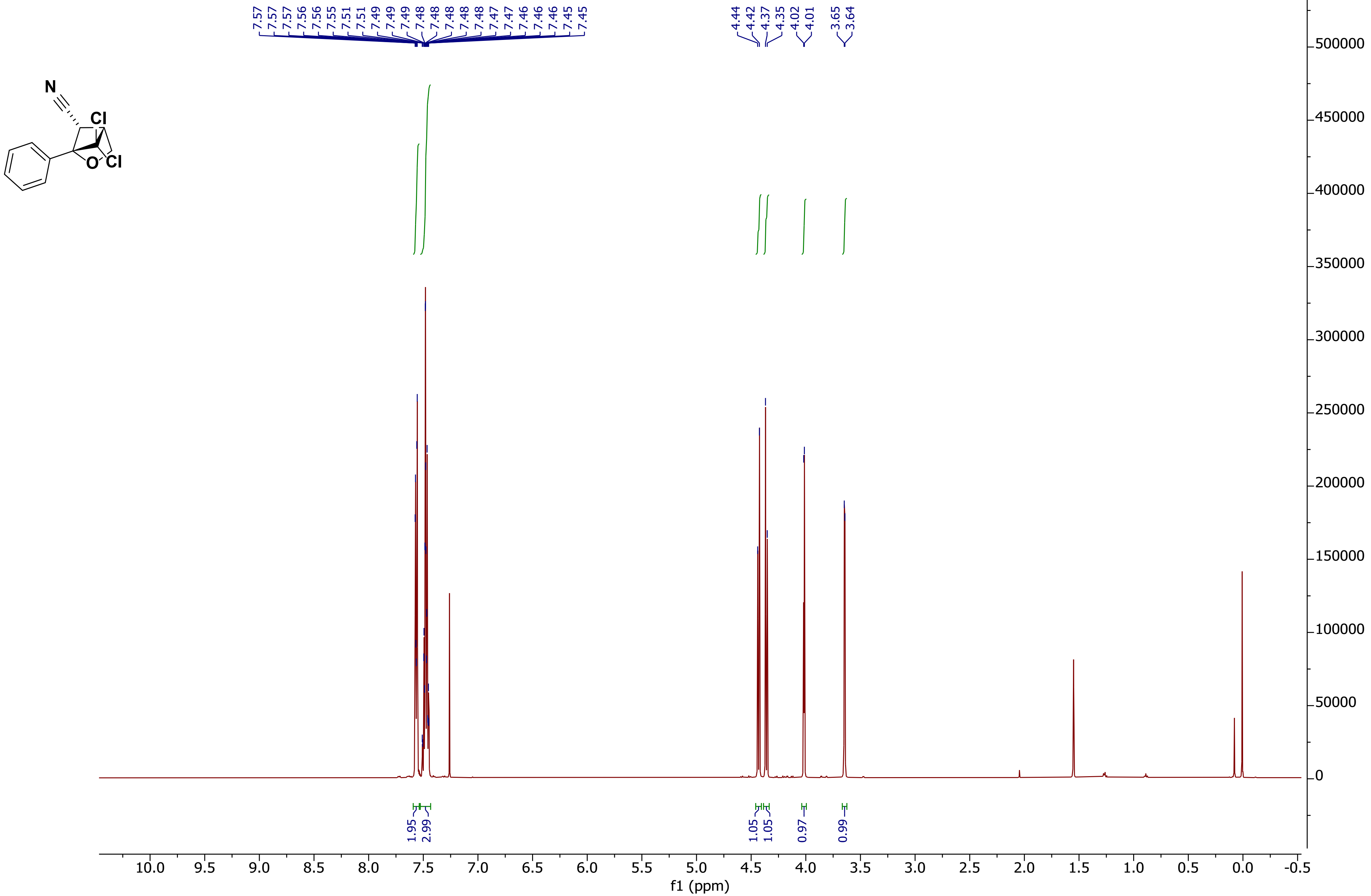

N
Cl
O
Cl
7.57
7.57
7.57
7.56
7.56
7.55
7.51
7.51
7.49
7.49
7.49
7.48
7.48
7.48
7.48
7.47
7.47
7.46
7.46
7.46
7.45
7.45
4.44
4.42
4.37
4.35
4.02
4.01
3.65
3.64
1.95
2.99
1.05
1.05
0.97
0.99
f1 (ppm)

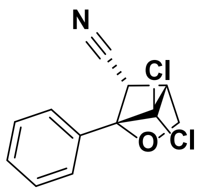

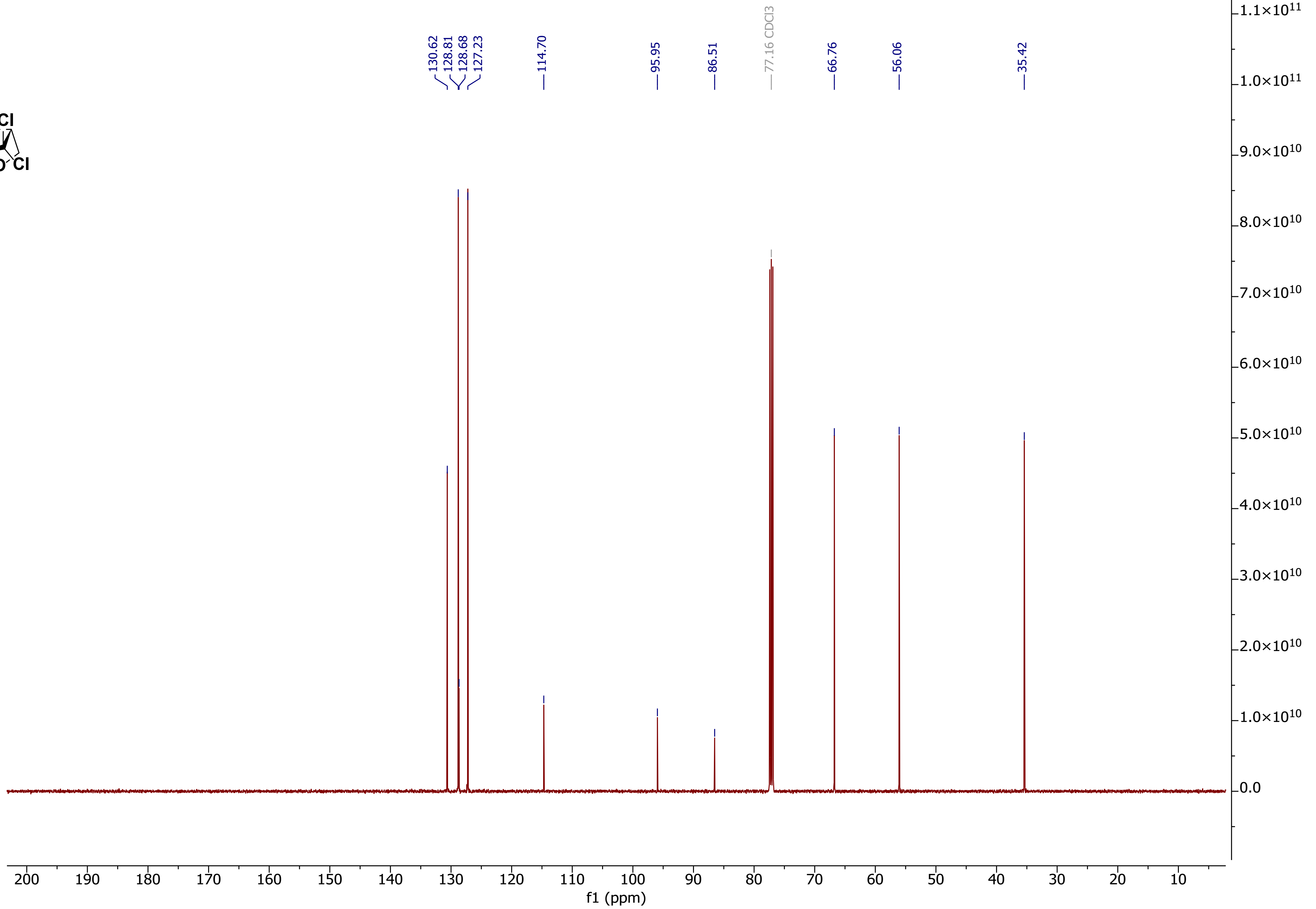
130.62
128.81
128.68
127.23
114.70
95.95
86.51
77.16 CDCl3
66.76
56.06
35.42
200
190
180
170
160
150
140
130
120
110
100
90
80
70
60
50
40
30
20
10
f1 (ppm)
1.1×10^11
1.0×10^11
9.0×10^10
8.0×10^10
7.0×10^10
6.0×10^10
5.0×10^10
4.0×10^10
3.0×10^10
2.0×10^10
1.0×10^10
0.0

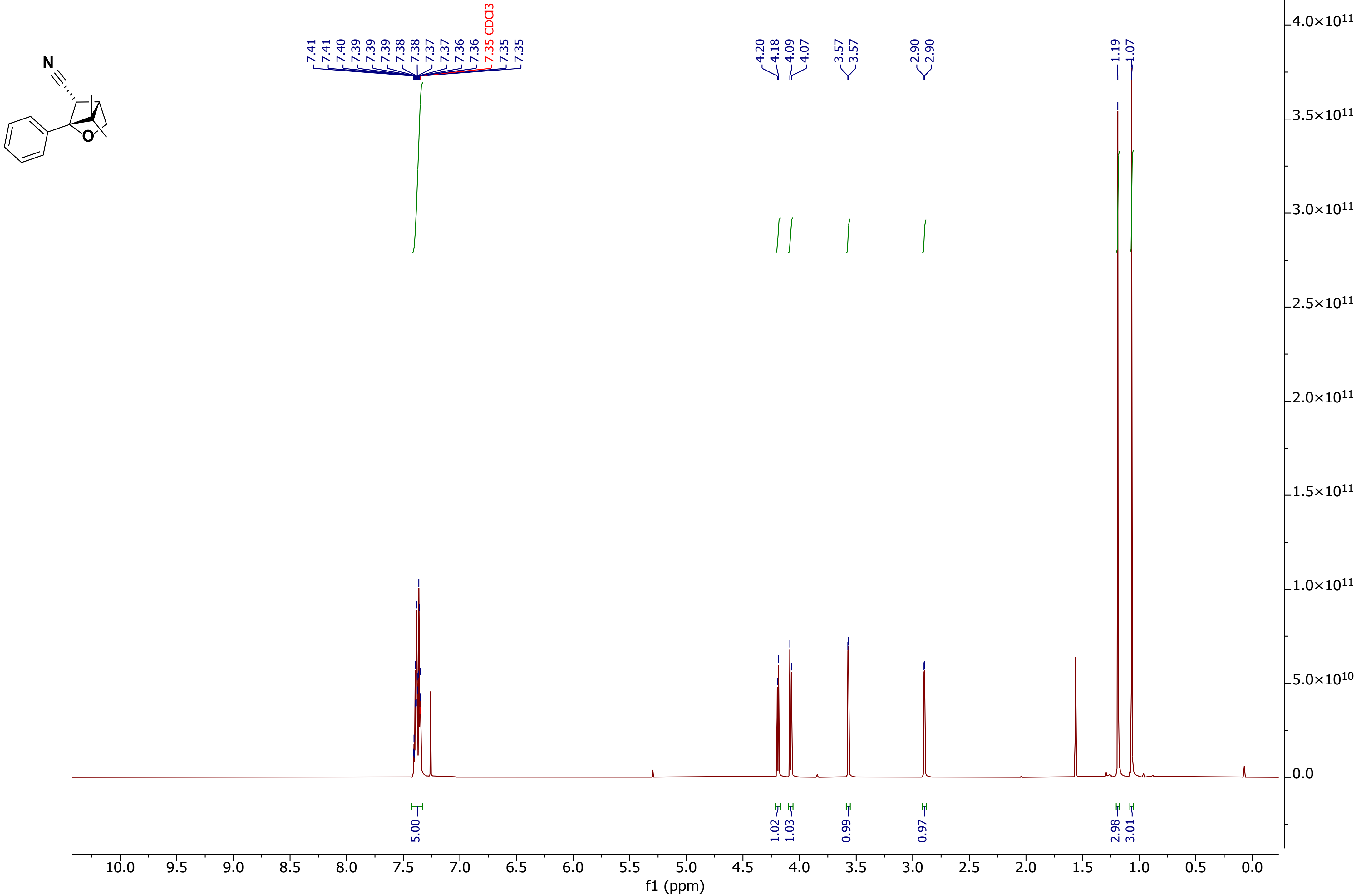
N
O
7.41
7.41
7.40
7.39
7.39
7.39
7.38
7.38
7.37
7.37
7.36
7.36
7.35 CDCl3
7.35
7.35
4.20
4.18
4.09
4.07
3.57
3.57
2.90
2.90
1.19
1.07
5.00
1.02
1.03
0.99
0.97
2.98
3.01
4.0×10^11
3.5×10^11
3.0×10^11
2.5×10^11
2.0×10^11
1.5×10^11
1.0×10^11
5.0×10^10
0.0
10.0
9.5
9.0
8.5
8.0
7.5
7.0
6.5
6.0
5.5
5.0
4.5
4.0
3.5
3.0
2.5
2.0
1.5
1.0
0.5
0.0
f1 (ppm)

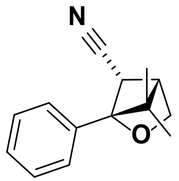

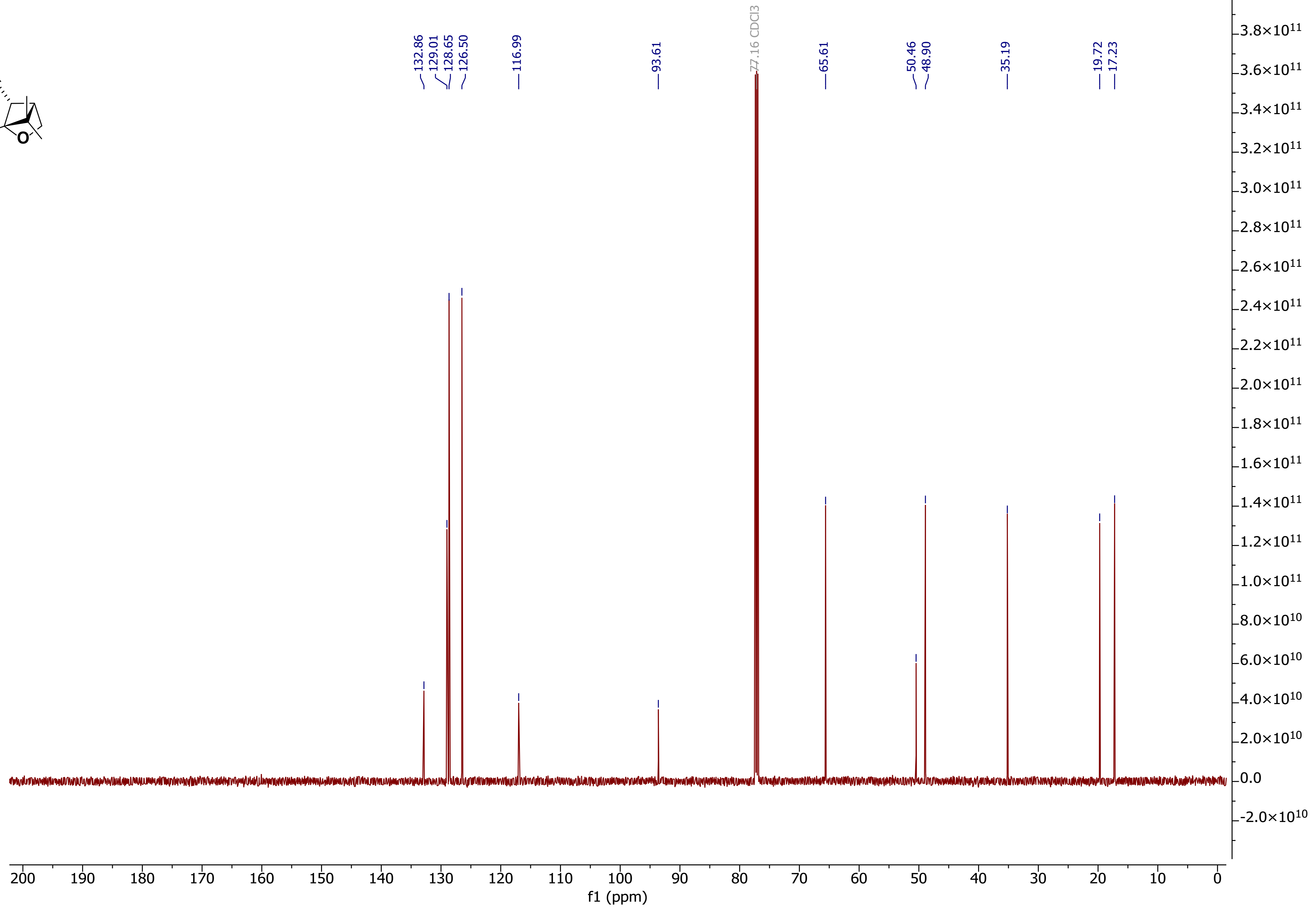
132.86
129.01
128.65
126.50
116.99
93.61
77.16 CDCl3
65.61
50.46
48.90
35.19
19.72
17.23
3.8×10^11
3.6×10^11
3.4×10^11
3.2×10^11
3.0×10^11
2.8×10^11
2.6×10^11
2.4×10^11
2.2×10^11
2.0×10^11
1.8×10^11
1.6×10^11
1.4×10^11
1.2×10^11
1.0×10^11
8.0×10^10
6.0×10^10
4.0×10^10
2.0×10^10
0.0
-2.0×10^10
200
190
180
170
160
150
140
130
120
110
100
90
80
70
60
50
40
30
20
10
0
f1 (ppm)

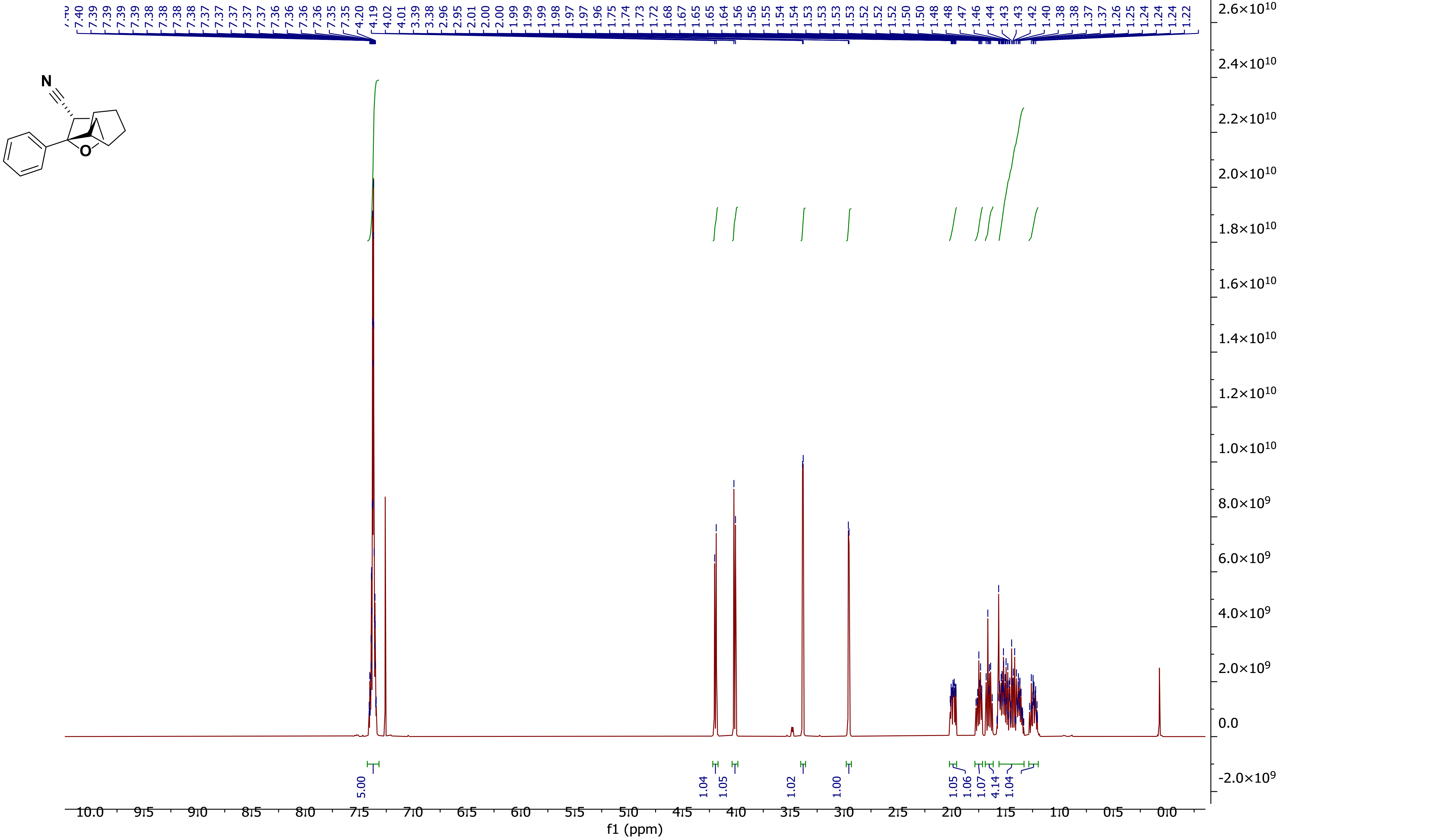
7.40 7.39 7.39 7.39 7.39 7.38 7.38 7.38 7.38 7.37 7.37 7.37 7.37 7.37 7.36 7.36 7.36 7.36 7.35 7.35 4.20 4.19 4.02 4.01 3.39 3.38 2.96 2.95 2.01 2.00 2.00 1.99 1.99 1.99 1.98 1.97 1.97 1.96 1.75 1.74 1.73 1.72 1.68 1.67 1.65 1.65 1.64 1.56 1.56 1.55 1.54 1.54 1.53 1.53 1.53 1.53 1.52 1.52 1.52 1.50 1.50 1.48 1.48 1.47 1.46 1.44 1.43 1.43 1.42 1.40 1.38 1.38 1.37 1.37 1.26 1.25 1.24 1.24 1.24 1.22
N
O
5.00
1.04
1.05
1.02
1.00
1.05
1.06
1.07
4.14
1.04
f1 (ppm)

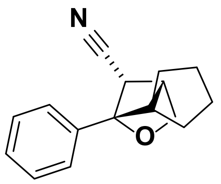
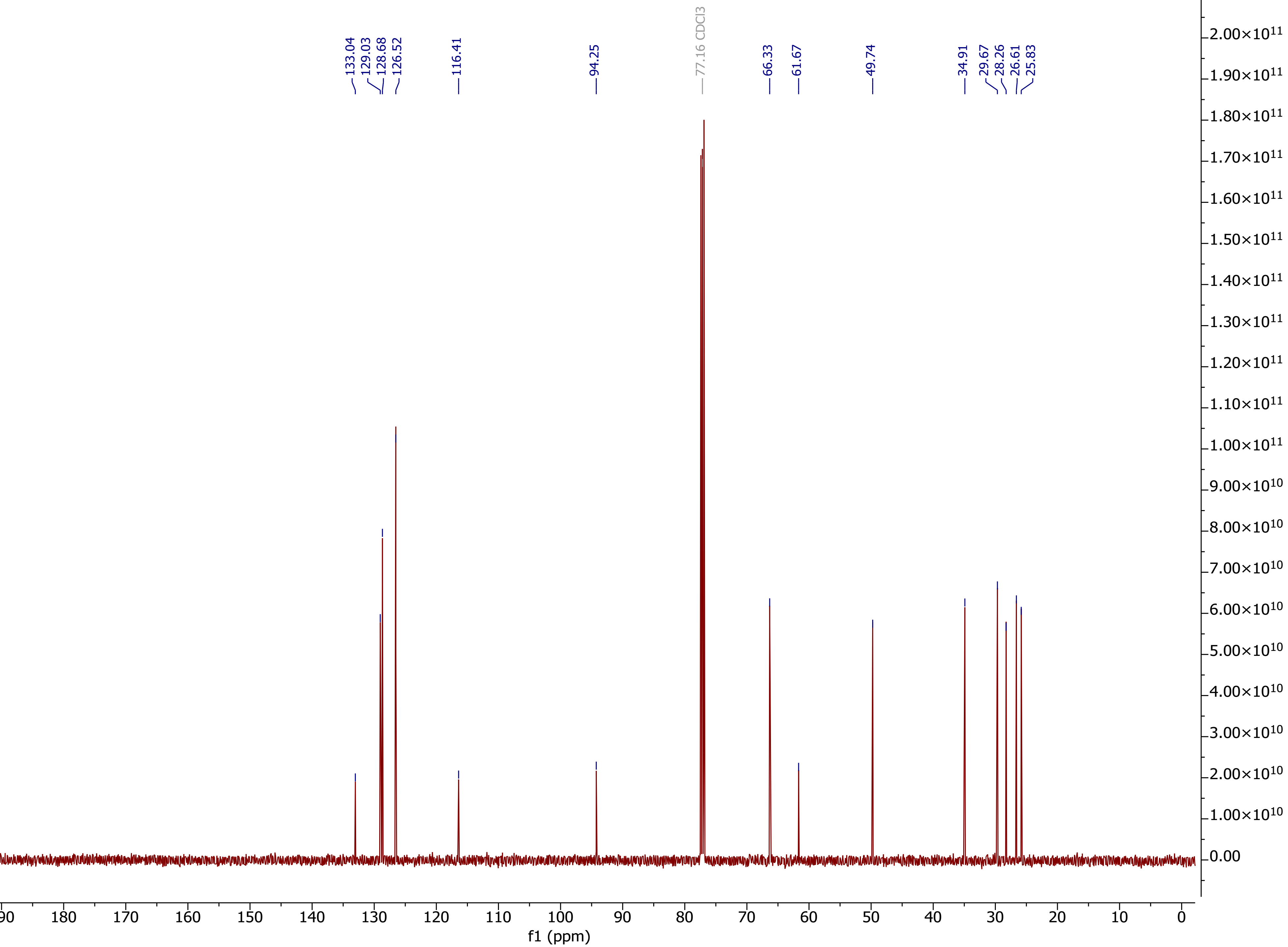
133.04
129.03
128.68
126.52
116.41
94.25
77.16 CDCl3
66.33
61.67
49.74
34.91
29.67
28.26
26.61
25.83
f1 (ppm)

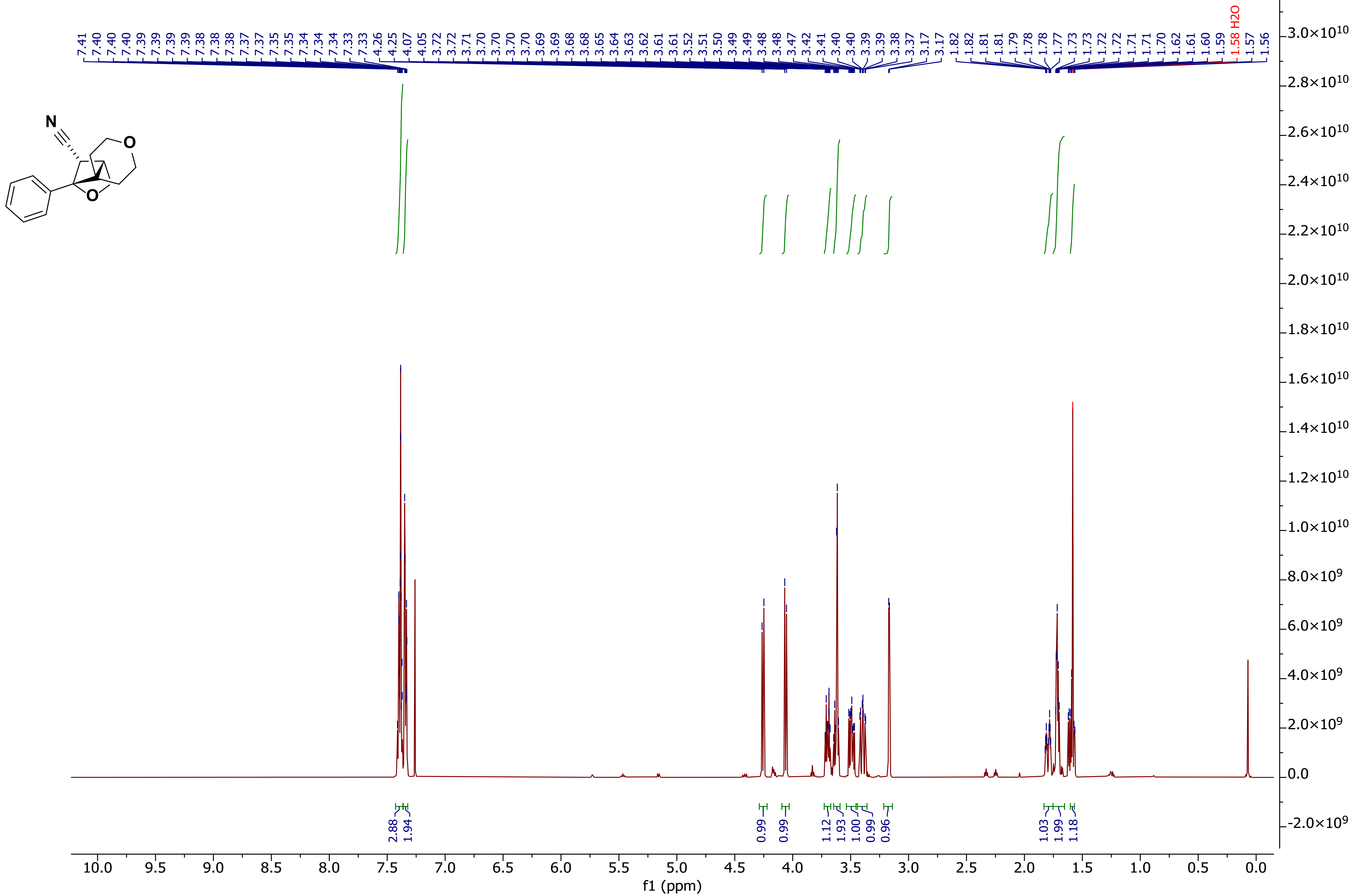

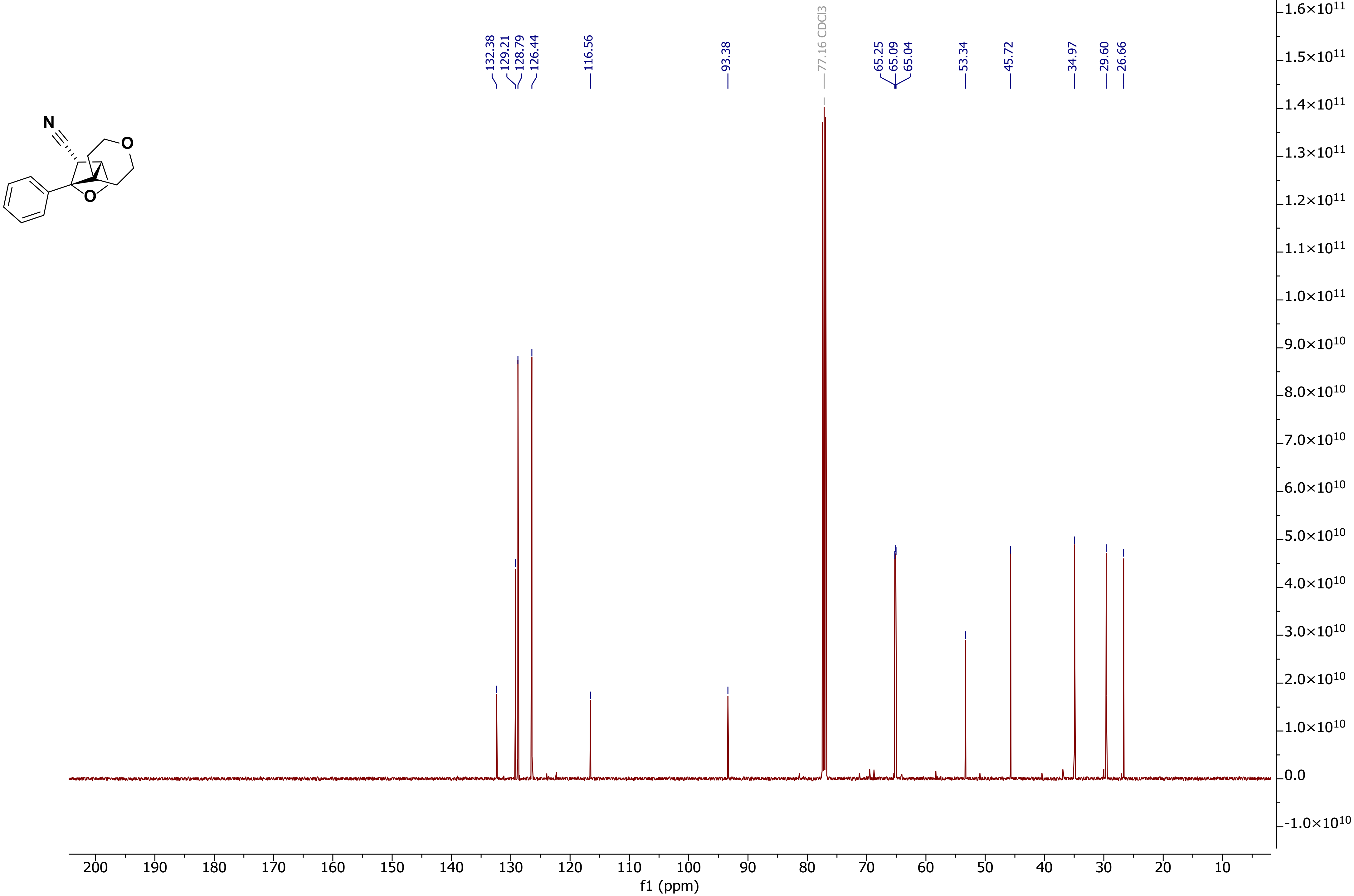
132.38
129.21
128.79
126.44
116.56
93.38
77.16 CDCl3
65.25
65.09
65.04
53.34
45.72
34.97
29.60
26.66
N
O
O
f1 (ppm)
200 190 180 170 160 150 140 130 120 110 100 90 80 70 60 50 40 30 20 10
1.6×10^11
1.5×10^11
1.4×10^11
1.3×10^11
1.2×10^11
1.1×10^11
1.0×10^11
9.0×10^10
8.0×10^10
7.0×10^10
6.0×10^10
5.0×10^10
4.0×10^10
3.0×10^10
2.0×10^10
1.0×10^10
0.0
-1.0×10^10

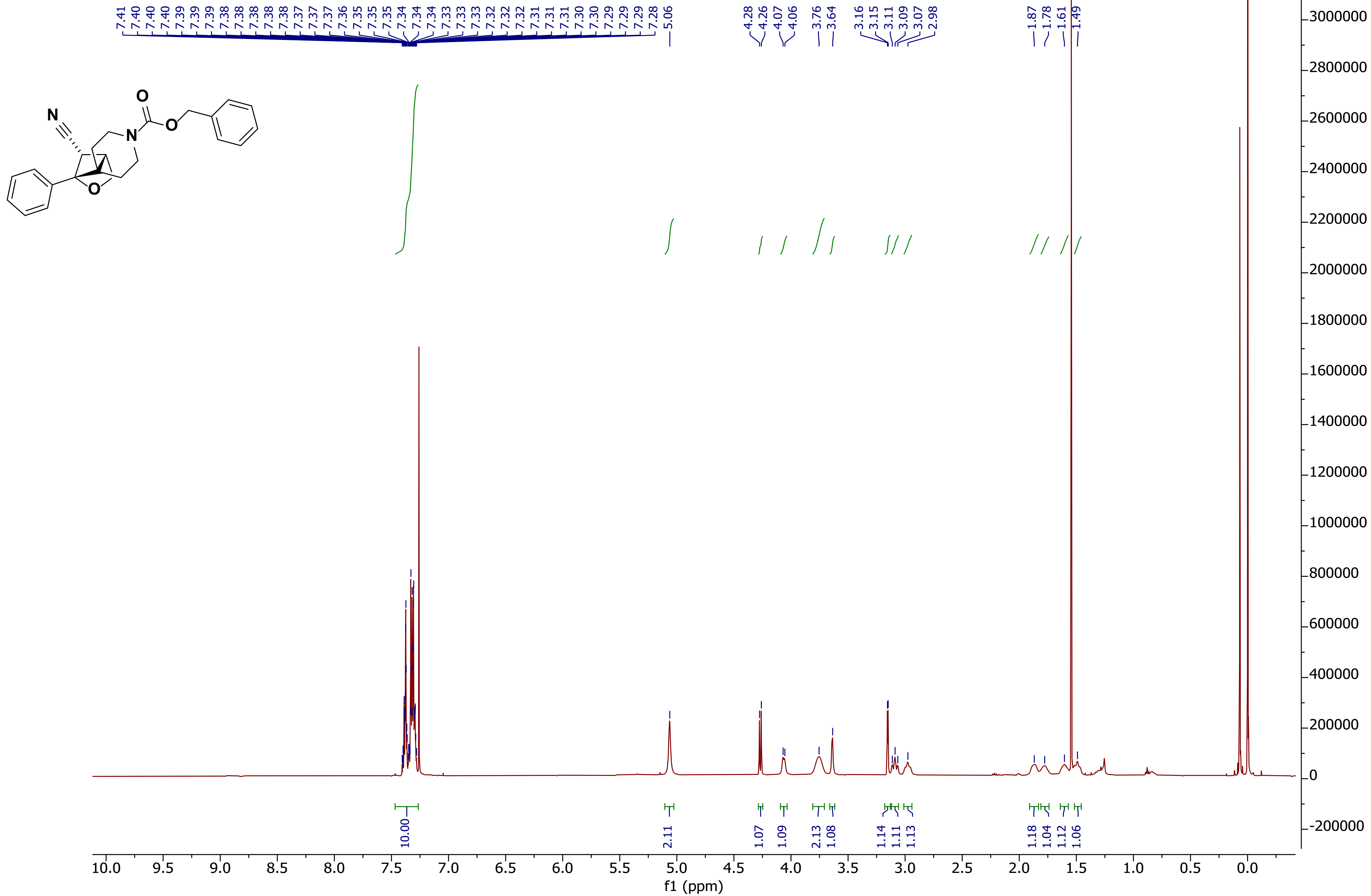

7.41 7.40 7.40 7.40 7.39 7.39 7.39 7.38 7.38 7.38 7.38 7.38 7.37 7.37 7.37 7.36 7.35 7.35 7.35 7.34 7.34 7.34 7.33 7.33 7.33 7.32 7.32 7.32 7.31 7.31 7.31 7.30 7.30 7.29 7.29 7.29 7.28 5.06
4.28 4.26 4.07 4.06 3.76 3.64 3.16 3.15 3.11 3.09 3.07 2.98
1.87 1.78 1.61 1.49
N
O
N
O
O
10.00
2.11
1.07
1.09
2.13
1.08
1.14
1.11
1.13
1.18
1.04
1.12
1.06
3000000
2800000
2600000
2400000
2200000
2000000
1800000
1600000
1400000
1200000
1000000
800000
600000
400000
200000
0
-200000
10.0 9.5 9.0 8.5 8.0 7.5 7.0 6.5 6.0 5.5 5.0 4.5 4.0 3.5 3.0 2.5 2.0 1.5 1.0 0.5 0.0
f1 (ppm)

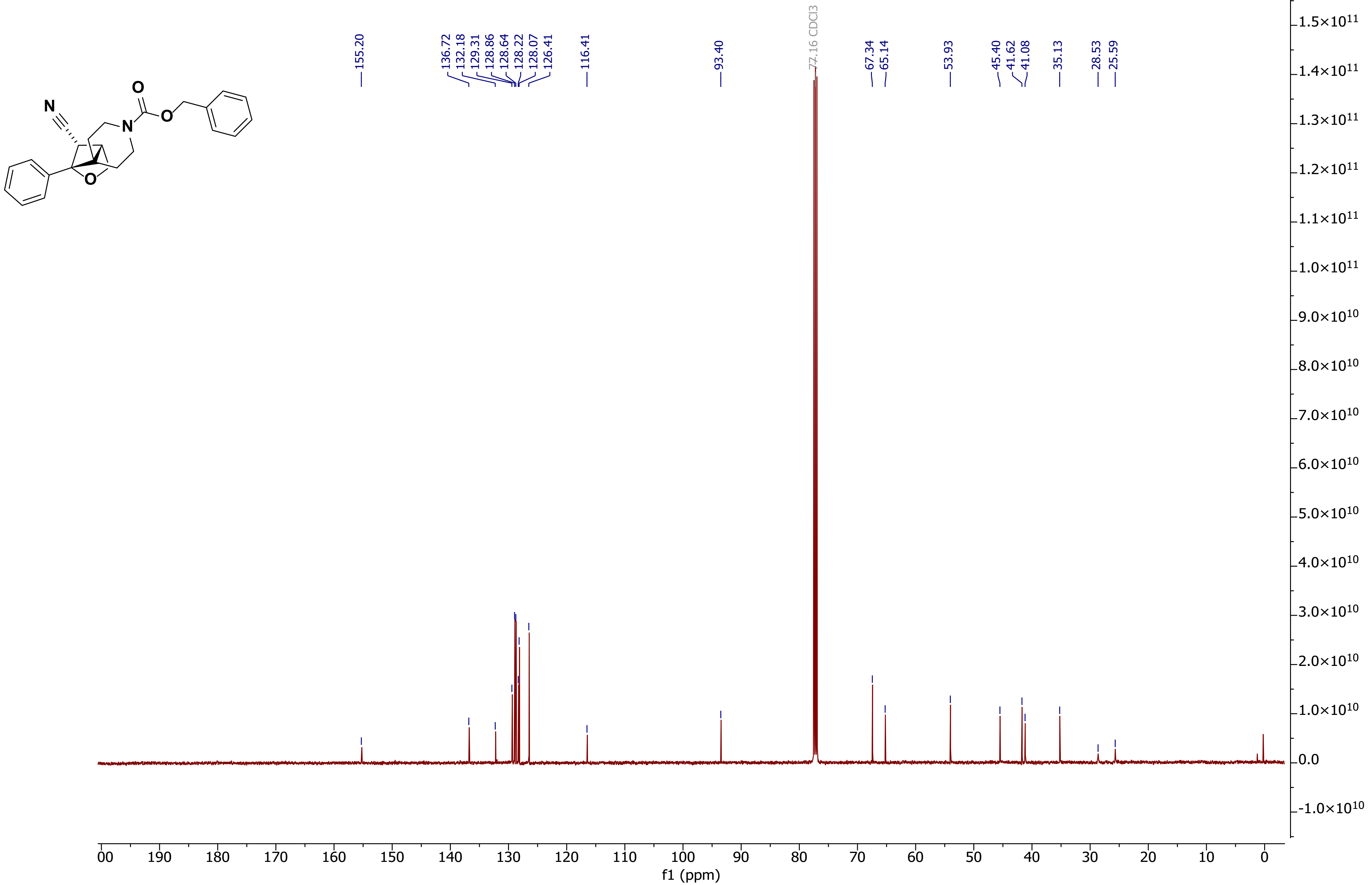

N
O
N
O
O
155.20
136.72
132.18
129.31
128.86
128.64
128.22
128.07
126.41
116.41
93.40
77.16 CDCl3
67.34
65.14
53.93
45.40
41.62
41.08
35.13
28.53
25.59
f1 (ppm)

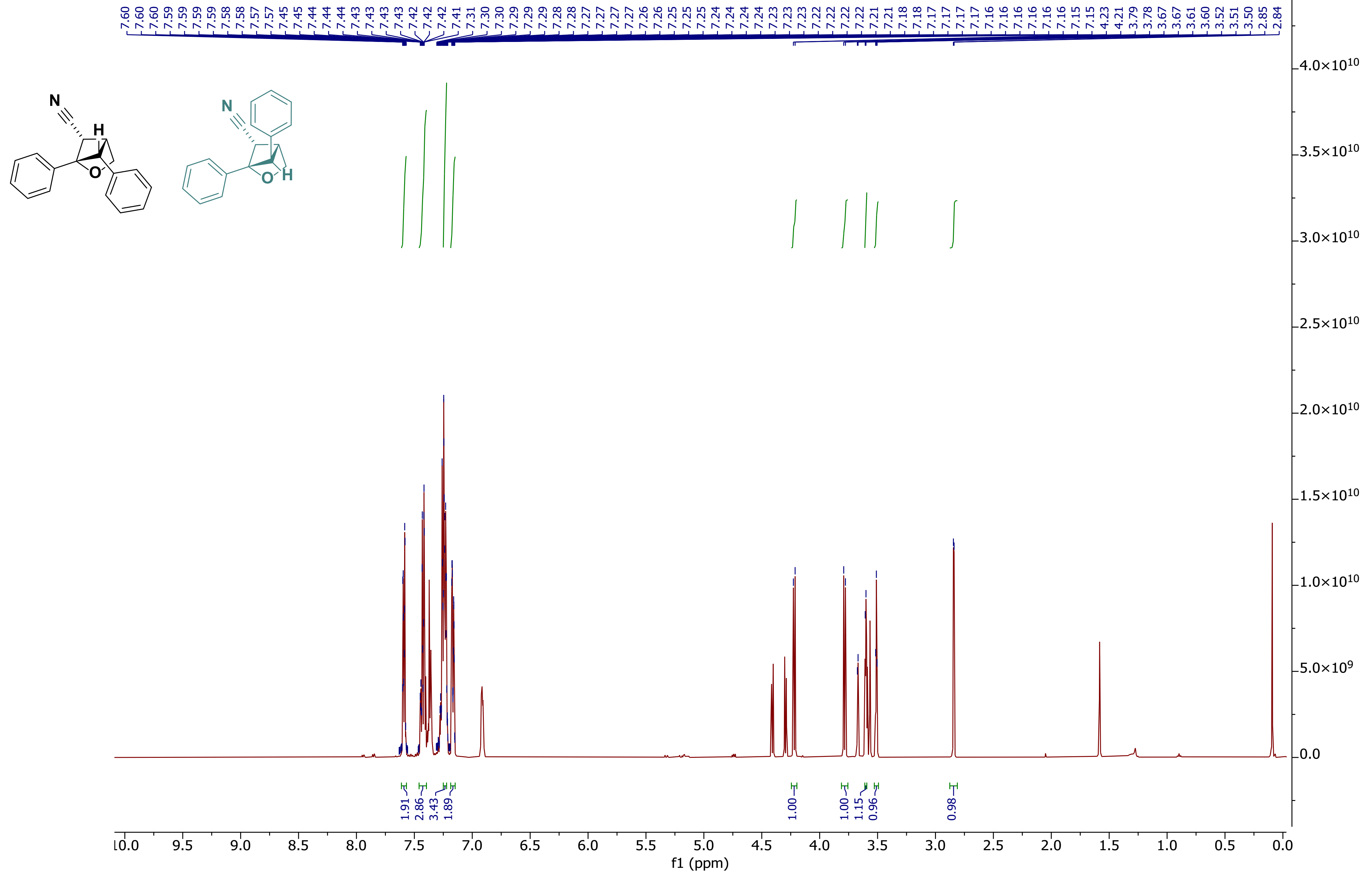

7.60 7.60 7.60 7.59 7.59 7.59 7.59 7.58 7.58 7.57 7.57 7.45 7.45 7.44 7.44 7.44 7.43 7.43 7.43 7.43 7.42 7.42 7.42 7.41 7.31 7.30 7.30 7.29 7.29 7.29 7.28 7.28 7.27 7.27 7.27 7.27 7.26 7.26 7.25 7.25 7.25 7.24 7.24 7.24 7.23 7.23 7.23 7.22 7.22 7.22 7.22 7.21 7.21 7.18 7.18 7.17 7.17 7.17 7.17 7.16 7.16 7.16 7.16 7.16 7.15 7.15 4.23 4.21 3.79 3.78 3.67 3.67 3.61 3.60 3.52 3.51 3.50 2.85 2.84
N
H
O
N
O
H
4.0×10^10
3.5×10^10
3.0×10^10
2.5×10^10
2.0×10^10
1.5×10^10
1.0×10^10
5.0×10^9
0.0
1.91
2.86
3.43
1.89
1.00
1.00
1.15
0.96
0.98
10.0 9.5 9.0 8.5 8.0 7.5 7.0 6.5 6.0 5.5 5.0 4.5 4.0 3.5 3.0 2.5 2.0 1.5 1.0 0.5 0.0
f1 (ppm)

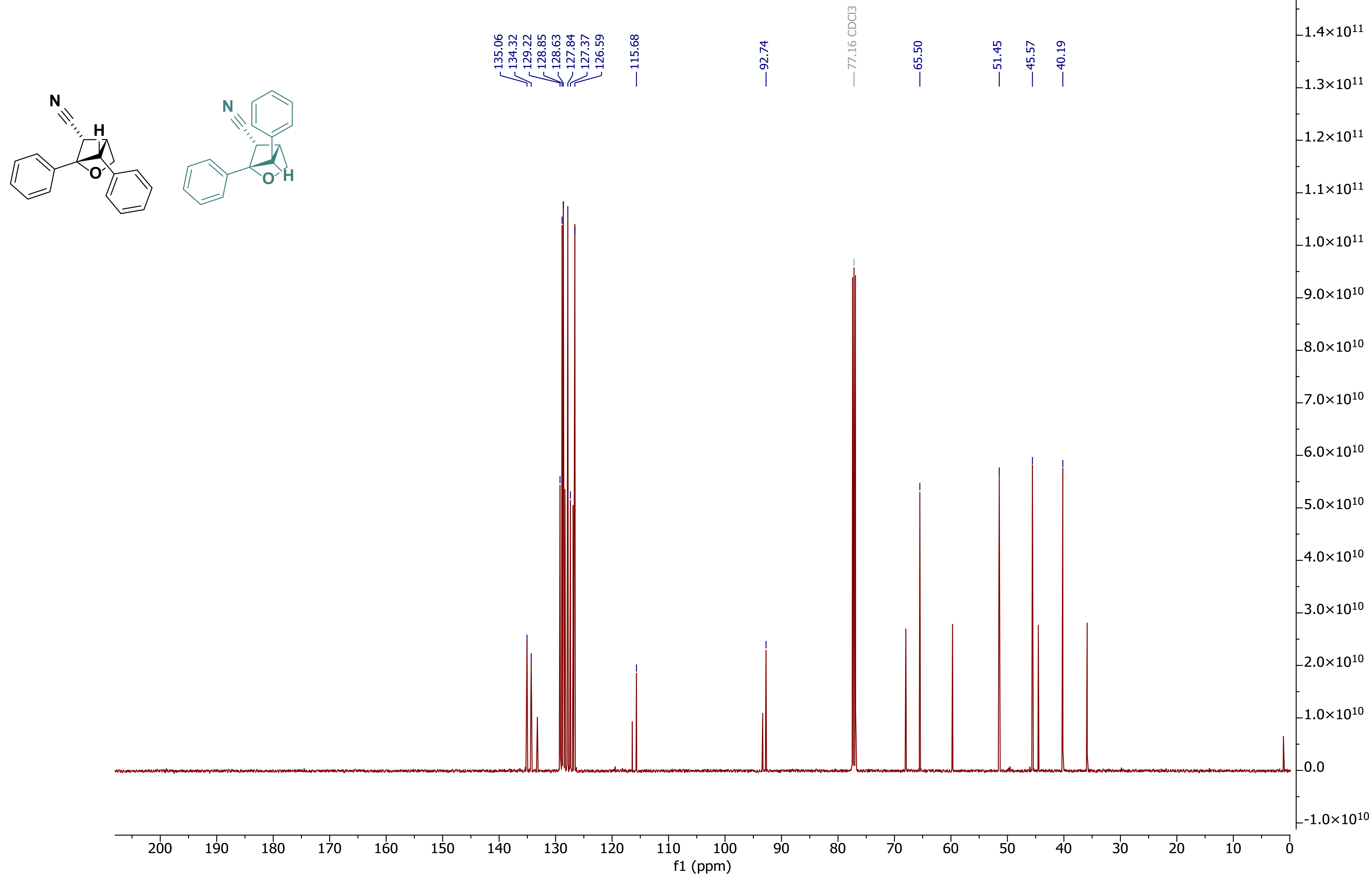
N
H
O
N
O
H
135.06
134.32
129.22
128.85
128.63
127.84
127.37
126.59
115.68
92.74
77.16 CDCl3
65.50
51.45
45.57
40.19
1.4×10^11
1.3×10^11
1.2×10^11
1.1×10^11
1.0×10^11
9.0×10^10
8.0×10^10
7.0×10^10
6.0×10^10
5.0×10^10
4.0×10^10
3.0×10^10
2.0×10^10
1.0×10^10
0.0
-1.0×10^10
200
190
180
170
160
150
140
130
120
110
100
90
80
70
60
50
40
30
20
10
0
f1 (ppm)

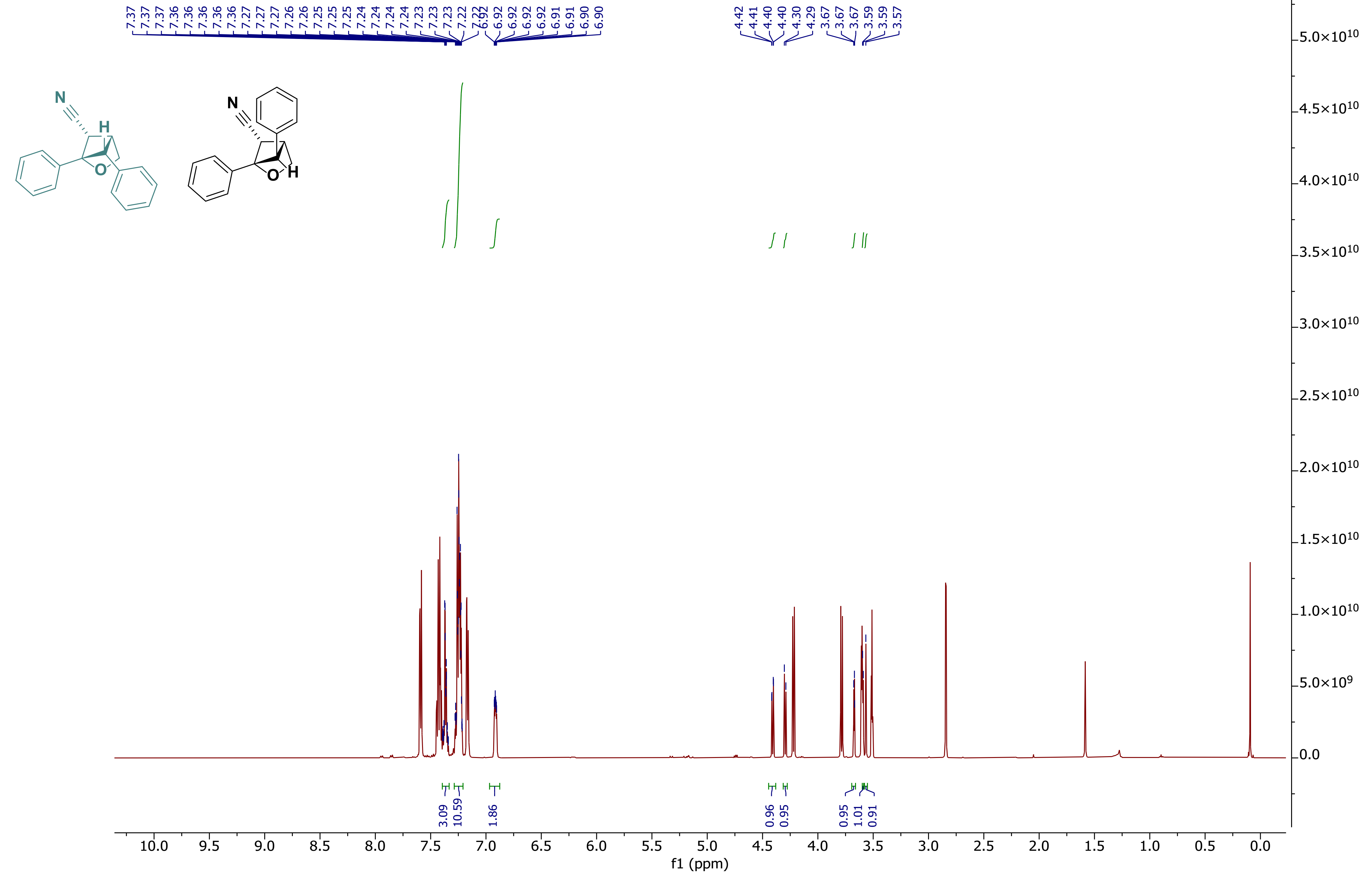
7.37
7.37
7.37
7.36
7.36
7.36
7.36
7.36
7.27
7.27
7.27
7.26
7.26
7.25
7.25
7.25
7.24
7.24
7.24
7.23
7.23
7.23
7.22
7.22
6.92
6.92
6.92
6.92
6.92
6.91
6.90
6.90
4.42
4.41
4.40
4.40
4.30
4.29
3.67
3.67
3.67
3.59
3.59
3.57
N
H
O
N
O
H
3.09
10.59
1.86
0.96
0.95
0.95
1.01
0.91
f1 (ppm)

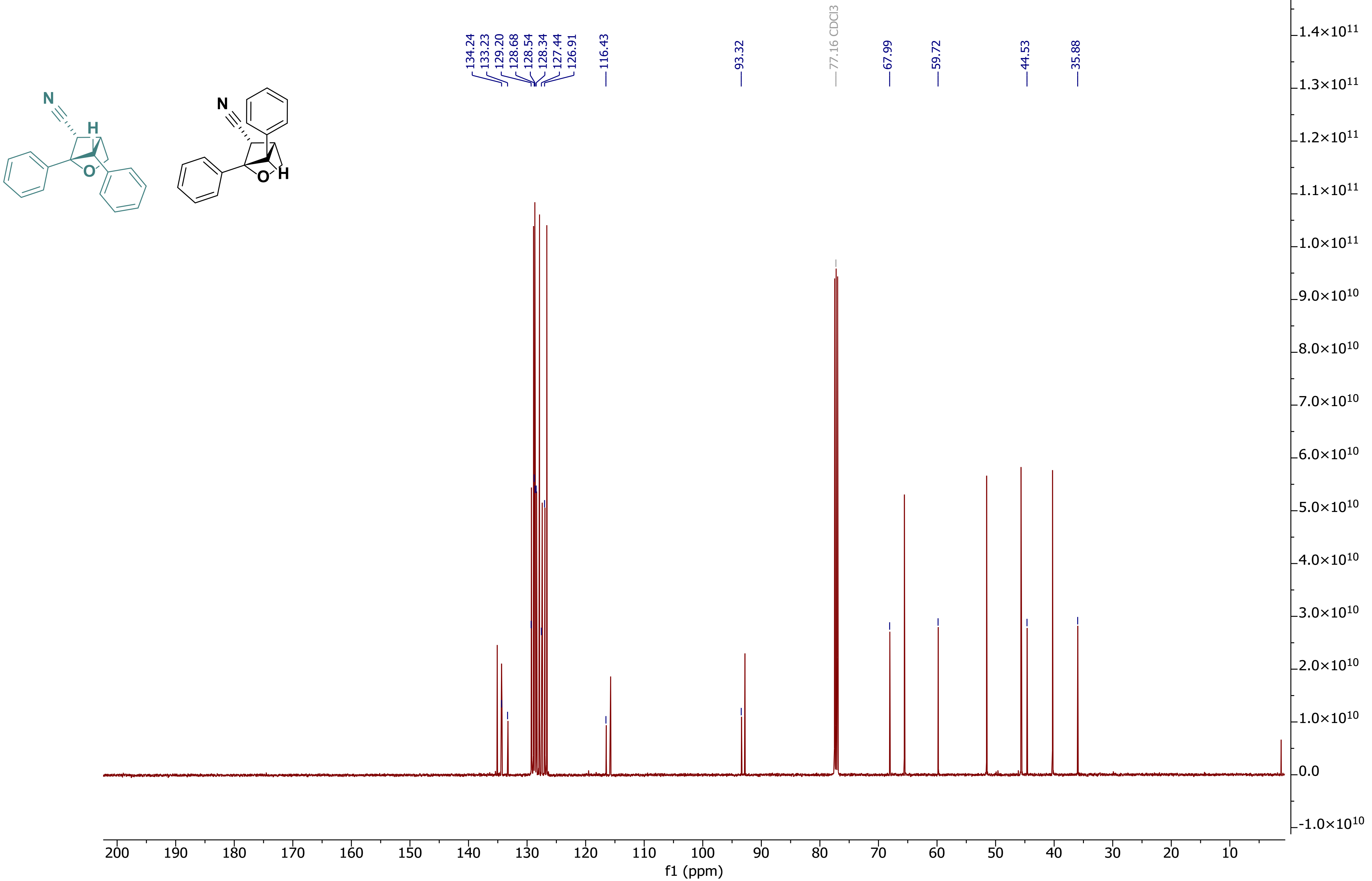
N
H
O
N
O
H
134.24
133.23
129.20
128.68
128.54
128.34
127.44
126.91
116.43
93.32
77.16 CDCl3
67.99
59.72
44.53
35.88
f1 (ppm)

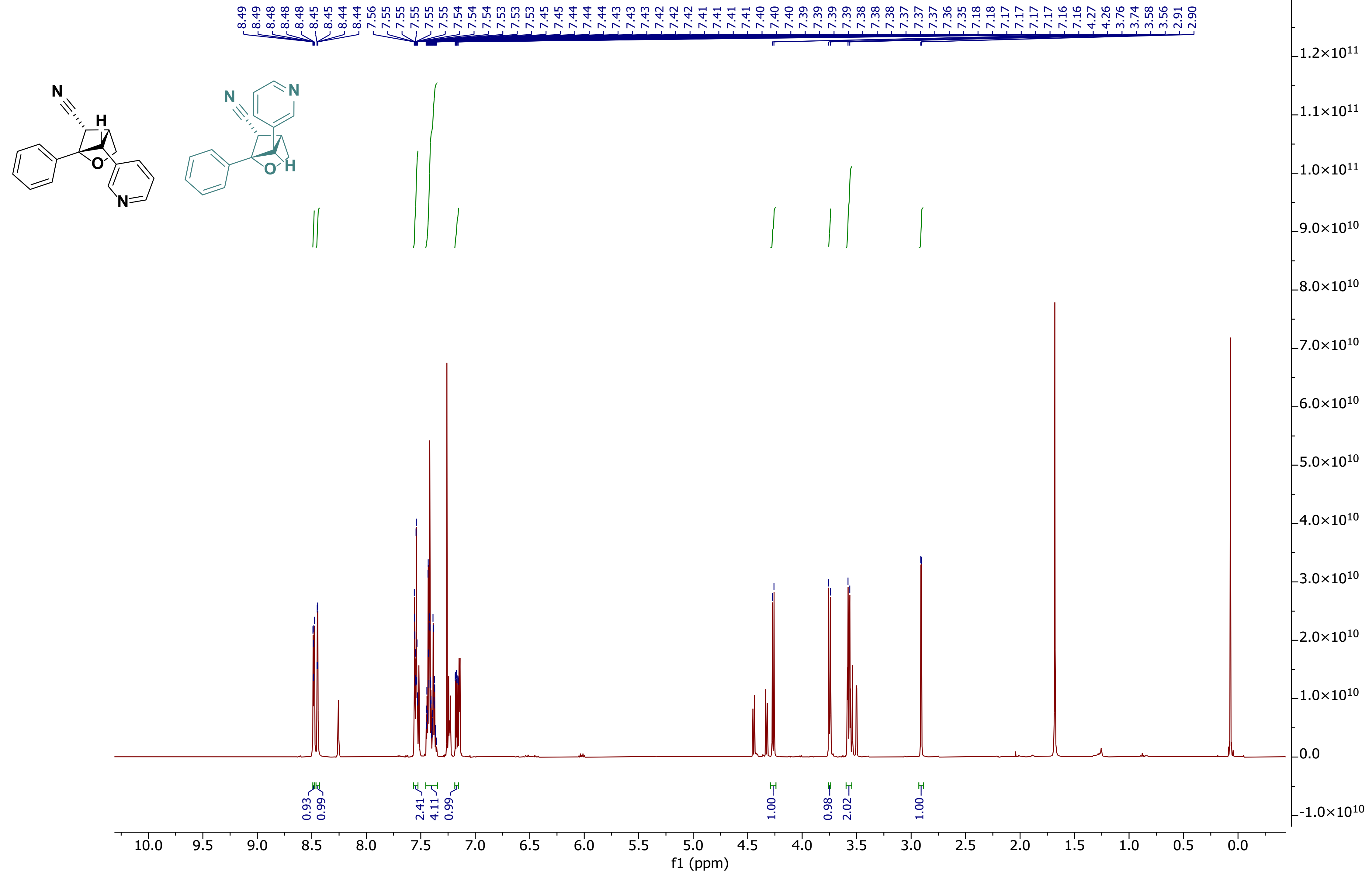

8.49 8.49 8.48 8.48 8.48 8.45 8.45 8.44 8.44 7.56 7.55 7.55 7.55 7.55 7.55 7.54 7.54 7.54 7.53 7.53 7.45 7.45 7.44 7.44 7.44 7.43 7.43 7.43 7.42 7.42 7.42 7.41 7.41 7.41 7.40 7.40 7.40 7.39 7.39 7.39 7.39 7.38 7.38 7.38 7.37 7.37 7.37 7.36 7.35 7.18 7.18 7.17 7.17 7.17 7.17 7.16 7.16 4.27 4.26 3.76 3.74 3.58 3.56 2.91 2.90
N
H
O
N
N
N
O
H
0.93
0.99
2.41
4.11
0.99
1.00
0.98
2.02
1.00
f1 (ppm)

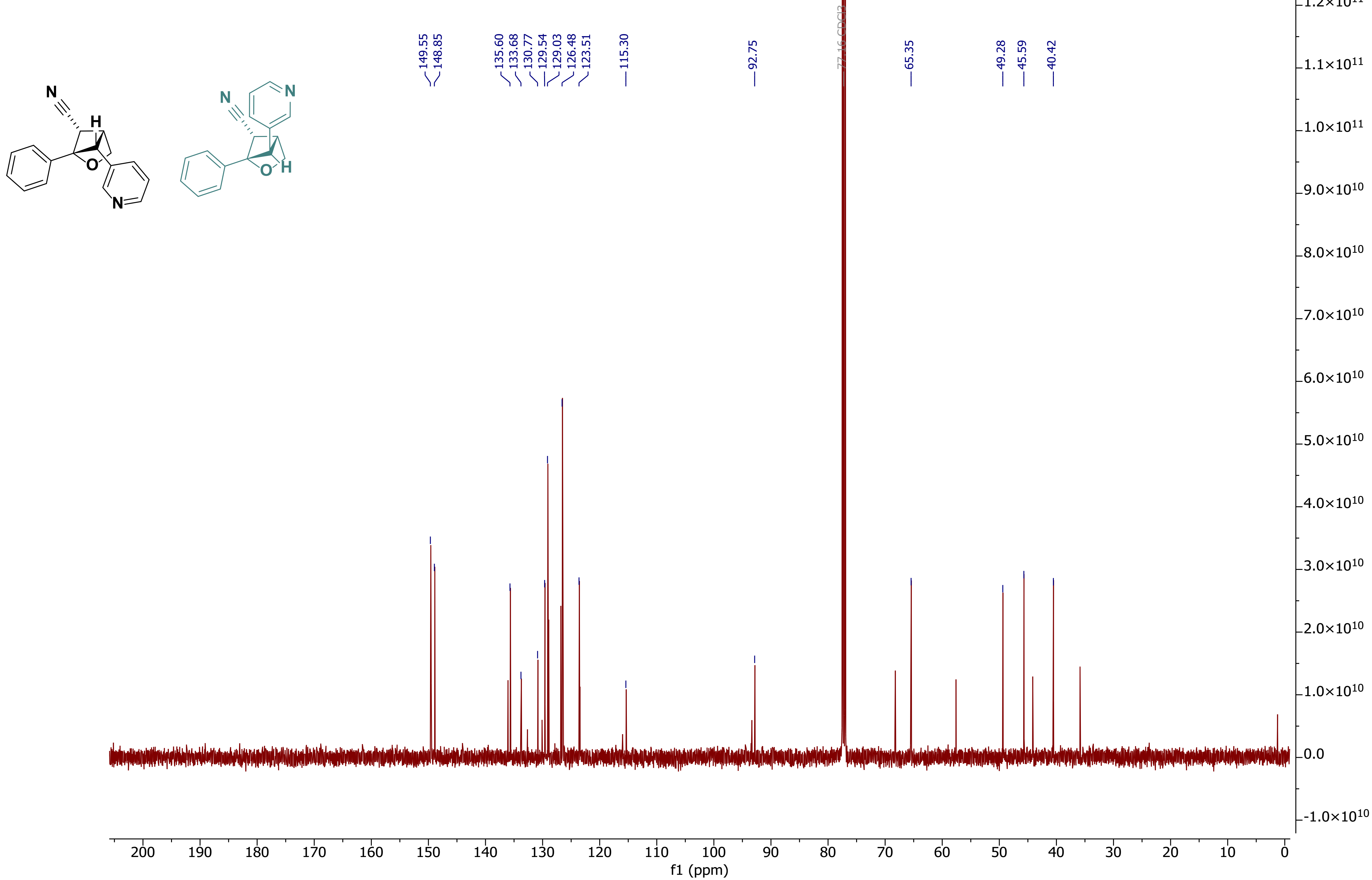
149.55
148.85
135.60
133.68
130.77
129.54
129.03
126.48
123.51
115.30
92.75
77.16 CDCl3
65.35
49.28
45.59
40.42
f1 (ppm)

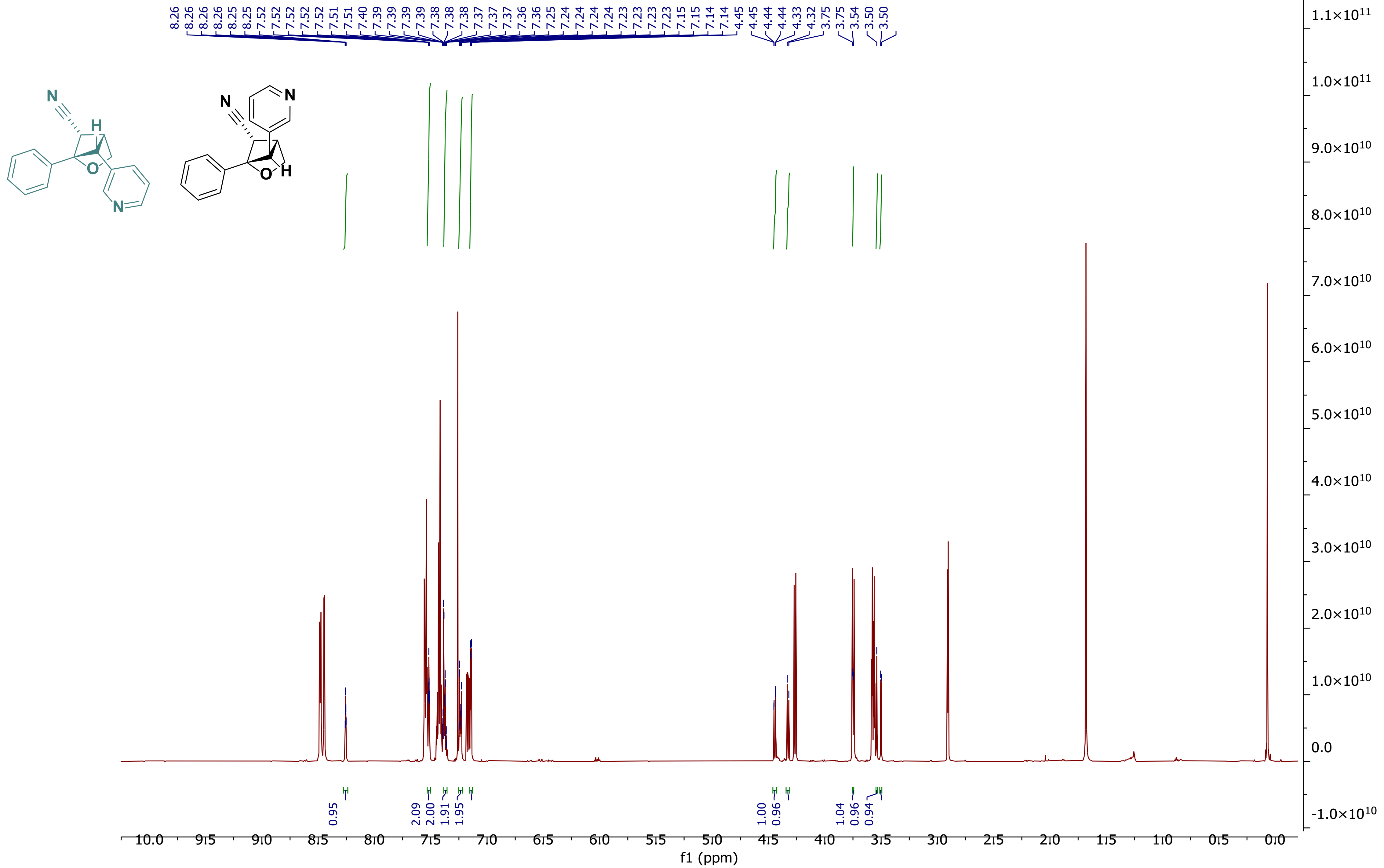

8.26 8.26 8.26 8.26 8.25 8.25 7.52 7.52 7.52 7.52 7.52 7.51 7.51 7.40 7.39 7.39 7.39 7.39 7.38 7.38 7.38 7.37 7.37 7.37 7.36 7.36 7.25 7.24 7.24 7.24 7.23 7.23 7.23 7.23 7.15 7.15 7.14 7.14 4.45 4.45 4.44 4.44 4.33 4.32 3.75 3.75 3.54 3.50 3.50
0.95
2.09
2.00
1.91
1.95
1.00
0.96
1.04
0.96
0.94
f1 (ppm)

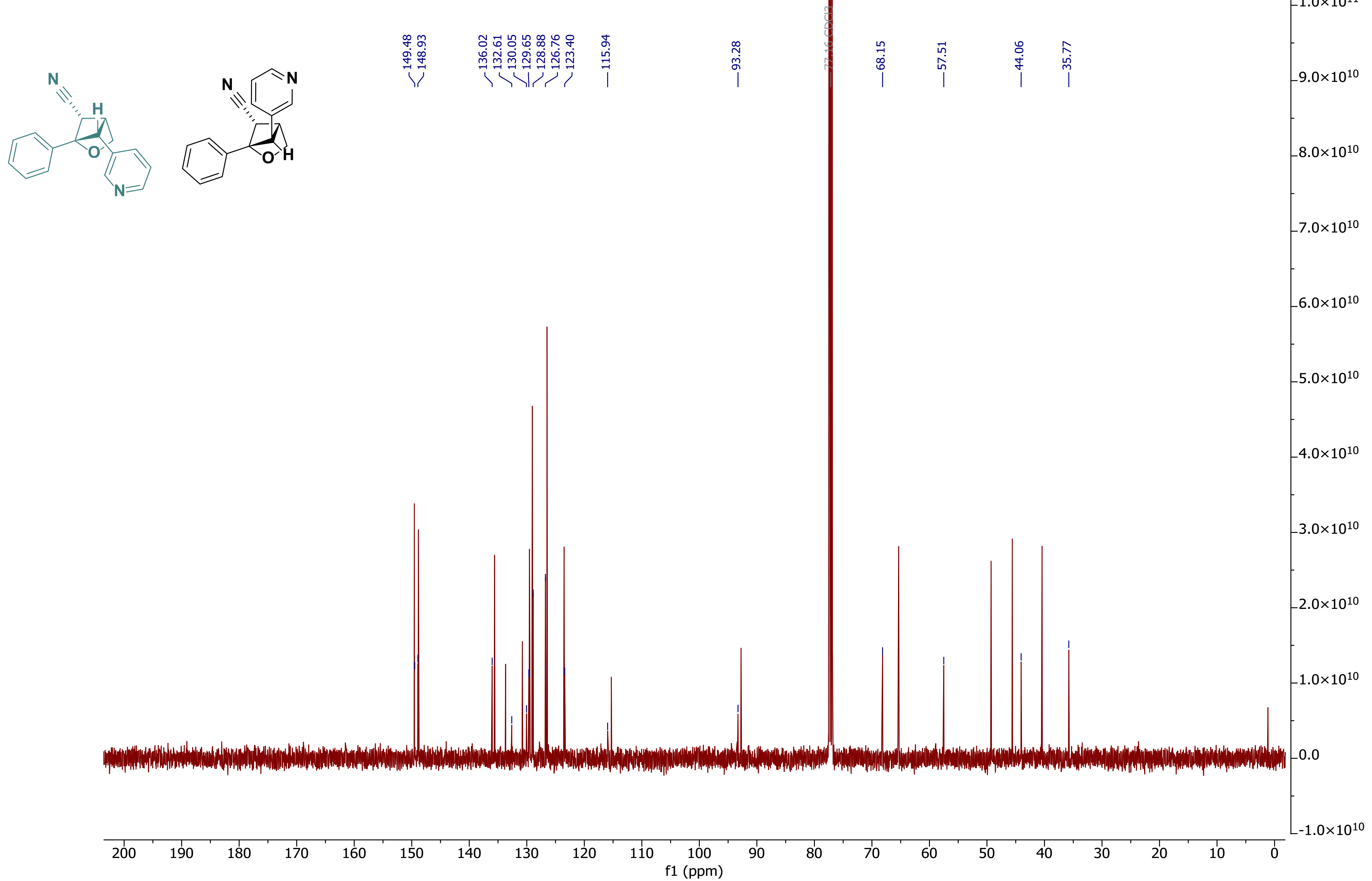

149.48
148.93
136.02
132.61
130.05
129.65
128.88
126.76
123.40
115.94
93.28
77.16 CDCl3
68.15
57.51
44.06
35.77
f1 (ppm)

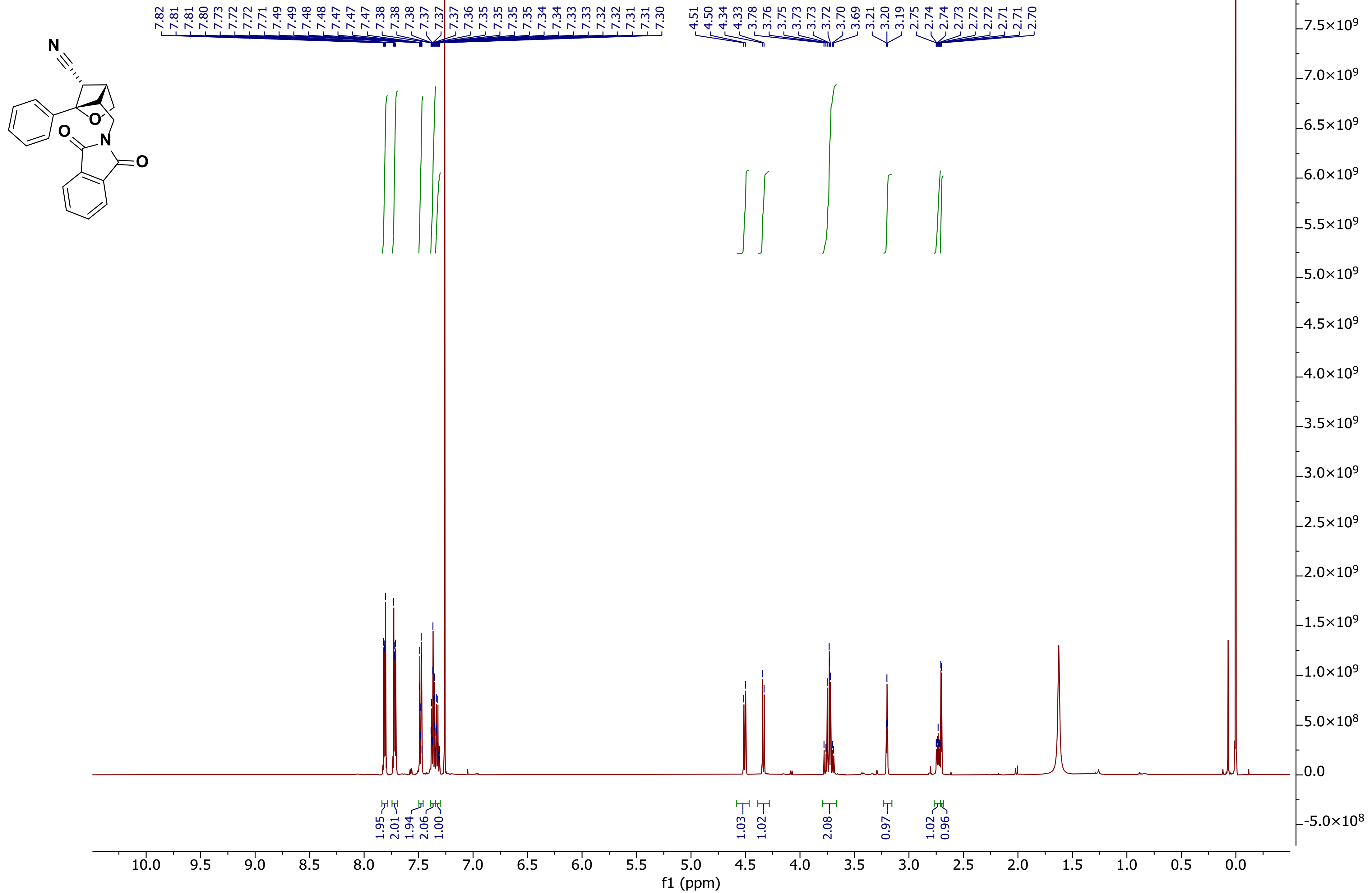
N
O
N
O
O
7.82
7.81
7.81
7.80
7.73
7.72
7.72
7.71
7.49
7.49
7.48
7.48
7.47
7.47
7.47
7.38
7.38
7.38
7.37
7.37
7.37
7.36
7.35
7.35
7.35
7.35
7.34
7.34
7.33
7.33
7.32
7.32
7.31
7.31
7.30
4.51
4.50
4.34
4.33
3.78
3.76
3.75
3.73
3.73
3.72
3.70
3.69
3.21
3.20
3.19
2.75
2.74
2.74
2.73
2.72
2.72
2.71
2.71
2.70
1.95
2.01
1.94
2.06
1.00
1.03
1.02
2.08
0.97
1.02
0.96
f1 (ppm)

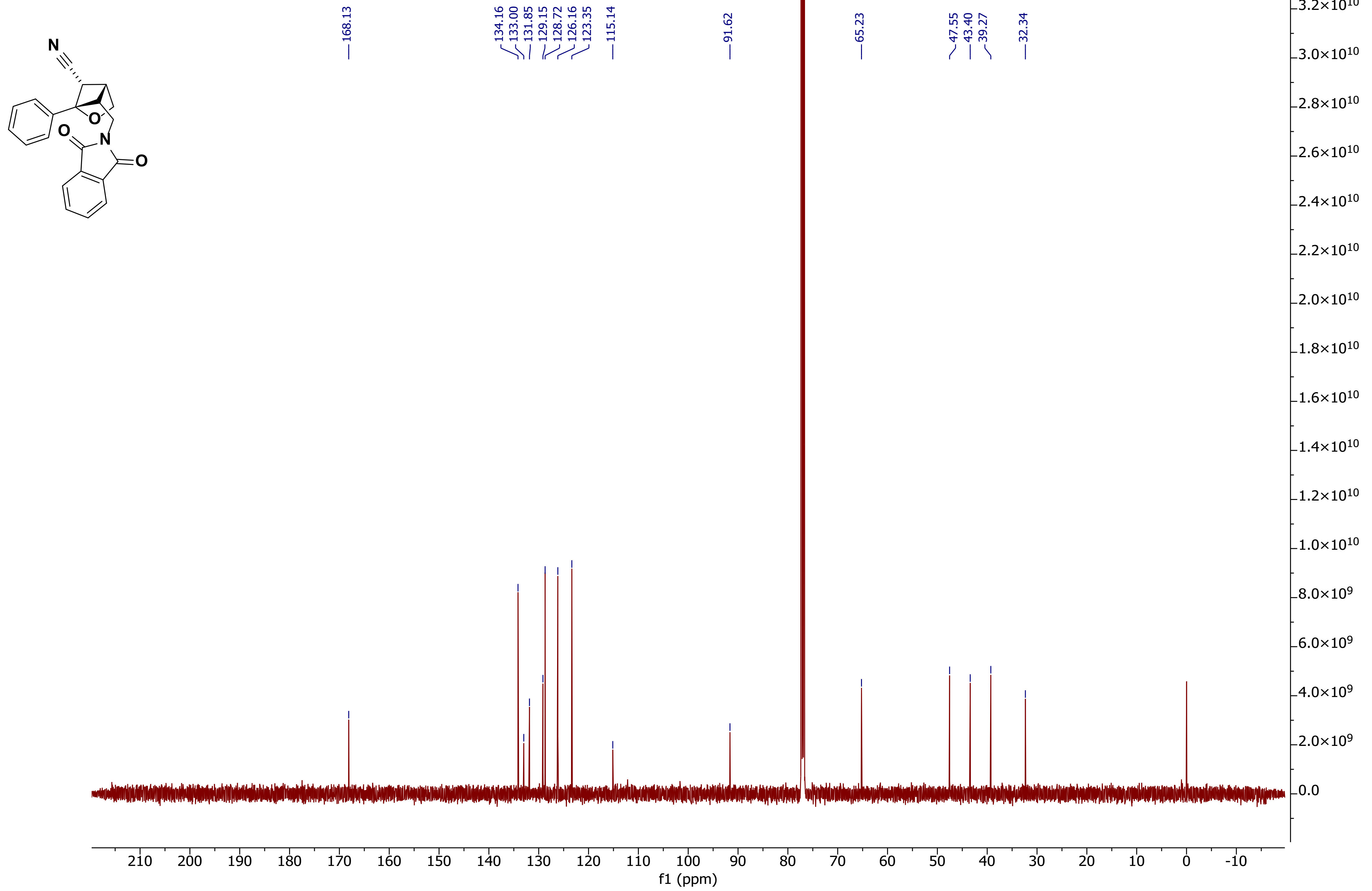
N
O
N
O
O
168.13
134.16
133.00
131.85
129.15
128.72
126.16
123.35
115.14
91.62
65.23
47.55
43.40
39.27
32.34
f1 (ppm)
210 200 190 180 170 160 150 140 130 120 110 100 90 80 70 60 50 40 30 20 10 0 -10
3.2×10^10
3.0×10^10
2.8×10^10
2.6×10^10
2.4×10^10
2.2×10^10
2.0×10^10
1.8×10^10
1.6×10^10
1.4×10^10
1.2×10^10
1.0×10^10
8.0×10^9
6.0×10^9
4.0×10^9
2.0×10^9
0.0

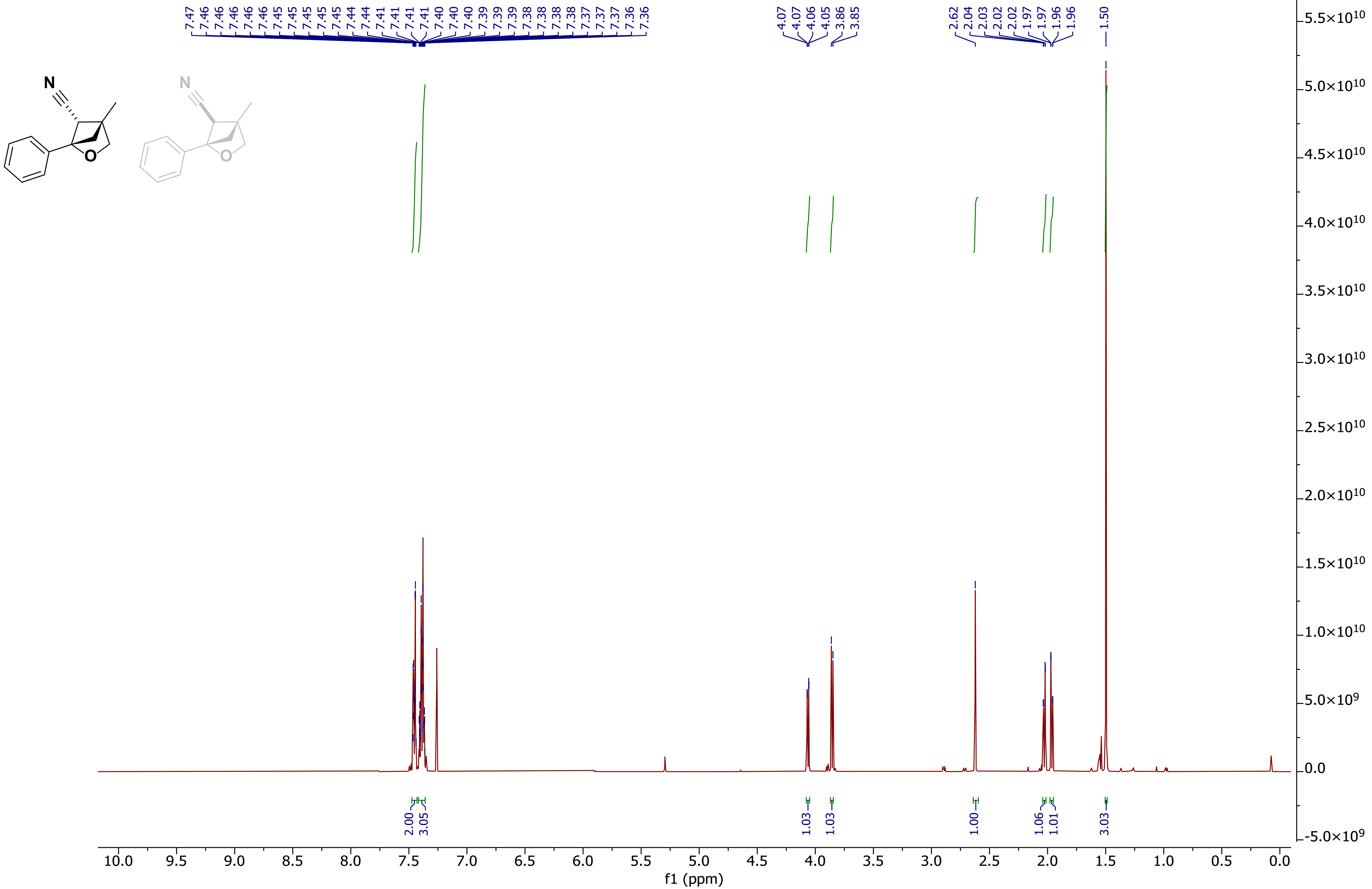

N
O
7.47 7.46 7.46 7.46 7.46 7.45 7.45 7.45 7.45 7.44 7.44 7.41 7.41 7.41 7.41 7.40 7.40 7.39 7.39 7.39 7.38 7.38 7.38 7.38 7.37 7.37 7.37 7.36 7.36
4.07 4.07 4.06 4.05 3.86 3.85
2.62 2.04 2.03 2.02 2.02 1.97 1.97 1.96 1.96
1.50
2.00 3.05 1.03 1.03 1.00 1.06 1.01 3.03
10.0 9.5 9.0 8.5 8.0 7.5 7.0 6.5 6.0 5.5 5.0 4.5 4.0 3.5 3.0 2.5 2.0 1.5 1.0 0.5 0.0
f1 (ppm)
5.5×10^10 5.0×10^10 4.5×10^10 4.0×10^10 3.5×10^10 3.0×10^10 2.5×10^10 2.0×10^10 1.5×10^10 1.0×10^10 5.0×10^9 0.0 -5.0×10^9

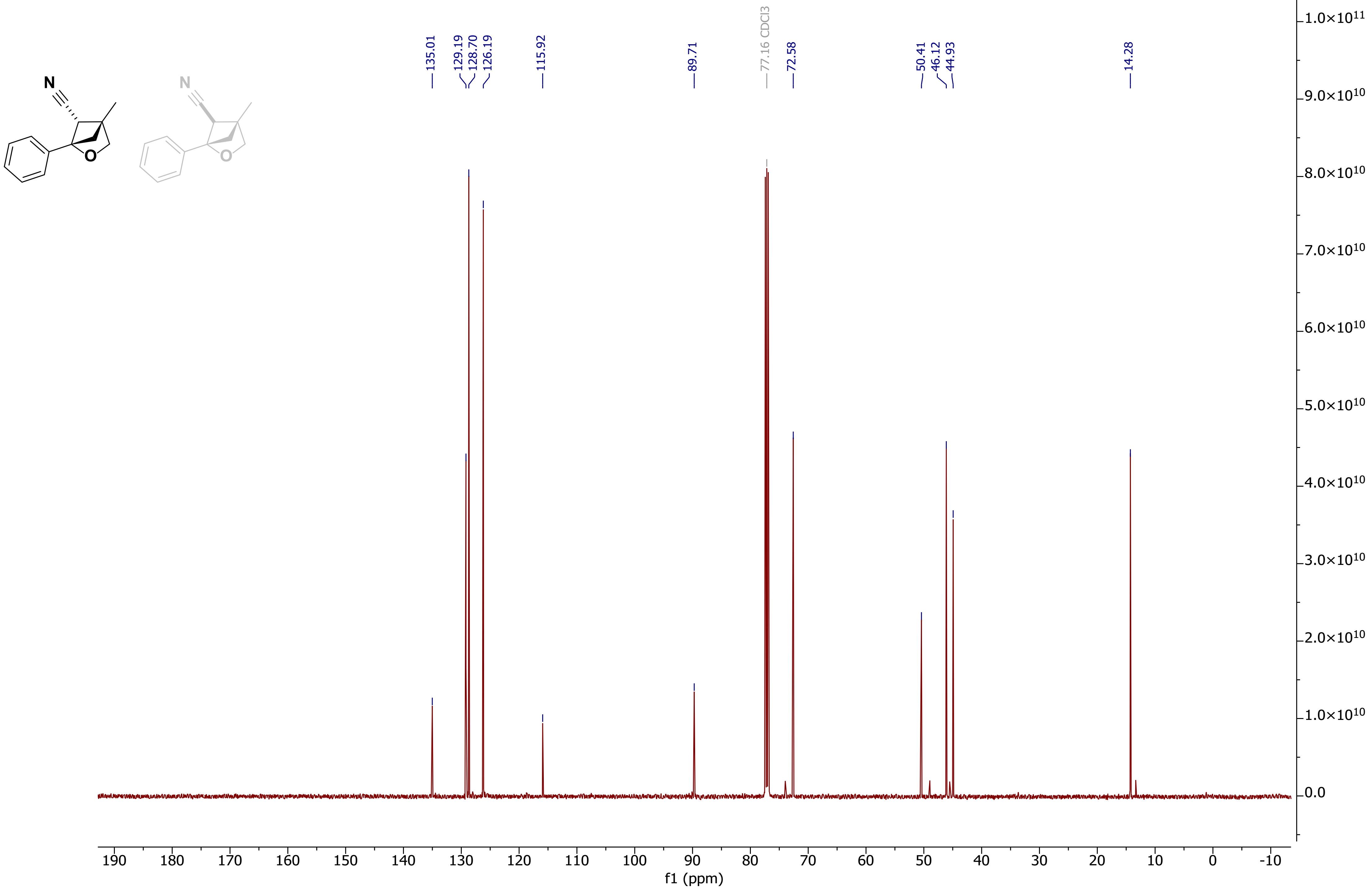

N
O
135.01
129.19
128.70
126.19
115.92
89.71
77.16 CDCl3
72.58
50.41
46.12
44.93
14.28
1.0×10^11
9.0×10^10
8.0×10^10
7.0×10^10
6.0×10^10
5.0×10^10
4.0×10^10
3.0×10^10
2.0×10^10
1.0×10^10
0.0
190 180 170 160 150 140 130 120 110 100 90 80 70 60 50 40 30 20 10 0 -10
f1 (ppm)

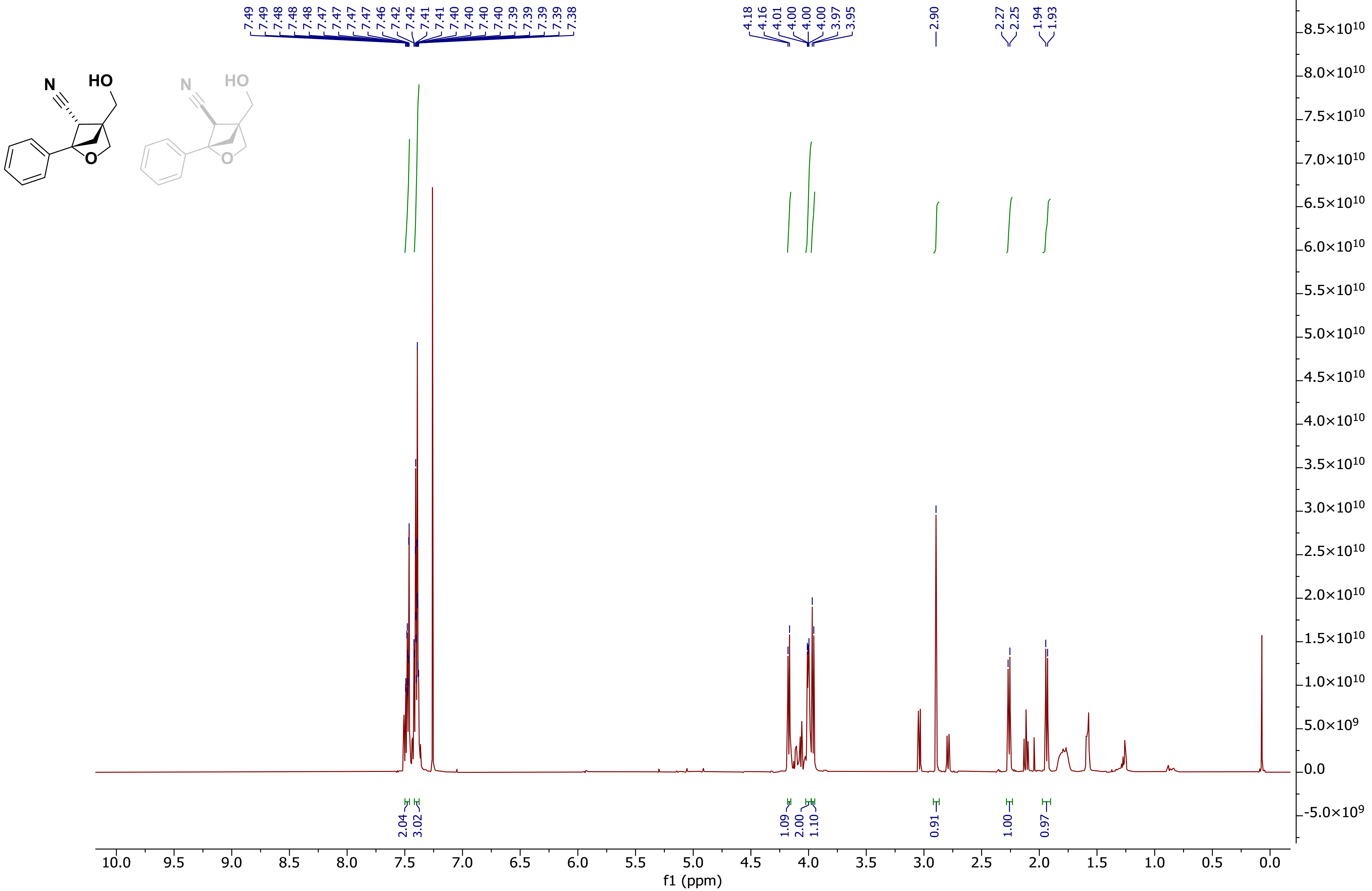

N
HO
O
7.49 7.49 7.48 7.48 7.48 7.47 7.47 7.47 7.47 7.46 7.42 7.42 7.41 7.41 7.40 7.40 7.40 7.40 7.39 7.39 7.39 7.39 7.39 7.38
4.18 4.16 4.01 4.00 4.00 4.00 3.97 3.95
2.90
2.27 2.25
1.94 1.93
2.04 3.02
1.09 2.00 1.10
0.91
1.00
0.97
f1 (ppm)

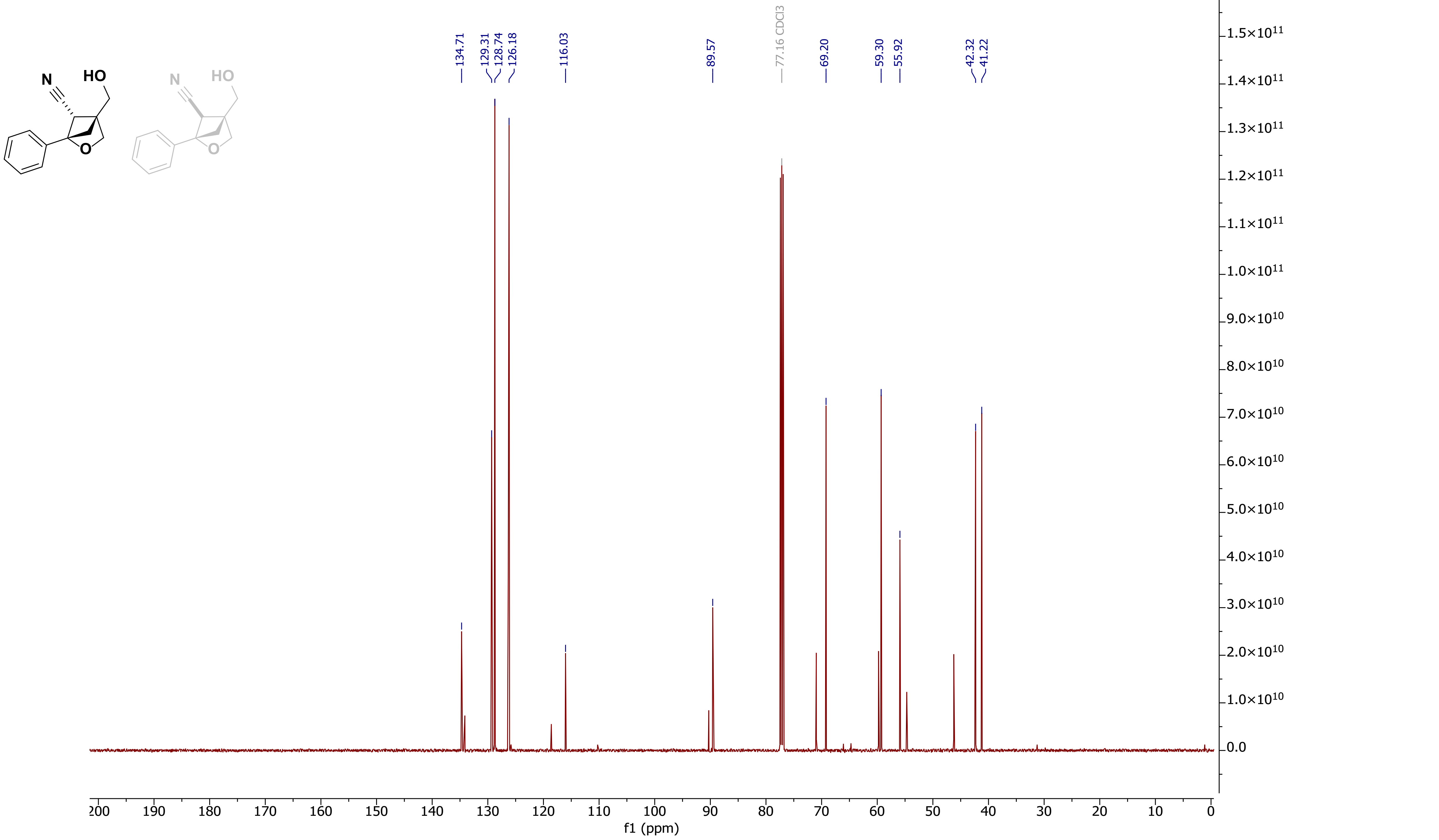

N
HO
134.71
129.31
128.74
126.18
116.03
89.57
77.16 CDCl3
69.20
59.30
55.92
42.32
41.22
f1 (ppm)

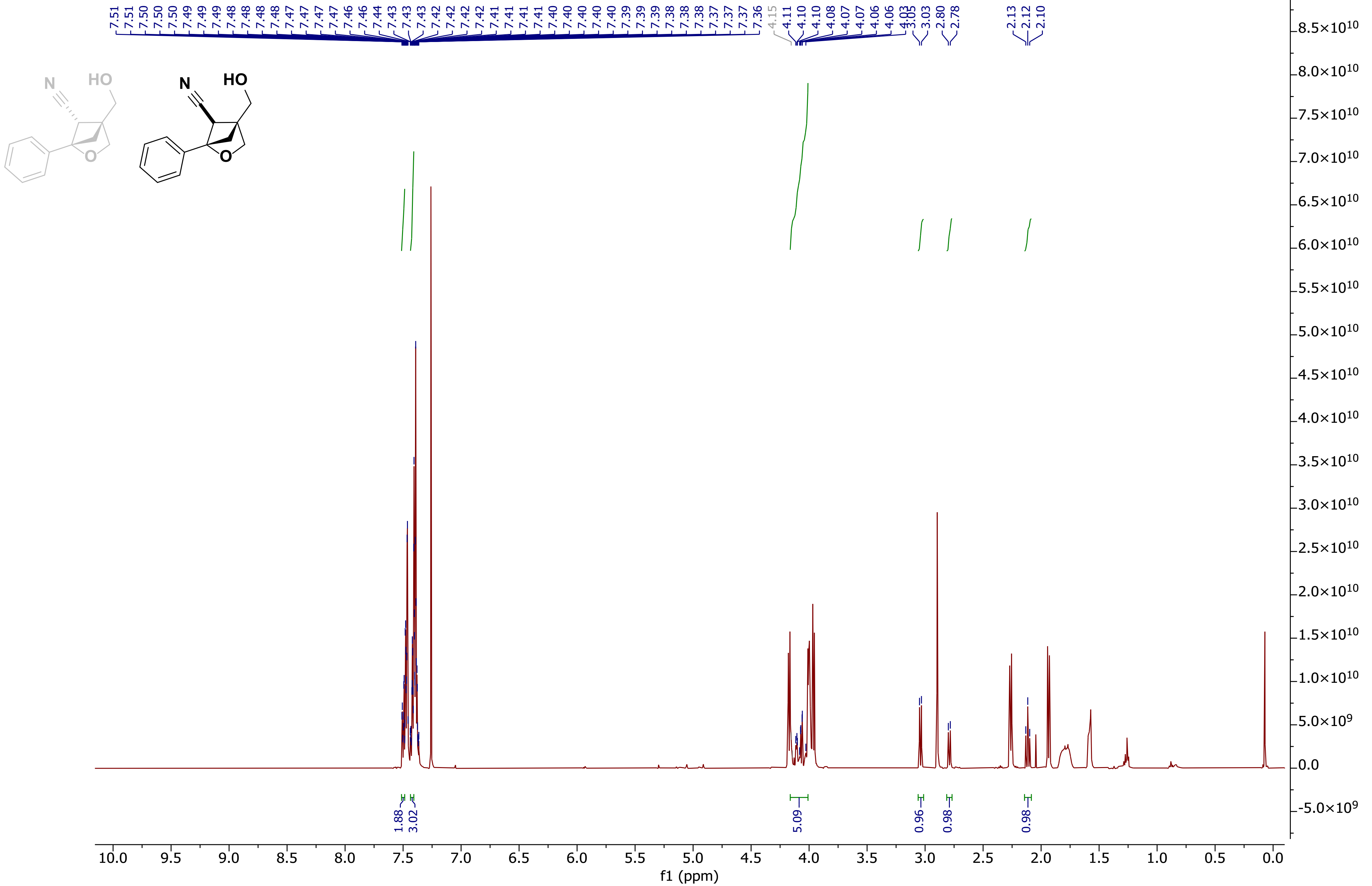

7.51 7.51 7.50 7.50 7.49 7.49 7.49 7.48 7.48 7.48 7.48 7.47 7.47 7.47 7.47 7.46 7.46 7.44 7.43 7.43 7.43 7.42 7.42 7.42 7.42 7.41 7.41 7.41 7.41 7.40 7.40 7.40 7.40 7.39 7.39 7.39 7.38 7.38 7.38 7.37 7.37 7.37 7.36
4.15 4.11 4.10 4.10 4.08 4.07 4.07 4.06 4.06 4.03
3.05 3.03 2.80 2.78
2.13 2.12 2.10
N
HO
O
1.88
3.02
5.09
0.96
0.98
0.98
f1 (ppm)

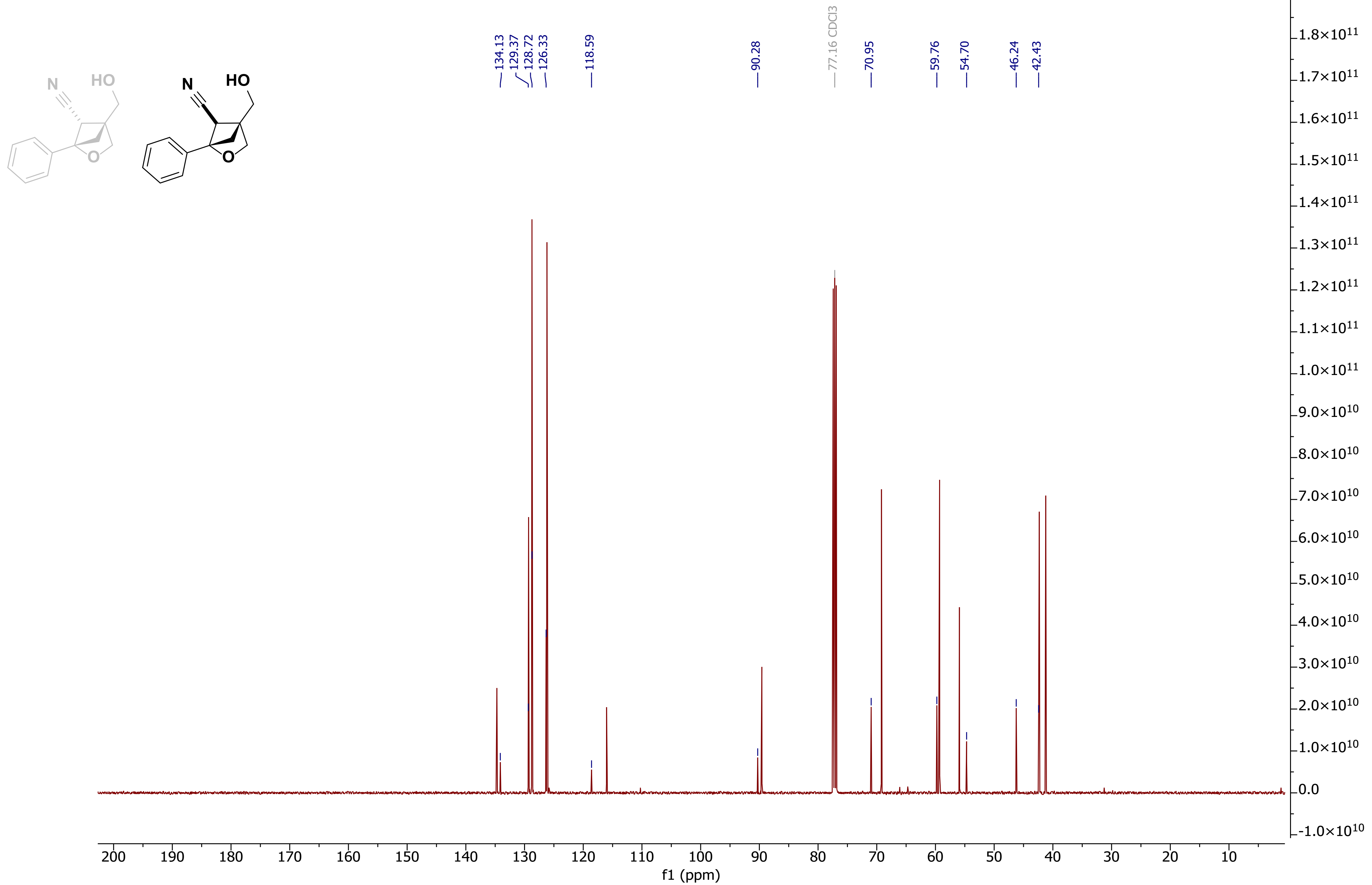

N
HO
O
134.13
129.37
128.72
126.33
118.59
90.28
77.16 CDCl3
70.95
59.76
54.70
46.24
42.43
f1 (ppm)

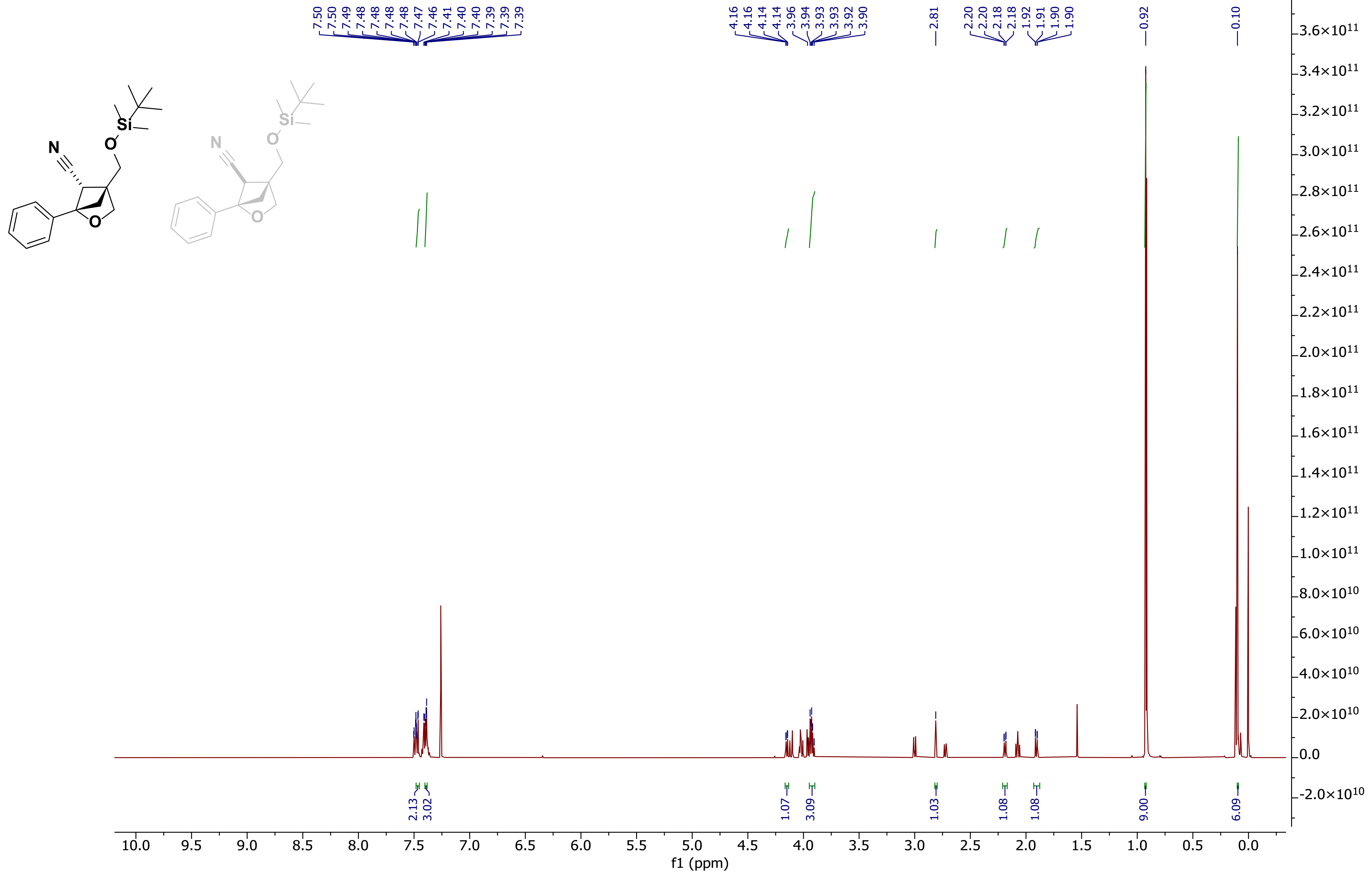

7.50 7.50 7.49 7.48 7.48 7.48 7.48 7.47 7.46 7.41 7.40 7.40 7.39 7.39 7.39
4.16 4.16 4.14 4.14 3.96 3.94 3.93 3.93 3.92 3.90
2.81
2.20 2.20 2.18 2.18 1.92 1.91 1.90 1.90
0.92
0.10
2.13 3.02 1.07 3.09 1.03 1.08 1.08 9.00 6.09
f1 (ppm)

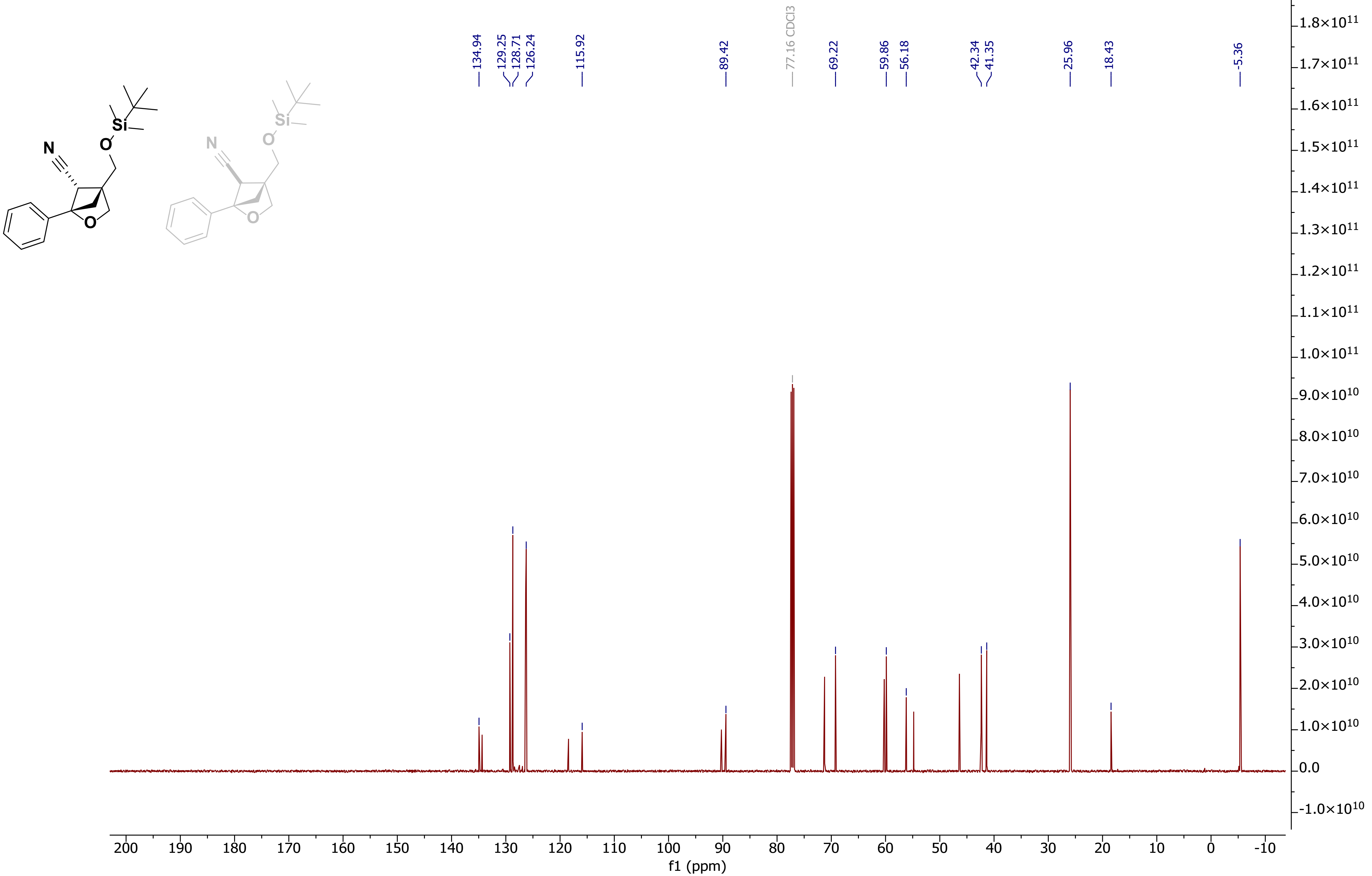
134.94
129.25
128.71
126.24
115.92
89.42
77.16 CDCl3
69.22
59.86
56.18
42.34
41.35
25.96
18.43
-5.36
f1 (ppm)

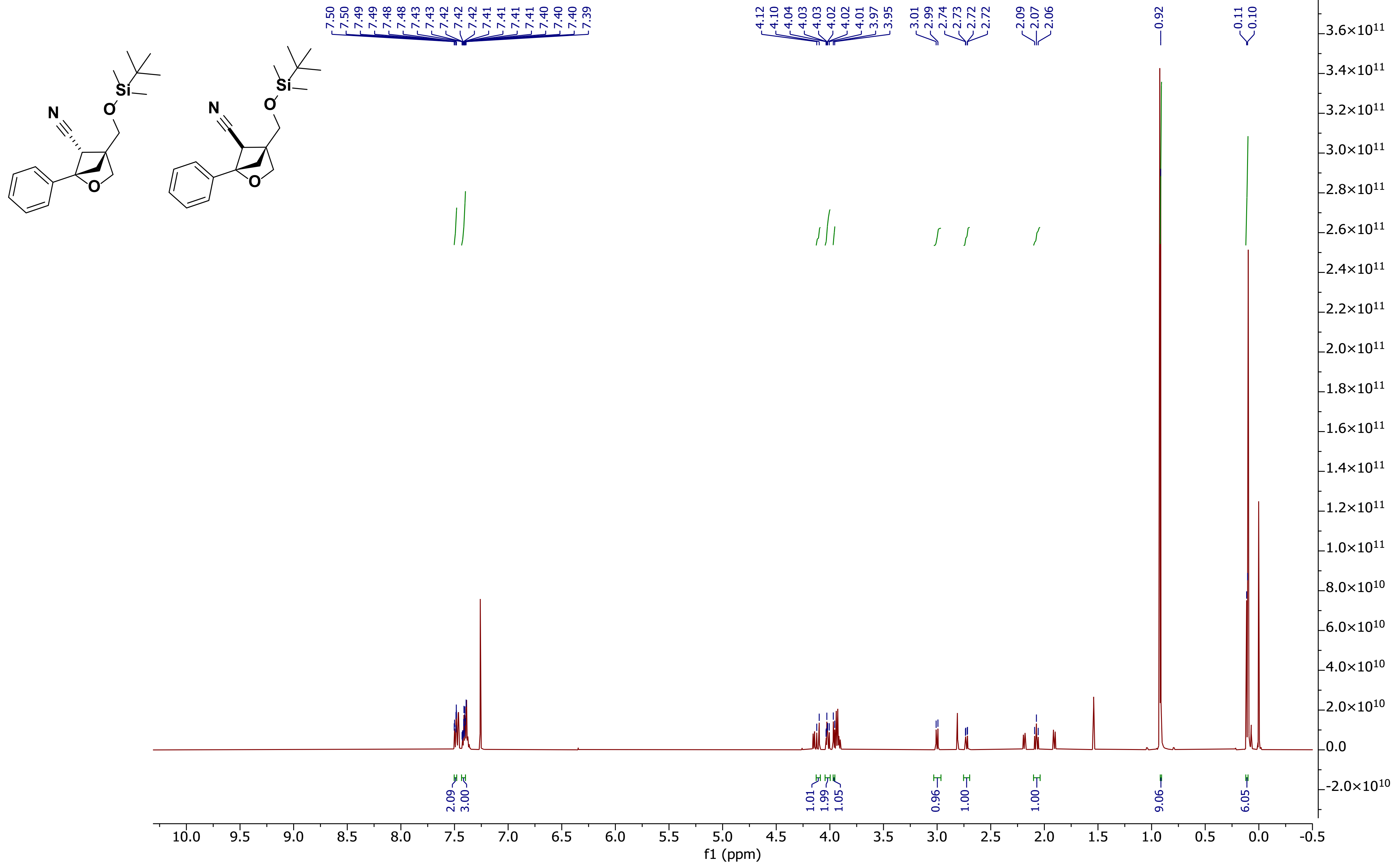

7.50 7.50 7.49 7.49 7.48 7.48 7.43 7.42 7.42 7.42 7.41 7.41 7.41 7.41 7.40 7.40 7.40 7.39
4.12 4.10 4.04 4.03 4.02 4.02 4.01 3.97 3.95
3.01 2.99 2.74 2.73 2.72 2.72
2.09 2.07 2.06
0.92
0.11 0.10
2.09 3.00 1.01 1.99 1.05 0.96 1.00 1.00 9.06 6.05
f1 (ppm)

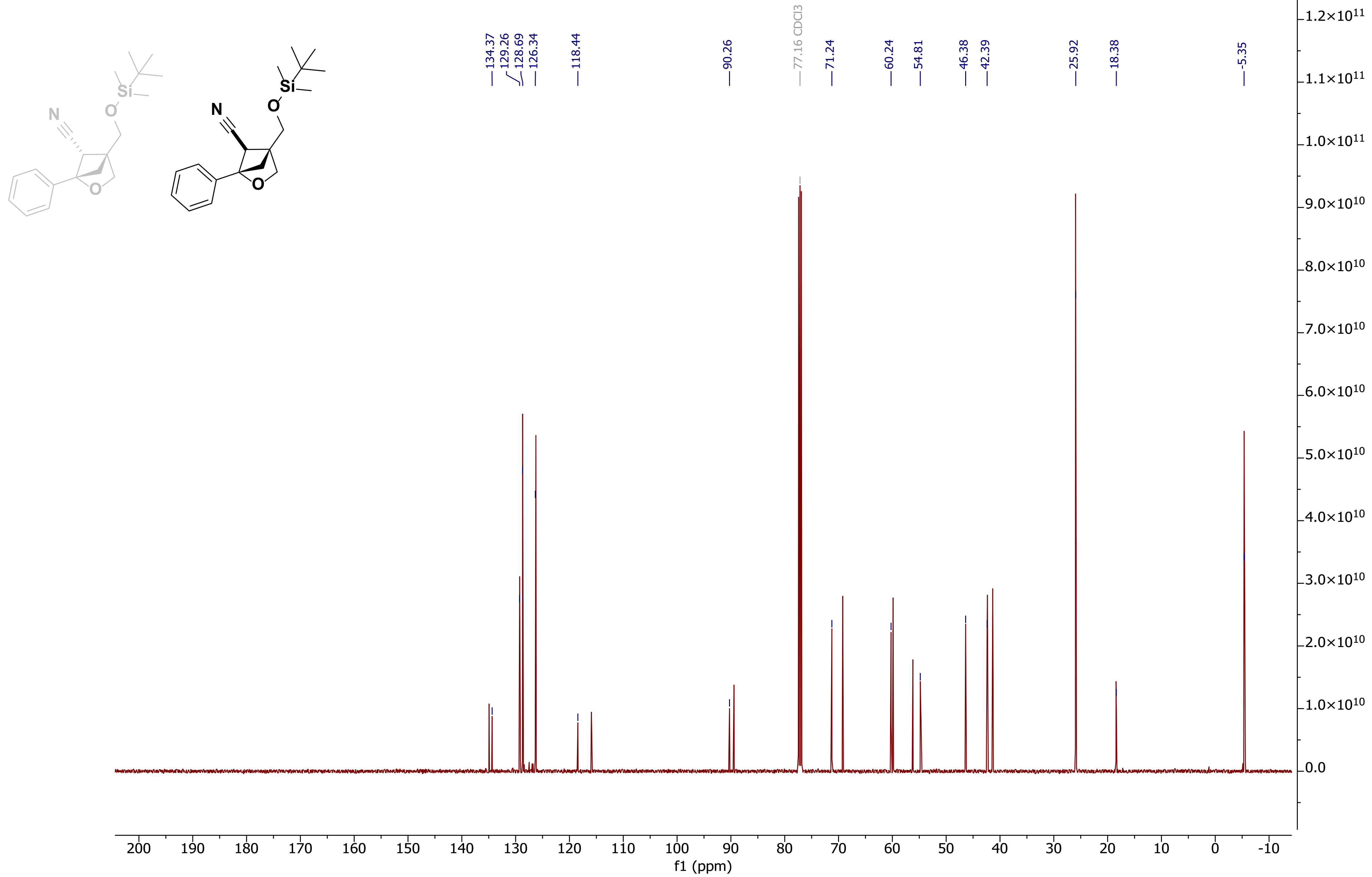

134.37
129.26
128.69
126.34
118.44
90.26
77.16 CDCl3
71.24
60.24
54.81
46.38
42.39
25.92
18.38
-5.35
N
O
Si
f1 (ppm)
200
190
180
170
160
150
140
130
120
110
100
90
80
70
60
50
40
30
20
10
0
-10
1.2×10^11
1.1×10^11
1.0×10^11
9.0×10^10
8.0×10^10
7.0×10^10
6.0×10^10
5.0×10^10
4.0×10^10
3.0×10^10
2.0×10^10
1.0×10^10
0.0

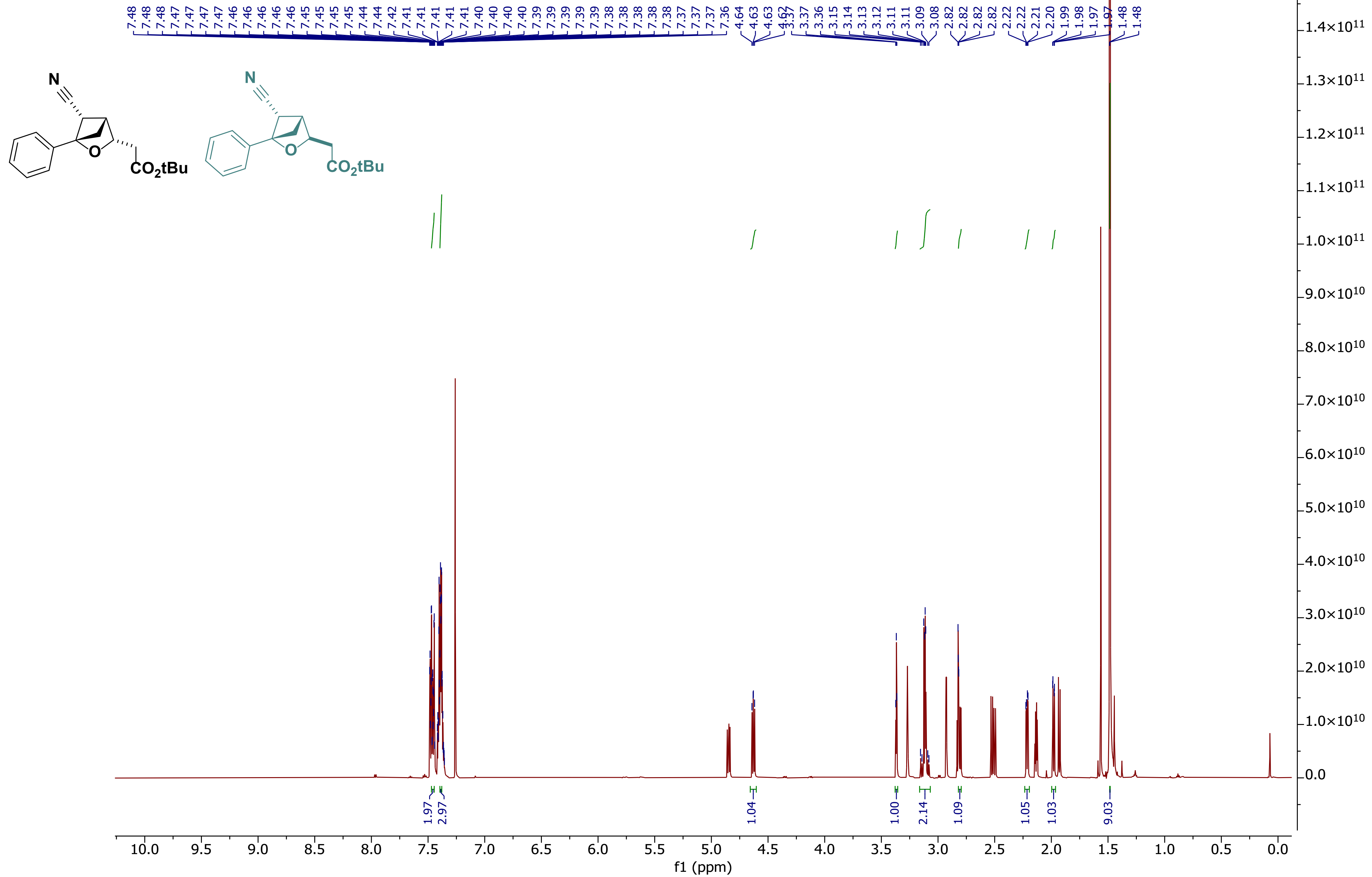

N
O
CO2tBu
N
O
CO2tBu
1.97
2.97
1.04
1.00
2.14
1.09
1.05
1.03
9.03
f1 (ppm)

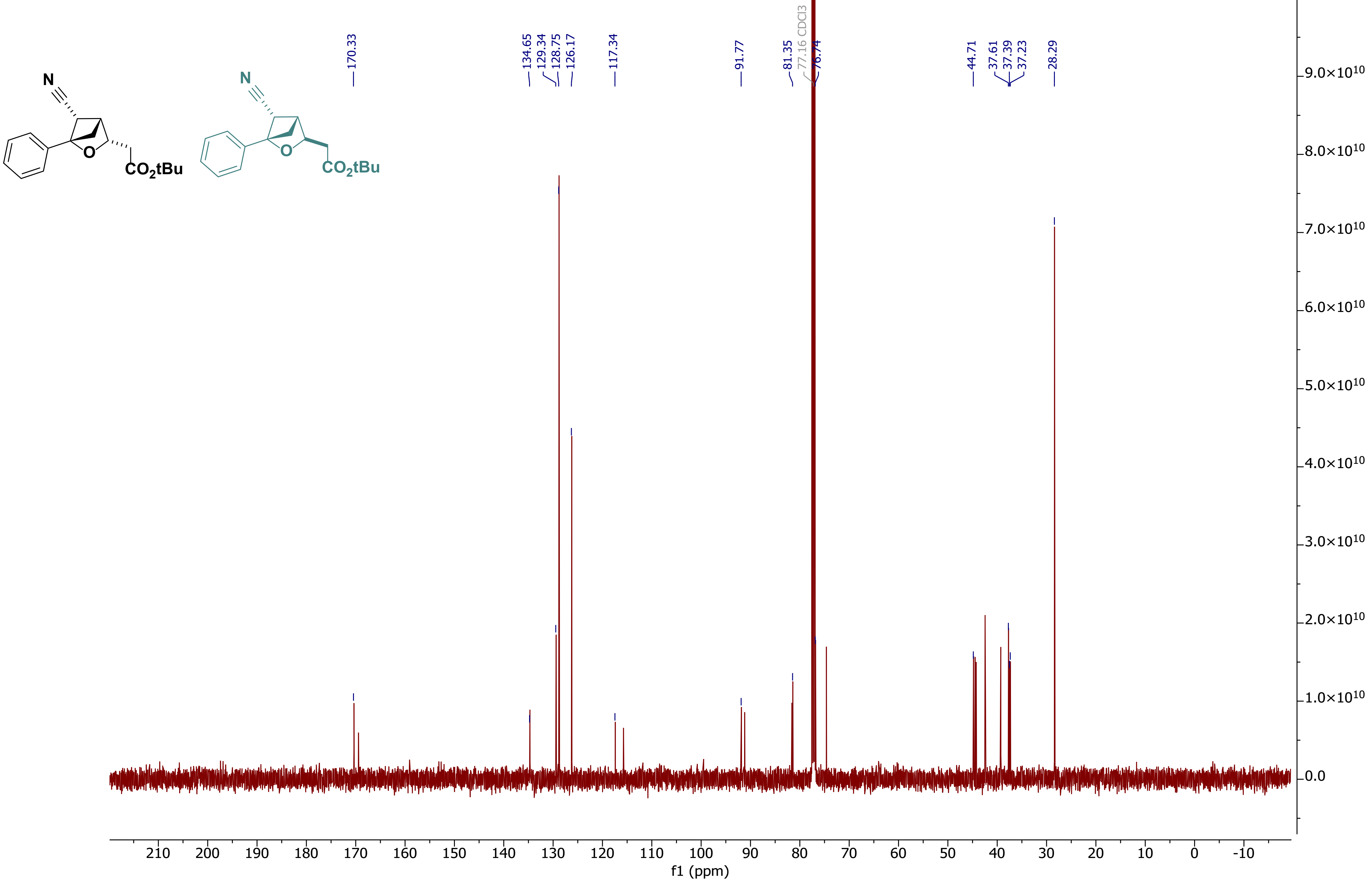
170.33
134.65
129.34
128.75
126.17
117.34
91.77
81.35
77.16 CDCl3
76.74
44.71
37.61
37.39
37.23
28.29
9.0×10^10
8.0×10^10
7.0×10^10
6.0×10^10
5.0×10^10
4.0×10^10
3.0×10^10
2.0×10^10
1.0×10^10
0.0
210
200
190
180
170
160
150
140
130
120
110
100
90
80
70
60
50
40
30
20
10
0
-10
f1 (ppm)

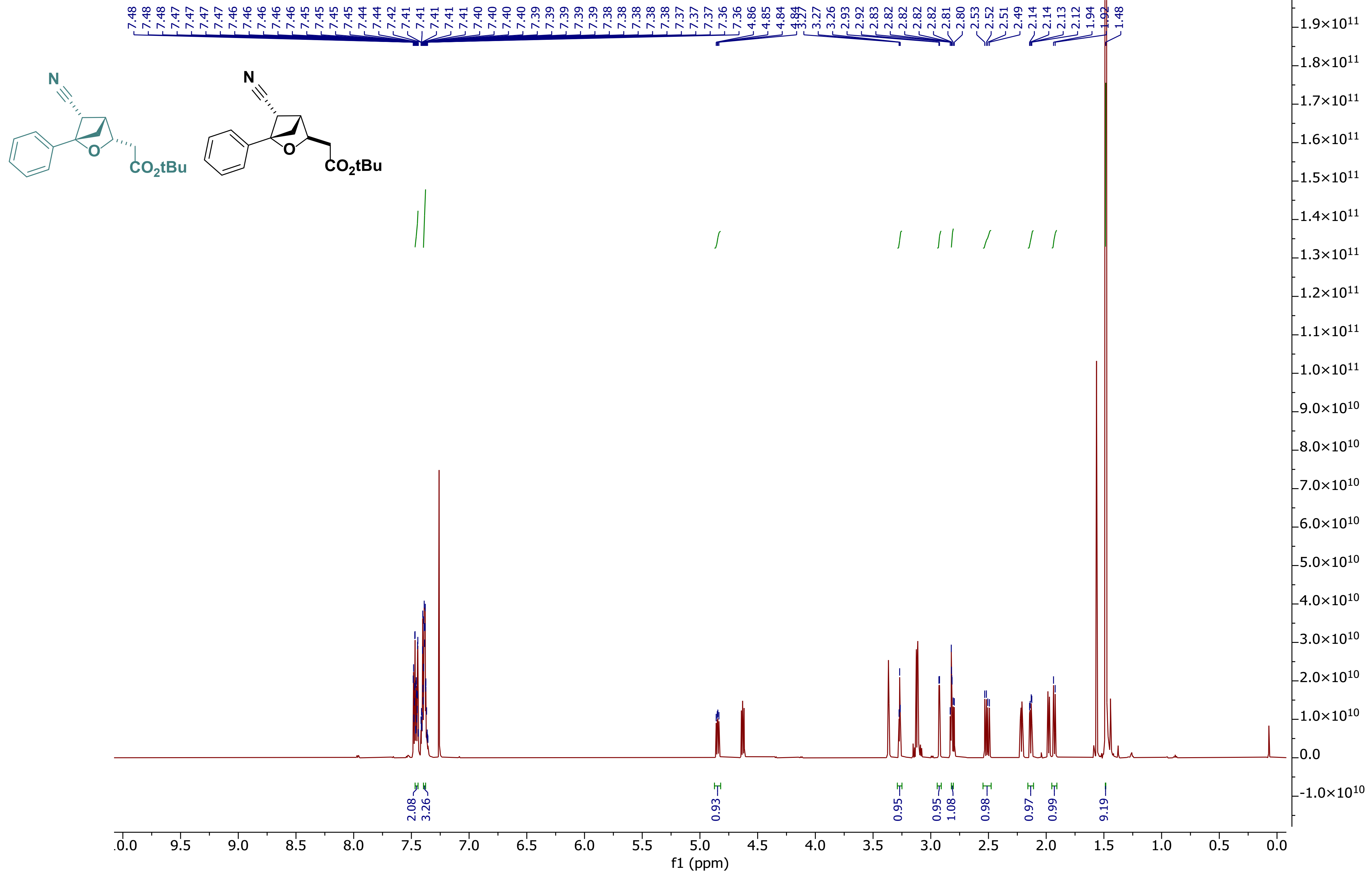

N
CO2tBu
O
N
CO2tBu
O
7.48
7.48
7.48
7.47
7.47
7.47
7.47
7.46
7.46
7.46
7.46
7.45
7.45
7.45
7.44
7.44
7.42
7.41
7.41
7.41
7.41
7.41
7.40
7.40
7.40
7.39
7.39
7.39
7.39
7.39
7.38
7.38
7.38
7.38
7.38
7.37
7.37
7.37
7.36
7.36
4.86
4.85
4.84
4.84
3.27
3.27
3.26
2.93
2.92
2.83
2.82
2.82
2.82
2.82
2.81
2.80
2.53
2.52
2.51
2.49
2.14
2.14
2.13
2.12
1.94
1.92
1.48
2.08
3.26
0.93
0.95
0.95
1.08
0.98
0.97
0.99
9.19
f1 (ppm)

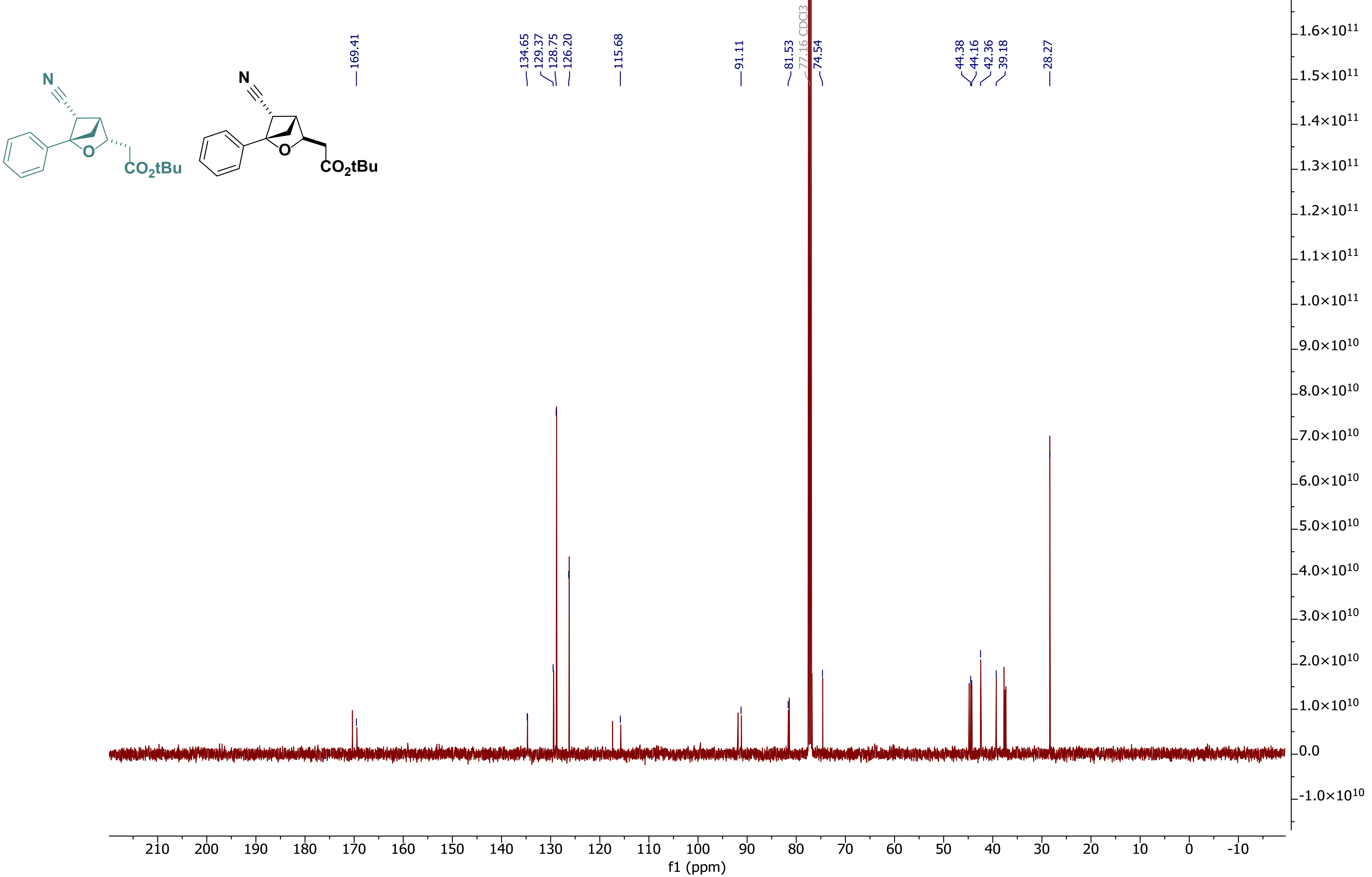
N
O
CO2tBu
N
O
CO2tBu
169.41
134.65
129.37
128.75
126.20
115.68
91.11
81.53
77.16 CDCl3
74.54
44.38
44.16
42.36
39.18
28.27
f1 (ppm)

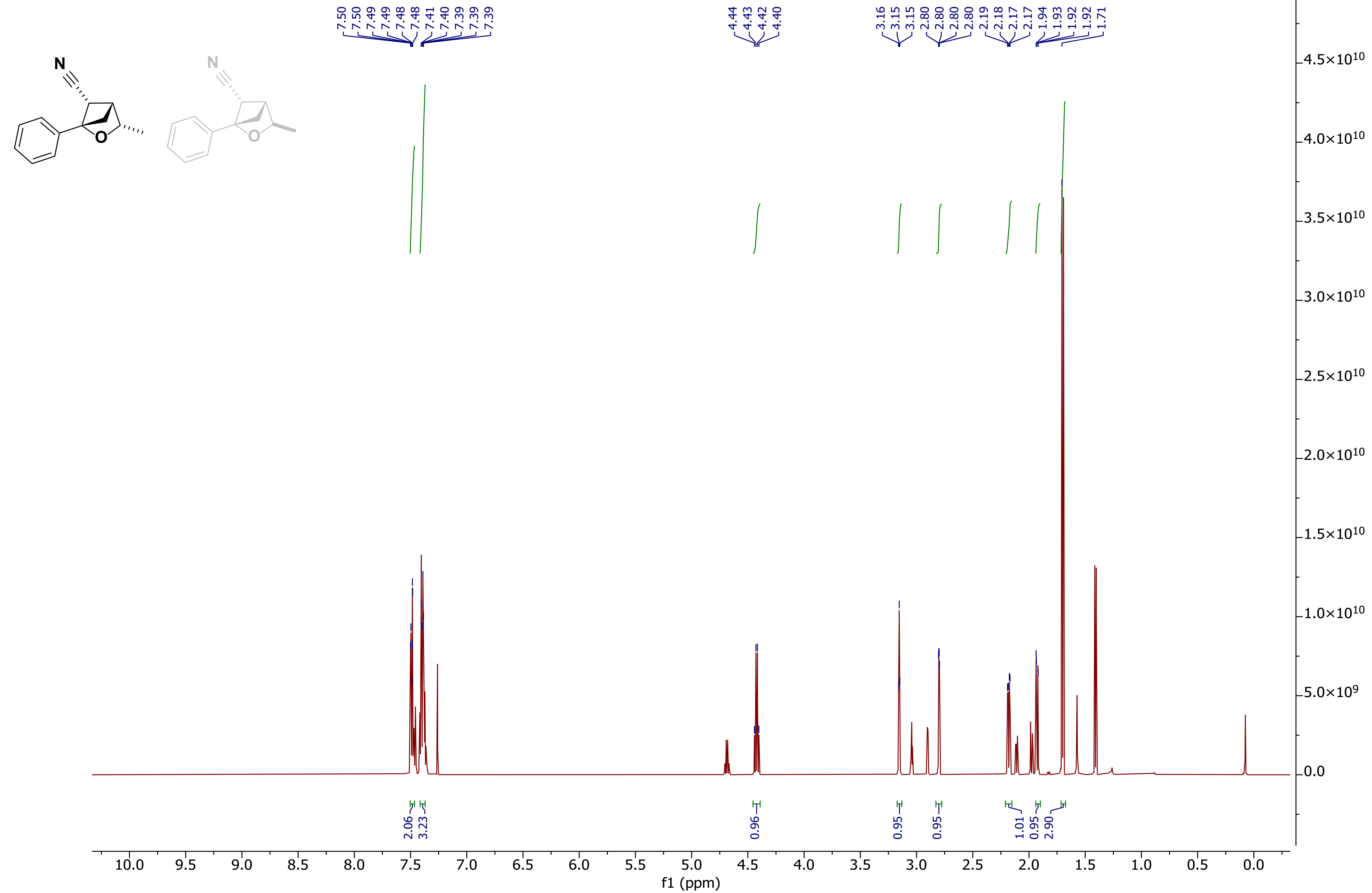

N
O
N
O
7.50
7.50
7.49
7.49
7.48
7.48
7.41
7.40
7.39
7.39
7.39
4.44
4.43
4.42
4.40
3.16
3.15
3.15
2.80
2.80
2.80
2.80
2.19
2.18
2.17
2.17
1.94
1.93
1.92
1.92
1.71
2.06
3.23
0.96
0.95
0.95
1.01
0.95
2.90
f1 (ppm)

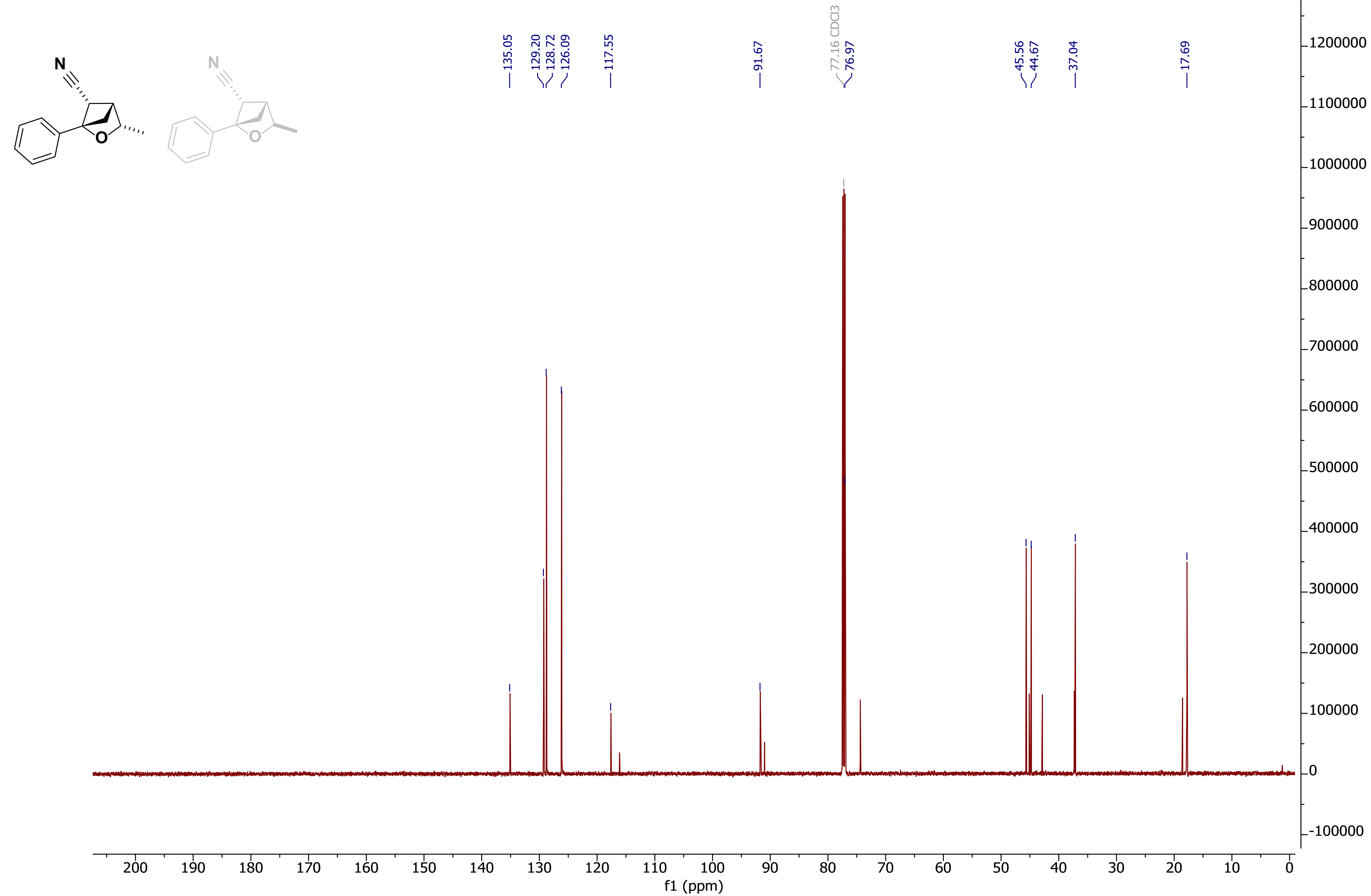
N
O
135.05
129.20
128.72
126.09
117.55
91.67
77.16 CDCl3
76.97
45.56
44.67
37.04
17.69
f1 (ppm)

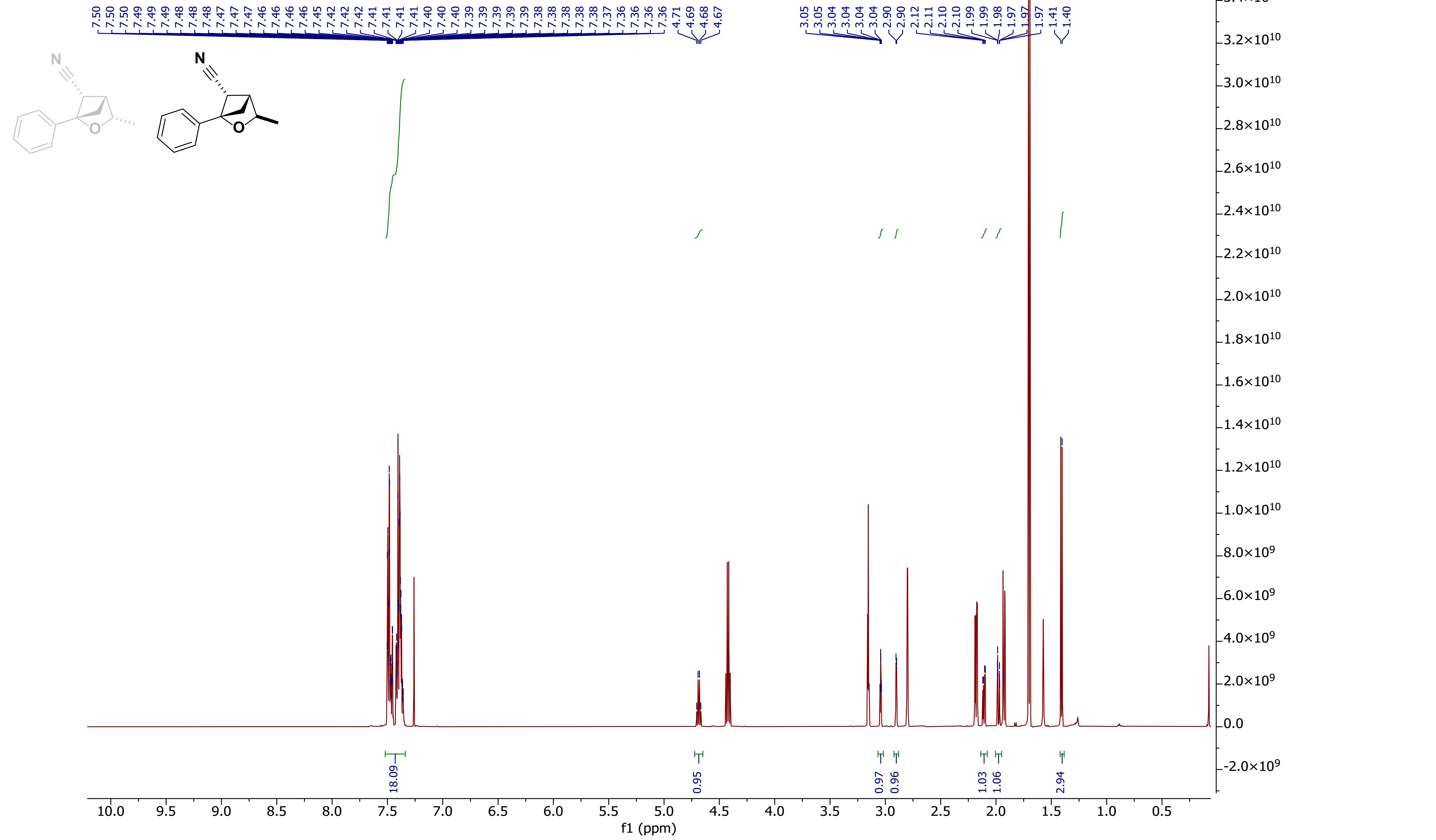

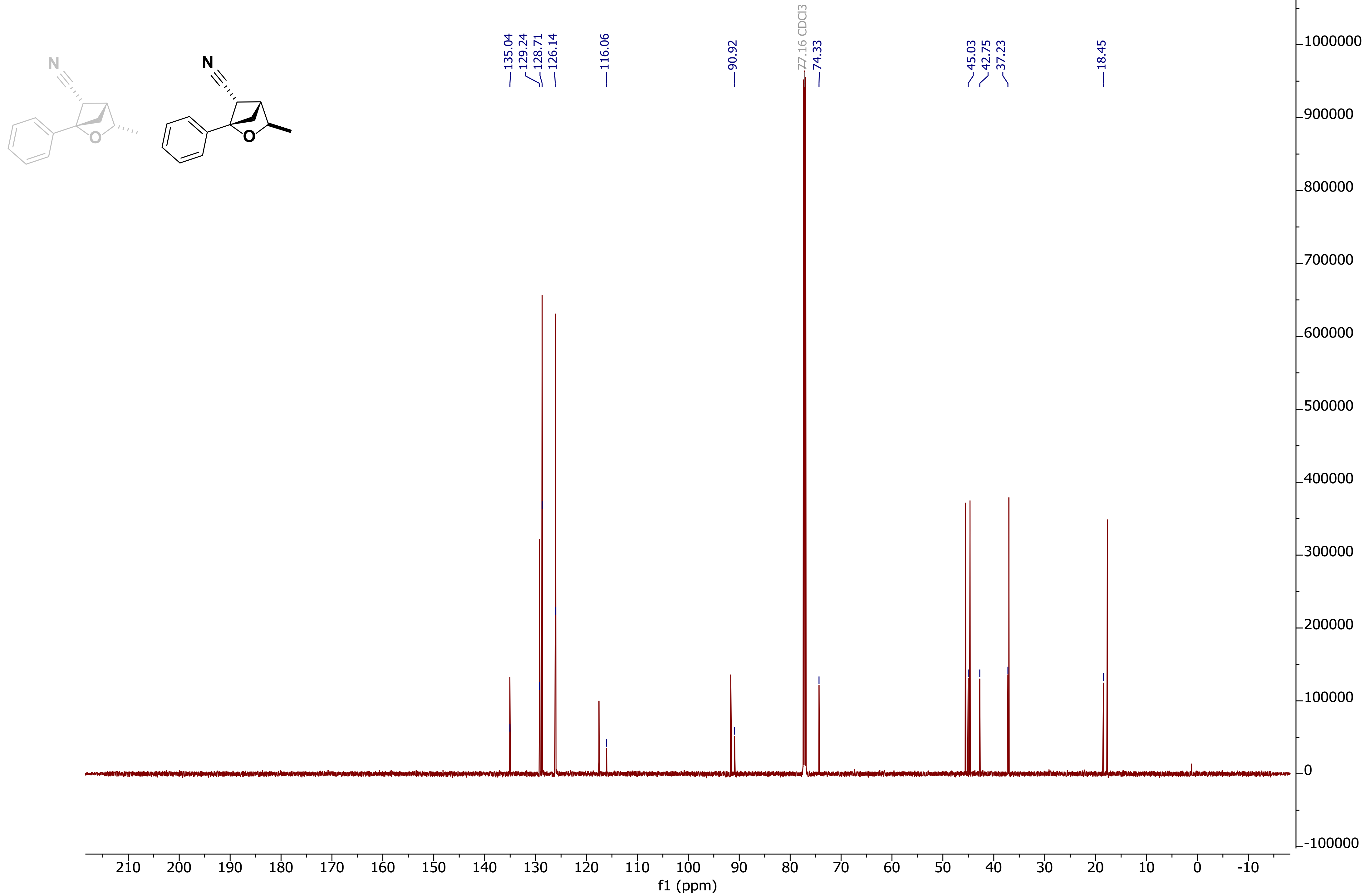
N
O
N
O
135.04
129.24
128.71
126.14
116.06
90.92
77.16 CDCl3
74.33
45.03
42.75
37.23
18.45
1000000
900000
800000
700000
600000
500000
400000
300000
200000
100000
0
-100000
210
200
190
180
170
160
150
140
130
120
110
100
90
80
70
60
50
40
30
20
10
0
-10
f1 (ppm)

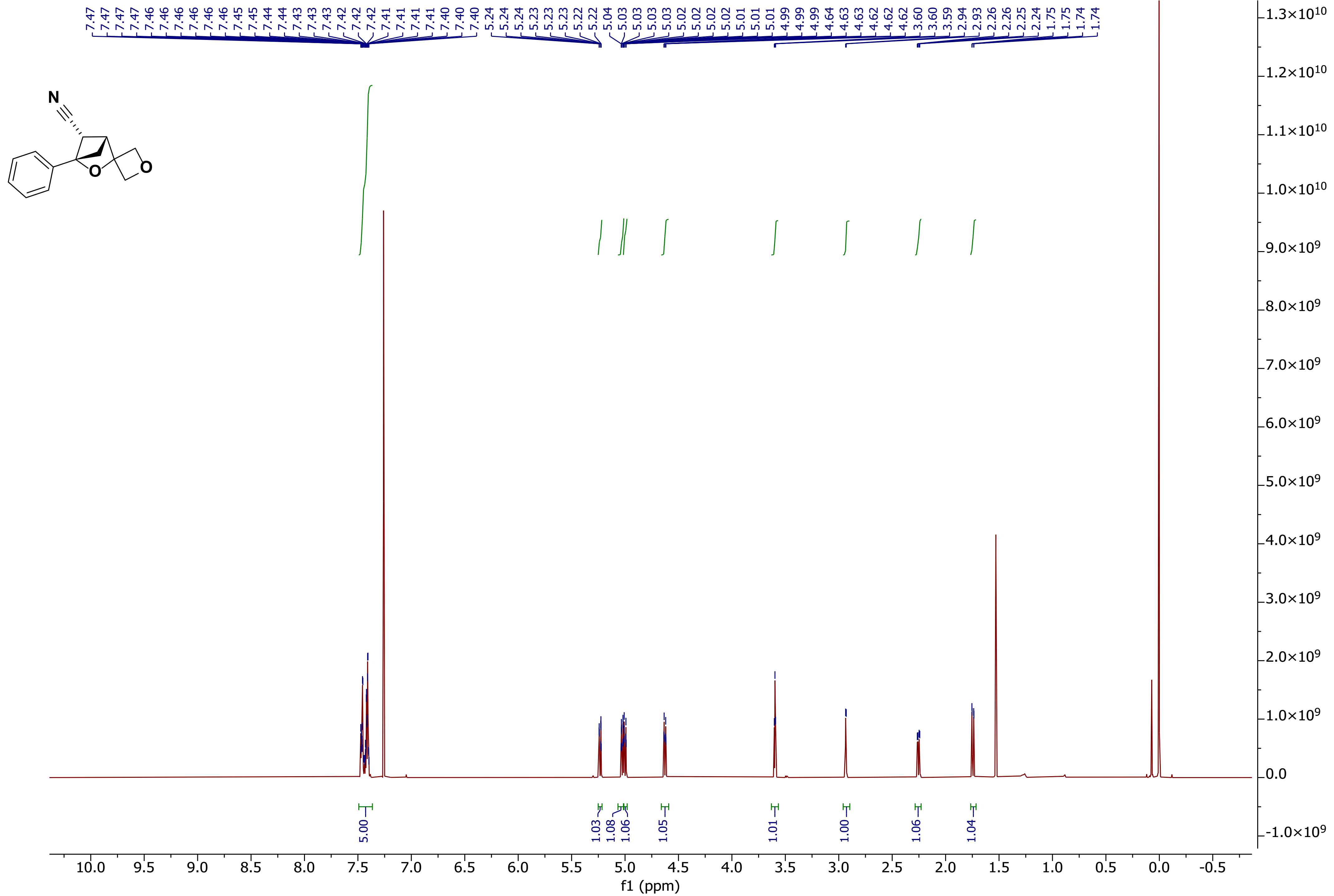

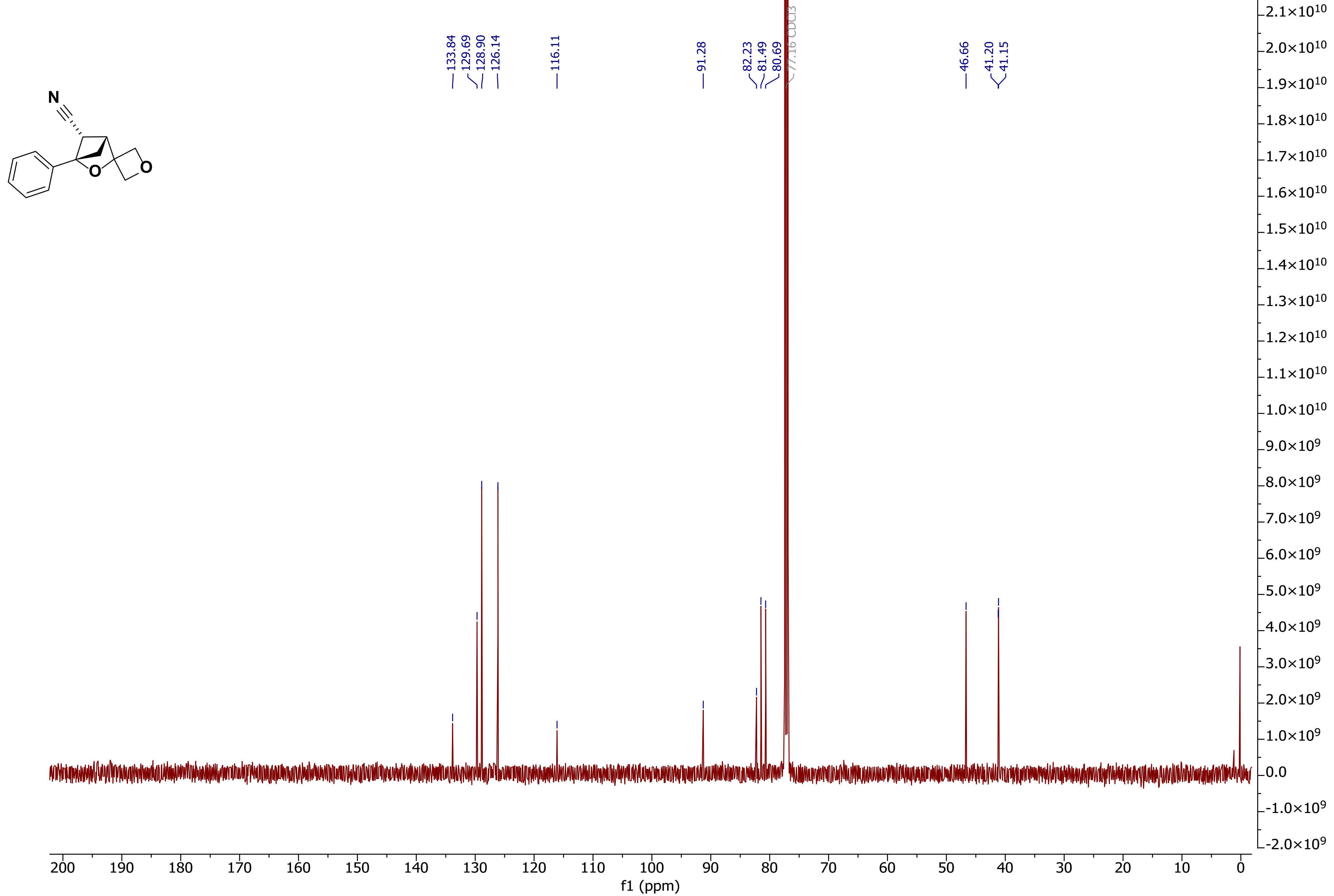
N
O
O
133.84
129.69
128.90
126.14
116.11
91.28
82.23
81.49
80.69
77.16 CDCl3
46.66
41.20
41.15
f1 (ppm)

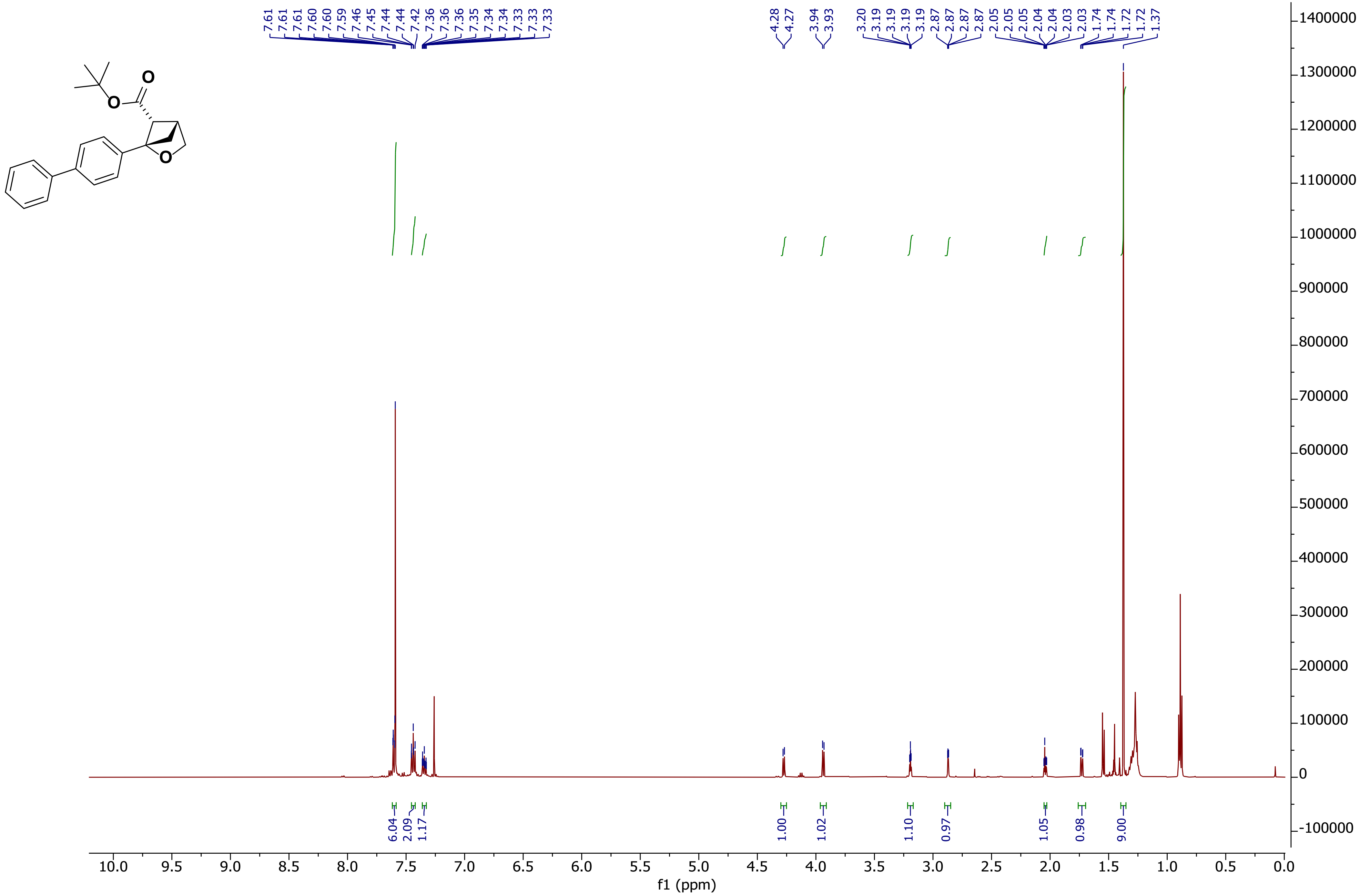
7.61
7.61
7.61
7.60
7.59
7.46
7.45
7.44
7.44
7.42
7.36
7.36
7.36
7.35
7.34
7.34
7.33
7.33
7.33
4.28
4.27
3.94
3.93
3.20
3.19
3.19
3.19
3.19
2.87
2.87
2.87
2.87
2.05
2.05
2.05
2.04
2.04
2.03
2.03
1.74
1.74
1.72
1.72
1.37
6.04
2.09
1.17
1.00
1.02
1.10
0.97
1.05
0.98
9.00
f1 (ppm)

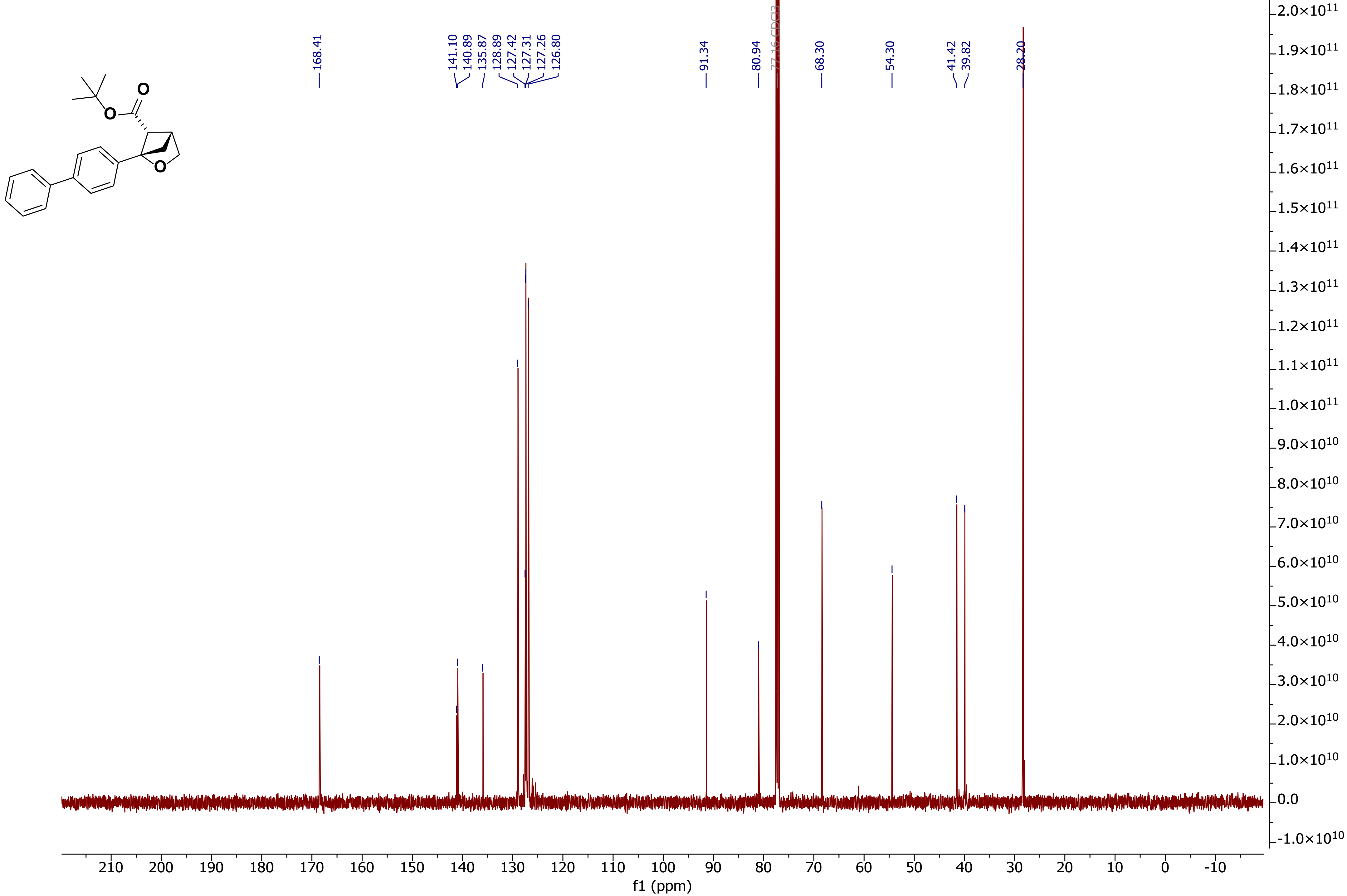

168.41
141.10
140.89
135.87
128.89
127.42
127.31
127.26
126.80
91.34
80.94
77.16 CDCl3
68.30
54.30
41.42
39.82
28.20
f1 (ppm)

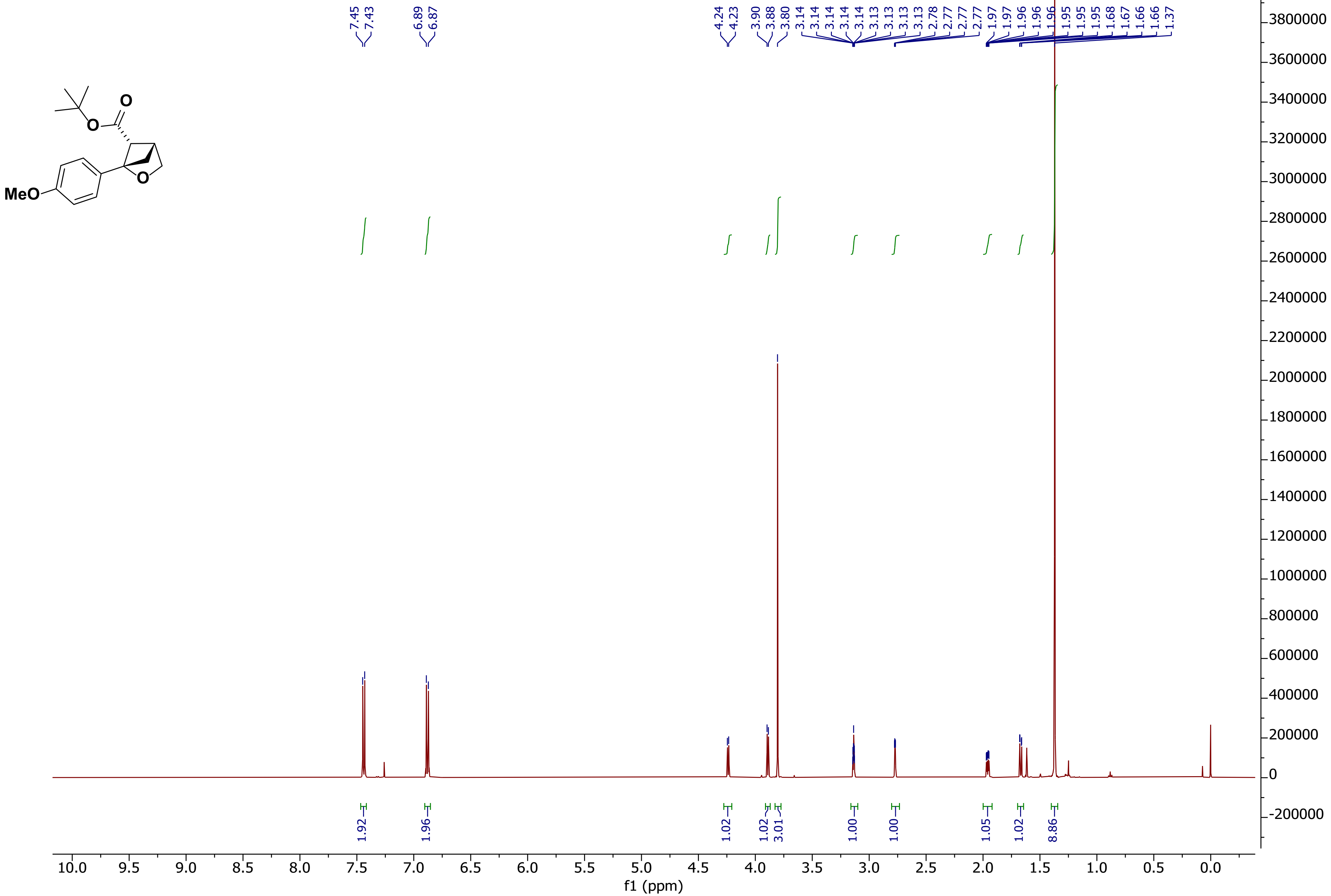
O
O
O
MeO
7.45
7.43
6.89
6.87
4.24
4.23
3.90
3.88
3.80
3.14
3.14
3.14
3.14
3.14
3.13
3.13
3.13
3.13
2.78
2.77
2.77
2.77
1.97
1.97
1.96
1.96
1.96
1.95
1.95
1.95
1.68
1.67
1.66
1.66
1.37
1.92
1.96
1.02
1.02
3.01
1.00
1.00
1.05
1.02
8.86
10.0
9.5
9.0
8.5
8.0
7.5
7.0
6.5
6.0
5.5
5.0
4.5
4.0
3.5
3.0
2.5
2.0
1.5
1.0
0.5
0.0
f1 (ppm)
3800000
3600000
3400000
3200000
3000000
2800000
2600000
2400000
2200000
2000000
1800000
1600000
1400000
1200000
1000000
800000
600000
400000
200000
0
-200000

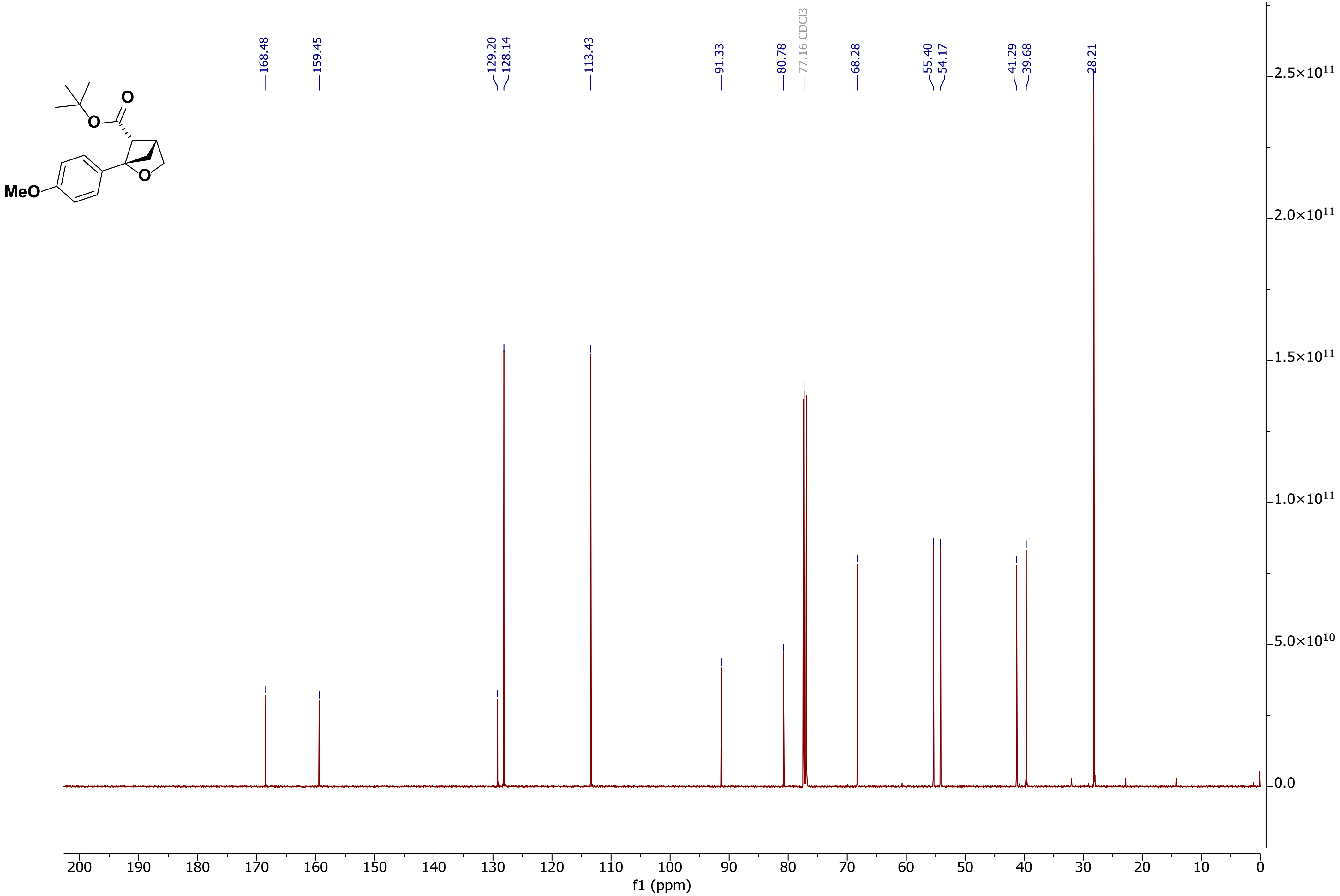

O
O
O
MeO
168.48
159.45
129.20
128.14
113.43
91.33
80.78
77.16 CDCl3
68.28
55.40
54.17
41.29
39.68
28.21
2.5×10^11
2.0×10^11
1.5×10^11
1.0×10^11
5.0×10^10
0.0
200 190 180 170 160 150 140 130 120 110 100 90 80 70 60 50 40 30 20 10 0
f1 (ppm)

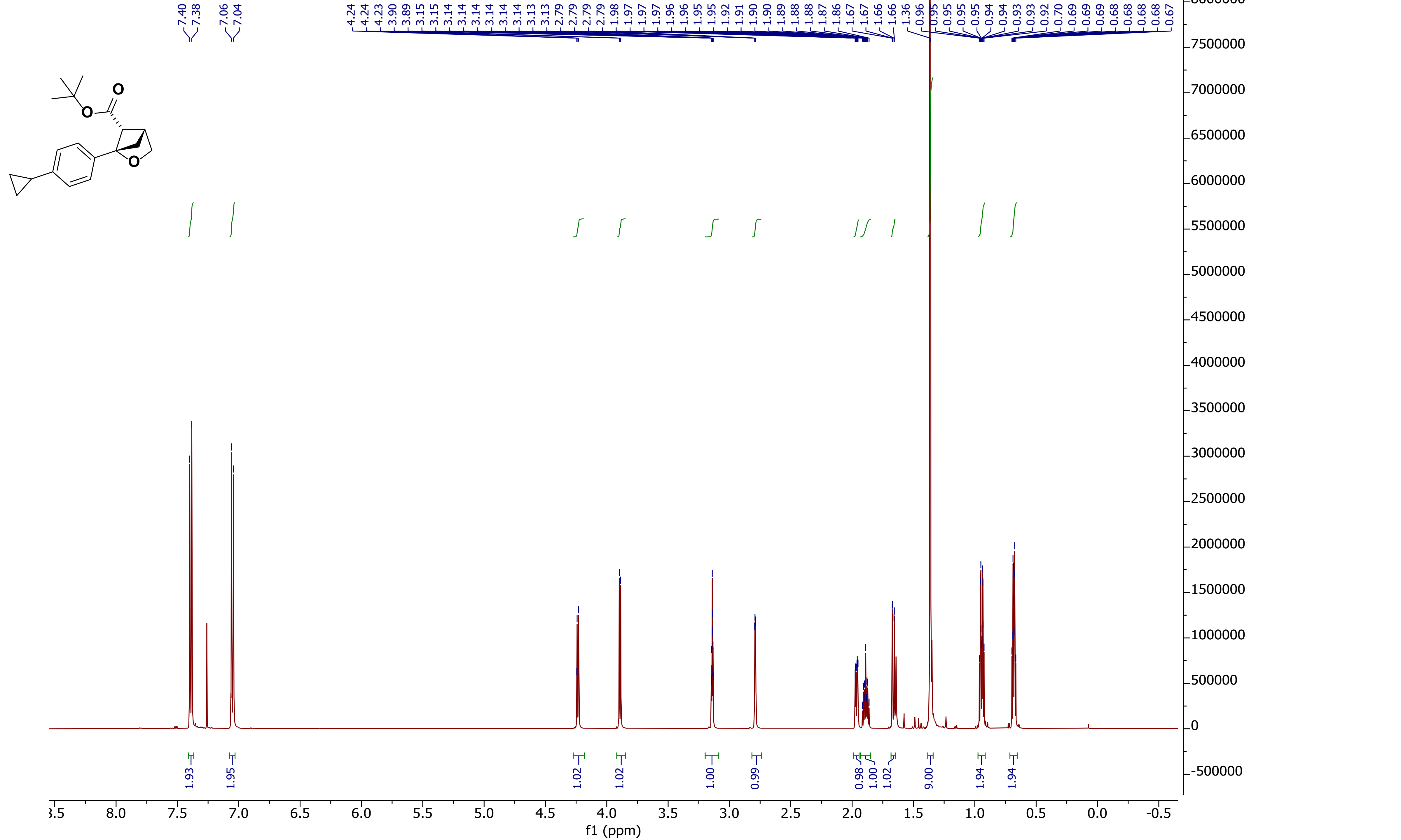

O
O
O
7.40
7.38
7.06
7.04
4.24
4.24
4.23
3.90
3.89
3.15
3.15
3.14
3.14
3.14
3.14
3.14
3.13
3.13
2.79
2.79
2.79
1.98
1.97
1.97
1.97
1.96
1.96
1.95
1.95
1.92
1.91
1.90
1.90
1.89
1.88
1.88
1.87
1.86
1.67
1.67
1.66
1.66
1.36
0.96
0.95
0.95
0.95
0.95
0.94
0.94
0.93
0.93
0.92
0.70
0.69
0.69
0.68
0.68
0.68
0.67
1.93
1.95
1.02
1.02
1.00
0.99
0.98
1.00
1.02
9.00
1.94
1.94
7500000
7000000
6500000
6000000
5500000
5000000
4500000
4000000
3500000
3000000
2500000
2000000
1500000
1000000
500000
0
-500000
8.0
7.5
7.0
6.5
6.0
5.5
5.0
4.5
4.0
3.5
3.0
2.5
2.0
1.5
1.0
0.5
0.0
-0.5
f1 (ppm)

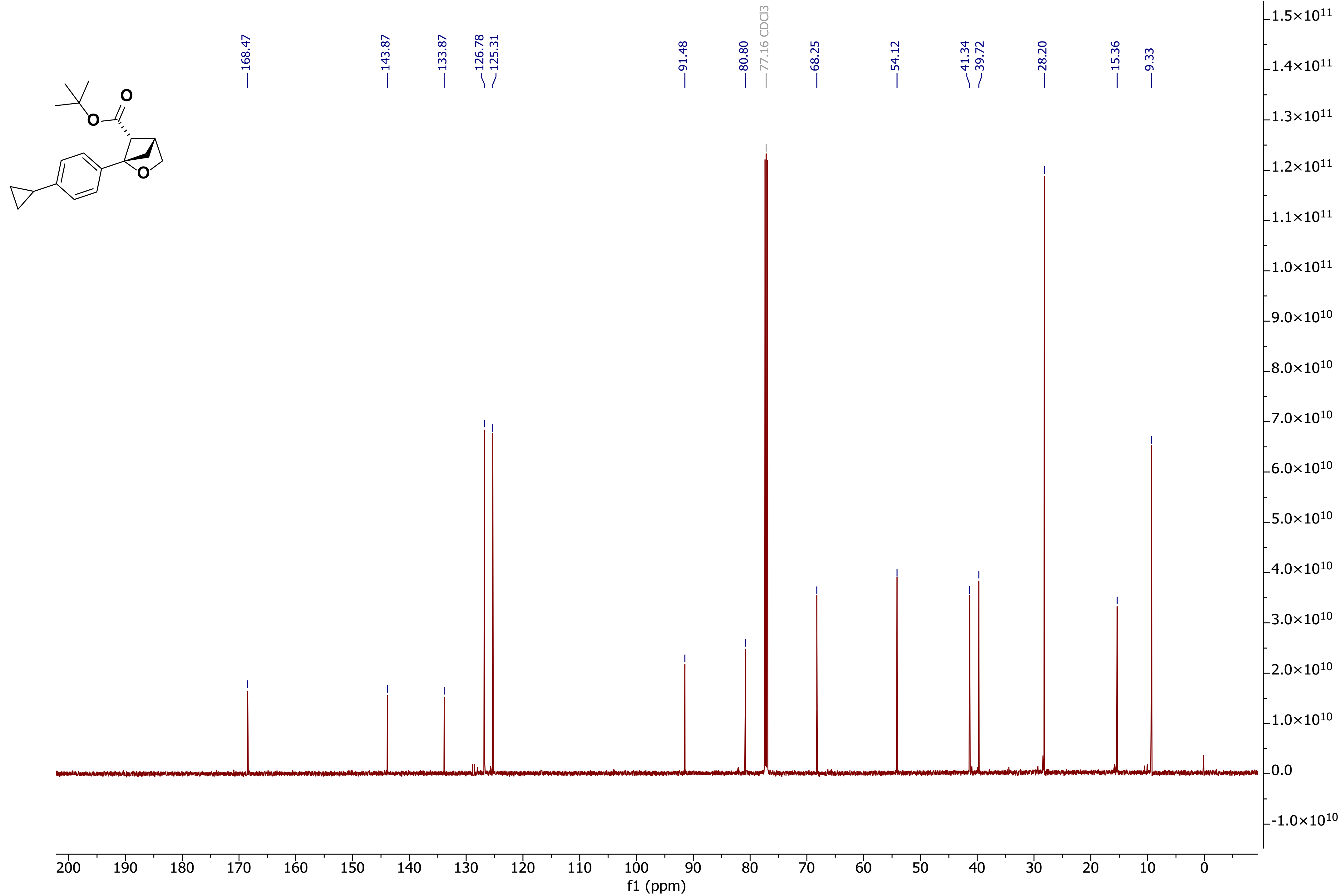

168.47
143.87
133.87
126.78
125.31
91.48
80.80
77.16 CDCl3
68.25
54.12
41.34
39.72
28.20
15.36
9.33
f1 (ppm)

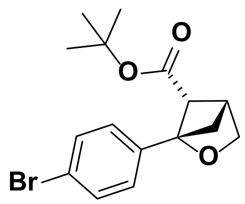
O
O
O
Br

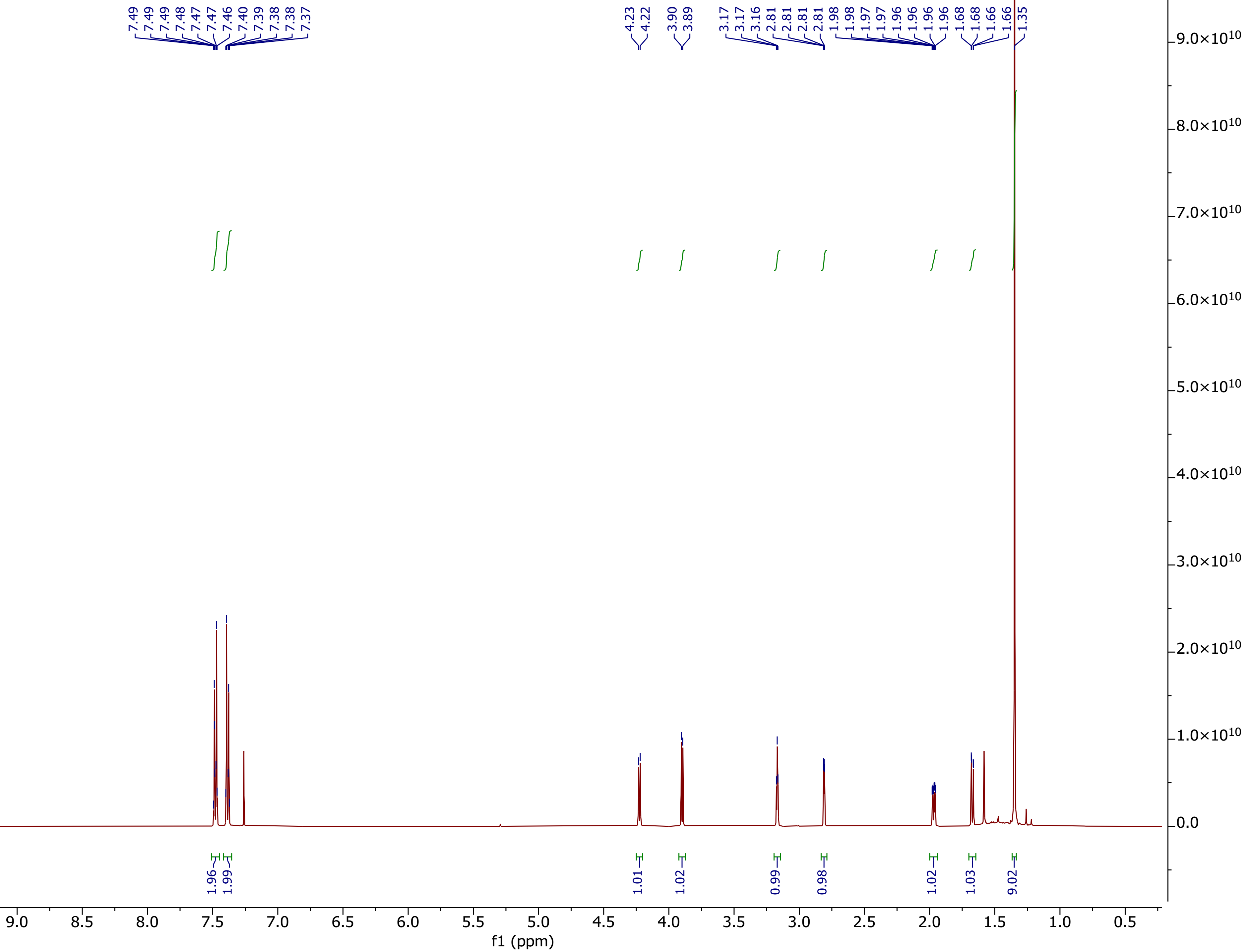
7.49
7.49
7.49
7.48
7.47
7.47
7.46
7.40
7.39
7.38
7.38
7.37
4.23
4.22
3.90
3.89
3.17
3.17
3.16
2.81
2.81
2.81
2.81
1.98
1.98
1.97
1.97
1.96
1.96
1.96
1.96
1.68
1.68
1.66
1.66
1.35
1.96
1.99
1.01
1.02
0.99
0.98
1.02
1.03
9.02
f1 (ppm)

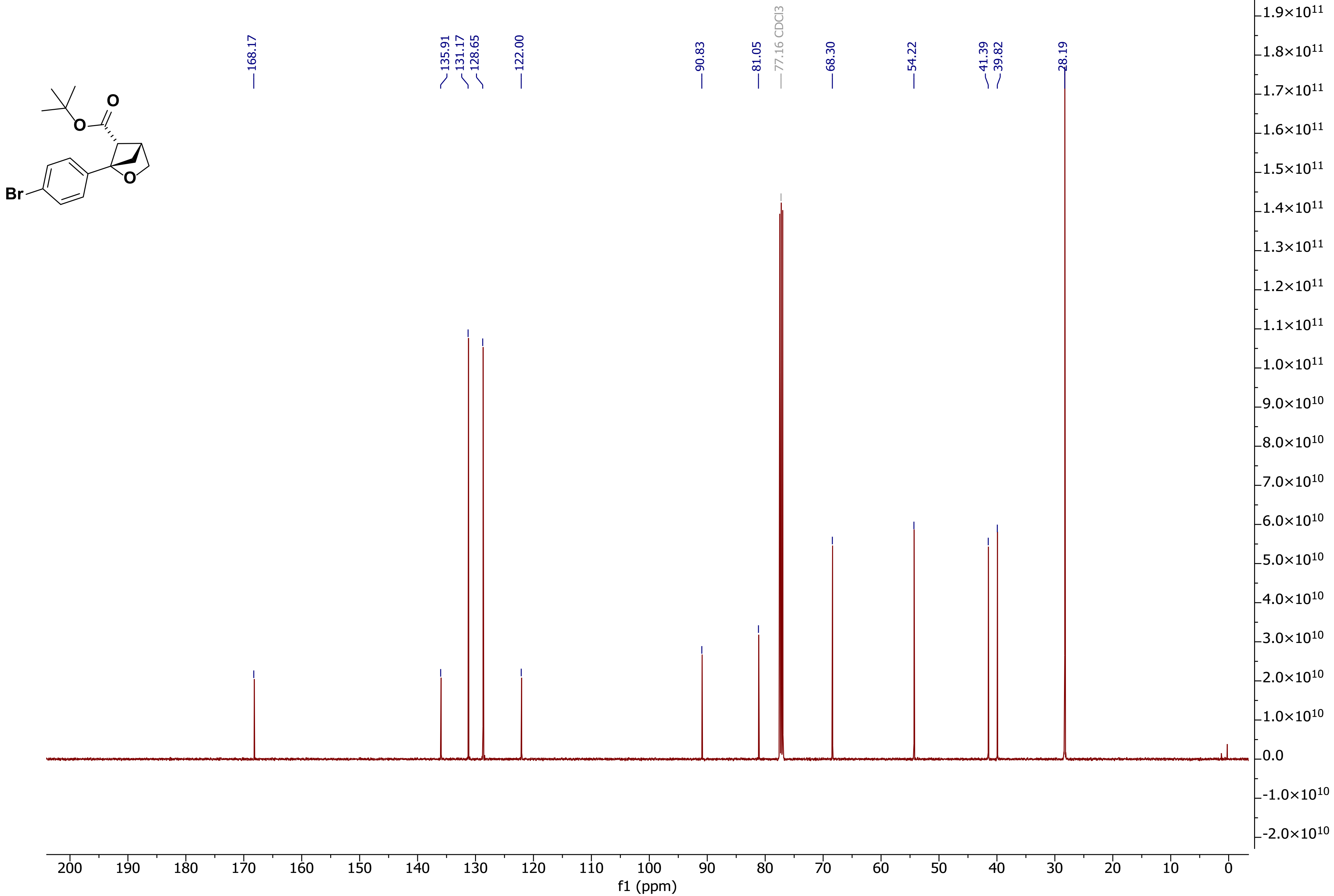

168.17
135.91
131.17
128.65
122.00
90.83
81.05
77.16 CDCl3
68.30
54.22
41.39
39.82
28.19
Br
O
O
O
f1 (ppm)
200 190 180 170 160 150 140 130 120 110 100 90 80 70 60 50 40 30 20 10 0
1.9×10^11
1.8×10^11
1.7×10^11
1.6×10^11
1.5×10^11
1.4×10^11
1.3×10^11
1.2×10^11
1.1×10^11
1.0×10^11
9.0×10^10
8.0×10^10
7.0×10^10
6.0×10^10
5.0×10^10
4.0×10^10
3.0×10^10
2.0×10^10
1.0×10^10
0.0
-1.0×10^10
-2.0×10^10

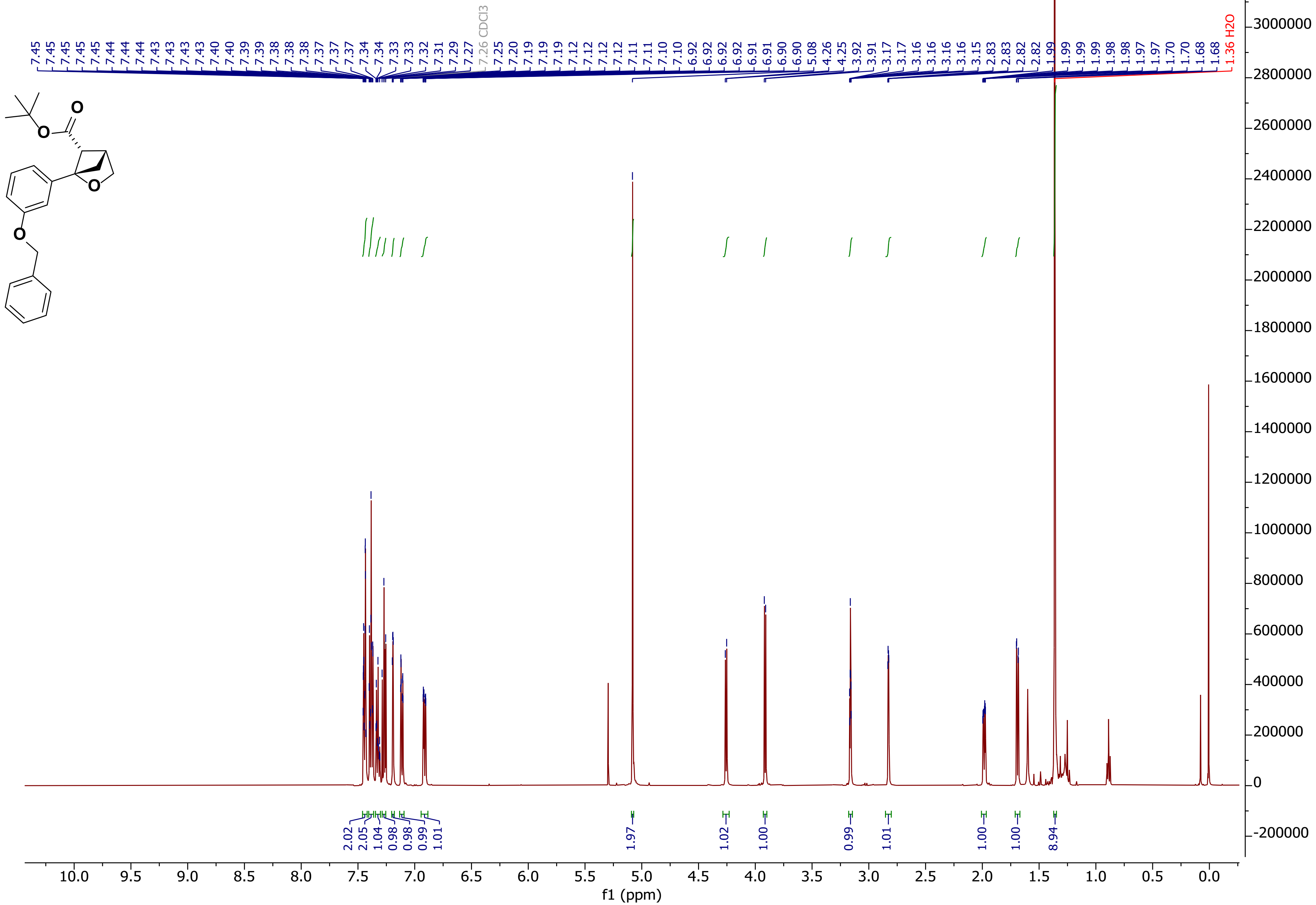
7.45 7.45 7.45 7.45 7.45 7.44 7.44 7.44 7.43 7.43 7.43 7.43 7.40 7.40 7.39 7.39 7.38 7.38 7.38 7.37 7.37 7.37 7.34 7.34 7.33 7.33 7.32 7.31 7.29 7.27
7.26 CDCl3
7.25 7.20 7.19 7.19 7.19 7.12 7.12 7.12 7.12 7.11 7.11 7.10 7.10 6.92 6.92 6.92 6.92 6.91 6.91 6.90 6.90 5.08 4.26 4.25 3.92 3.91 3.17 3.17 3.16 3.16 3.16 3.16 3.15 2.83 2.83 2.82 2.82 1.99 1.99 1.99 1.99 1.98 1.98 1.97 1.97 1.70 1.70 1.68 1.68
1.36 H2O
2.02 2.05 1.04 0.98 0.98 0.99 1.01 1.97 1.02 1.00 0.99 1.01 1.00 1.00 8.94
3000000 2800000 2600000 2400000 2200000 2000000 1800000 1600000 1400000 1200000 1000000 800000 600000 400000 200000 0 -200000
10.0 9.5 9.0 8.5 8.0 7.5 7.0 6.5 6.0 5.5 5.0 4.5 4.0 3.5 3.0 2.5 2.0 1.5 1.0 0.5 0.0
f1 (ppm)

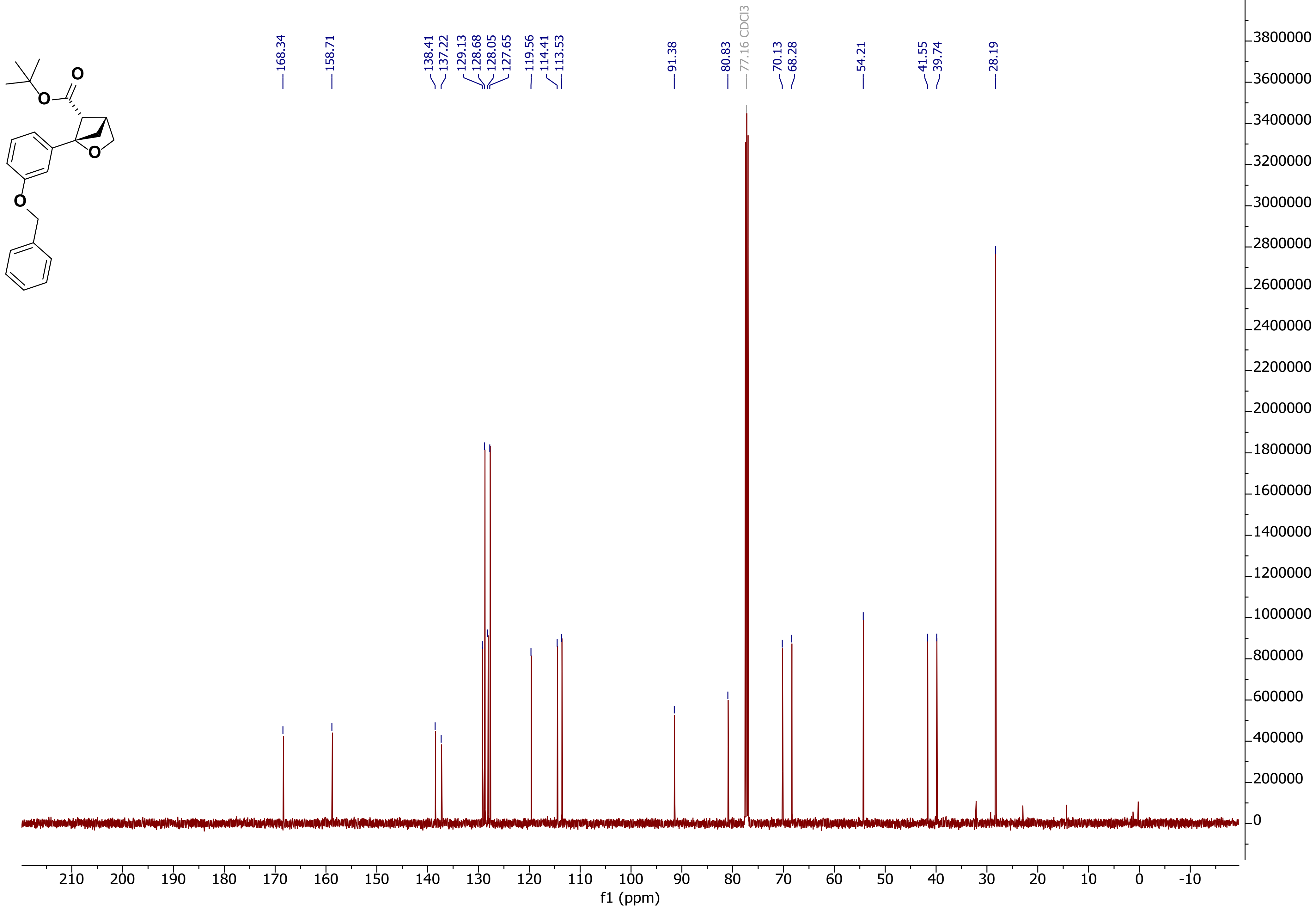

168.34
158.71
138.41
137.22
129.13
128.68
128.05
127.65
119.56
114.41
113.53
91.38
80.83
77.16 CDCl3
70.13
68.28
54.21
41.55
39.74
28.19
f1 (ppm)

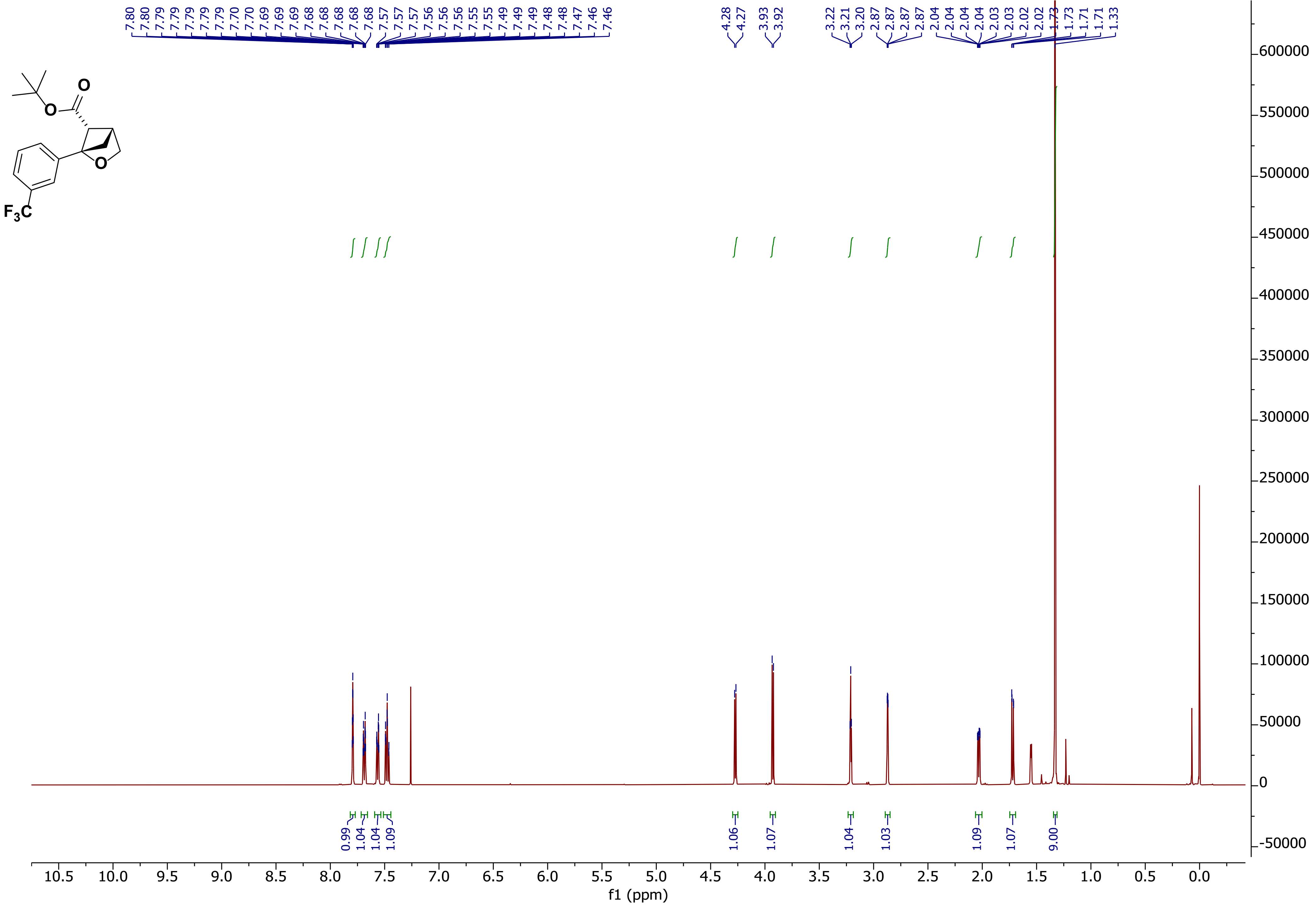

7.80 7.80 7.79 7.79 7.79 7.79 7.79 7.70 7.70 7.69 7.69 7.69 7.68 7.68 7.68 7.68 7.68 7.57 7.57 7.57 7.56 7.56 7.56 7.55 7.55 7.49 7.49 7.49 7.48 7.48 7.47 7.46 7.46
4.28 4.27 3.93 3.92
3.22 3.21 3.20 2.87 2.87 2.87 2.87 2.04 2.04 2.04 2.04 2.03 2.03 2.02 2.02 1.73 1.73 1.71 1.71 1.33
O
O
O
F3C
0.99 1.04 1.04 1.09 1.06 1.07 1.04 1.03 1.09 1.07 9.00
10.5 10.0 9.5 9.0 8.5 8.0 7.5 7.0 6.5 6.0 5.5 5.0 4.5 4.0 3.5 3.0 2.5 2.0 1.5 1.0 0.5 0.0
f1 (ppm)
600000 550000 500000 450000 400000 350000 300000 250000 200000 150000 100000 50000 0 -50000

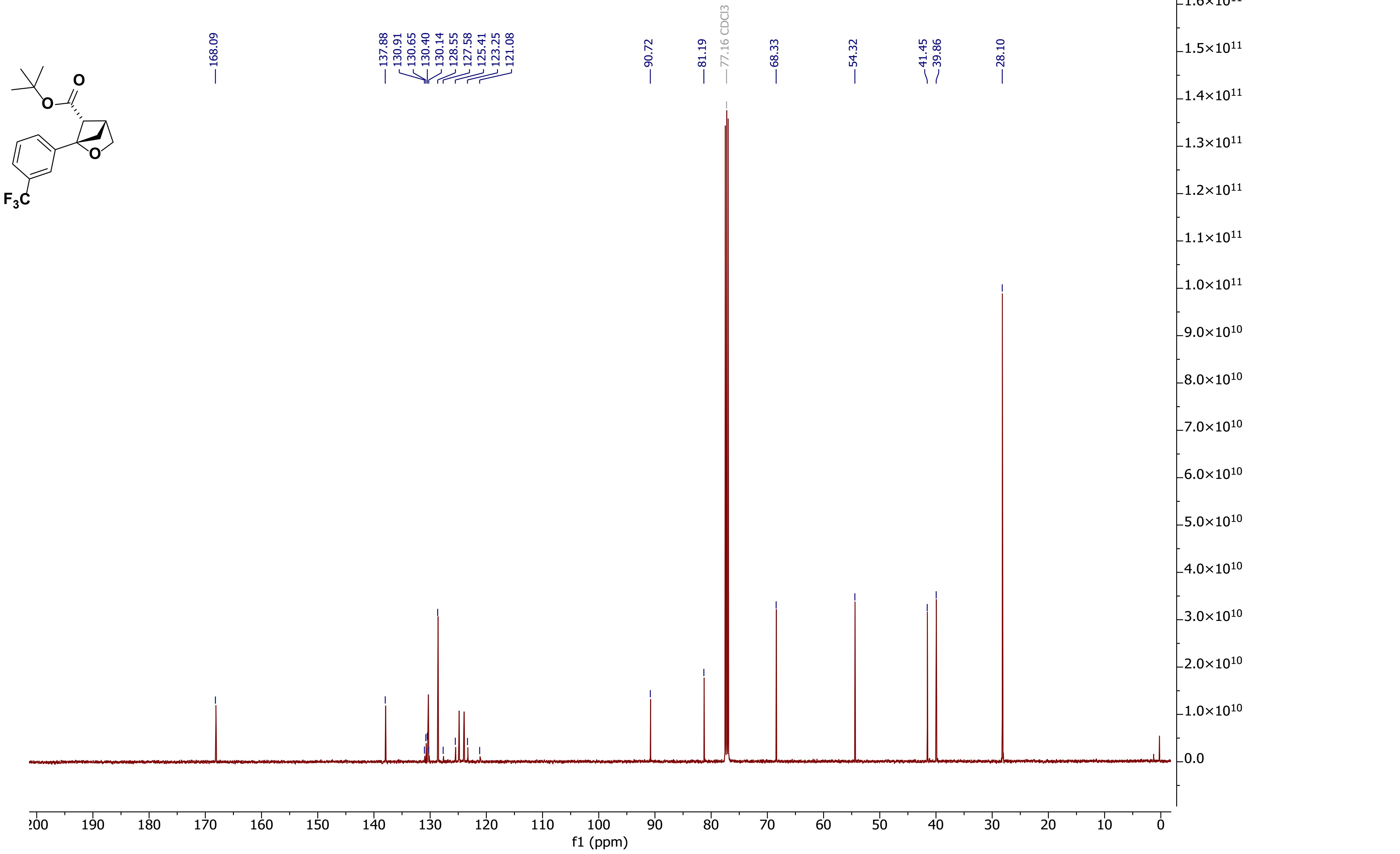
168.09
137.88
130.91
130.65
130.40
130.14
128.55
127.58
125.41
123.25
121.08
90.72
81.19
77.16 CDCl3
68.33
54.32
41.45
39.86
28.10
F3C
O
f1 (ppm)

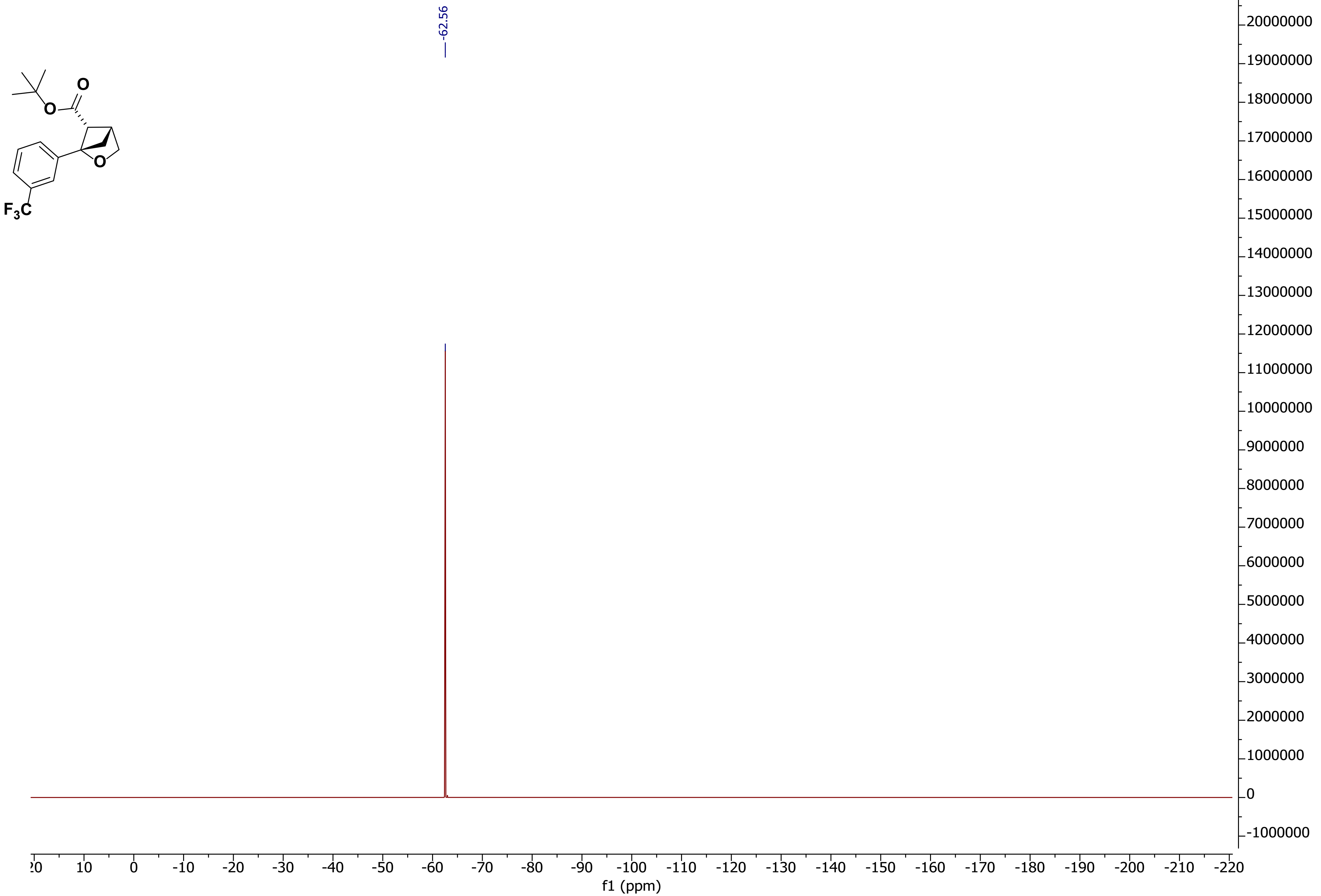
-62.56
O
O
O
F3C
f1 (ppm)

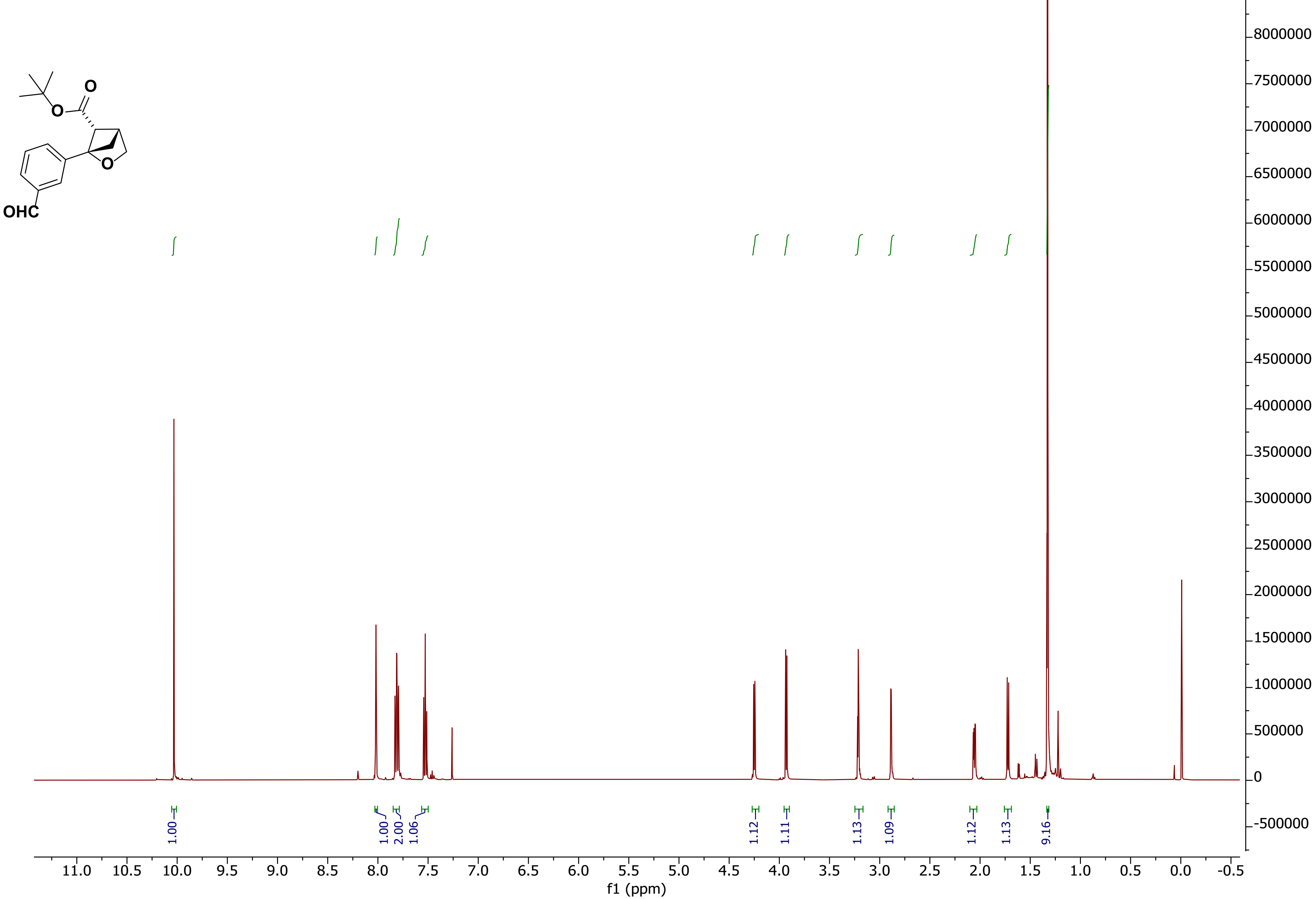

O
O
O
OHC
1.00
1.00
2.00
1.06
1.12
1.11
1.13
1.09
1.12
1.13
9.16
f1 (ppm)

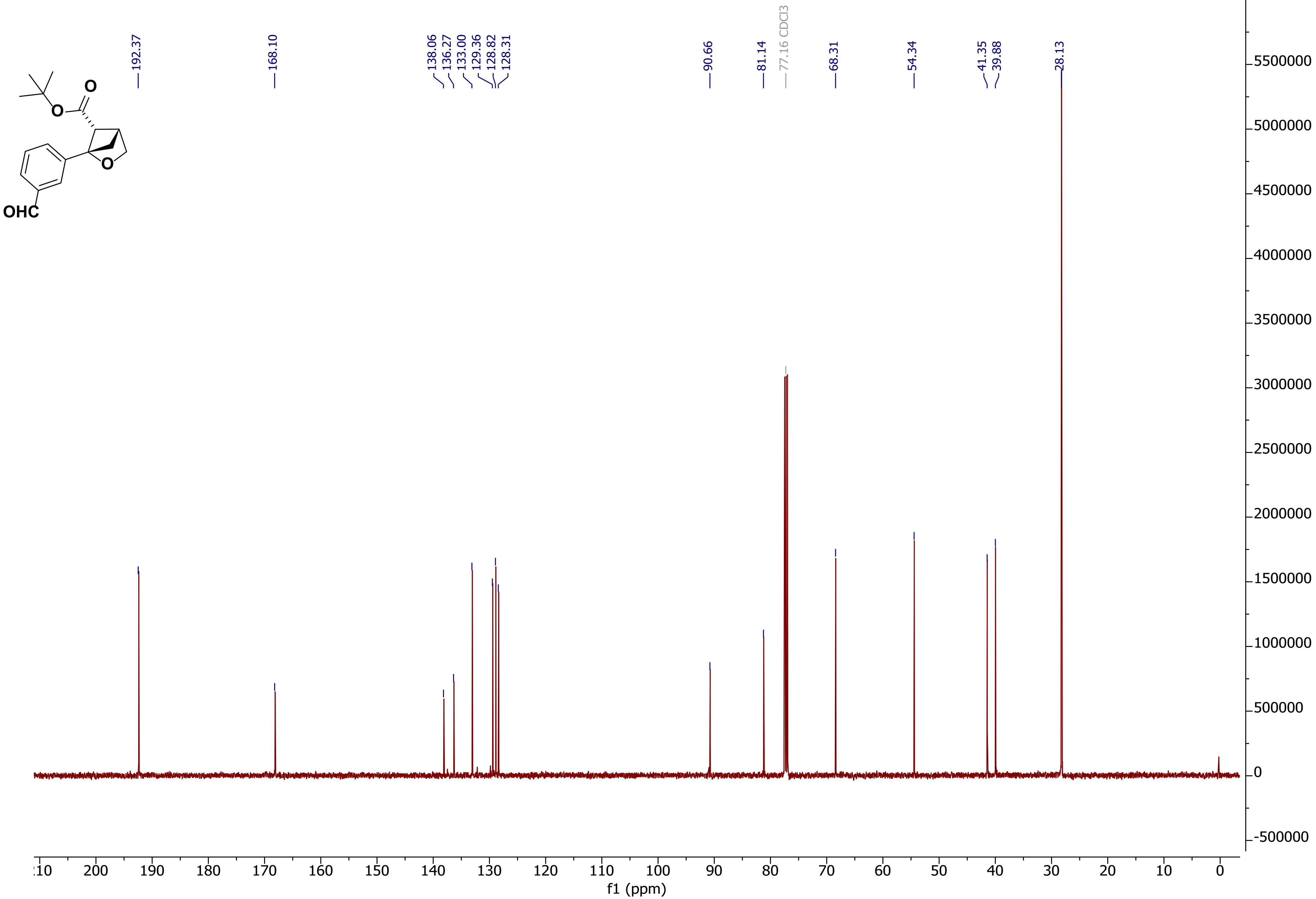

O
O
O
OHC
192.37
168.10
138.06
136.27
133.00
129.36
128.82
128.31
90.66
81.14
77.16 CDCl3
68.31
54.34
41.35
39.88
28.13
5500000
5000000
4500000
4000000
3500000
3000000
2500000
2000000
1500000
1000000
500000
0
-500000
210
200
190
180
170
160
150
140
130
120
110
100
90
80
70
60
50
40
30
20
10
0
f1 (ppm)

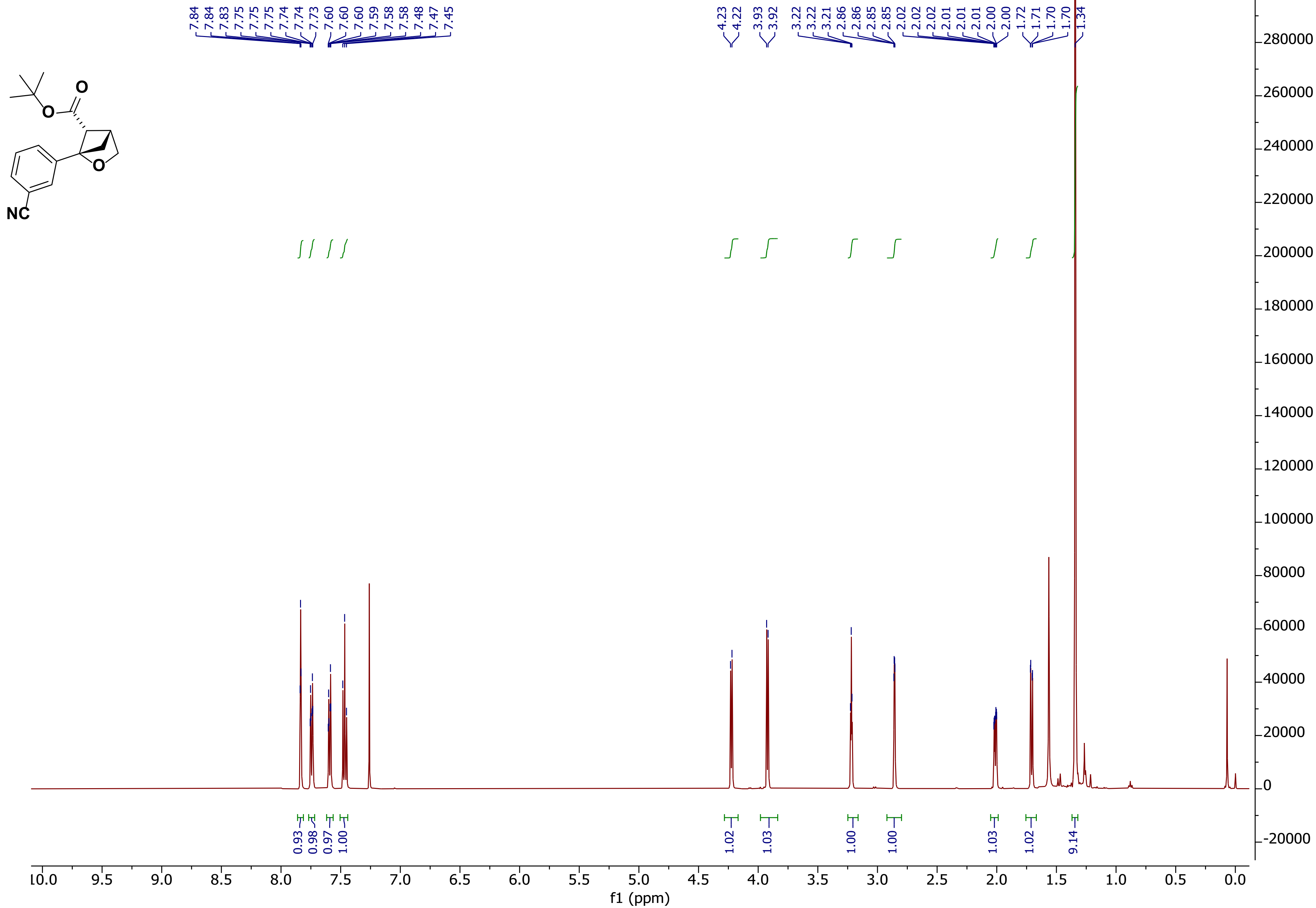

NC
O
O
O
7.84 7.84 7.83 7.75 7.75 7.75 7.74 7.74 7.73 7.60 7.60 7.60 7.59 7.58 7.58 7.48 7.47 7.45
4.23 4.22 3.93 3.92 3.22 3.22 3.21 2.86 2.85 2.85 2.02 2.02 2.02 2.01 2.01 2.01 2.00 2.00 1.72 1.71 1.70 1.70 1.34
0.93 0.98 0.97 1.00 1.02 1.03 1.00 1.00 1.03 1.02 9.14
f1 (ppm)

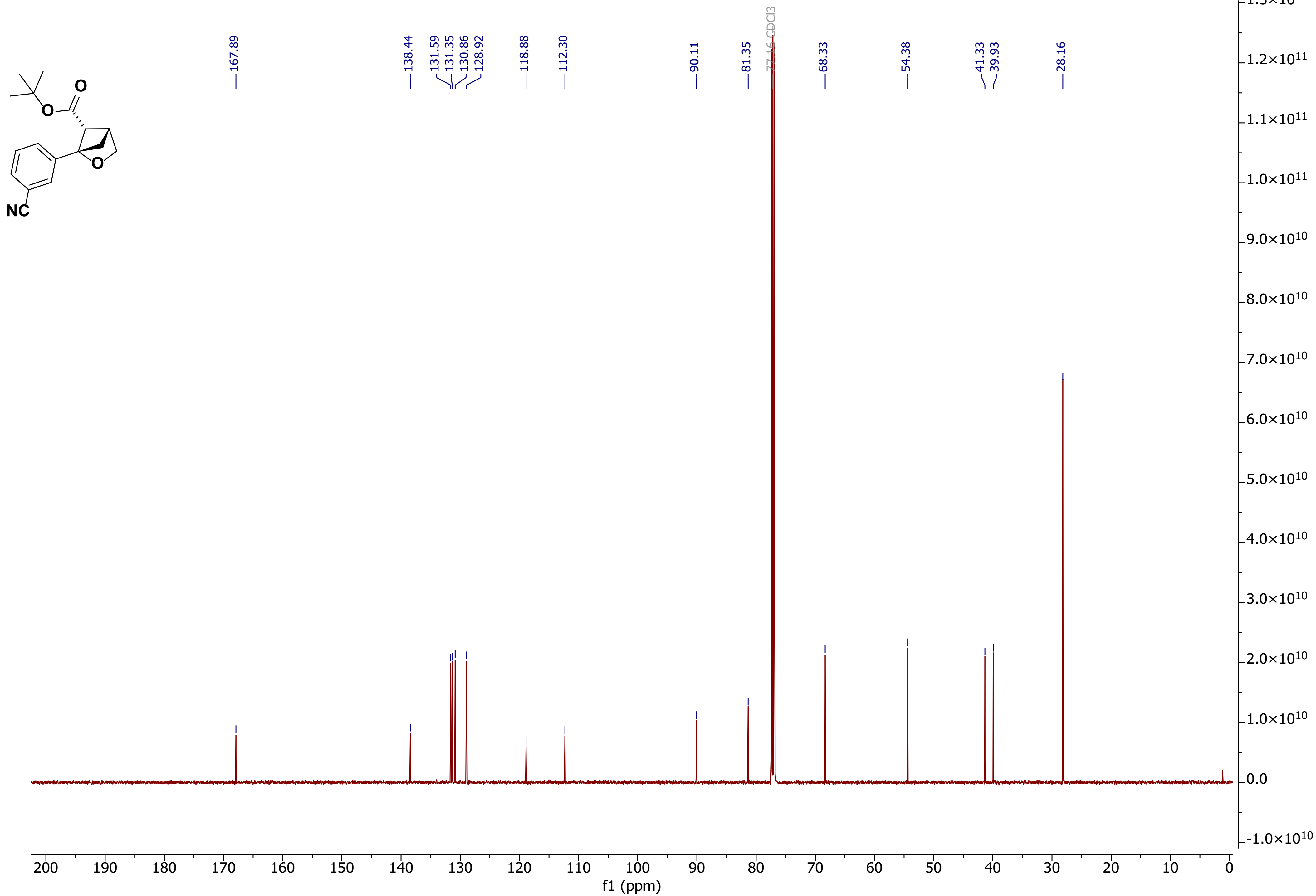
NC
O
O
O
167.89
138.44
131.59
131.35
130.86
128.92
118.88
112.30
90.11
81.35
77.16 CDCl3
68.33
54.38
41.33
39.93
28.16
f1 (ppm)
200
190
180
170
160
150
140
130
120
110
100
90
80
70
60
50
40
30
20
10
0
1.2×10^11
1.1×10^11
1.0×10^11
9.0×10^10
8.0×10^10
7.0×10^10
6.0×10^10
5.0×10^10
4.0×10^10
3.0×10^10
2.0×10^10
1.0×10^10
0.0
-1.0×10^10

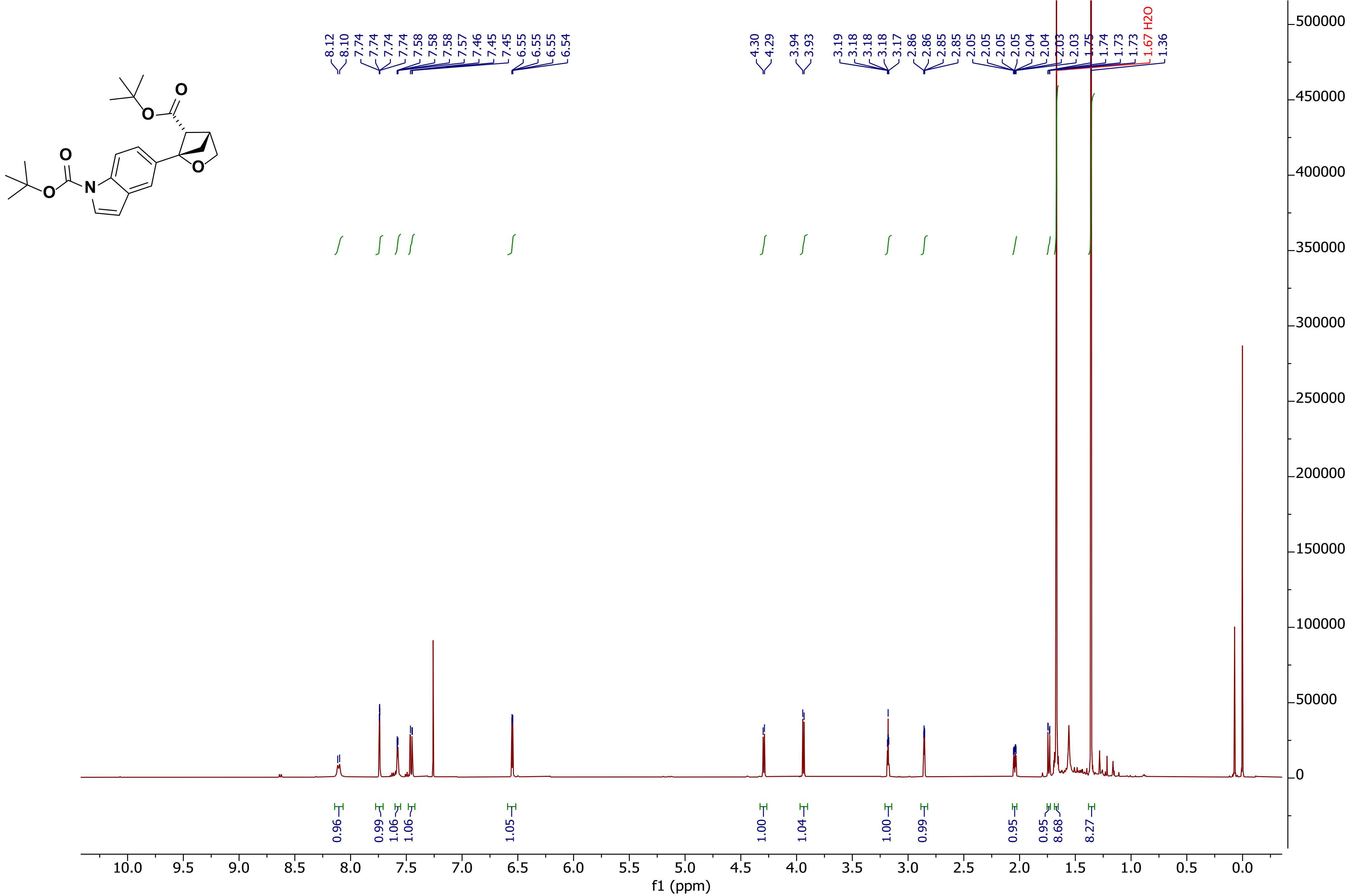

O
O
O
O
N
O
8.12
8.10
7.74
7.74
7.74
7.74
7.58
7.58
7.58
7.57
7.46
7.45
7.45
6.55
6.55
6.55
6.54
4.30
4.29
3.94
3.93
3.19
3.18
3.18
3.18
3.17
2.86
2.86
2.85
2.85
2.05
2.05
2.05
2.05
2.04
2.04
2.03
2.03
1.75
1.74
1.73
1.73
1.67 H2O
1.36
0.96
0.99
1.06
1.06
1.05
1.00
1.04
1.00
0.99
0.95
0.95
8.68
8.27
500000
450000
400000
350000
300000
250000
200000
150000
100000
50000
0
10.0
9.5
9.0
8.5
8.0
7.5
7.0
6.5
6.0
5.5
5.0
4.5
4.0
3.5
3.0
2.5
2.0
1.5
1.0
0.5
0.0
f1 (ppm)

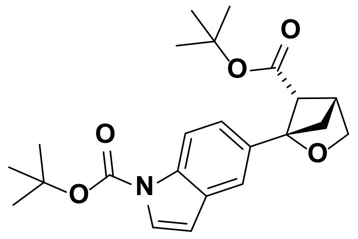

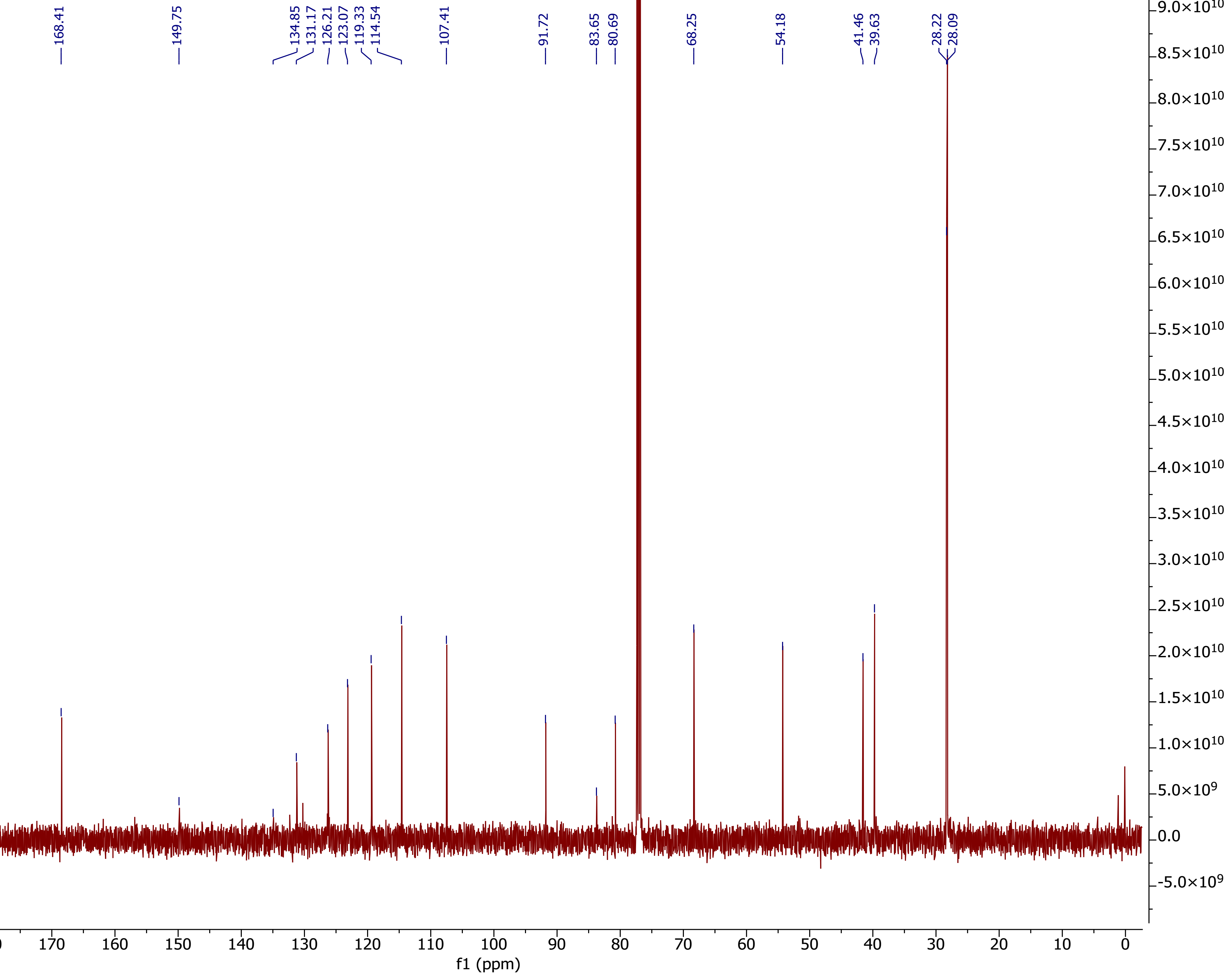
168.41
149.75
134.85
131.17
126.21
123.07
119.33
114.54
107.41
91.72
83.65
80.69
68.25
54.18
41.46
39.63
28.22
28.09
f1 (ppm)

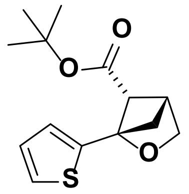

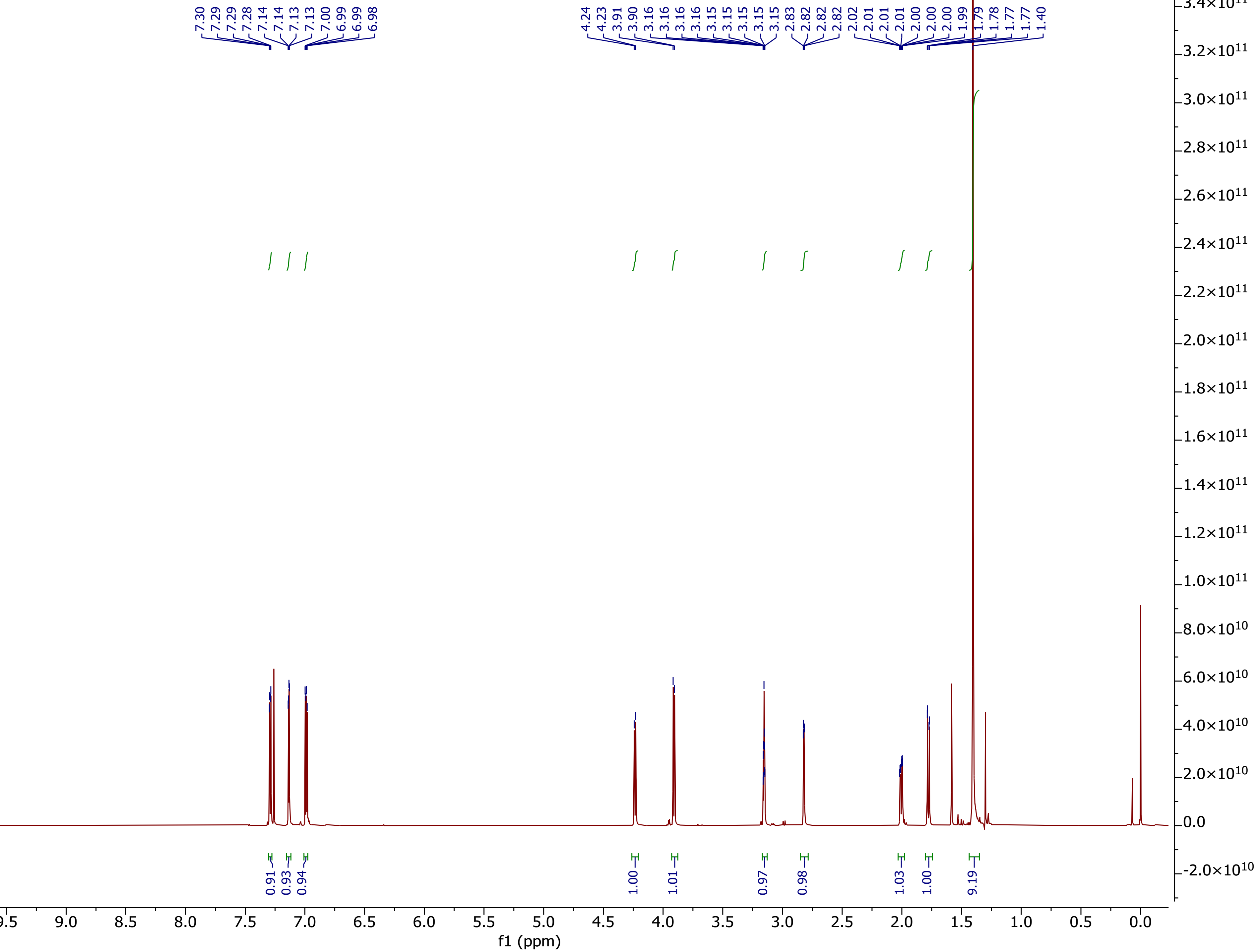
7.30
7.29
7.29
7.28
7.14
7.14
7.13
7.13
7.00
6.99
6.99
6.98
4.24
4.23
3.91
3.90
3.16
3.16
3.16
3.16
3.15
3.15
3.15
3.15
3.15
2.83
2.82
2.82
2.82
2.02
2.01
2.01
2.01
2.00
2.00
2.00
1.99
1.79
1.78
1.77
1.77
1.40
0.91
0.93
0.94
1.00
1.01
0.97
0.98
1.03
1.00
9.19
f1 (ppm)

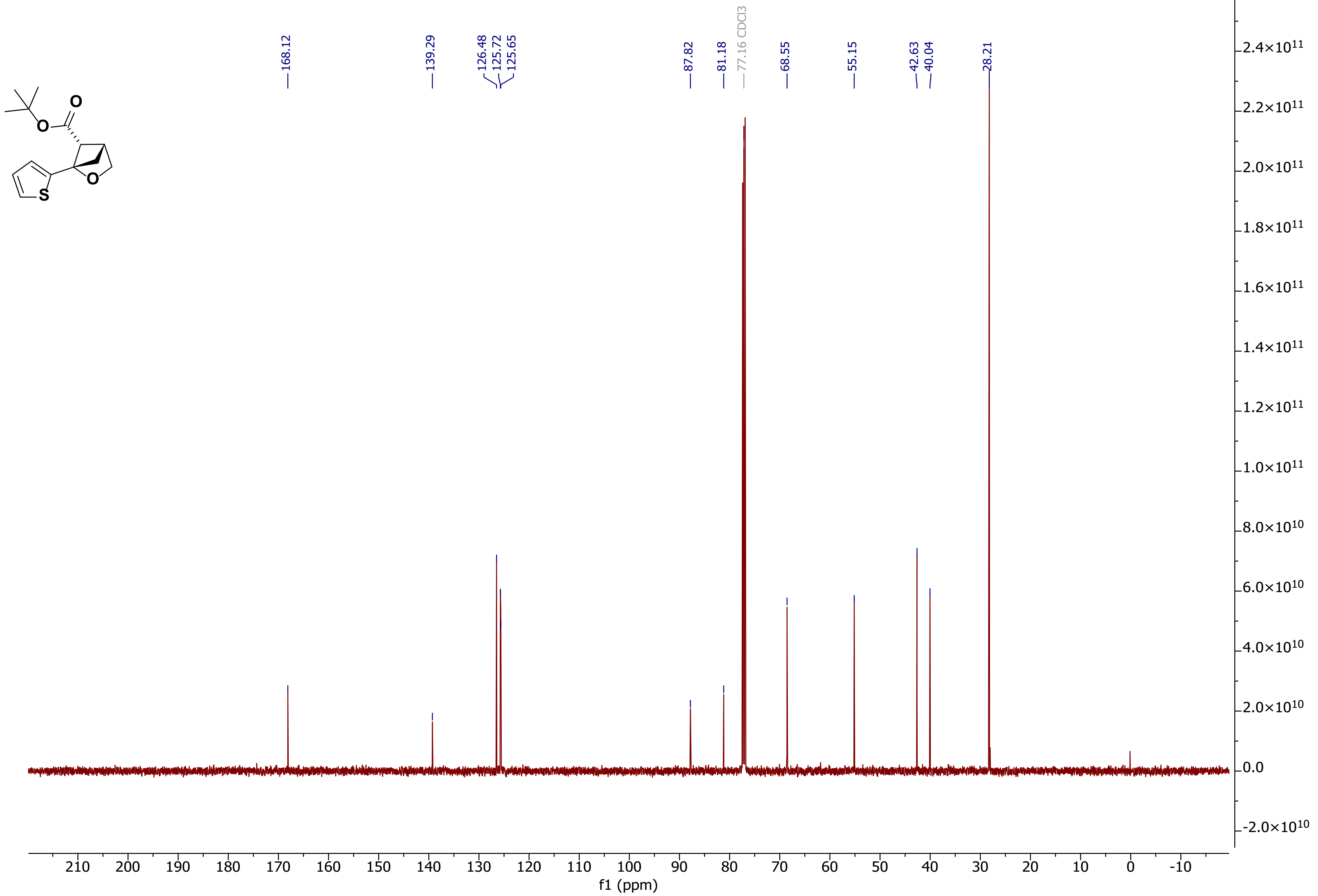
O
O
O
S
168.12
139.29
126.48
125.72
125.65
87.82
81.18
77.16 CDCl3
68.55
55.15
42.63
40.04
28.21
f1 (ppm)

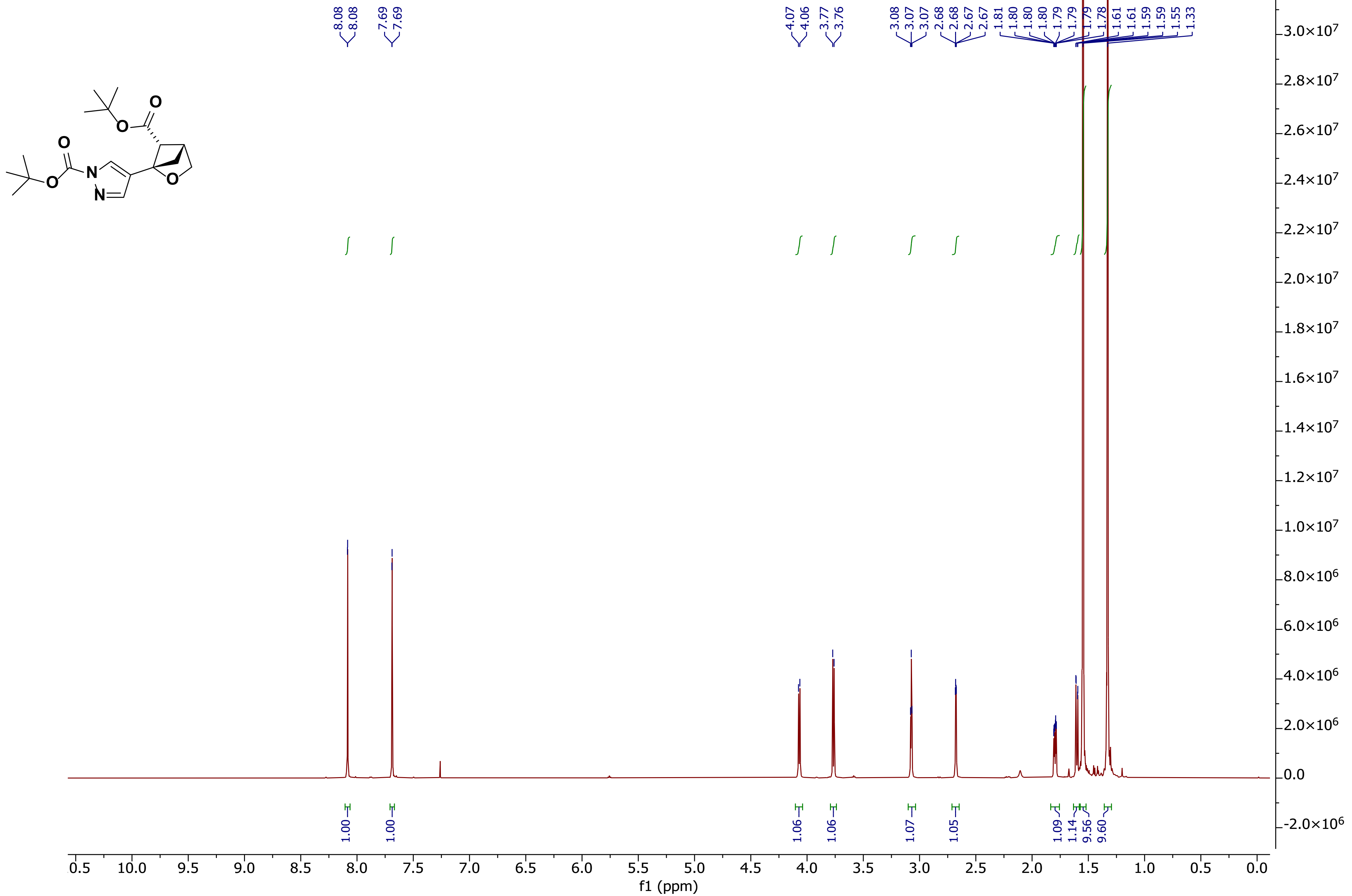
8.08
8.08
7.69
7.69
4.07
4.06
3.77
3.76
3.08
3.07
3.07
2.68
2.68
2.67
2.67
1.81
1.80
1.80
1.80
1.79
1.79
1.79
1.78
1.61
1.61
1.59
1.59
1.55
1.33
1.00
1.00
1.06
1.06
1.07
1.05
1.09
1.14
9.56
9.60
f1 (ppm)

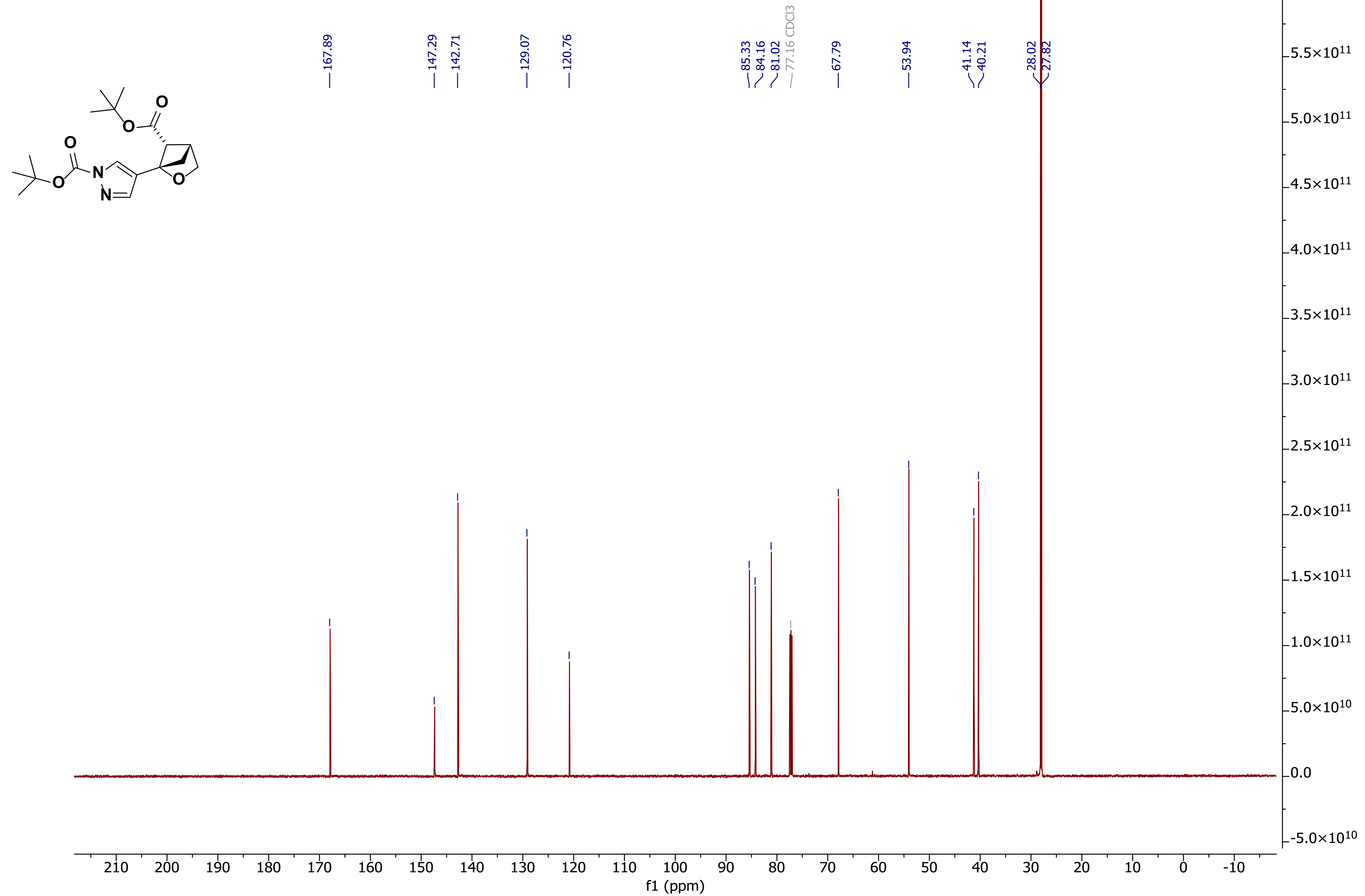
167.89
147.29
142.71
129.07
120.76
85.33
84.16
81.02
77.16 CDCl3
67.79
53.94
41.14
40.21
28.02
27.82
5.5×10^11
5.0×10^11
4.5×10^11
4.0×10^11
3.5×10^11
3.0×10^11
2.5×10^11
2.0×10^11
1.5×10^11
1.0×10^11
5.0×10^10
0.0
-5.0×10^10
210 200 190 180 170 160 150 140 130 120 110 100 90 80 70 60 50 40 30 20 10 0 -10
f1 (ppm)

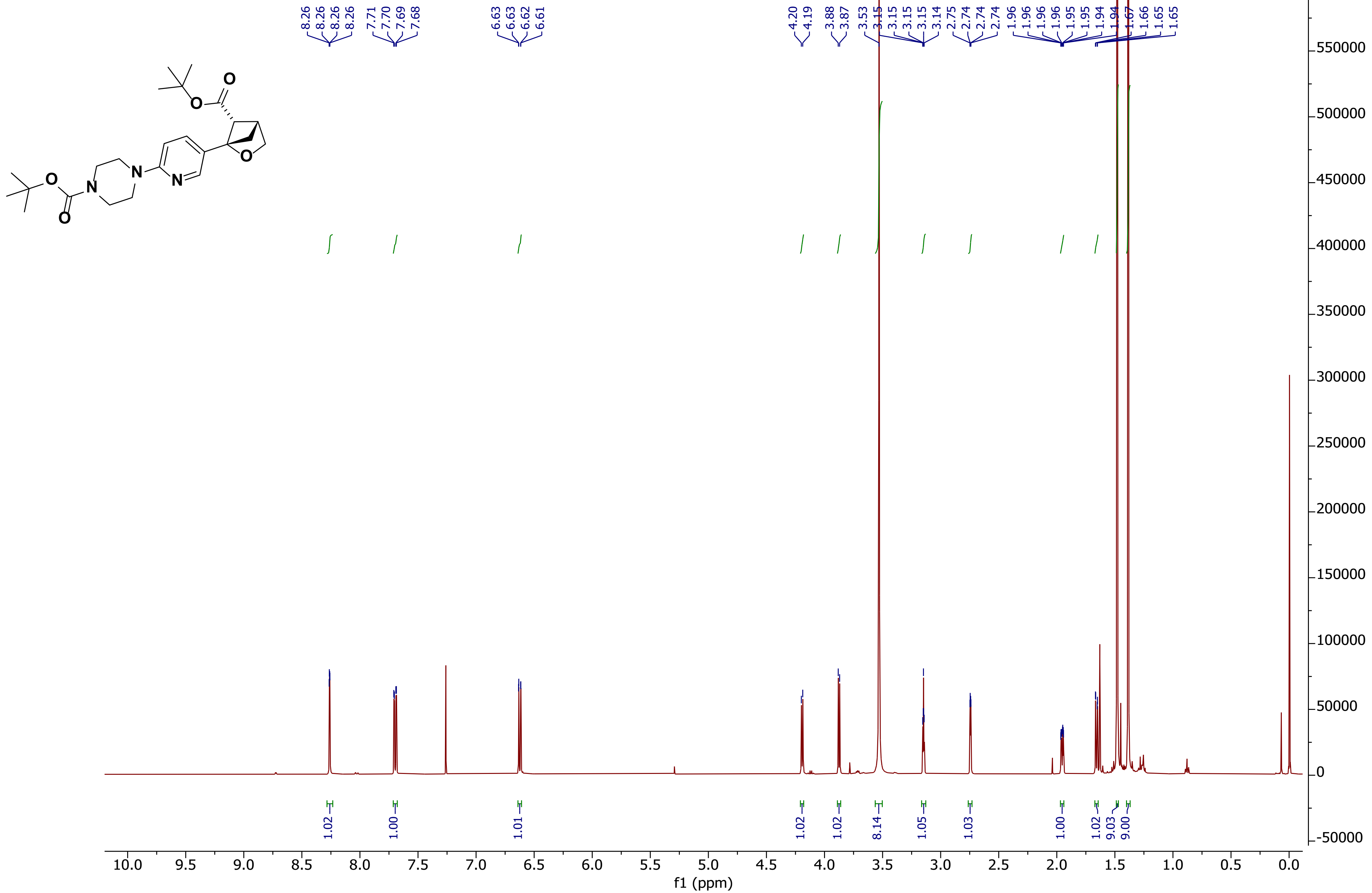
8.26
8.26
8.26
8.26
7.71
7.70
7.69
7.68
6.63
6.63
6.62
6.61
4.20
4.19
3.88
3.87
3.53
3.15
3.15
3.15
3.15
3.14
2.75
2.74
2.74
2.74
1.96
1.96
1.96
1.95
1.95
1.94
1.94
1.67
1.66
1.65
1.65
O
O
O
O
O
N
N
N
1.02
1.00
1.01
1.02
1.02
8.14
1.05
1.03
1.00
1.02
9.03
9.00
550000
500000
450000
400000
350000
300000
250000
200000
150000
100000
50000
0
-50000
10.0
9.5
9.0
8.5
8.0
7.5
7.0
6.5
6.0
5.5
5.0
4.5
4.0
3.5
3.0
2.5
2.0
1.5
1.0
0.5
0.0
f1 (ppm)

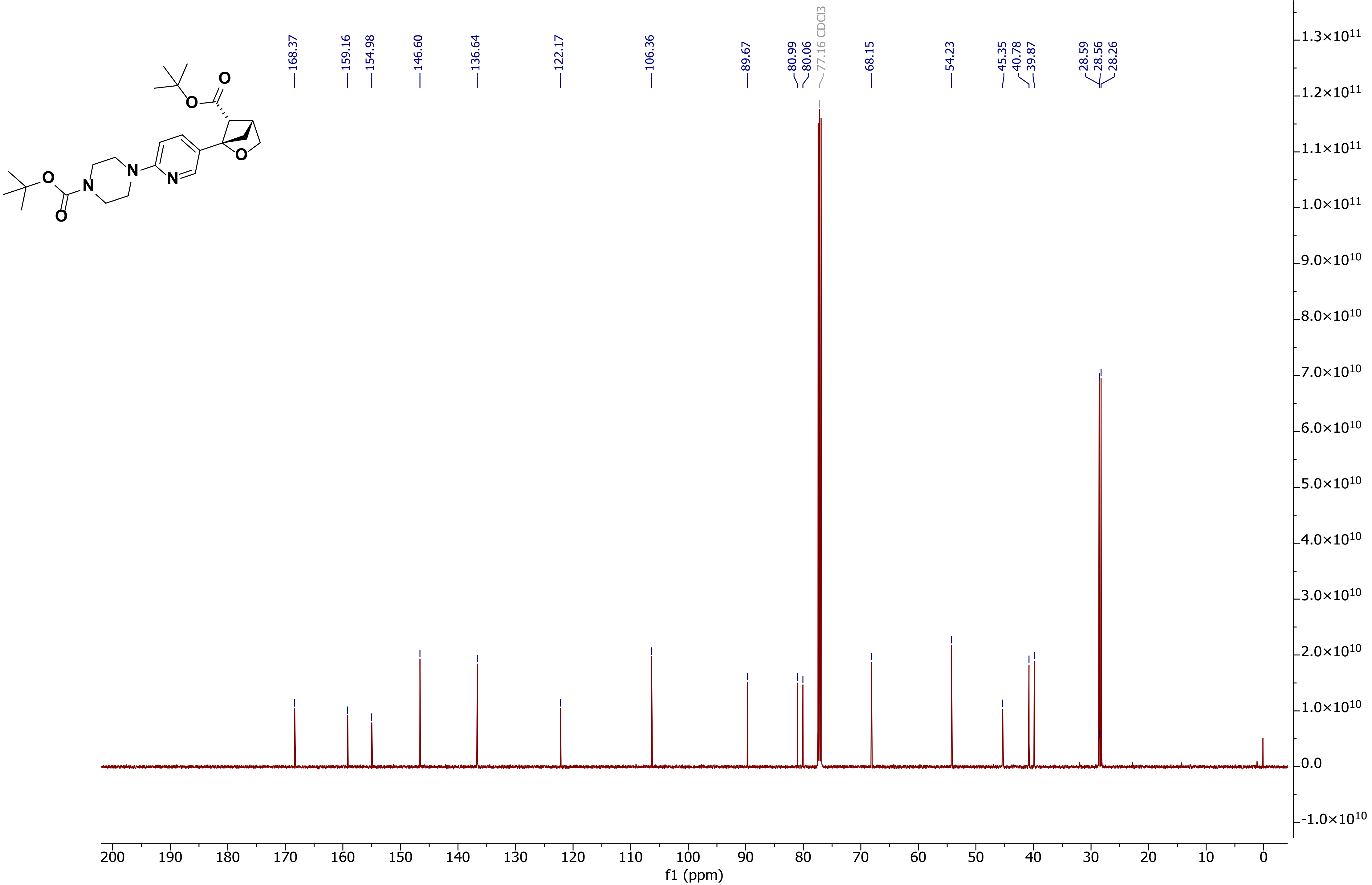

168.37
159.16
154.98
146.60
136.64
122.17
106.36
89.67
80.99
80.06
77.16 CDCl3
68.15
54.23
45.35
40.78
39.87
28.59
28.56
28.26
f1 (ppm)

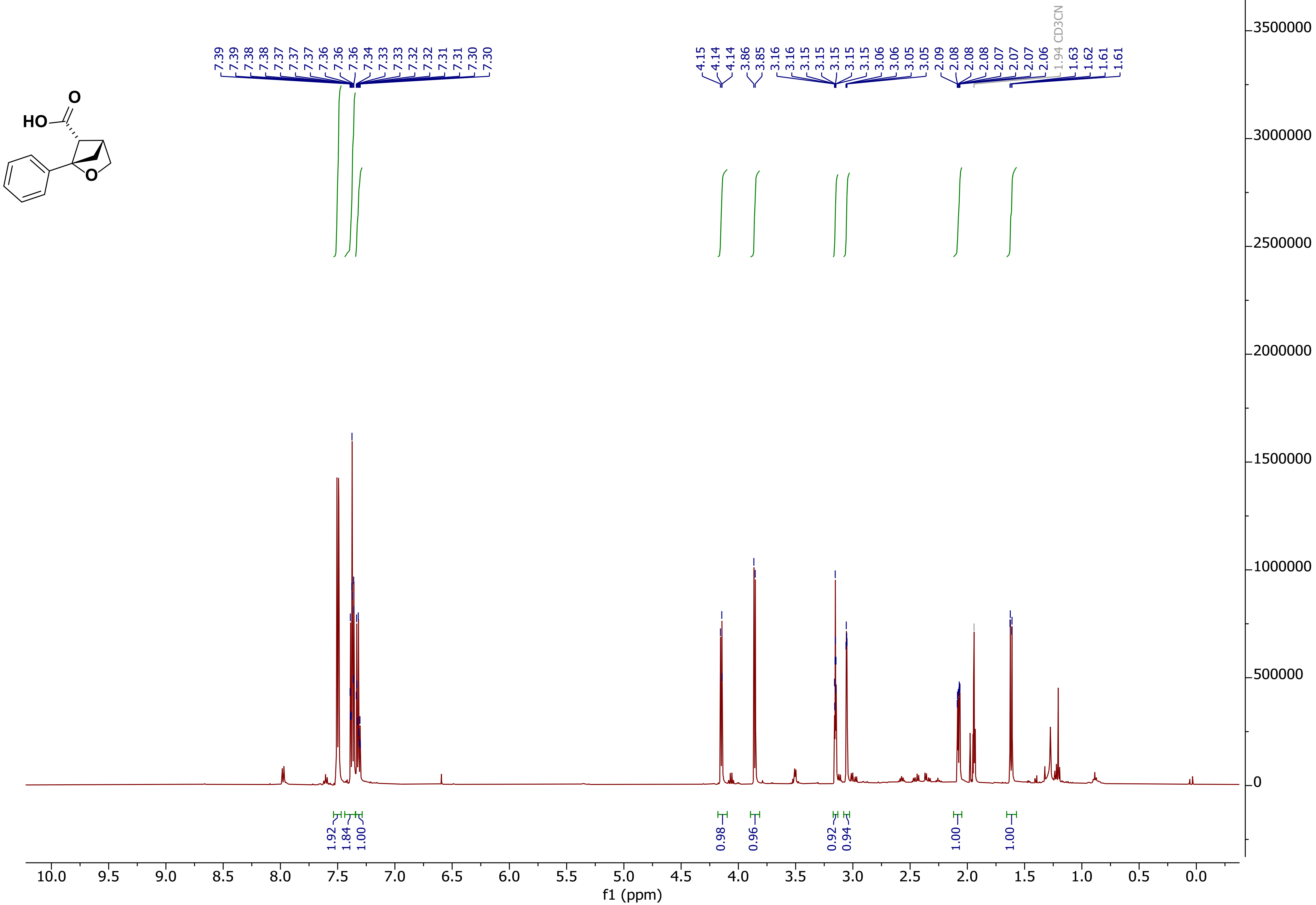

7.39 7.39 7.38 7.38 7.37 7.37 7.37 7.36 7.36 7.36 7.34 7.33 7.33 7.32 7.32 7.31 7.31 7.30 7.30
4.15 4.14 4.14 3.86 3.85 3.16 3.16 3.15 3.15 3.15 3.15 3.15 3.06 3.06 3.05 3.05 2.09 2.08 2.08 2.08 2.07 2.07 2.07 2.06 1.94 CD3CN 1.63 1.62 1.61 1.61
1.92 1.84 1.00 0.98 0.96 0.92 0.94 1.00 1.00
HO
O
O
f1 (ppm)

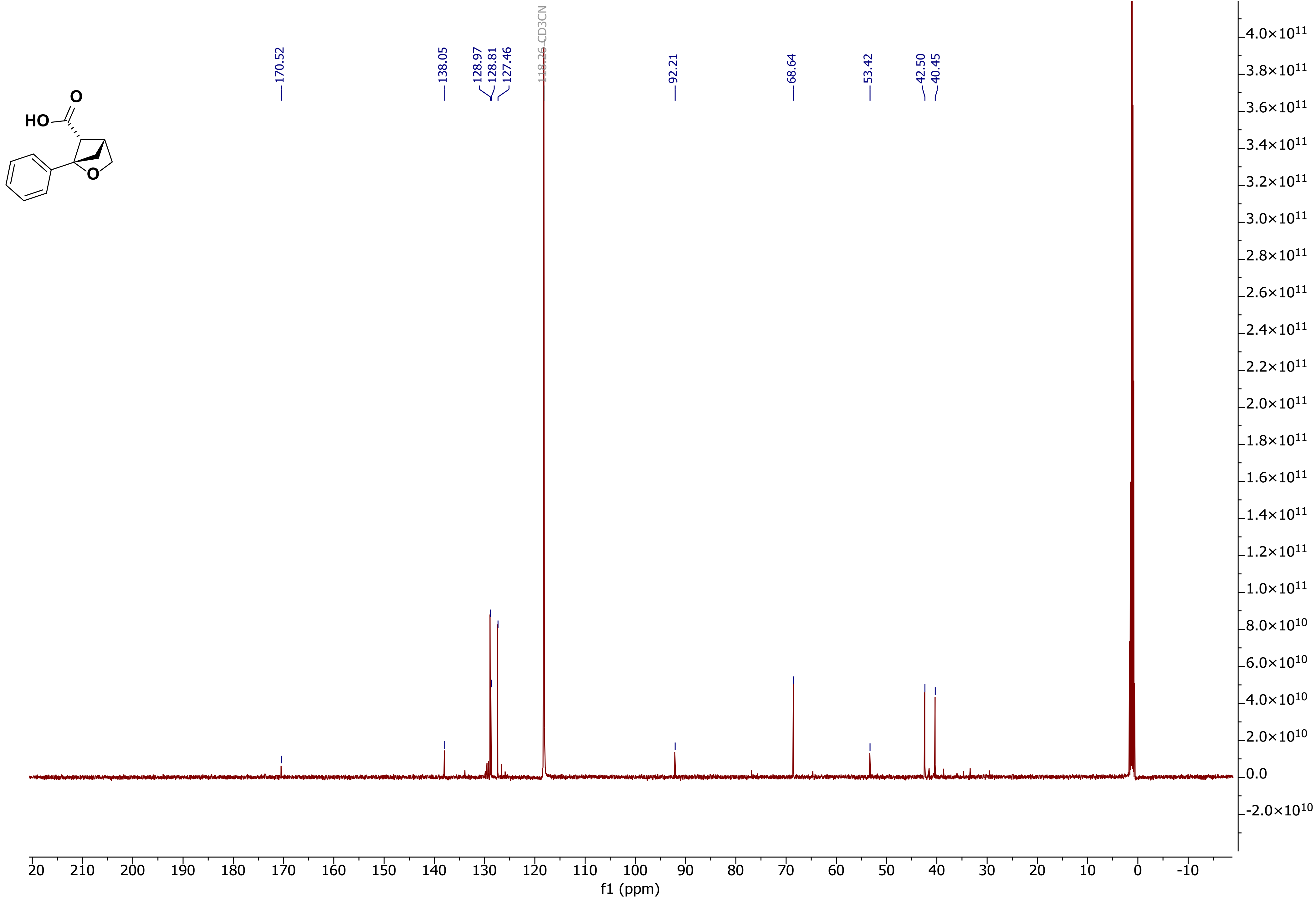
HO
O
O
170.52
138.05
128.97
128.81
127.46
118.26 CD3CN
92.21
68.64
53.42
42.50
40.45
f1 (ppm)

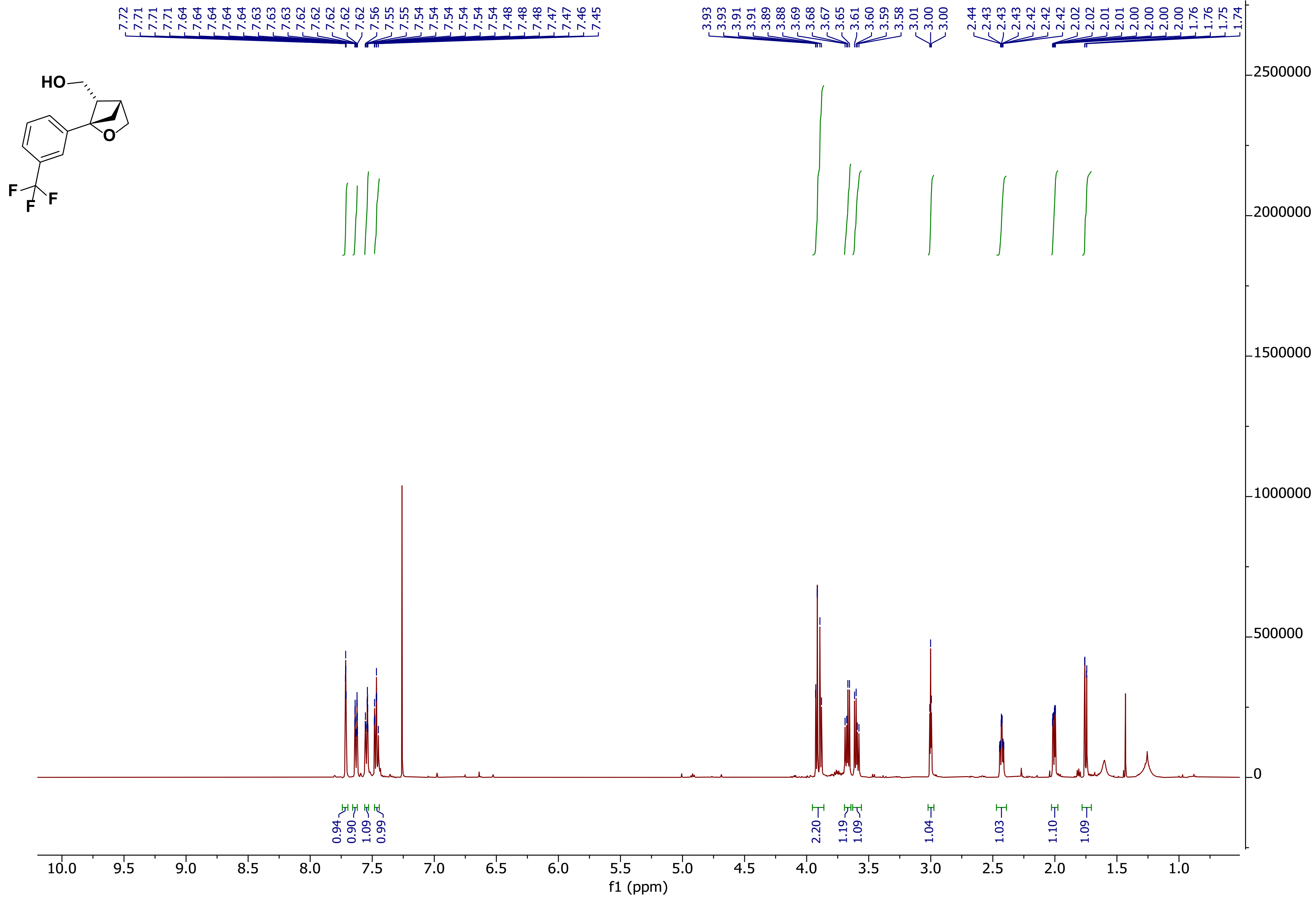
7.72 7.71 7.71 7.71 7.64 7.64 7.64 7.64 7.63 7.63 7.63 7.62 7.62 7.62 7.62 7.62 7.56 7.55 7.55 7.54 7.54 7.54 7.54 7.54 7.54 7.48 7.48 7.47 7.47 7.46 7.45
3.93 3.93 3.91 3.91 3.89 3.88 3.69 3.68 3.67 3.65 3.61 3.60 3.59 3.58 3.01 3.00 3.00
2.44 2.43 2.43 2.43 2.42 2.42 2.42 2.02 2.02 2.01 2.01 2.00 2.00 2.00 2.00 1.76 1.76 1.75 1.74
HO
O
F
F
F
2500000
2000000
1500000
1000000
500000
0
0.94 0.90 1.09 0.99 2.20 1.19 1.09 1.04 1.03 1.10 1.09
10.0 9.5 9.0 8.5 8.0 7.5 7.0 6.5 6.0 5.5 5.0 4.5 4.0 3.5 3.0 2.5 2.0 1.5 1.0
f1 (ppm)

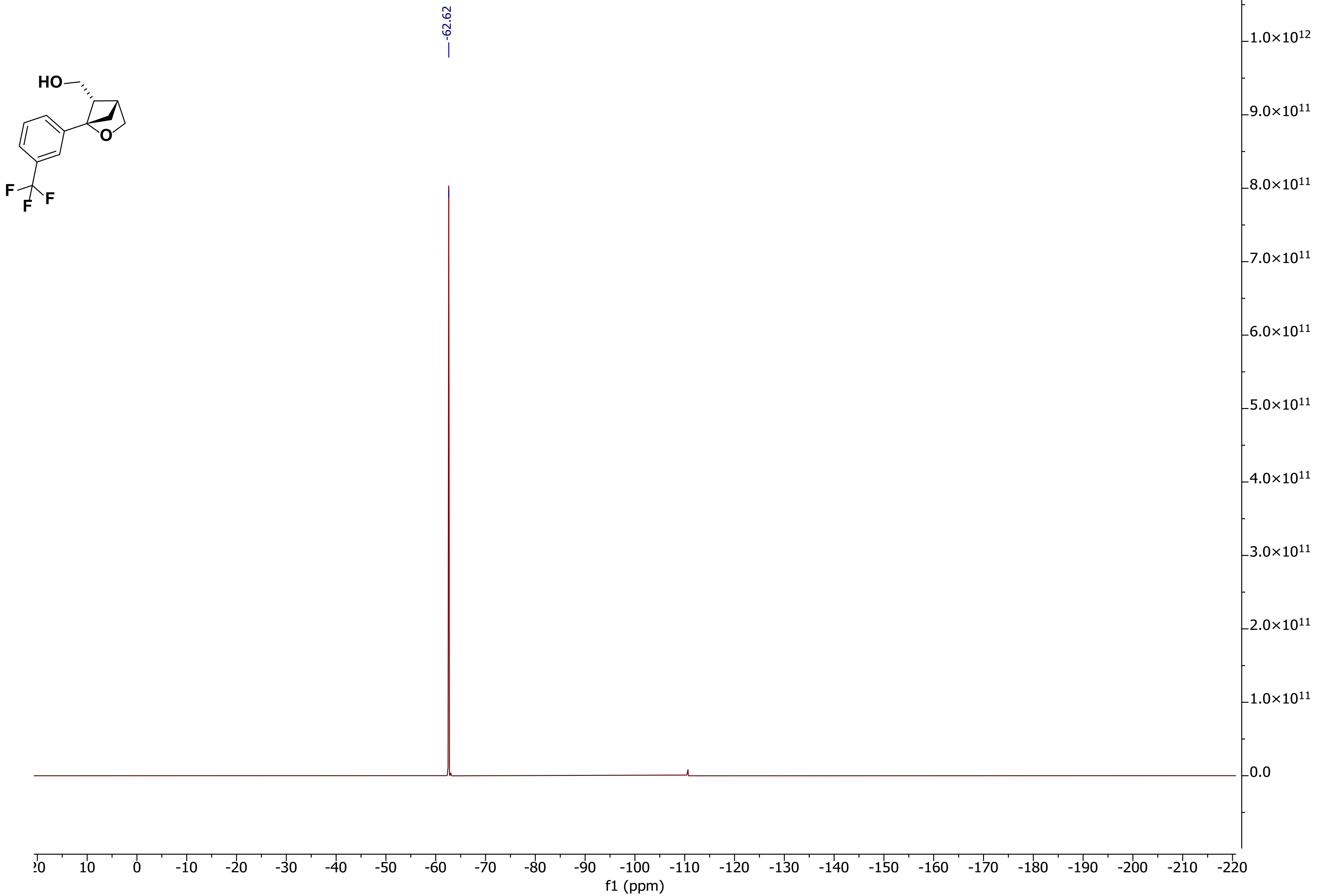

-62.62
HO
O
F
F
F
1.0×10^12
9.0×10^11
8.0×10^11
7.0×10^11
6.0×10^11
5.0×10^11
4.0×10^11
3.0×10^11
2.0×10^11
1.0×10^11
0.0
20 10 0 -10 -20 -30 -40 -50 -60 -70 -80 -90 -100 -110 -120 -130 -140 -150 -160 -170 -180 -190 -200 -210 -220
f1 (ppm)

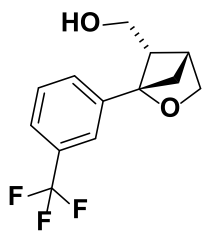

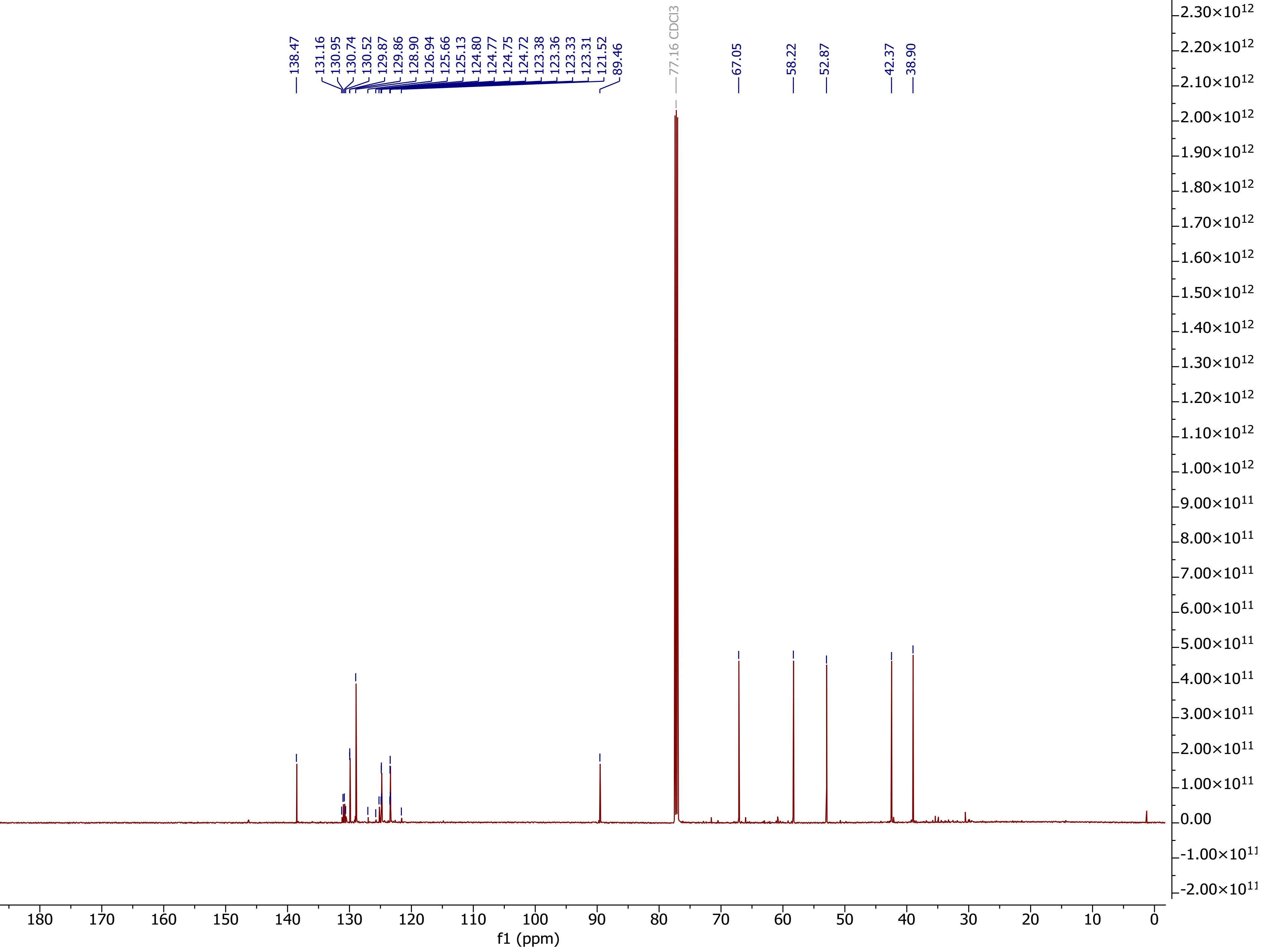

138.47
131.16
130.95
130.74
130.52
129.87
129.86
128.90
126.94
125.66
125.13
124.80
124.77
124.75
124.72
123.38
123.36
123.33
123.31
121.52
89.46
77.16 CDCl3
67.05
58.22
52.87
42.37
38.90
f1 (ppm)

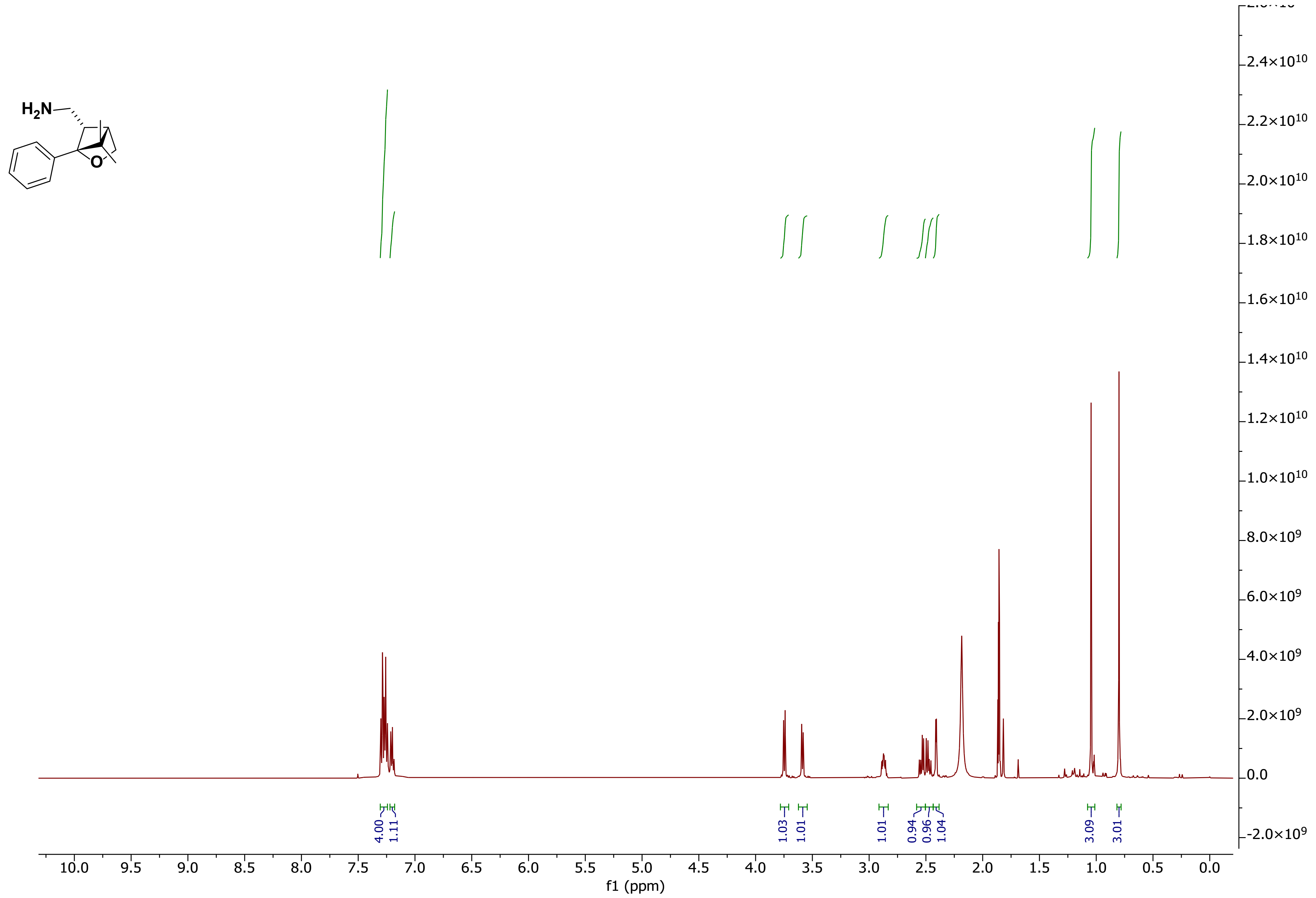
H2N
O
4.00
1.11
1.03
1.01
1.01
0.94
0.96
1.04
3.09
3.01
10.0 9.5 9.0 8.5 8.0 7.5 7.0 6.5 6.0 5.5 5.0 4.5 4.0 3.5 3.0 2.5 2.0 1.5 1.0 0.5 0.0
f1 (ppm)
2.4×10^10
2.2×10^10
2.0×10^10
1.8×10^10
1.6×10^10
1.4×10^10
1.2×10^10
1.0×10^10
8.0×10^9
6.0×10^9
4.0×10^9
2.0×10^9
0.0
-2.0×10^9

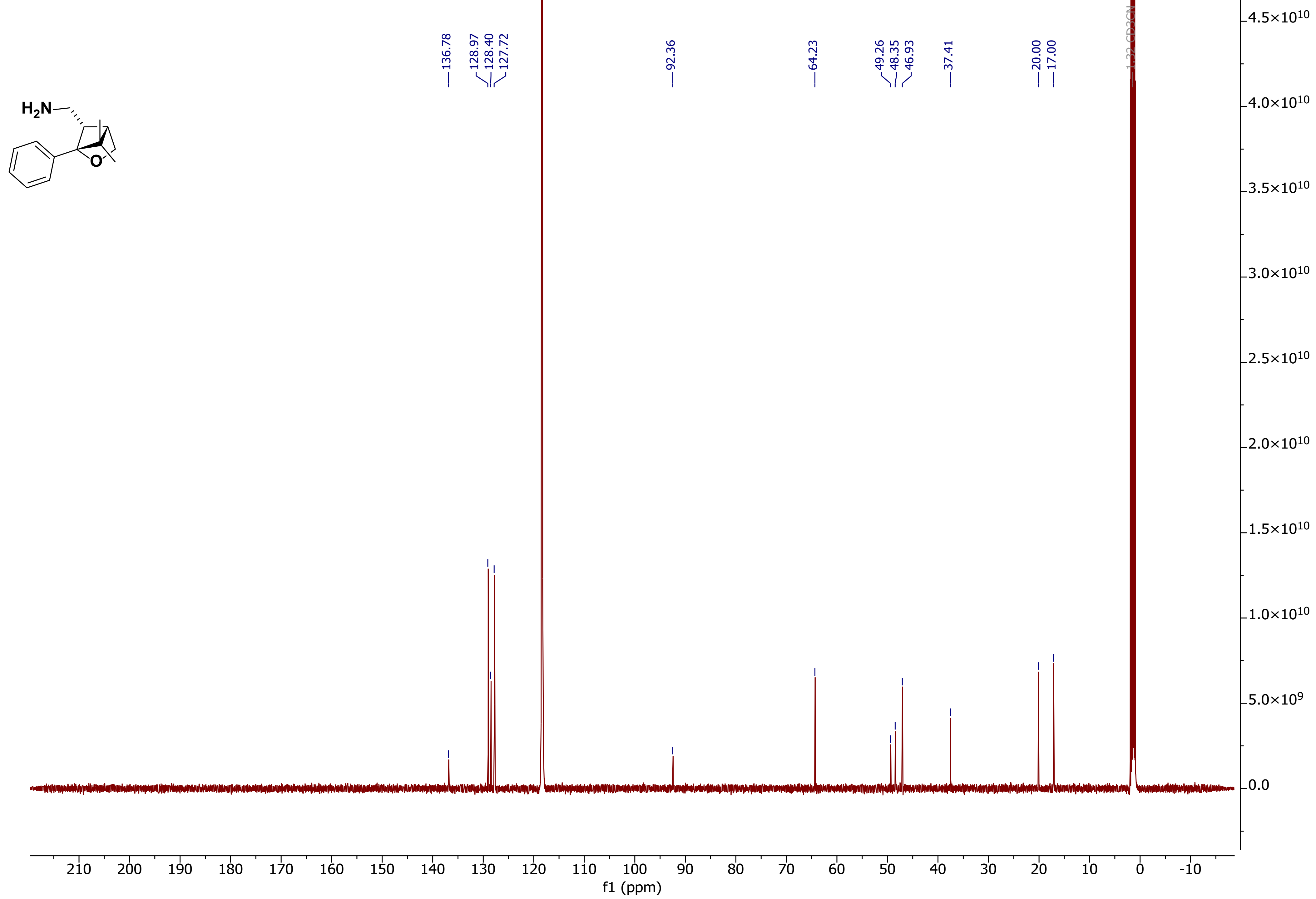
H2N
O
136.78
128.97
128.40
127.72
92.36
64.23
49.26
48.35
46.93
37.41
20.00
17.00
1.32 CD3CN
4.5×10^10
4.0×10^10
3.5×10^10
3.0×10^10
2.5×10^10
2.0×10^10
1.5×10^10
1.0×10^10
5.0×10^9
0.0
210
200
190
180
170
160
150
140
130
120
110
100
90
80
70
60
50
40
30
20
10
0
-10
f1 (ppm)

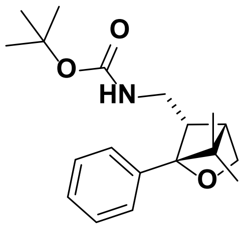

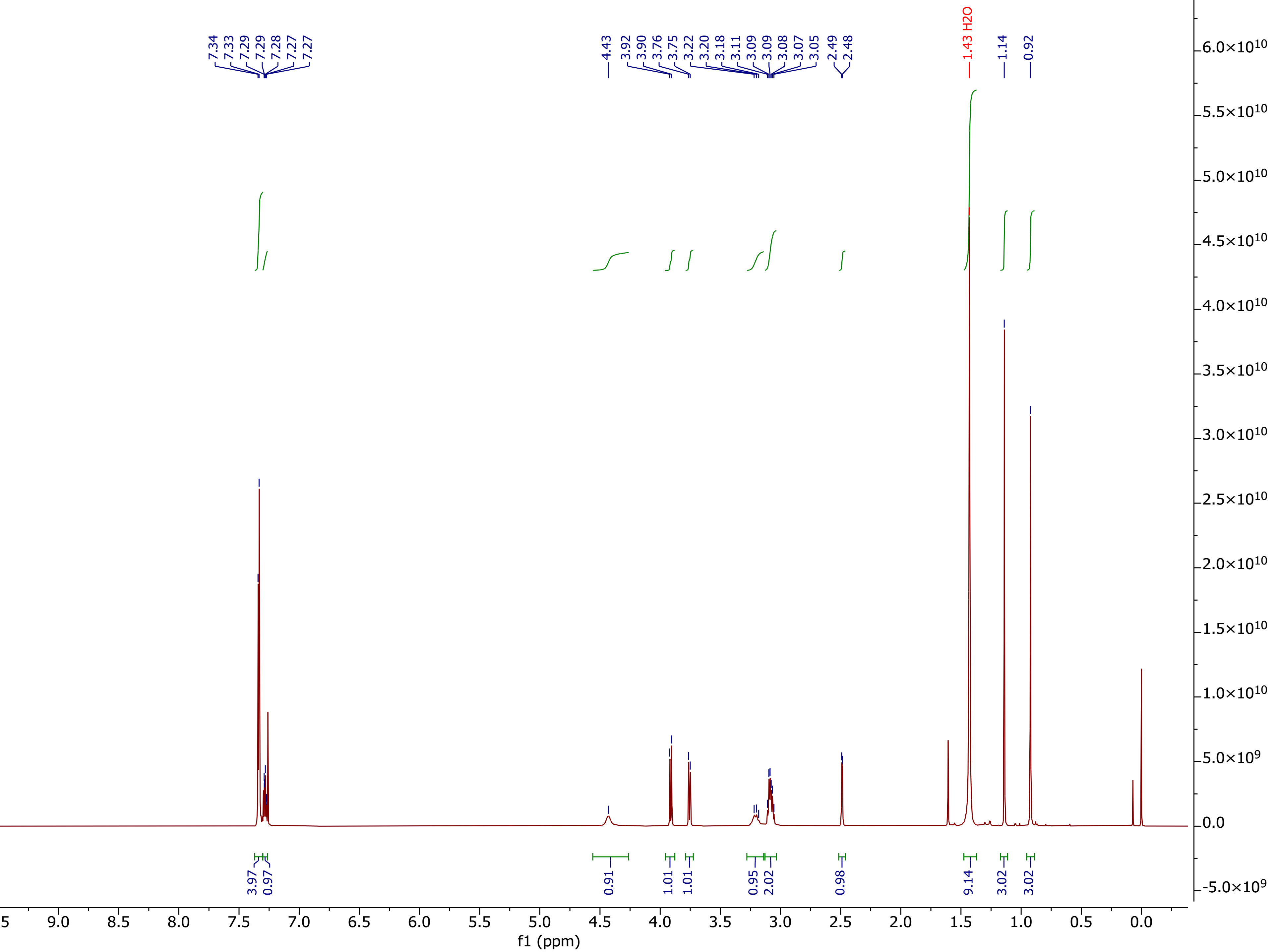
7.34
7.33
7.29
7.29
7.28
7.27
7.27
4.43
3.92
3.90
3.76
3.75
3.22
3.20
3.18
3.11
3.09
3.09
3.08
3.07
3.05
2.49
2.48
1.43 H2O
1.14
0.92
3.97
0.97
0.91
1.01
1.01
0.95
2.02
0.98
9.14
3.02
3.02
f1 (ppm)

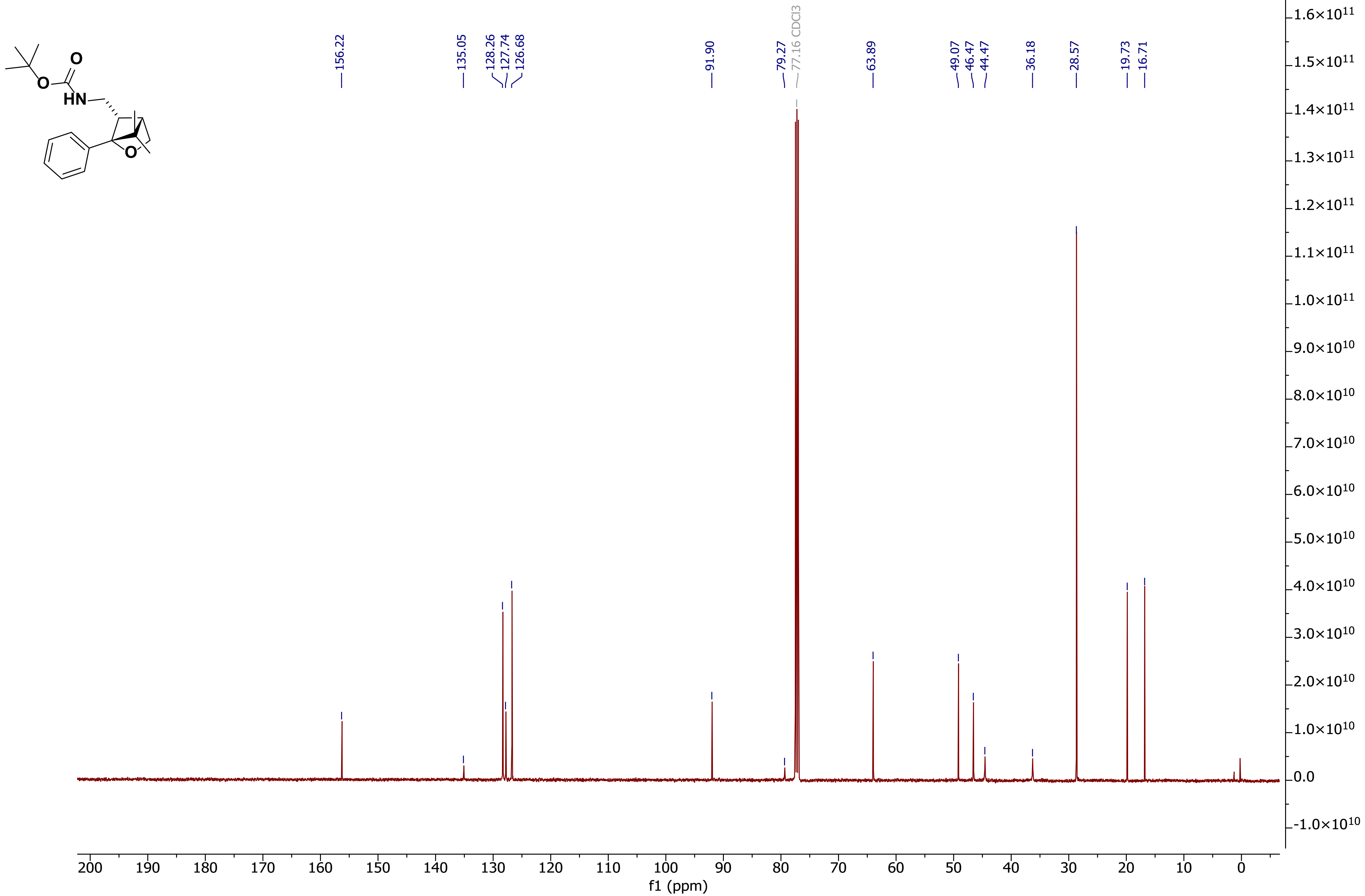

O
O
HN
O
156.22
135.05
128.26
127.74
126.68
91.90
79.27
77.16 CDCl3
63.89
49.07
46.47
44.47
36.18
28.57
19.73
16.71
1.6×10^11
1.5×10^11
1.4×10^11
1.3×10^11
1.2×10^11
1.1×10^11
1.0×10^11
9.0×10^10
8.0×10^10
7.0×10^10
6.0×10^10
5.0×10^10
4.0×10^10
3.0×10^10
2.0×10^10
1.0×10^10
0.0
-1.0×10^10
200 190 180 170 160 150 140 130 120 110 100 90 80 70 60 50 40 30 20 10 0
f1 (ppm)
